# PhytoMolecularTasteDB: An integrative database on the “molecular taste” of Indian medicinal plants

**DOI:** 10.1016/j.dib.2018.04.048

**Published:** 2018-04-21

**Authors:** Dorin Dragos, Marilena Gilca

**Affiliations:** a Medical Semiology Dept., Faculty of General Medicine, Carol Davila University of Medicine and Pharmacy, B-dul Eroilor Sanitari nr. 8, 050471 Bucharest, Romania; b Nephrology Clinic, University Emergency Hospital Bucharest, Bucharest, Romania; c Biochemistry Dept., Faculty of General Medicine, Carol Davila University of Medicine and Pharmacy, B-dul Eroilor Sanitari nr. 8, 050471 Bucharest, Romania

## Abstract

PhytoMolecularTaste database (PhytoMolecularTasteDB) described in the present work is related to the article “Main phytocompunds׳ tastes: a better predictor for the ethnopharmacological activities of medicinal plant than the phytochemical class?” (Dragos and Gilca, 2018) [1]. It includes a comprehensive list of plant derived tastants, as well as details on the “phyto-molecular taste” (PMT) (the combination of tastes resulted from the main tastants found in a medicinal plant). To collect the data, we searched publications in various databases and journals by using relevant keywords. Wherever necessary, manual search of lacking information was also performed in several books. We then extracted the reported phytoconstituents and PMT of all the ayurvedic medicinal plants included in DB. Data were compiled in Excel. In total, PhytoMolecularTasteDB includes 431 ayurvedic medicinal plants, 94 EPAs, 223 phytochemical classes, and 438 plant-derived tastants.

**Specifications table**

**Table:** 

Subject area	*Biology*
More specific subject area	*Ethnopharmacology*
Type of data	*Table, text file, figure, Excel database*
How data was acquired	*Literature search of published data*
Data format	*Coded, filtered, analyzed*
Experimental factors	*Data on the plant derived tastants found in Indian medicinal plants*
Experimental features	*Publications with data searched using keywords in databases. Keywords: English and Latin names of medicinal plants,* “composition”, “phytochemical”, “phytocompound” “chemical compounds”, “ingredients” *name of phytochemicals, “taste”,” bitter/bitterness/tikta”,” sweet/sweetness/madhura”,” sour/sourness/amla”, “astringent/astringency/kashaya”,” pungent/pungency/katu”, “salty/saltyness/lavana”,” sensory”,” organoleptic”, “tastant”, name of taste receptors (e.g. TAS2R) or* orosensation transducers (e.g. TRP- transient receptor potential channels)
Data source location	*Elsevier ScienceDirect, PubMed, Google Academic, Google Books, BitterDB, SweetenersDB, SuperSweet, HMDB, FoodDB*
Data accessibility	*Data is within this article*

**Value of the data**

• First database on plant-derived tastants and “molecular taste” of ayurvedic medicinal plants
• Data scattered in various publications, databases, gathered in one place.
• PhytoMolecularTasteDB will facilitate future research on the patterns that shape the traditional medical knowledge.
• PhytoMolecularTasteDB can be used in cross-cultural comparative studies on medicinal plants and phyto-molecular taste.
• PhytoMolecularTasteDB can inform pharmacologists about new therapeutic agents from ethnomedicine and potential biological activities of plant-derived tastants.

## 1 Data

PhytoMolecularTasteDB presents an inventory of plant-derived tastants and “phyto-molecular taste” (PMT) of ayurvedic medicinal plants. In total, PhytoMolecularTaste DB includes 431 ayurvedic medicinal plants with their Sanskrit and Latin name, 94 EPAs, 223 phytochemical classes, and 438 plant-derived tastants. Data are analysed in a related article [1].

## 2 Experimental design, materials and methods

We have build, for the first time to our knowledge, a mixed database (PhytoMolecularTasteDB) on ayurvedic medicinal plants, by integrating modern data (medicinal plant composition, phytochemical taste) with traditional data (ethnopharmacological activities of plant). We elaborated PhytoMolecularTasteDB in three steps.

### 2.1 First step (ethnopharmacological data)

We have included in our analysis all the plants (in total 454 plants) described (in dedicated monographs) in one recognized text of ayurvedic medicine (Pandey׳s “Dravyaguna vijnana”) [2], for which a clear description of the EPAs (traditionally called *karman*) was available.

Here is the distribution among the various botanical families of medicinal plants included in our database (the number of plants in each family is indicated in the paranthesis):

*Acanthaceae* (5)*, Agaricaceae* (1)*, Alangiaceae* (1)*, Amaranthaceae* (5)*, Amaryllidaceae* (2)*, Anacardiaceae* (8)*, Annonaceae* (2)*, Apiaceae* (12)*, Apocynaceae* (8)*, Araceae* (5)*, Arecaceae* (6)*, Aristolochiaceae* (2)*, Asclepiadaceae* (6)*, Asparagaceae* (2)*, Asphodelaceae* (1)*, Asteraceae* (19)*, Basellaceae* (1)*, Berberidaceae* (1)*, Betulaceae* (1)*, Bignoniaceae* (3)*, Bixaceae* (1)*, Boraginaceae* (4)*, Brassicaceae* (7)*, Bromeliaceae* (1)*, Burseraceae* (3)*, Caesalpiniaceae* (2)*, Calophyllaceae* (3)*, Cannabinaceae* (1)*, Capparaceae* (2)*, Capparidaceae* (2)*, Caricaceae* (1)*, Celastraceae* (2)*, Ceratophyllaceae* (1)*, Cleomaceae* (1)*, Clusiaceae* (2)*, Colchicaceae* (1)*, Combretaceae* (5)*, Convolvulaceae* (5)*, Crassulaceae* (1)*, Cucurbitaceae* (16)*, Cupressaceae* (1)*, Cyperaceae* (2)*, Dilleniaceae* (1)*, Dioscoreaceae* (1)*, Dipterocarpaceae* (3)*, Ebenaceae* (1)*, Elaeocarpaceae* (1)*, Ephedraceae* (1)*, Euphorbiaceae* (7)*, Fabaceae* (49)*, Fagaceae* (1)*, Flacourtiaceae* (1)*, Gentianaceae* (3)*, Iridaceae* (2)*, Juglandaceae* (1)*, Lamiaceae* (8)*, Lauraceae* (3)*, Lecythidaceae* (2)*, Liliaceae* (5)*, Linaceae* (1)*, Loganiaceae* (1)*, Loranthaceae* (1)*, Lythraceae* (2)*, Magnoliaceae* (1)*, Malvaceae* (18)*, Marsileaceae* (1)*, Meliaceae* (4)*, Menispermaceae* (5)*, Moraceae* (10)*, Moringaceae* (1)*, Musaceae* (1)*, Myricaceae* (1)*, Myristicaceae* (1)*, Myrsinaceae* (1)*, Myrtaceae* (3)*, Nyctaginaceae* (2)*, Nymphaeaceae* (2)*, Oleaceae* (5)*, Orchidaceae* (1)*, Oxalidaceae* (2)*, Paeoniaceae* (1)*, Pandanaceae* (1)*, Papaveraceae* (3)*, Parmeliaceae* (1)*, Pedaliaceae* (1)*, Pinaceae* (3)*, Piperaceae* (5)*, Plantaginaceae* (1)*, Plumbaginaceae* (1)*, Poaceae* (14)*, Polygonaceae* (1)*, Portulacaceae* (1)*, Pteridaceae* (2)*, Punicaceae* (1)*, Putranjivaceae* (1)*, Ranunculaceae* (7)*, Rhamnaceae* (2)*, Rosaceae* (6)*, Rubiaceae* (7)*, Rutaceae* (7)*, Salicaceae* (4)*, Santalaceae* (1)*, Sapindaceae* (3)*, Sapotaceae* (3)*, Saxifragaceae* (1)*, Scrophulariaceae* (3)*, Simarubaceae* (2)*, Smilacaceae* (1)*, Solanaceae* (9)*, Sterculiaceae* (2)*, Styracaceae* (1)*, Symplocaceae* (1)*, Trapaceae* (1)*, Ulmaceae* (1)*, Valerianaceae* (2)*, Verbenaceae* (7)*, Violaceae* (1)*, Vitaceae* (2)*, Zingiberaceae* (10)*, Zygophyllace* (2).

### 2.2 Second step (chemical composition data)

We collected the information about the main chemical constituents of the plants from several acknowledged books on ayurvedic/Indian medicinal plants [2], [3], [4], [5], [6] and by performing a systematic search in PubMed (1077 references, see Appendix B) using as keywords the English or Latin name of the medicinal plants and “composition”, “phytochemical”, “phytocompound” “chemical compounds”, “ingredients”. For a given plant, we considered as major the following constituents:

1) phytocompounds mentioned in Ayurveda/Indian Materia Medica or Pharmacopoeia to the herbal chemical composition;
2) phytocompounds to which one of the following terms “main”, “principal”, “major” were attributed, as revealed by the 1077 scientific references (see Appendix B);
3) phytocompounds to which one of the scientifically recognized pharmacodynamic actions of that plant is currently attributed, as revealed by the 1077 scientific references (see Appendix B).

In order to increase the reliability of information included in our database, we introduced the double checking criterion: only the phytochemicals with at least two references have been included.

These sources provided useful information for most, but not all the ayurvedic herbs in Pandey׳s “Dravyaguna vijnana” [2], therefore from the initial 454 plants we were left with 431 plants for which a clear description of both the spectrum of EPAs and of the chemical composition was available.

### 2.3 Third step (plant-derived tastants data)

**Fig. 1:**
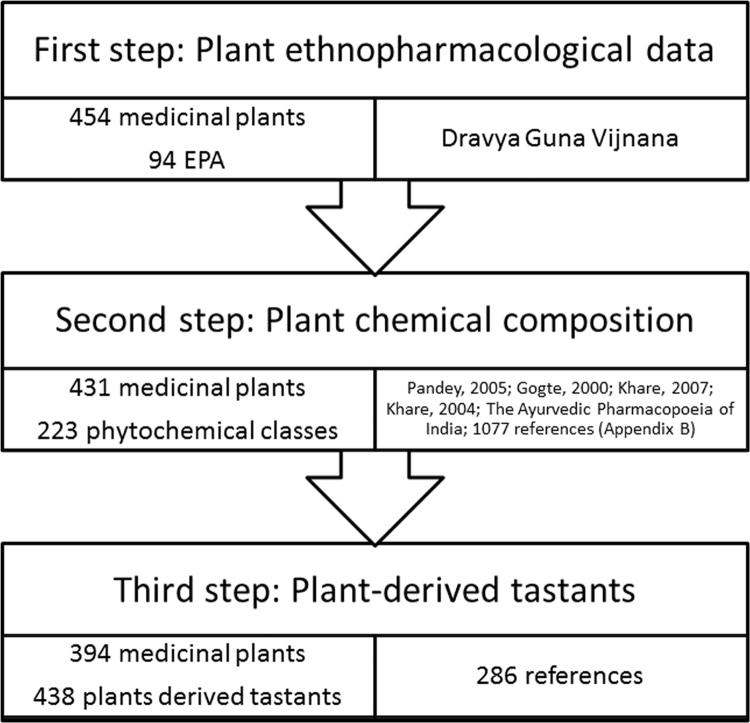
PhytoMolecularTasteDB building steps.

For only 394 plants were we able to define a taste based on the chemical constituents (see Appendix A, Table A.6 for a list of plant-derived tastants, and Table A.7 for a list of medicinal plants included in the study, or PhytoMolecularTasteDB Excel). The main resources used to identify the tastes (sanskr. *rasa*) of various phytochemicals were: 1) BitterDB, a database on bitter compounds [7]; 2) SweetenersDB and SuperSweet databases on sweet compounds [8], [9]; 3) HMDB: the Human Metabolome Database [10]; 4) FooDB, the world׳s largest electronic resource on food constituents, chemistry and biology (http://foodb.ca/) [11]; 3) PubChem [12]; 4) PubMed database; 5) Elsevier ScienceDirect database. Eventually, we performed a systematic search on the potential taste of all the phytoconstituents listed during the second step, using Google Academic and Google Books as search engine. The keywords used for our search were: English and Latin names of medicinal plants, name of phytochemicals, taste, English and Sanskrit names of tastes, orosensations or *rasas* (bitter/bitterness or *tikta*, sweet/sweetness or *madhura*, sour/sourness or *amla*, astringent/astringency or *kashaya*, pungent/pungency/tingling/burning/cooling or *katu*, salty/saltyness or *lavana*, sensory, organoleptic, tastant, name of taste receptors (e.g. TAS2R) or orosensation transducers (e.g. TRP- transient receptor potential channels). Phytochemicals were included in the PhytoMolecularTasteDB only if they were reported as having a certain taste or if they displayed experimentally activatory potential on at least one taste receptor or orosensation transducer. Supplementary information was also obtained by manual search in various books (e.g. “Bitterness in Foods and Beverages”, edited by RL Rouseff) [13]. In order to increase the reliability of information included in our phyto-tastants database, we used again the double checking criterion: we aimed, as much as the available scientific data allowed this, to have at least two supportive references for each plant-derived tastant. More than 70% of the phytoconstituent tastants found in PhytoMolecularTasteDB fulfilled this criterion, so that the average number of references per tastant was higher than 2 (Fig. 1).
