# Supplementary material for: PhytoMolecularTasteDB: An integrative database on the “molecular taste” of Indian medicinal plants
Source: Data Brief. 2018 Apr 21;19:1237–41. doi: 10.1016/j.dib.2018.04.048 (PMC6141601; doi:10.1016/j.dib.2018.04.048)
Supplement: Supplementary material [file mmc2.docx]

**Appendix A**

# Table A.1. The initial 223 categories of active principles.

acetylene; acridone alkaloid; alcohol; aldehyde; aliphatic aldehyde; aliphatic amine; aliphatic ketone; alkaloid; amide; amide alkaloid; amine; amine alkaloid; amino acid; anthocyan; anthraquinone; aporphine alkaloid; aromatic alcohol; aromatic aldehyde; aromatic amine; aromatic ester; aromatic hydrocarbon; aromatic ketone; ascorbic acid; aurone; benzochromone; benzodioxole; benzophenone; benzopyran; benzoquinolizidine alkaloid; benzoxazinoid; benzylisoquinoline alkaloid; benzyltetrahydroisoquinoline alkaloid; biflavanone; biflavone; biflavonoid; bisbenzylisoquinoline alkaloid; C6-C1 compounds; C6-C2 compounds; C6-C3 compounds; cannabinoid; carbolinebenzoquinolizidine alkaloid; carbolineindole alkaloid; cardenolide; cardiac glycoside; carotene; chalcone; choline; chromone; coumarin; coumarone; coumestan; cyanogenic glycosides; cyclic polyol; cyclitol carboxylic acid; cyclopropan; cyclopropene high aliphatic acid; diketone; diterpene; diterpene alkaloid; diterpene alcohol; diterpene ester; diterpene ketone; diterpene lactone; epoxide; ergolineindole alkaloid; essential oil; ester; ester of cyclic polyol; ester of cyclopropene high aliphatic acid; ester of high aliphatic acid; ester of high aliphatic alcohol; fatty oil; flavanol; flavanone; flavanonol; flavone; flavones and anthocyans; flavones and chalcones; flavones, chalcones and anthocyans; flavonoid; flavonol; flavonolignan; folic acid; furanocoumarin; furanoditerpene; furanolactone; furofuranlignan; furoquinoline alkaloid; glucosinolate; glyceride; glycoalkaloid; glycoprotein; gum; hemiterpene; high aliphatic acid; high aliphatic alcohol; high aliphatic aldehyde; high aliphatic ester; high aliphatic hydrocarbon; high aliphatic ketone; hydrocarbon; imidazole alkaloid; indole alkaloid; indolizidine alkaloid; inulin; iridoid; isoflavanone; isoflavone; isoflavonoid; isoquinoline alkaloid; ketolignan; ketone; ketonealdehyde; ketonesteroid; ketonesterol; lactone; lectin; lignan; lipid; low aliphatic alcohol; meroterpene; mineral salts; monoterpene; monoterpene alkaloid; monoterpene alcohol; monoterpene aldehyde; monoterpene ester; monoterpene hydrocarbon; monoterpene indole alkaloid; monoterpene ketone; monoterpene oxide; monoterpene peroxide; monoterpene phenol; monovalent ketone; mucilage; naphthoquinone; naphthyridine alkaloid; nitroderivative; organic acid; oxazolidinone alkaloid; pectin; pentacyclic triterpene; peptide; phenanthraindolizidine alkaloid; phenanthrene; phenanthridine alkaloid; phenol; phenolic acid; phenolic lipid; phenylalanine-derived alkaloid; phenyl-methyl eters; phospholipid; phosphor compound; phthalid tetrahydroisoquinoline alkaloid; phytic acid; piperidine alkaloid; polyketide; polyphenol; polyunsaturated high aliphatic acid; proanthocyan; protein; proteinogenic aminoacid;; proto alkaloid; pterocarpan; purine alkaloid; pyrazoline alkaloid; pyridine alkaloid; pyridone alkaloid; pyrrolidine alkaloid; pyrrolizidine alkaloid; quassinoid; quinazoline alkaloid; quinoline alkaloid; quinolizidine alkaloid; quinolone alkaloid; quinone; quinuclidine alkaloid; resin; rotenoid; saccharide; salicylate; saponin; sesquiterpene; sesquiterpene alkaloid; sesquiterpene alcohol; sesquiterpene aldehyde; sesquiterpene ester; sesquiterpene hydrocarbon; sesquiterpene ketone; sesquiterpene lactone; sesquiterpene phenol; short aliphatic acid; spermidine alkaloid; steroid; steroid alkaloid; steroid lactone; steroid saponin; sterol; stilbene; sugar acid; sulphur compound; tannin; terpene; terpene alkaloid; tetracyclic triterpene; tetranortriterpenoid; toxalbumin; tricyclic triterpene; triglyceride; triterpene; triterpene alcohol; triterpene saponin; tropane alkaloid; urea; uronide; vitamin D; vitamin E; vitamin K; vitamin P; vitamins; vitamins B; withanolide; xanthone.

# Table A.2. The 109 categories of active principles after eliminating to broad or overlapping categories.

acridone alkaloid; aliphatic aldehyde; aliphatic amine; aliphatic ketone; amide alkaloid; amine alkaloid; amino acid; anthocyan; anthraquinone; aporphine alkaloid; aurone; cardenolide; carotene; chalcone; choline; chromone; coumarin; coumarone; cyanogenic glycosides; cyclic polyol; diterpene alcohol; diterpene alkaloid; diterpene ester; diterpene ketone; diterpene lactone; aromatic alcohol; aromatic ester; monoterpene alcohol; monoterpene aldehyde; monoterpene ester; monoterpene hydrocarbon; monoterpene ketone; monoterpene oxide; monoterpene peroxide; monoterpene phenol; phenyl-methyl eters; sesquiterpene ester; sesquiterpene hydrocarbon; sesquiterpene ketone; ester of cyclic polyol; flavanol; flavanone; flavanonol; flavone; flavonol; furanoditerpene ; furoquinoline alkaloid; glucosinolate; glycoalkaloid; gum; high aliphatic acid; ester of high aliphatic acid; high aliphatic alcohol; high aliphatic aldehyde; high aliphatic hydrocarbon; high aliphatic ketone; imidazole alkaloid; indole alkaloid; indolizidine alkaloid; iridoid; isoflavanone; isoflavone; isoquinoline alkaloid; ketonesteroid; lignan; low aliphatic alcohol; mineral salts; mucilage; naphthoquinone; naphthyridine alkaloid; pentacyclic triterpene ; peptide; phenanthridine alkaloid; phospholipid; piperidine alkaloid; proanthocyan; protein ; pterocarpan; purine alkaloid; pyrazoline alkaloid; pyridine alkaloid; pyridone alkaloid; pyrrolidine alkaloid; pyrrolizidine alkaloid; quinazoline alkaloid; quinoline alkaloid; quinolizidine alkaloid; quinolone alkaloid; resin; rotenoid; salicylate; short aliphatic acid; spermidine alkaloid; sesquiterpene alcohol; sesquiterpene aldehyde; sesquiterpene alkaloid; sesquiterpene lactone; sesquiterpene phenol; steroid alkaloid; steroid saponin; tannin; tetracyclic triterpene ; tetranortriterpenoid; tricyclic triterpene ; triterpene alcohol; tropane alkaloid; uronide; withanolide; xanthone.

# Table A.3. The 46 categories of active principles after eliminating those with a column marginal sum below 100.

aliphatic amine; amino acid; anthocyan; anthraquinone; aporphine alkaloid; carotene; chalcone; choline; coumarin; monoterpene alcohol; monoterpene aldehyde; monoterpene hydrocarbon; monoterpene ketone; monoterpene oxide; monoterpene phenol; sesquiterpene hydrocarbon; flavanone; flavone; flavonol; glucosinolate; gum; high aliphatic acid; ester of high aliphatic acid; high aliphatic alcohol; high aliphatic hydrocarbon; indole alkaloid; iridoid; isoflavone; isoquinoline alkaloid; lignan; mineral salts; mucilage; pentacyclic triterpene; protein; pyridine alkaloid; quinoline alkaloid; resin; salicylate; short aliphatic acid; sesquiterpene alcohol; sesquiterpene lactone; steroid alkaloid; steroid saponin; tannin; tetracyclic triterpene; xanthone;

# Table A.4. The 18 categories of active principles after eliminating those with a column marginal sum below 300.

amino acid; anthocyan; coumarin; monoterpene alcohol; monoterpene hydrocarbon; monoterpene phenol; flavone; flavonol; gum; high aliphatic acid; high aliphatic alcohol; mucilage; pentacyclic triterpene; protein; resin; short aliphatic acid; tannin; tetracyclic triterpene;

# Table A.5. The 8 categories of active principles after eliminating those with a column marginal sum below 500.

amino acid; flavone; flavonol; high aliphatic acid; pentacyclic triterpene ; protein ; resin; tannin.

# Table A.6. Phytocompounds classified by ayur-taste (rasa)

|  | Phytocompounds |
| --- | --- |
| Kashaya (astringent) | anthocyan [1]  cyanidin- [1, 2]  cyanidin-3-glucoside (chrysanthemin) [1, 2]  chrysanthemin  cyanidin-3-rutinoside [1, 2]  monoglycosides of cyanidin [1, 2]  delphinidin [1]  ellagitannin [3]  gallotannin [4, 5]  leucocyanidin [5–7]  leucopelargonidin [6]  malvidin [1, 8]  3-monoglucofuranoside of 7-O-methyl leucopelargonidin [9]  petunidin [1, 8]  phlobatannin [10]  punicalagin [11]  tannin [4, 10, 12, 13]  hydroxybenzoic acids [14, 15]  phenolic acids [14–16]  gallo tannic (tannic acid) [17, 18]  tannic acid  corilagin [17, 18]  kaempferol 3-O-beta-galactoside [19]  kaempferol-3-O-α-lrhamnopyranosyl-(1 → 6)-β-d-glucopyranoside [19]  dicaffeoylquinic [20] |
| Kashaya (astringent) + tikta (bitter) | afzelechin [21]  biochanin A [22–25]  biochanin A-7-glucoside [25]  catechin [12, 14, 24, 26–28]  daidzein [12, 28][12, 24–26]  daidzin [12, 29]  ellagic [3, 30]  epiafzelechin [21]  epicatechin [24–26, 28, 31, 32]  epigallocatechin [32, 33]  epicatechin gallate [32, 33]  epigallocatechin gallate [24, 32–34]  fisetinidol [35]  formononetin-7-O-beta-D-glucopyranoside (ononin) [25]  ononin  gallate [4, 14, 36]  galloyl [5, 24, 37]  pentagalloyl glucose [4, 24, 38]  genistein [14, 26, 31, 39, 40]  genistin [25, 29, 31, 41, 42]  kaempferol [21, 22, 24, 26, 43]  kaempferol-3-glucoside (kaempferol-3-O-β-D-glucopyranoside) [21]  kaempferol-3-O-β-D-glucopyranoside  kaempferol 3-O-rutinoside [21]  malvidin diglucoside [1, 8, 44]  naringenin [22, 24, 26, 28, 31, 45]  pelargonidin [1, 22, 46]  procyanidin [14, 25, 27, 40, 47]  quercetin [24, 26, 28, 48]  rutin [21, 48, 49] |
| Kashaya (astringent) + tikta (bitter) + amla (sour) | chlorogenic (caffeoylquinic) [20] |
| Kashaya (astringent) + tikta (bitter) + amla (sour) + madhura (sweet) | gentisic acid [51]  protocatechuic [14, 51, 52]  3,4-dihydroxybenzoic acid [51, 52] |
| astringent+umami | rubemamine [53, 54] |
| astringent + pungent + bitter + sour + sweet | salicylic acid [51, 55, 56] |
| bitter | absinthin [25–27, 57]  absintholide [58, 59]  acoretin [48, 60]  acorone [61, 62]  ailanthone [61, 63]  ajmalicine (raubasine) [64]  raubasine  alkaloid [12]  alkylamine [25, 65]  aloin [26, 48, 66–68]  amarogentin [25–27, 69, 70]  amaropanin [66, 69, 71]  amaroswerin [66, 71]  amygdalin [25–27, 68]  andrographolide [26, 61, 70, 72]  angelicin [17, 73]  anthraquinone [74–76]  apigenin [22, 24, 26]  apterin [77]  arbutin [26, 61, 68, 78]  arctigenin [79, 80]  arginine [81–84]  aristolochic acid (aristolochin) [26, 27, 68, 72, 85]  aristolochin  arjunetin [61]  asparagine (L-asparagine) [84, 86]  atisine [48, 87]  atropine [48, 85, 88, 89]  aucubin [61, 90, 91]  aurantiamaric acid [48]  azadirachtin [92, 93]  baicalein [45]  benzamide [24, 26, 68]  berberine [26, 43, 45, 57]  berbamine [45]  bergapten [17, 71, 94]  bergenin (cuscutin) [48]  cuscutin  boldine [48, 95–97]  bonducin [61, 98, 99]  bitter principle  brucine [26, 27, 68, 100, 101]  butein [22, 24]  caesalpin [61, 102]  caffeine [24, 36, 48]  calotropin [61, 103]  cardenolide [25]  carpaine [48, 104, 105]  catechol [65]  chalcone [24, 26]  chicoriin [48, 61]  chiratin [48]  choline [25]  chrysin [24, 26, 106]  chrysoeriol [24, 26]  cedrin (6-methyldihydromyricetin) [107]  6-methyldihydromyricetin  cinchonine [26, 43, 48, 88]  cinchonidine [48, 107, 108]  citronellol [109, 110]  citrulline (citrullin) [83]  citrullin  clerodin [102, 111]  cocaine [26, 112, 113]  Codeine [64, 97]  colchicine [26, 48, 68]  colubrine [26]  columbin [25, 26, 66, 114, 115]  conessine [26, 48]  costunolide [100]  coumarin [24, 26]  cryptopine [48]  cucurbitacin [25, 26, 116]  cyanidin [22, 24]  dehydroandrographolide [45, 117]  digitalin [48, 107]  digitonin [68]  dihydrotaraxine acid glucopyranoside [118]  13,18-dehydroexcelsin [61]  11β,13-dihydrolactucin [119, 120]  11,13-dihydrotaraxinic acid-beta-glucopiranoside [121, 122]  diosbulbin [123]  diosmetin [106]  ecboline (ergotoxin) [48, 124]  ergotoxin  echitamine [48]  echitenine [48]  ecliptasaponin [61]  emodin [61, 67]  eriocitrin [45]  eriodictyol [22, 24]  ergometrine (ergonovine) [64, 125]  ergonovine  ergotamine [64]  erythrocentaurin [17, 48, 66]  esculetin [48, 118, 126]  esculin [48, 78, 100, 118, 126]  falcarindiol [24, 68, 127]  falcarinol [127]  fenchone [26, 128]  fisetin [26]  flavanone [24, 26]  formononetin [24, 42]  fraxetin [48, 126, 129]  furanogermacrane [66]  fustin [22, 24]  gamma - fagarine [64]  genkwanin [22, 23]  gentianine [17, 26, 101]  gentianose [66]  gentiobiose [25, 26, 65, 130]  gentiopicroside (gentiopicrin) [17, 45, 66, 69, 71, 131]  gentiopicrin  gilenin [132, 133]  giloinin [132, 134]  glaucarubin [63, 130, 135]  glaucarubinone [63]  globularin [48]  glucobrassicin [31, 136]  glucosinolate (glycosinolate) [12, 118, 136, 137]  glycosinolate  gossypetin [22, 26]  gymnemagenin [138]  gymnemic acid [61, 138]  harmine [48, 64]  hederagenin (as saponin) [139]  helenalin [26, 140]  herbacetin [22, 24, 26]  7,4′-dihydroxyflavone [22]  histidine [26, 84, 141]  hydroquinine [48]  igasurine [48]  imperialin [48, 142]  indican [48, 143]  iridoid [144, 145]  isobutylamide [146]  isoleucine [26, 83, 84]  isoliquiritigenin [22]  isorhamnetin [22, 24, 26]  jatrorrhizine [45, 95]  juglone (5-hydroxy-1, 4-naphthoquinone) [147]  5-hydroxy-1, 4-naphthoquinone  lactone [25, 61, 65, 148]  lactucin [25, 66, 118, 120, 149]  lactucopicrin [25, 66, 118, 120, 149]  leucine (L-leucine) [26, 83, 84, 141]  ligstroside [118, 150]  limettin [151]  limonin [12, 25–27, 68, 114]  linamarin [26, 118, 152]  linolenic [26]  liquiritigenin [22, 24, 26]  loganic [58]  luteolin [24, 26, 106, 138]  lycoctonine [26, 48]  oleuropein [45, 100, 118, 153]  margosine [154, 155]  margosinolide [156]  matairesinol [118]  matairesinol monoglucoside [157, 158]  alpha-hederin (melanthin) [48, 159]  melanthin  melianone [160]  melongoside [161, 162]  menthofuran [100]  mesuol [163, 164]  methoxsalen [17, 26]  mollugogenol [165]  morin [22, 24]  moschamine [53]  myricetin [22–24]  naringin [27, 31, 68, 71]  N-Caffeoyltyramine [53]  neoandrographolide [61, 117, 166]  neohesperidin [68]  neral [26]  nerol [26]  N-feruloyltyramine (moupinamide) [53]  moupinamide  nicotinic acid [36, 64]  nimbin [68, 160, 167]  nimbinin [167]  nimbidin [167]  nobiletin [12, 23, 45]  noscapine [24, 26, 68, 101]  obacunone [114, 130, 168, 169]  oleandrin [48]  oleanolic [170–172]  palmarin [134, 168]  palmatine [95]  pantothen [26]  papaverine [24, 26, 68]  parthenolide [24, 26, 68]  peganine [64, 173]  L- phenylalanine [25, 26, 83, 84, 89, 141]  phloridzin [5, 26, 45, 130, 174]  phyllanthin [48]  picrocrocin [25, 26, 66]  picroside [45, 71, 175, 176]  picrotin [24, 100]  pimpinellin [17, 177, 178]  pinocembrin [22, 24]  porphyrine [48]  protodioscin [179, 180]  prunasin [181]  prunetin [22, 24, 26]  pseudoephedrine [48]  psoralen [17, 73, 182]  punarnavine [61, 183]  putranjivoside [184]  putranoside [184]  ranunculin [130]  rhamnetin [48]  rhododendrin [185]  quassinoid [17, 24–26, 57, 92, 130, 141]  quercetagetin [22, 23]  quercetin glycoside [186]  quercitrin [43, 48, 187]  quinidine [26, 43, 48]  quinine [24–26, 68]  quinone [147]  resveratrol [22, 24, 26]  riboflavin [68]  rotenoid [17]  salicin [26, 48, 68, 78, 138]  santamarin [100]  santonin [17, 26, 43, 48, 138]  scillaren [17, 26]  scopoletin [126]  scutellarein [22, 24, 26]  senecionine [26, 101]  sesquiterpene lactone [141, 188]  sitosterol [118, 121, 122]  skimmianine [64, 182]  solanine [68, 189–191]  solasonine [101, 190, 192, 193]  strychnine [26, 85, 100, 141]  sweroside [66, 194]  swertiamarin [25, 26, 66, 69, 131, 195]  sulfuretin [22, 24]  tamarindienal [58, 71, 196]  tangeretin [12, 23, 45]  taraxacin [48, 197]  taraxinic acid [138]  taraxinic acid glucopyranoside [118, 121, 122]  taraxerol [198]  taurine [26, 68, 100]  taxifolin [22, 24, 26]  taxine [48]  tetrandine [64, 199]  tetranortriterpenoid [25]  theobromine [26, 48, 85, 138]  theophylline [26, 45, 101]  thiamine [26, 68, 200]  thujone [24, 26, 68, 100]  tiliacorine [26]  tinosporine [201]  trichosanthin [71]  trigonelline [20, 50]  7,3',4'-trihydroxyflavone [22]  trimethoxyflavone [24]  tryptophan (L-tryptophan) [25, 26, 84, 141]  tyramine [64, 202]  tyrosine [27, 86, 203]  tyrosol [202]  umbelliferone [26]  ursolic [171]  vellarin [48]  wogonin [45]  α resorcylic acid [204]  p - aminobenzoic acid (4-aminobenzoic acid) [25, 204]  isoandrographanolide [166]  14-deoxy 11, 12- didehydroandrographolide [166]  andrograpanin [166]  xanthone (Roland et al., 2013; Wiener et al., 2012)  xanthotoxin (Fugmann et al., 2000)  xanthotoxol (Dreyer, 1966)  yohimbine (Meyerhof et al., 2010; Wiener et al., 2012) |
| Tikta (bitter) + amla (sour) | ophelic [17, 48, 71] |
| Tikta (bitter) + katu (pungent) | aconitine [48, 205]  acorin [48, 60]  belladonnine [48]  benzaldehyde [26, 206]  camphor [24, 68, 109, 207, 208]  hyoscyamine [48, 64, 209]  linoleic [118]  nicotine [26, 68]/ [48, 206]  rosmarinic [187, 210–212]  sinapine [25, 118]/[213]  sinigrin [12, 26, 68]/[213]  solanidine [68, 85, 118, 191, 192]/[48]  thebaine [48, 64]  turpethin [48] |
| Tikta (bitter) + katu (pungent) + madhura (sweet) | cubebin [214, 215]/ [107]  cinnamaldehyde [206, 216–220] |
| Tikta (bitter) + madhura (sweet) | alanine [25, 83, 84, 141, 221]  betaine [222, 223]/[224, 225]  glycyrrhizin [48, 218, 221]  hesperetin [24, 48]  β resorcylic acid [25, 204]  lysine [83, 84, 226]  mannose [65, 130, 227]  valine [83, 84, 86] |
| Tikta (bitter) +katu (pungent) + amla (sour) + madhura (sweet) | benzoic acid [51, 128, 204, 228] |
| Katu (pungent) | acetoxychavicol acetate [229–231]  ajoene [232]  allicin [232, 233]  aliin [170]  asarone [213]  bornyl acetate [109, 210]  cadinene [109, 210, 234]  capsaicin [216, 235–239]  δ-3-carene [210, 240]  carvacrol [219, 241]  carvone [210, 242]  cinnamate [210]  citral (geranial) [210, 213, 243]  citronellyl acetate (geranyl acetate) [213]  geranyl acetate  cuminaldehyde (cuminic aldehyde) [217, 244]  cuminic aldehyde  cymene [245]  dihydrocapsaicin [235–237]  dipropyl disulfide [232]  dithiines [232]  essential oil [210, 211]  geraniol [246–248]  6-gingediol [249, 250]  6-gingesulphonic acid [249]  gingerol [17, 71, 170, 216, 242, 251]  homocapsaicin [252]  homodihydrocapsaicin [235–237, 252]  isochavicine [247]  isopiperine [247]  isothiocyanate [12, 118, 174, 253]  limonene (dipentene) [245, 246]  dipentene  menthol [210]  monoterpene [246, 254]  norhydrocapsaicin [252]  linalool [248]  paradol [249, 255]  pellitorine [54, 256]  phellandrene [210, 246]  pinene [210, 246]  piperanine [213, 247, 257]  piperine [105, 130, 216, 217, 258]  piperolein [247, 259]  piperylene [258]  sesquiphellandrene [213]  shogaol [71, 170, 251, 260]  sabinene [210, 261, 262]  sinalbin [213]  sulphur containing [128, 213, 232]  terpinene [210]  terpineol [210, 245]  12-O-tetradecanoylphorbol-13-acetate? skin, eye irritant [263]  thiocyanate [253]  vanillin [206, 247, 264]  volatile oil [210, 211]  zingerone [71, 216, 265–268] |
| Katu (pungent) + amla (sour) | angelic acid [214] |
| Katu (pungent) + madhura (sweet) | cineol [213, 269–271] [206]  eugenol [206, 213, 217, 219, 242, 247] |
| Lavana (salty) + tikta (bitter) | minerals, mineral matter [272, 273] |
| Amla (sour) | acetic [40, 218, 274]  ascorbic acid  citric [40, 130, 214, 218, 274]  fumaric [218, 274, 275]  galacturonic [40]  lactic [40, 218, 274]  malic [40, 214, 218, 274]  oxalic [218]  pyruvic [206, 218]  quinic acid [218]  succinic [40, 218, 274]  tartaric [40, 214, 218, 274] + [4]  vitamin C  phytic [206]  formic [206]  butyric [274]  capric [274]  caproic [274] |
| Madhura (sweet) | abrusoside A, B and C [218, 276]  arabinogalactan [277]  arabinose [214]  dulcitol [214, 218]  erythritol [277, 278]  fructose [218, 221]  galactose [221, 279]  glucose [218, 221]  glycine [27, 83, 84, 221]  inositol [218, 221]  inulin [58, 226]  maltose [279]  mannan [280]  mannitol [206, 277]  meso-inositol [281]  neoastilbin [130]  phloroglucinol [221]  pinitol [282]  proline [83, 84, 86, 226]  raffinose [221, 279]  rhamnose [130, 138, 221]  sorbitol [221, 277]  sucrose [221, 283]  threonine [84, 86, 221, 284]  xylose [221]  phytol [206]  gossypol [206]  lanosterol [206]  tocopherol [206]  phytosterol [206]  amylopectin, amylose, carbohydrate, saccharide, starch, sugar, sugars |
| Madhura (sweet) + amla (sour) | serine [84, 86]  anthranilic acid [138, 206, 221] |
| Madhura (sweet) + umami | glutamine [86] |
| umami/amla (sour) + tikta (bitter) + lavana (salty) | glutamic acid [285, 286]/ [84, 86] |
| Umami/amla (sour) + tikta (bitter) | aspartic acid [285, 286]/ [84, 86] |
| Umami + madhura (sweet) + tikta (bitter) | methionine [84, 86] |

Table A.7. Medicinal plants included in our study (in the alphabetical order of their latin names)

| Sanskrit name | Latin name | Family |
| --- | --- | --- |
| talisa | Abies webbiana Lindle. syn. Abies spectabilis (D. Don.) Spach. | Pinaceae |
| pisacakarpasa | Abroma augusta Linn. f. | Sterculiaceae |
| gunja | Abrus precatorius Linn. | Fabaceae |
| atibala | Abutilon indicum G. Don. | Malvaceae |
| khadira | Acacia catechu Willd. syn. Mimosa catechu L.f. | Fabaceae |
| irimeda | Acacia farnesiana Willd. | Fabaceae |
| babbula | Acacia nilotica ssp. indica (Benth.) Breman. Acacia arobica Willd. | Fabaceae |
| apamarga | Achyranthes aspera Linn. | Amaranthaceae |
| vatsanabha | Aconitum ferox wall. ex Seringe. | Ranunculaceae |
| ativisa | Aconitum heterophyllum wall. | Ranunculaceae |
| prativisa | Aconitum palmatum D. Don. | Ranunculaceae |
| vaca | Acorus calamus Linn. | Araceae |
| vasa | Adhatoda vasica Nees. | Acanthaceae |
| mayurasikha | Adiantum caudatum Linn. | Pteridaceae |
| hamsapadi | Adiantum lunulatum Burm. | Pteridaceae |
| haridru | Adina cordifolia Benth. & Hooker | Rubiaceae |
| bilva | Aegle marmelos Corr. | Rutaceae |
| goraksaganja | Aerva lanata Juss. | Amaranthaceae |
| chhatraka | Agaricus campestris Linn. Psalliota Campestris Linn. | Agaricaceae |
| katvanga -aralu | Ailanthus excelsa Roxb | Simarubaceae |
| ankota | Alangium salviifolium (Linn. f.) Wang. Alangium lamarkii Thw. | Alangiaceae |
| sirisa | Albizia lebbeck Benth. | Fabaceae |
| yavasa -yavasaka | Alhagi maurorum Medik. syn. Alhagi camelorum Fisch. Alhagi pseudalhagi (Biab.) Desv. | Fabaceae |
| palandu | Allium cepa Linn. | Liliaceae |
| rasona | Allium sativum Linn. | Liliaceae |
| manakanda | Alocasia indica (Roxb.) Schott. | Araceae |
| kumari | Aloe vera Tourn. ex Linn syn. Aloe barbadensis Mill. | Asphodelaceae |
| malayavaca | Alpinia galanga (L.) Willd. syn. Languas galanga (L.) Stuntz | Zingiberaceae |
| saptaparna | Alstonia scholaris R. Br. | Apocynaceae |
| matsyaksaka | Alternanthera sessilis (Linn.) R. Br. | Amaranthaceae |
| khatmi | Althaea officinalis Linn | Malvaceae |
| tandulyya | Amaranthus spinosus Linn. | Amaranthaceae |
| brhadela | Amomum subulatum Roxb. | Zingiberaceae |
| surana | Amorphophallus campanulatus Blume. | Araceae |
| kajutaka | Anacardium occidentale Linn. | Anacardiaceae |
| akarakarabha | Anacyclus pyrethrum DC. | Asteraceae |
| panasi -ananasa | Ananas comosus (Linn.) Merr. | Bromeliaceae |
| kalamegha | Andrographis paniculata (Burm.b.) Wall ex. Nees. | Acanthaceae |
| satapuspa | Anethum sowa Kurz. syn. Peucedanum graveolens Linn. | Apiaceae |
| canda | Angelica archangelica Linn. Archangelica officinalis Hoffm. | Apiaceae |
| sitaphala | Annona squamosa Linn. | Annonaceae |
| dhava | Anogeissus latifolia Wall. | Combretaceae |
| kadamba | Anthocephalus cadamba (Roxb.) Miq. Anthocephalus chinensis (Lamk.) A. Rich. Anthocephalus indicus Miq. syn. | Rubiaceae |
| ajamoda | Apium graveolens Linn. Trachyspermum roxburghianum (DC) Sprague. Carum roxburghianum Pseucedanum graveolens (Linn) Hiers. | Apiaceae |
| mandapi | Arachis hypogaea Linn. | Fabaceae |
| puga | Areca catechu Linn. | Arecaceae |
| svarnaksiri | Argemone mexicana Linn. | Papaveraceae |
| vrddhadaruka | Argyreia speciosa Sweet. Operculina petaloidea (Choisy) Oststr. syn. Ipomoea petaloides Choisy. | Convolvulaceae |
| kitamari | Aristolochia bracteata Retz. | Aristolochiaceae |
| isvari | Aristolochia indica Linn. | Aristolochiaceae |
| kitamari yavani tiktapatra -afasantin | Artemisia absinthium Linn. | Asteraceae |
| kitamari yavani chuhara | Artemisia maritima Linn. | Asteraceae |
| panasa | Artocarpus heterophyllus Lam. syn. Artocarpus integra (Thunb.) Merrill. Artocarpus integrifolia auct. non L. | Moraceae |
| lakuca | Artocarpus lakoocha Roxb. | Moraceae |
| nala | Arundo donax Linn. | Poaceae |
| musali | Asparagus adscendens Roxb. | Liliaceae |
| satavari | Asparagus racemosus willd. | Liliaceae |
| kokilaksa | Hygrophila spinosa T. Anders. syn. Hygrophila auriculata (Schum.) Reine., Asteracantha longifolia Nees. | Acanthaceae |
| suci | Atropa belladona Linn. | Solanaceae |
| karmaranga | Averrhoa carambola Linn. | Oxalidaceae |
| nimba | Azadirachta indica A. Juss. | Meliaceae |
| brahmi | Bacopa monnieri (Linn.) Pennel. Bacopa monniera Wettst. Herpestis monniera (Linn.) H.B. & K. | Scrophulariaceae |
| ingudi | Balanites aegyptiaca (Linn.) Delile. Balanites roxburghii Planch. | Simarubaceae |
| danti | Baliospermum montanum Muell-Arg. | Euphorbiaceae |
| bola | Balsamodendron myrrha T. Nees. Commiphora myrrha (Nees.) Engl. | Burseraceae |
| saireyaka | Barleria prionitis Linn. | Acanthaceae |
| hijjala | Barringtonia acutangula Gaertn. | Lecythidaceae |
| upodika | Basella rubra Linn. | Basellaceae |
| kancanara | Bauhinia variegata syn. Phanera variegata (L.) Benth. | Fabaceae |
| kusmanda | Benincasa hispida (Thunb.) Cogn | Cucurbitaceae |
| daruharidra | Berberis aristata DC. | Berberidaceae |
| pasanabheda | Bergenia ligulata (Wall.) Engl. | Saxifragaceae |
| bhurja | Betula utilis D. Don. Syn. Betula bhojpattra Lindl. ex Wall. | Betulaceae |
| utangana | Blepharis edulis Pers. | Acanthaceae |
| kukundara | Blumea lacera Dc. syns. Conyza lacera Burm. f., Blumea subcapitata Dc. | Asteraceae |
| punarnava | Boerhaavia diffusa Linn. | Nyctaginaceae |
| vrsciva -sveta punarnava | Boerhaavia verticillata Poir. | Nyctaginaceae |
| salmali | Bombax ceiba L. syn. Salmalia malabarica (DC.) Schott & Endl | Malvaceae |
| tala | Borassus flabellifer Linn. | Araceae |
| sallaki | Boswellia serrata Roxb. | Burseraceae |
| sarsapa | Brassica campestris Linn. var. Sarson Prain. | Brassicaceae |
| rajika | Brassica juncea Czern. & Coss. syn. Synapsis juncea L. | Brassicaceae |
| krsna rajika -rajika bheda (krsna sarsapa) | Brassica nigra (Koch) Linn. | Brassicaceae |
| priyala | Buchanania lanzan spreng. syn. Buchanania latifolia Roxb. | Anacardiaceae |
| palasa | Butea monosperma (Linn.) Kuntze. | Fabaceae |
| kantaki karanja | Caesalpinia crista Linn. syn. Caesalpinia bonduc (L.) Roxb., Caesalpinia bonducella (L.) Flem. | Caesalpiniaceae |
| pattanga | Caesalpinia sappan Linn. | Caesalpiniaceae |
| adhaki | Cajanus cajan (Linn.) Mills | Fabaceae |
| vetraka | Calamus tenuis Roxb. | Arecaceae |
| priyangu | Callicarpa macrophylla Vahl. | Verbenaceae |
| punnaga | Calophyllum inophyllum Linn. | Calophyllaceae |
| arka | Calotropis gigantea (L.) Dryand. + Calotropis procera (Ait.) R. Br. | Asclepiadaceae |
| bhanga | Cannabis sativa Linn. | Cannabinaceae |
| karira | Capparis aphylla Roth. syns. Capparis decidua Edgew., Sodala decidua Forsk. | Capparaceae |
| rudanti | Capparis moonii Wight. | Capparaceae |
| himsra, vyaghranakhi | Capparis sepiaria Linn. Capparis zeylanica Linn. Capparis horrida Linn. f | Capparidaceae |
| katuvira -lanka | Capsicum annuum Linn. F Capsicum annuum Linn. var. acuminatum Fingh. | Solanaceae |
| kakadani (sakralata) | Cardiospermum halicacabum Linn. | Sapindaceae |
| kumbhika | Careya arborea Roxb. | Lecythidaceae |
| erandakarkati | Carica papaya Linn. | Caricaceae |
| karamarda | Carissa spinarum L. Syn. Carissa congesta W. syn. Carissa carandas Lodd. | Apocynaceae |
| kusumbha | Carthamus tinctorius Linn. | Asteraceae |
| krsna jiraka -karavi | Carum carvi Linn. | Apiaceae |
| cilhaka | Casearia tomentosa Roxb. Casearia graveolens Dalz. | Salicaceae |
| caksusya | Cassia absus Linn. | Fabaceae |
| markandika -svarnapatri | Cassia senna L. var. senna Brenan., syn. Cassia angustifolia Vahl., Senna officinalis Roxb. | Fabaceae |
| avartaki | Cassia auriculata Linn. | Fabaceae |
| aragvadha | Cassia fistula Linn. | Fabaceae |
| kasamarda | Cassia occidentalis Linn. | Fabaceae |
| cakramarda | Cassia tora Linn. | Fabaceae |
| devadaru | Cedrus deodara (Roxb.) Loud | Pinaceae |
| jyotismati | Celastrus paniculatus Linn | Celastraceae |
| mandukaparni | Centella asiatica (Linn.) Urban., syn. Hydrocotyle asiatica Linn. | Apiaceae |
| chhikkika | Centipeda orbicularis Lour. | Asteraceae |
| aranyajiraka | Centratherum anthelminticum O. Kuntze. | Asteraceae |
| saivala | Ceratophyllum demersum Linn. syn. Ceratophyllum verticillatum Roxb. | Ceratophyllaceae |
| canaka | Cicer arietinum Linn. | Fabaceae |
| kasani | Cichorium intybus Linn. | Asteraceae |
| kutikta -kunayana | Cinchona officinalis Linn. | Rubiaceae |
| karpura | Cinnamomum Camphora Nees & Eberm | Lauraceae |
| tvak | Cinnamomum zeylanicum Breyn. | Lauraceae |
| patha | Cissampelos pareira Linn. | Menispermaceae |
| asthisamharaka | Cissus quadrangularis Linn. Vitis quadrangularis Wall. | Vitaceae |
| indravaruni | Citrullus colocynthis Schard. | Cucurbitaceae |
| kalinda | Citrullus lanatus (Thumb.) Matsumara Citrullus vulgaris Schrad. syn. Colocynthis citrullus (Linn.) Kuntze. | Cucurbitaceae |
| dindisa | Citrullus vulgaris Var. fistulosus Duthie & Fuller. | Cucurbitaceae |
| nimbuka | Citrus aurantifolia (christm.) Swingle., syn. Citrus medica var. acida watt., Limonia aurantifolia Christon. = Citrus aurantiifolia | Rutaceae |
| naranga | Citrus aurantium Linn., Citrus reticulate Blance. | Rutaceae |
| bijapuraka | Citrus medica Linn. | Rutaceae |
| ajagandha | Cleome gynandra Linn. Gynandropsis gynandra Briq. Gynandropsis pentaphylla DC. Cleome viscosa Linn. | Cleomaceae |
| agnimantha | Clerodendrum phlomidis Linn. f. Premna integrifolia Linn. | Verbenaceae |
| bharngi | Clerodendrum serratum (Linn.) Moon | Verbenaceae |
| aparajita | Clitoria ternatea Linn. | Fabaceae |
| bimbi | Coccinia indica Wight. & Arn. Coccinia grandis (Linn.) Voigt. | Cucurbitaceae |
| patalagarudi | Cocculus hirsutus (Linn.) Diels. syn. Menispermum hirsutum L. Cocculus villosus Dc. ; Cocculus villosus Dc. | Menispermaceae |
| pitakarpasa | Cochlospermum religiosum (Linn.) Alston. syn. Cochlospermum gossypium Dc. | Bixaceae |
| narikela | Cocos nucifera Linn. | Arecaceae |
| kaphika -kaphi | Coffea arabica Linn. | Rubiaceae |
| suranjana -surinjana | Colchicum luteum Baker. | Colchicaceae |
| parnayavani | Coleus amboinicus Lour. syn. Coleus aromaticus Benth. | Lamiaceae |
| guggulu | Commiphora mukul (Hook ex Stocks) Engl. | Burseraceae |
| sankhapuspi | Convolvulus pluricaulis Choiss. syns. Convolvulus prostratus Forsk. syn. Convolvulus microphyllus Sieb ex. Spreng. | Convolvulaceae |
| cancu | Corchorus aestuans Linn. Corchorus acutangulas Lamk. | Malvaceae |
| slesmataka | Cordia dichotoma Forst. f. syn. Cordia obliqua Willd ; Cordia myxa Roxb. non Linn. | Boraginaceae |
| dhanyaka | Coriandrum sativum Linn | Apiaceae |
| kasthalata -kalambaka | Coscinium fenestratum Colebr. | Menispermaceae |
| kemuka -kebuka (kevuka) | Costus speciosus (Koen.) Sm. | Zingiberaceae |
| varuna | Crataeva nurvala Buch -Ham. Crateva nurvala F. Ham. syns. Crateva religiosa var. nun/ala (F. Ham.) Hook. L. & Thoms. | Capparidaceae |
| sudarsana | Crinum latifolium Linn. | Amaryllidaceae |
| kunkuma | Crocus sativus Linn. | Iridaceae |
| sana | Crotalaria juncea Linn. | Fabaceae |
| sanapuspi | Crotalaria verrucosa Linn. | Fabaceae |
| jayapala | Croton tiglium Linn. | Euphorbiaceae |
| krsnasariva | Cryptolepis buchanani Roem. & Schult. | Asclepiadaceae |
| trapusa | Cucumis sativus Linn. | Cucurbitaceae |
| ervaru -karkati | Cucumis melo var utilissimus Duthie & Fuller. Cucumis utilissimus Roxb. | Cucurbitaceae |
| gudayogaphala | Cucurbita maxima Duchesne. | Cucurbitaceae |
| jiraka | Cuminum cyminum Linn. | Apiaceae |
| talamuli | Curculigo orchioides Gaertn. | Amaryllidaceae |
| amragandhiharidra -aranyaharidra | Curcuma amada Roxb. | Zingiberaceae |
| aranyaharidra | Curcuma aromatica Salish. | Zingiberaceae |
| haridra | Curcuma longa Linn. | Zingiberaceae |
| karcura | Curcuma zedoaria Rosf. | Zingiberaceae |
| amaravalli | Cuscuta reflexa Roxb. | Convolvulaceae |
| rohisa | Cymbopogon martinii (Roxb.) Wats. syn. Andropogon martinii Roxb. | Poaceae |
| durva | Cynodon dactylon (Linn.) Pers. | Poaceae |
| mustaka | Cyperus rotundus Linn., syns. Cyperus L. ssp. retzil kuk., Cyperus retzil kuk., Cyperus tuberosus sensu Cl., Cyperus scariosus R. Br. | Cyperaceae |
| raktaniryasa | Daemonorops draco Blume (Daemenorops draco Blume.) syn. Calamus draco willd. | Arecaceae |
| goraksa | Dalbergia lanceolaria Linn. | Fabaceae |
| simsapa -simsipa | Dalbergia sissoo Roxb. | Fabaceae |
| dhattura | Datura metel Linn. Datura stramonium Linn. | Solanaceae |
| garjara | Daucus carota Linn. Daucus carota Linn. var. sativa Dc. | Apiaceae |
| sprkka | Delphinium zalil Aitch & Hemsl. | Ranunculaceae |
| bandaka | Loranthus falcatus Linn Desr. Loranthus longifolius. | Loranthaceae |
| salaparni | Desmodium gangeticum DC. | Fabaceae |
| kusa | Desmostachya bipinnata Stapf syn. Briza bipinnata L, Eragrostis bipinnata L. | Poaceae |
| virataru | Dichrostachys cinerea wight & Arn. syn. Caillica cinerea Macb. | Fabaceae |
| tilapuspi -hrtpatri | Digitalis purpurea Linn. | Scrophulariaceae |
| bhavya | Dillenia indica Linn. | Dilleniaceae |
| varahi | Dioscorea bulbifera Linn. syn. Dioscorea sativa Linn. | Dioscoreaceae |
| tinduka | Diospyros peregrina (Gaertn.) Gurke. syn. Diospyros embryopteris Pers. | Ebenaceae |
| asvakarna -garjana | Dipterocarpus alatus Roxb. Dipterocarpus incanus Roxb. | Dipterocarpaceae |
| usaka | Dorema ammoniacum D. Don. | Apiaceae |
| ustrakantaka | Echinops echinatus Roxb. | Asteraceae |
| bhrngaraja | Eclipta alba Hassk. | Asteraceae |
| kesaraja | Wedelia calendulacea Less. syn. Wedelia chinensis Merril | Asteraceae |
| carmakasa (carmavrksa) | Ehretia laevis Roxb. Ehretia aspera Willd. | Boraginaceae |
| rudraksa | Elaeocarpus ganitrus Roxb. | Elaeocarpaceae |
| eladvaya | Elettaria cardamomum Matom. | Zingiberaceae |
| madhulika | Eleusine coracana Gaertn. | Poaceae |
| vidanga | Embelia ribes Burm. f. | Myrsinaceae |
| amalaki | Emblica officinalis Gaertn. Syn. Phyllanthus emblica L. | Euphorbiaceae |
| katunahi | Enicostemma hyssopifolium | Gentianaceae |
| soma | Ephedra vulgaris Wall., Ephedra gerardiana Wall. | Ephedraceae |
| paribhadra | Erythrina variegata Linn. var. orientalis (Linn.) Merill., syn. Erythrina indica Lam. | Fabaceae |
| trisiraparna -ajaparna | Eupatorium triplinerve Vahl. syn. Eupatorium ayapana Vent. | Asteraceae |
| dugdhika | Euphorbia thymifolia Linn. Euphorbia microphylla Heyne. Euphorbia hirta Linn. Euphorbia pilulifera anct. non Linn. | Euphorbiaceae |
| makhanna | Euryale ferox salisb., syn. Anneslia spinosa Roxb. | Nymphaeaceae |
| dhanvayasa | Fagonia cretica Linn. | Zygophyllaceae |
| kapittha | Feronia limonia (Linn.) Swingle., Limonia elephantianum (Correa) Panigrahi (Limonia elephantum, Limonia elephantinum). syn. Feronia limonia correa, Feronia acidissima L. Feronia elephantum Correa. | Rutaceae |
| hingu | Ferula narthex Boiss. Ferula foetida Regel. Narthex asafoetida Fule. | Apiaceae |
| vata | Ficus benghalensis Linn. (Ficus bengalensis Linn.) | Moraceae |
| phalgu | Ficus carica Linn. | Moraceae |
| udumbara | Ficus glomerata Roxb. syn. Ficus racemosa Linn. | Moraceae |
| kakodumbara | Ficus hispida Linn. f. | Moraceae |
| plaksa | Ficus lacor Buch -Ham. | Moraceae |
| asvattha | Ficus religiosa Linn. | Moraceae |
| vikankata | Flacourtia indica (Burm. f.) Merr. syns. Flacourtia ramontchi L. Herit., Gmelina indica Burm. f., Flacourtia sepiaria Roxb. | Salicaceae |
| misreya | Foeniculum vulgare Mill. | Apiaceae |
| parpata | Fumaria vaillantii Loise. syn. Fumaria indica (Hassk.) Pugsley. , Fumaria vaillantii Loisel. var. indica Hassk. , Fumaria parviflora subsp. vaillantii sensu Hook. f. | Papaveraceae |
| vrksamla | Garcinia indica chois. | Clusiaceae |
| amlavetasa | Garcinia pedunculata Roxb. Garcinia indica Choiss. | Clusiaceae |
| nadihingu | Gardenia gummifera Linn. f. | Rubiaceae |
| trayamana | Gentiana kurroo Royle. | Gentianaceae |
| langali | Gloriosa superba Linn. | Liliaceae |
| madhuyasti | Glycyrrhiza glabra Linn. | Fabaceae |
| gambhari | Gmelina arborea Roxb. | Verbenaceae |
| karpasi | Gossypium herbaceum Linn. | Malvaceae |
| parusaka | Grewia asiatica Linn. syn. Grewia subinaequaelis Dc., Grewia hainesiana Dc. | Malvaceae |
| nagabala | Grewia hirsuta vahl. | Malvaceae |
| gangeruki | Grewia populifolia Vahl. Grewia tenax Fiori. | Malvaceae |
| mesasrngi | Gymnema sylvestre R. Br. | Asclepiadaceae |
| sati | Hedychium spicatum Buch -Ham. | Zingiberaceae |
| avartani | Helicteres isora Linn. | Sterculiaceae |
| sariva | Hemidesmus indicus R. Br. | Asclepiadaceae |
| latakasturika | Abelmoschus moschatus Medic. syn. Hibiscus abelmoschus Linn. | Malvaceae |
| bhenda | Hibiscus esculentus Linn. | Malvaceae |
| japa | Hibiscus rosa-sinensis Linn. | Malvaceae |
| kutaja | Holarrhena antidysenterica (Linn.) Wall ex G. Don. | Apocynaceae |
| cirabilva | Holoptelea integrifolia Planch | Ulmaceae |
| yava | Hordeum vulgare Linn. | Poaceae |
| tuvaraka | Hydnocarpus laurifolia (Dennst.) Sleumer. syn. Hydnocarpus wightiana Blume. | Flacourtiaceae |
| parasika yavani | Hyoscyamus niger Linn. | Solanaceae |
| nili | Indigofera tinctoria Linn. | Fabaceae |
| puskaramula | Inula racemosa Hook. f.J. | Asteraceae |
| krsnabija | Ipomoea nil (Linn.) Roth. | Convolvulaceae |
| haimavativaca | Iris versicolor L., Iris × germanica L. | Iridaceae |
| yuthika | Jasminum auriculatum Vahl. | Oleaceae |
| jati | Jasminum officinale Linn. forma grandiflora (Linn.) Kobuski Jasminum grandiflorum Linn. | Oleaceae |
| mallika | Jasminum sambac (Linn.) Ait. | Oleaceae |
| aksota | Juglans regia Linn. | Juglandaceae |
| hapusa | Juniperus communis Linn. | Cupressaceae |
| parnabija | Kalanchoe pinnata (Lamk.) Pers. syn. Bryophyllum pinnatum (Lam.) Kuntz. syn. Bryophyllum calycinum Salilb. | Crassulaceae |
| nispava -simbi | Lablab purpureus (L.) Sweet., Lablab purpurea (L.) Sweet. syn. Dolichos purpurea L., D. purpureus L., Dolichos lablab L. | Fabaceae |
| alabu | Lagenaria siceraria (Molina) Standl. Syn. Cucurbita lagenaria L. | Cucurbitaceae |
| malanga (tutamalanga) | Lallemantia royleana Benth. | Lamiaceae |
| jingini | Lannea grandis Engl. Odina woodier Roxb. | Anacardiaceae |
| ustakhaddusa (ustakhudusa) | Lavandula stoechas Linn. | Lamiaceae |
| madayantika | Lawsonia inermis Linn. | Lythraceae |
| masura | Lens culinaris Medic. syns. Ervum lens L., Lens esculenta Moench. | Fabaceae |
| todari | Lepidium iberis Linn. | Brassicaceae |
| candrasura | Lepidium sativum Linn. | Brassicaceae |
| gandhaprasarini | Leptadenia pyrotechnica (Forsk.) Decne. syn. Paederia foetida Linn, Leptadenia spartium W. & A. V. | Apocynaceae |
| jivanti | Leptadenia reticulata Wight & Arn. | Apocynaceae |
| dronapuspi | Leucas cephalotes (Roth.) Spreng. | Lamiaceae |
| atasi | Linum usitatissimum Linn. | Linaceae |
| medasaka | Litsea chinensis Lamk., syn. Litsea glutinosa (Lour.) C.B. Robins., Litsea sebifera Pors., Tetranthera longifolia Jacq. | Lauraceae |
| samudra narikela | Lodoicea maldivica (Poir.) Pers. syn. Lodoicea seycheliarum Labill. | Arecaceae |
| kosataki krtavedhana | Luffa acutangula (Linn.) Thumb. | Cucurbitaceae |
| kosataki -dhamargava | Luffa cylindrica (Linn.) M.J Roem. Syn. Luffa aegyptiaca Mill. ex Hook.f. | Cucurbitaceae |
| jimutaka | Luffa echinata Roxb. | Cucurbitaceae |
| madhuka | Madhuca indica J.F. Gmelin., Madhuca longifolia ssp. latifolia. (Roxb.) chev syn. Bassia latifolia Roxb. | Sapotaceae |
| marubaka | Majorana hortensis Moench., syn. Origanum majorana Linn. | Lamiaceae |
| kampillaka | Mallotus philippinensis Muell. -Arg. | Euphorbiaceae |
| simbitika -seva | Malus sylvestris Mill. syns. Pyrus malus Linn., Malus pumila Mill., Malus communis DC., Malus sylvestris Hort. non Mill., Malus domestica Borkh. | Rosaceae |
| khubbija-khubbaji | Malva sylvestris Linn. | Malvaceae |
| surapunnaga | Mammea longifolia Planch & Trianna. | Calophyllaceae |
| amra | Mangifera indica Linn ; | Anacardiaceae |
| murva | Marsdenia tenacissima W. & A. | Asclepiadaceae |
| sunisannaka | Marsilea minuta Linn. | Marsileaceae |
| mahanimba | Melia azedarach Linn. | Meliaceae |
| putiha | Mentha spicata Linn. emmend. Nathh. syns. Mentha spicata var. viridis Linn ; Mentha viridis Linn. | Lamiaceae |
| nagakesara | Mesua ferrea Linn., syn. Mesua coromandeliana wight., Mesua pedunculata wight, Mesua speciosa chois. | Calophyllaceae |
| campaka | Michelia champaca Linn. | Magnoliaceae |
| lajjalu | Mimosa pudica Linn. | Fabaceae |
| bakula | Mimusops elengi Linn. | Sapotaceae |
| rajadana | Mimusops hexandra Roxb. syn. Manilkara hexandra (Roxb.) Desv. | Sapotaceae |
| karavellaka | Momordica charantia Linn. | Cucurbitaceae |
| karkotaka | Momordica dioica Roxb. ex willd. syn. Momordica balsamina (wall.) W. & A. | Cucurbitaceae |
| sigru | Moringa oleifera Lam. | Moringaceae |
| tuta | Morus alba Linn. | Moraceae |
| kapikacchu | Mucuna prurita Hook. | Fabaceae |
| kadali | Musa paradisiaca Linn. syn. Musa sapientum Linn. | Musaceae |
| katphala | Myrica esculenta Buch -Ham. | Myricaceae |
| jatiphala | Myristica fragrans Houtt. | Myristicaceae |
| jatamamsi | Nardostachys jatamamsi Dc. | Valerianaceae |
| kamala | Nelumbo nucifera Gaertn. syns. Nymphaea nelumbo L., Nelumbium speciosum Willd. | Nymphaeaceae |
| karavira | Nerium indicum Mill. | Apocynaceae |
| upakuncika | Nigella sativa Linn. | Ranunculaceae |
| Parijata | Nyctanthes arbor-tristis L. | Oleaceae |
| tulasi | Ocimum sanctum Linn. | Lamiaceae |
| gojihva | Onosma bracteatum Wall. | Boraginaceae |
| trivrt | Operculina turpethum (Linn.) Silva Manso. | Convolvulaceae |
| munjataka | Dactylorhiza incarnata (L.) Soó syn. Orchis latifolia Linn. | Orchidaceae |
| syonaka | Oroxylum indicum Vent. | Bignoniaceae |
| sali | Oryza sativa Linn. | Poaceae |
| cangeri | Oxalis corniculata Linn. | Oxalidaceae |
| utasalapa -candrayana | Paeonia emodi wall. | Paeoniaceae |
| ketaki | Pandanus tectorius Soland ex Parkinson., Pandanus fascicularis Lamk. syn. Pandanus odoratissimus Linn. f. | Pandanaceae |
| ahiphena | Papaver somniferum Linn. | Papaveraceae |
| saileya | Parmelia perlata Ach. | Parmeliaceae |
| balaka -hrivera | Pavonia odorata Willd. | Malvaceae |
| masa | Vigna mungo (L.) Heppe syn. Phaseolus mungo L. | Fabaceae |
| Kharjura | Phoenix sylvestris (L.) Roxb. (Type) syn. Elate sylvestris L. Phoenix dactylifera Linn. | Arecaceae |
| jalapippali | Lippia nodiflora Mich | Verbenaceae |
| bhumyamalaki | Phyllanthus niruri Linn. Phyllanthus urinaria Linn. | Euphorbiaceae |
| katuka | Picrorhiza kurroa Royle ex Benth. | Scrophulariaceae |
| sarala | Pinus roxburghii Sargent. | Pinaceae |
| tambula | Piper betle Linn. | Piperaceae |
| cavya | Piper Chaba Hunter. | Piperaceae |
| kankola | Piper cubeba Linn. | Piperaceae |
| pippali | Piper longum L. | Piperaceae |
| marica | Piper nigrum Linn. | Piperaceae |
| karkatasrngi | Pistacia integerrima Stewart ex Brandis. | Anacardiaceae |
| mastaki -(rumimastagi) | Pistacia lentiscus Linn. | Anacardiaceae |
| jalakumbhi -hathah | Pistia stratiotes Linn. | Araceae |
| isadgola | Plantago ovata Forssk.; Plantago major Linn. | Plantaginaceae |
| rasna | Pluchea lanceolata C. B. Clarke. | Asteraceae |
| citraka | Plumbago zeylanica Linn. | Plumbaginaceae |
| kasthadaru -asapallava | Polyalthia longifolia Thw. syn. Uvaria longifolia Sonner. | Annonaceae |
| karanja | Pongamia pinnata (L.) Pierre. syns. Derris indica (Lam.) Benn. Cytisus Pinnatus L. Pongamia glabra Vent. | Fabaceae |
| lonika | Portulaca oleracea Linn: Portulaca quadrifida Linn. | Portulacaceae |
| sami | Prosopis cineraria Druce. | Fabaceae |
| vatada -vatama | Prunus dulcis (Mill.) D.A.Webb syn. Prunus amygdalus Batsch syn. Prunus communis Fritsch., Amydgalus communis Linn. | Rosaceae |
| urumana | Prunus armeniaca Linn. syn. Prunus vulgaris Lam. | Rosaceae |
| padmaka | Prunus cerasoides D. Don. syn. Prunus puddum Roxb. ex Brandis non Miq. | Rosaceae |
| aruka | Prunus persica Stokes Amygdalus persica Linn. | Rosaceae |
| peruka | Psidium guajava Linn. | Myrtaceae |
| bakuci | Psoralea corylifolia Linn. | Fabaceae |
| bijaka | Pterocarpus marsupium Roxb. | Fabaceae |
| raktacandana | Pterocarpus santalinus Linn. | Fabaceae |
| mucakunda | Pterospermum acerifolium willd., syn. Pentapetes acerifolia L. | Malvaceae |
| vidari | Pueraria tuberosa Dc. | Fabaceae |
| dadima | Punica granatum Linn. | Punicaceae |
| putrajivaka | Putranjiva roxburghii Wall. | Putranjivaceae |
| tanka | Pyrus communis Linn. | Poaceae |
| mayaphala | Quercus infectoria Oliv. | Fagaceae |
| kandira | Ranunculus sceleratus Linn. | Ranunculaceae |
| mulaka | Raphanus sativus Linn. | Brassicaceae |
| sarpagandha | Rauwolfia serpentina Benth ex Kurz. (Rauvolfia serpentina Benth ex Kurz.) | Apocynaceae |
| tintidika | Rhus parviflora Roxb. | Anacardiaceae |
| eranda | Ricinus communis Linn. | Euphorbiaceae |
| taruni | Rosa centifolia Linn. | Rosaceae |
| manjistha | Rubia cordifolia Linn. | Rubiaceae |
| cukra | Rumex Vesicarius Linn. | Polygonaceae |
| satapa -sidava | Ruta graveolens Linn. syn. Ruta graveolens L. var. angustifolia Hook. f. | Rutaceae |
| sara | Saccharum munja Roxb. syns. Erianthus munja. Saccharum bengalense Retz., Erianthus sara Rumke, Erianthus ciliaris Jesw., Saccharum sara Roxb., Saccharum ciliare Anders. Saccharum arundinaceum Hook. f. | Poaceae |
| iksu | Saccharum officinarum L. | Poaceae |
| kasa | Saccharum spontaneum Linn. | Poaceae |
| saptacakra | Salacia chinensis Linn. syns. Salacia latifolia Wall ex. M. Laws., Salacia prinoides Dc.. | Celastraceae |
| vetasa | Salix caprea Linn. | Salicaceae |
| jalavetasa | Salix tetrasperma Roxb. | Salicaceae |
| samudrasosa | Salvia plebeia R. Br. | Lamiaceae |
| nagadamana | Sansevieria roxburghiana Schult. f. | Asparagaceae |
| candana | Santalum album Linn. | Santalaceae |
| aristaka | Sapindus trifoliatus Linn | Sapindaceae |
| asoka | Saraca indica Linn. | Fabaceae |
| kustha | Saussurea costus (Fale) Lipsch. syn. Saussurea lappa C. B. Clarke. | Asteraceae |
| kosamra | Schleichera oleosa (Lour.) Oken. syn. Pistacia oleosa Lour., Schleichera trijuga willd. | Sapindaceae |
| moksaka | Schrebera swietenioides Roxb. | Oleaceae |
| kaseruka | Scirpus grossus Linn. syn. Scirpus kysoor Roxb. | Cyperaceae |
| bhallataka | Semecarpus anacardium Linn. | Anacardiaceae |
| tila | Sesamum indicum Linn. | Pedaliaceae |
| agastya | Sesbania grandiflora Retz. | Fabaceae |
| jayanti | Sesbania aegyptiaca Poir. Sesbania sesban (Linn.) Miere. | Fabaceae |
| kanguka | Setaria italica Beauv. | Poaceae |
| sala | Shorea robusta Gacrtn. | Dipterocarpaceae |
| prasarini -rajabala | Sida cordata (Burm. f.) Borss. syn. Sida veronicufolia Linn., Sida veronicaefolia Lam., Sida humilis var. veronicaefolia (Lam.) Mast., Melochia cordata Burm. f., Sida humilis Cav. | Malvaceae |
| bala | Sida cordifolia Linn. | Malvaceae |
| mahabala | Sida rhombifolia (Linn.) Mast. syns. Sida rhomboidea Roxb. ex. Fleming., Sida rhombifolia var. rhomboidea (Roxb. ex Fleming) Mast. | Malvaceae |
| kala -khakasi (khubakalan) | Sisymbrium irio Linn. | Brassicaceae |
| dvipantaravaca | Smilax glabra Roxb. Smilax china Linn. | Smilacaceae |
| brhati | Solanum indicum L. | Solanaceae |
| vrntaka | Solanum melongena Linn. | Solanaceae |
| kakamaci | Solanum nigrum Linn. | Solanaceae |
| kantakari | Solanum surattense Burm. f. syn. Solanum xanthocarpum Sebr. & wende. | Solanaceae |
| mamsarohini | Soymida febrifuga (Roxb.) A. Juss. syn. Swietenia febrifuga Roxb. | Meliaceae |
| mundi | Sphaeranthus senegalensis Dc. , syns. Sphaeranthus indicus Linn. ; Sphaeranthus indicus auct. non L. | Asteraceae |
| palakya | Spinacia oleracea Linn. | Amaranthaceae |
| patala | Stereospermum suaveolens Dc. | Bignoniaceae |
| sakhotaka | Streblus asper Lour. | Moraceae |
| kupilu | Strychnos nux vomica Linn. | Fabaceae |
| kataka | Strychnos potatorum Linn. | Loganiaceae |
| lohavana (loban) | Styrax benzoin Dryand. | Styracaceae |
| Kiratatikta | Swertia chirayita (Roxb.) Buch.-Ham. ex C.B.Clarke syn. Swertia chirata Buch.-Ham. | Gentianaceae |
| lodhra | Symplocos racemosa Roxb. | Symplocaceae |
| lavanga | Syzygium aromaticum (Linn.) Merrill & Perry syns. Eugenia caryophyllus (Spr.) Bull & Harr., Eugenia aromaticus (L.) Baile. | Myrtaceae |
| jambu | Syzygium cumini (Linn.) Skeels. Eugenia jambolana Lam. | Myrtaceae |
| jhandu -sahasrasuma | Tagetes erecta Linn. | Asteraceae |
| amlika | Tamarindus indica Linn | Fabaceae |
| dugdhapheni | Taraxacum officinale Weber. | Asteraceae |
| rohitaka | Tecoma undulata G. Don. | Bignoniaceae |
| saka | Tectona grandis Linn. f. | Verbenaceae |
| sarapunkha | Tephrosia purpurea Pers. syn. Tephrosia hamiltonii Drumm. | Fabaceae |
| masaparni | Teramnus labialis Spreng | Fabaceae |
| arjuna | Terminalia arjuna W. & A. | Combretaceae |
| haritaki | Terminalia chebula Retz. | Combretaceae |
| asana | Terminalia tomentosa W.&A. | Combretaceae |
| bibhitaka | Terminalia bellirica Roxb. Terminalia belerica Roxb. | Combretaceae |
| pitamula -pitaranga | Thalictrum foliolosum Dc. | Ranunculaceae |
| parisa | Thespesia populnea Soland ex. Correa. syn. Hibiscus populneus L. | Malvaceae |
| krsna vetra | Tiliacora acuminata (Lim.) Hook. & Thoms. syn. Tiliacora racemosa Colebr., Menispermum Acuminatum Lam. | Menispermaceae |
| guduci | Tinospora cordifolia (Wild) Miers. | Menispermaceae |
| tuni -tunnaka | Toona ciliata Roem. syn. Cedrela toona Roxb. ex Rottl. | Meliaceae |
| yavani | Trachyspermum ammi (Linn.) Sprague. | Apiaceae |
| srngataka | Trapa natans Linn. var. bispinosa (Roxb.) Makino. syns. Trapa bispinosa Roxb., Trapa quadrispinosa Wall. | Trapaceae |
| goksura | Tribulus terrestris L. | Zygophyllaceae |
| natapuspika-adhahpuspi | Trichodesma indicum R. Br. | Boraginaceae |
| cicinda | Trichosanthes anguina Lam. | Cucurbitaceae |
| patola | Trichosanthes dioica Roxb. | Cucurbitaceae |
| methika | Trigonella foenum-graecum Linn. | Fabaceae |
| godhuma | Triticum aestivum Linn. | Poaceae |
| arkaparni | Tylophora asthmatica W. & A. Tylophora indica Burm. L. Miers. | Asclepiadaceae |
| prsniparni | Uraria picta Desv. syn. Hedysarum pictum Jacq. | Fabaceae |
| kolakanda -vanapalandu | Urginea indica Kunth. | Asparagaceae |
| tagara | Valeriana jatamansi Jones. syn. Valeriana wallichii Dc. | Valerianaceae |
| sarja | Vateria indica Linn. | Dipterocarpaceae |
| sahadevi | Vernonia cinerea Less. | Asteraceae |
| usira | Vetiveria zizanioides (Linn.) Nash. | Poaceae |
| mudgaparni | Vigna trilobata (L.) Verdicourt., Phaseolus trilobus Ait ; Dolichos trilobata L. ; Phaseolus trilobatus auct. non (L.) Ait. ; Phaseolus trilobatus (L.) Schreb. ; Phaseolus trilobus sensu Baker. | Fabaceae |
| kulattha | Dolichos biflorus Linn. syn. Macrotyloma uniflorum (Lamk.) Verde. | Fabaceae |
| sadapuspi | Lochnera rosea (Linn.) Reichhb. syns. Vinca rosea Linn., Catharanthus roseus G. Don. | Apocynaceae |
| vanapsika | Viola odorata Linn. | Violaceae |
| nirgundi | Vitex negundo Linn. | Verbenaceae |
| draksa | Vitis vinifera Linn. | Vitaceae |
| asvagandha | Withania somnifera (Linn.) Dunal. | Solanaceae |
| dhataki | Woodfordia fruticosa Kurz. | Lythraceae |
| madanaphala | Randia dumetorum (Retz.) Poir, Catunaregan spinosa (Thunb) Trivengadum Xeromphis spinosa (Thunb.) Kesv syn. Randia spinosa Poir. | Rubiaceae |
| tumburu | Zanthoxylum armatum Dc. syn. Zanthaxylum alatum Roxb. | Rutaceae |
| ardraka -sunthi | Zingiber officinale Rose: | Zingiberaceae |
| badara | Ziziphus jujuba lam. | Rhamnaceae |
| sauvira -sauvirabadara | Ziziphus sativa Gaertn. syn. Ziziphus vulgaris Linn. | Rhamnaceae |

# References

1. Naik SR, Ganesh RK (2013) Development and Discovery Avenues in Bioactive Natural Products for Glycemic Novel. In: Atta-ur-Rahman F (ed) Ther. Stud. Nat. Prod. Chem. Vol. 39. p 504

2. Wu X, Gu L, Prior RL, et al (2004) Characterization of anthocyanins and proanthocyanidins in some cultivars of Ribes, Aronia, and Sambucus and their antioxidant capacity. J Agric Food Chem 5:7846–7856. doi: 10.1155/S1110724304404045

3. Glabasnia A, Hofmann T (2006) Sensory-Directed Identification of Taste-Active Ellagitannins in American (Quercus alba L.) and European Oak Wood (Quercus robur L.) and Quantitative Analysis in Bourbon Whiskey and Oak-Matured Red Wines. J Agric Food Chem 54:3380–3390. doi: 10.1021/jf052617b

4. Bajec MR, Pickering GJ (2008) Astringency : Mechanisms and Perception Astringency : Mechanisms. Crit Rev Food Sci Nutr 48:858–875. doi: 10.1080/10408390701724223

5. Lea AGH (1990) Bitterness and astringency:the procyanidins of fermented apple ciders. In: Rouseff RL (ed) Bitternes foods beverages. Dev. Food Sci. vol. 25. Elsevier Science Publishers B.V., Amsterdam, pp 123–143

6. Mathew AG, Lakshminarayana S (1969) Polyphenols of immature sapota fruit. Phytochemistry 8:507–509. doi: https://doi.org/10.1016/S0031-9422(00)85457-6

7. Ito S, Joslyn MA (1965) Apple Leucoanthocyanins. J Food Sci 30:44–51. doi: 10.1111/j.1365-2621.1965.tb00261.x

8. Gambuti A, Rinaldi A, Ugliano M, Moio L (2013) Evolution of Phenolic Compounds and Astringency during Aging of Red Wine: E ff ect of Oxygen Exposure before and after Bottling. JAgricFood Chem 61:1618–1627.

9. Bharathi K, Pushpalatha B, Jain CM (2014) Clinical evaluation of herbal compound drugs in the management of leiomyoma induced menorrhagia. Ayushdhara 1:38–42.

10. Joslyn MA, Goldstein JL (1964) Astringency of Fruits and Fruit Products in Relation to Phenolic Content. In: Chichester CO, Mrak EM, Stewart GFBT-A in FR (eds). Academic Press, pp 179–217

11. Silva MS, García-Estévez I, Brandão E, et al (2017) Molecular Interaction Between Salivary Proteins and Food Tannins. J Agric Food Chem 65:6415–6424. doi: 10.1021/acs.jafc.7b01722

12. Drewnowski A, Gomez-Carneros C (2000) Bitter taste, phytonutrients, and the consumer: a review. Am J Clin Nutr 72:1424–35.

13. El Gharras H (2009) Polyphenols: food sources, properties and applications - a review. Int J Food Sci Technol 44:2512–2518. doi: 10.1111/j.1365-2621.2009.02077.x

14. Hufnagel JC, Hofmann T (2008) Orosensory-directed identification of astringent mouthfeel and bitter-tasting compounds in red wine. J Agric Food Chem 56:1376–1386. doi: 10.1021/jf073031n

15. Huang CJ, Zayas JF (1991) Phenolic Acid Contributions to Taste Characteristics of Corn Germ Protein Flour Products. J Food Sci 56:1308–1310. doi: 10.1111/j.1365-2621.1991.tb04759.x

16. Sosulski F, Krygier K, Hogge L (1982) Free, esterified, and insoluble-bound phenolic acids. 3. Composition of phenolic acids in cereal and potato flours. J Agric Food Chem 30:337–340. doi: 10.1021/jf00110a030

17. Kar A (2003) Pharmacognosy and pharmacobiotechnology. New Age International (P) Limited, New Dehli

18. Critchley HD, Rolls ET (1996) Responses of primate taste cortex neurons to the astringent tastant tannic acid. Chem Senses 21:135–145.

19. Scharbert S, Holzmann N, Hofmann T (2004) Identification of the Astringent Taste Compounds in Black Tea Infusions by Combining Instrumental Analysis and Human Bioresponse. J Agric Food Chem 52:3498–3508. doi: 10.1021/jf049802u

20. McCamey DA, Thorpe TM, McCarthy JP (1990) Coffe bitterness. In: Rouseff RL (ed) Bitternes foods beverages. Dev. Food Sci. vol. 25. Elsevier Science Publishers B.V., Amsterdam, pp 171–182

21. Zhu M, Li N, Zhao M, et al (2017) Metabolomic profiling delineate taste qualities of tea leaf pubescence. Food Res Int 94:36–44. doi: 10.1016/j.foodres.2017.01.026

22. Roland WSU, van Buren L, Gruppen H, et al (2013) Bitter Taste Receptor Activation by Flavonoids and Isoflavonoids: Modeled Structural Requirements for Activation of hTAS2R14 and hTAS2R39. J Agric Food Chem 61:10454–10466. doi: 10.1021/jf403387p

23. Wiener A, Shudler M, Levit A, Niv MY (2012) BitterDB: a database of bitter compounds. Nucleic Acids Res 40:D413–D419. doi: 10.1093/nar/gkr755

24. Guichard E, Salles C, Morzel M, Le Bon A-M (2017) Flavour From Food to Perception. John Wiley & Sons, Ltd, Chichester, West SussexHoboken, NJ

25. Belitz H, Wieser H (1985) Bitter compounds: Occurrence and structure‐activity relationships. Food Rev Int 1:271–354. doi: 10.1080/87559128509540773

26. Wiener A, Shudler M, Levit A, Masha YN (2012) BitterDB: a database of bitter compounds. Nucleic Acids Res 2012, 40(Database issue):D413-419.

27. Rouseff RL (1990) Bitternes in food products: an overview. In: Rouseff RL (ed) Bitternes foods beverages. Dev. Food Sci. vol. 25. Elsevier Science Publishers B.V., Amsterdam, pp 1–14

28. Plet B, Delcambre A, Chaignepain S, Schmitter J-M (2015) Affinity ranking of peptide–polyphenol non-covalent assemblies by mass spectrometry approaches. Tetrahedron 71:3007–3011. doi: https://doi.org/10.1016/j.tet.2015.02.015

29. Okubo K, Iijima M, Kobayashi Y, et al (1992) Components Responsible for the Undesirable Taste of Soybean Seeds. Biosci Biotechnol Biochem 56:99–103. doi: 10.1271/bbb.56.99

30. Puech J-L, Feuillat F, Mosedale JR (1999) The Tannins of Oak Heartwood: Structure, Properties, and Their Influence on Wine Flavor. Am J Enol Vitic 50:469 LP-478.

31. Drewnowski A, Gomez-carneros C (2000) Bitter taste , phytonutrients , and the consumer : a review 1 – 3. 1424–1435.

32. Narukawa M, Noga C, Ueno Y, et al (2011) Evaluation of the bitterness of green tea catechins by a cell-based assay with the human bitter taste receptor hTAS2R39. Biochem Biophys Res Commun 405:620–625. doi: https://doi.org/10.1016/j.bbrc.2011.01.079

33. Brossaud F, Cheynier V, Noble AC (2001) Bitterness and astringency of grape and wine polyphenols. Aust J grape wine Res 7:33–39.

34. Canon F, Giuliani A, Pate F, Sarni-Manchado P (2010) Ability of a salivary intrinsically unstructured protein to bind different tannin targets revealed by mass spectrometry. Anal Bioanal Chem 398:815–822. doi: 10.1007/s00216-010-3997-9

35. Versari A, du Toit W, Parpinello GP (2013) Oenological tannins: a review. Aust J Grape Wine Res 19:1–10. doi: 10.1111/ajgw.12002

36. Yaminishi T (1990) Bitter compounds in tea. In: Rouseff RL (ed) Bitternes foods beverages. Dev. Food Sci. vol. 25. Elsevier Science Publishers B.V., Amsterdam, pp 159–167

37. Bajec MR, Pickering GJ (2008) Astringency : Mechanisms and Perception. Crit Rev Food Sci Nutr 48:858–875. doi: 10.1080/10408390701724223

38. Soares S, Ferrer-Galego R, Brandao E, et al (2016) Contribution of Human Oral Cells to Astringency by Binding Salivary Protein/Tannin Complexes. J Agric Food Chem. doi: 10.1021/acs.jafc.6b02659

39. Schiffman SS, Suggs MS, Sostman AL, Simon SA (1992) Chorda tympani and lingual nerve responses to astringent compounds in rodents. Physiol Behav 51:55–63.

40. Wollmann N, Hufnagel J-C, Hoffman T (2013) Decoding the taste of wine using a sensomic approach. In: Ferreira V, López R (eds) Flavour Sci. Proc. from XIII Weurman Flavour Res. Symp. Elsevier Science, pp 525–532

41. Chang SS, Huang A-S, Ho C-T (1990) Isolation and identification of bitter compounds in defatted soybean flour. In: Rouseff RL (ed) Bitternes foods beverages. Dev. Food Sci. vol. 25. Elsevier Science Publishers B.V., Amsterdam, pp 267–274

42. Roland WSU, Vincken J-P, Gouka RJ, et al (2011) Soy Isoflavones and Other Isoflavonoids Activate the Human Bitter Taste Receptors hTAS2R14 and hTAS2R39. J Agric Food Chem 59:11764–11771. doi: 10.1021/jf202816u

43. Sharma KM, Mukesh B (2013) Significance of Plant Bitters in the Field of Pharmacognosy. Asian J Pharmacutical Technol Innov 1:3–12.

44. Soares S, Kohl S, Thalmann S, et al (2013) Different Phenolic Compounds Activate Distinct Human Bitter Taste Receptors. J Agric Food Chem 61:1525–1533. doi: 10.1021/jf304198k

45. Behrens M, Gu M, Fan S, et al (2017) Bitter substances from plants used in traditional Chinese medicine exert biased activation of human bitter taste receptors. Chem Biol Drug Des n/a-n/a. doi: 10.1111/cbdd.13089

46. Matsuo T, Itoo S (1981) Comparative Studies of Condensed Tannins from Several Young Fruits. J Japan Soc Hort Sci 50:262–269.

47. Hofman T, Glabasnia A, Schwarz B, et al (2006) Protein binding & astringent taset of a polymeric procyanidin, 1,2,3,6-penta-O-galloyl-B-D-glycopyranose castalagin, and grandinin. J Agric Food Chem 54:9503–9509.

48. Sohn CE (1894) Dictionary of the active principles of plants : alkaloids, bitter principles, glucosides, their sources, nature, and chemical characteristics, with tabular summary, classification of reactions, and full botanical and general indexes. Baillière, Tindall and Cox, London

49. Koch IS (2011) Development of a sensory lexicon and sensory wheel for rooibos (Aspalathus linearis) and the role of its phenolic composition on taste and mouthfeel (PhD Thesis).

50. Kraehenbuehl K, Page-Zoerkler N, Mauroux O, et al (2017) Selective enzymatic hydrolysis of chlorogenic acid lactones in a model system and in a coffee extract. Application to reduction of coffee bitterness. Food Chem 218:9–14. doi: 10.1016/j.foodchem.2016.09.055

51. Peleg H, Noble AC (1995) Perceptual Properties of Benzoic Acid Derivatives. Chem Senses 20:393–400.

52. Dadic M, Belleau G (1973) Polyphenols and beer flavor. In: Proc. Am. Soc. Brew. Chem. pp 107–114

53. Backes M, Obst K, Bojahr J, et al (2015) Rubemamine and Rubescenamine, Two Naturally Occurring N-Cinnamoyl Phenethylamines with Umami-Taste-Modulating Properties. J Agric Food Chem 63:8694–8704. doi: 10.1021/acs.jafc.5b04402

54. Frerot E, Neirynck N, Cayeux I, et al (2015) New Umami Amides: Structure–Taste Relationship Studies of Cinnamic Acid Derived Amides and the Natural Occurrence of an Intense Umami Amide in Zanthoxylum piperitum. J Agric Food Chem 63:7161–7168. doi: 10.1021/acs.jafc.5b02359

55. Shallenberger RS (1993) Taste Chemistry, 1st ed. Springer Science+Business Media Dordrecht, Northampton

56. Kim S, Thiessen PA, Bolton EE, et al (2016) PubChem Substance and Compound databases. Nucleic Acids Res 44:D1202–D1213. doi: 10.1093/nar/gkv951

57. Mander LN, Liu H, Townsend CA, Ebizuka Y (2010) Comprehensive natural products II : chemistry and biology, Volume I, Natural Products Structural Diversity. Secondary Metabolites. Organization and Biosynthesis, First.

58. Khan IA, Abourashed EA (2010) Leung’s Encyclopedia of Common Natural Ingredients Used in Food, Drugs, and Cosmetics, 3rd ed. John Wiley & Sons, Inc., Hoboken, New Jersey

59. Beigh YA, Ganai AM (2017) Potential of Wormwood (Artemisia absinthium Linn.) herb for use as additive in livestock feeding: A review. Pharma Innov 6:176.

60. Sharma V, Singh I, Chaudhary P (2014) Acorus calamus (The Healing Plant): a review on its medicinal potential, micropropagation and conservation. Nat Prod Res 28:1454–1466. doi: 10.1080/14786419.2014.915827

61. Saroya AS (2011) Herbalism , Phytochemistry and Ethnopharmacology. Science Publishers, CRC Press Taylor & Francis Group, Enfield USA

62. Stahl E, Keller K (1983) Extraktion labiler Naturstoffe mit überkritischen Gasen. Planta Med 47:75–78.

63. Chang CWJ, Flament I, Matson JA, et al (1979) Fortschritte der Chemie Organischer Naturstoffe (Progress in the chemistry of organic natural products) vol.36. Springer Science & Business Media, Vienna

64. Aniszewski T (2015) Chapter 1 - Definition, typology, and occurrence of alkaloids. In: Alkaloids, 2nd ed. Elsevier, Boston, pp 1–97

65. Maga JA (1990) Compound structure versus bitter taste. In: Rouseff RL (ed) Bitternes foods beverages. Dev. Food Sci. vol. 25. Elsevier Science Publishers B.V., pp 35–48

66. Chialva F, Dada G (1990) Bitterness in alcoholic beverages. In: Rouseff RL (ed) Bitternes foods beverages. Dev. Food Sci. vol. 25. Elsevier Science Publishers B.V., pp 103–122

67. Joseph B, Raj Sj (2010) Pharmacognostic and phytochemical properties of Aloe vera Linn- an overview. Int J Pharm Sci Rev Res 4:17.

68. Meyerhof W, Batram C, Kuhn C, et al (2010) The molecular receptive ranges of human TAS2R bitter taste receptors. Chem Senses 35:157–170. doi: 10.1093/chemse/bjp092

69. Niiho Y, Yamazaki T, Nakajima Y, et al (2006) Gastroprotective effects of bitter principles isolated from Gentian root and Swertia herb on experimentally-induced gastric lesions in rats. J Nat Med 60:82–88. doi: 10.1007/s11418-005-0014-2

70. Behrens M, Brockhoff A, Batram C, et al (2009) The human bitter taste receptor hTAS2R50 is activated by the two natural bitter terpenoids andrographolide and amarogentin. J Agric Food Chem 57:9860–9866. doi: 10.1021/jf9014334

71. Khare CP (2004) Indian Herbal Remedies: Rational Western Therapy, Ayurvedic and Other Traditional Usage, Botany. doi: 10.1007/978-3-642-18659-2_1

72. Brockhoff A, Behrens M, Roudnitzky N, et al (2011) Receptor Agonism and Antagonism of Dietary Bitter Compounds. J Neurosci 31:14775 LP-14782.

73. Sheshagiri S, Patel KS, Rajagopala S (2015) Randomized placebo-controlled clinical study on enhancement of Medha (intelligence quotient) in school going children with Yahstimadhu granules. Ayu 36:56–62. doi: 10.4103/0974-8520.169011

74. Zhang C, Li L, Xiao Y (2010) Approaches to Revealing the Relation between the Processing and Property of Chinese Medicines. World Sci Technol 12:876–881. doi: 10.1016/S1876-3553(11)60031-8

75. Anonymous (2007) Final report on the safety assessment of AloeAndongensis Extract, Aloe Andongensis Leaf Juice,aloe Arborescens Leaf Extract, Aloe Arborescens Leaf Juice, Aloe Arborescens Leaf Protoplasts, Aloe Barbadensis Flower Extract, Aloe Barbadensis Leaf, Aloe Barba. Int J Toxicol 26 Suppl 2:1–50. doi: 10.1080/10915810701351186

76. Rathore MS, Chikara J, Shekhawat NS (2011) Plantlet Regeneration from Callus Cultures of Selected Genotype of Aloe vera L.—An Ancient Plant for Modern Herbal Industries. Appl Biochem Biotechnol 163:860–868. doi: 10.1007/s12010-010-9090-1

77. Steck W, Wetter LR (1974) Apterin, an unusual glucoside of Zizia aptera. Phytochemistry 13:1925–1927. doi: 10.1016/0031-9422(74)85117-4

78. Bufe B, Hofmann T, Krautwurst D, et al (2002) The human TAS2R16 receptor mediates bitter taste in response to β-glucopyranosides. Nat Genet 32:397–401. doi: 10.1038/ng1014

79. Wszelaki N, Agnieszka K, Anna K (2010) Screening of traditional European herbal medicines for acetylcholinesterase and butyrylcholinesterase inhibitory activity. Acta Pharm 60:119. doi: 10.2478/v10007-010-0006-y

80. Horn G, Kupfer A, Rademacher A, et al (2015) Cnicus benedictus as a potential low input oil crop. Eur J Lipid Sci Technol 117:561–566.

81. Rojas C, Todeschini R, Ballabio D, et al (2017) A QSTR-Based Expert System to Predict Sweetness of Molecules . Front Chem 5:53.

82. Suzuki T, Morita M, Kobayashi Y, Kamimura A (2016) Oral L-citrulline supplementation enhances cycling time trial performance in healthy trained men: Double-blind randomized placebo-controlled 2-way crossover study. J Int Soc Sports Nutr 13:6. doi: 10.1186/s12970-016-0117-z

83. Kubickova J, Grosch W (1998) Evaluation of Flavour Compounds of Camembert Cheese. Int Dairy J 8:11–16. doi: 10.1016/S0958-6946(98)00015-6

84. Haefeli R, Glaser D (1990) Taste responses and thresholds obtained with the primary amino acids in humans. Leb und Technol 23:523–527.

85. Fattorusso E, Taglialatela-Scafati O, Wiley InterScience (Online service) (2008) Modern alkaloids : structure, isolation, synthesis and biology. Wiley-VCH

86. Schiffman SS, Sennewald K, Gagnon J (1981) Comparison of taste qualities and thresholds of D- and L-amino acids. Physiol Behav 27:51–59. doi: https://doi.org/10.1016/0031-9384(81)90298-5

87. Ukani MD, Mehta NK, Nanavati DD (1996) Aconitum heterophyllum (ativisha) in ayurveda. Anc Sci Life 16:166–171.

88. Li X, Xu H, Li Q, et al (2008) Identification of bitter ligands that specifically activate human t2r receptors and related assays for identifying human bitter taste modulators.

89. Behrens M, Brockhoff A, Kuhn C, et al (2004) The human taste receptor hTAS2R14 responds to a variety of different bitter compounds. Biochem Biophys Res Commun 319:479–485. doi: 10.1016/j.bbrc.2004.05.019

90. Wagner H., Wolff PM (1976) New Natural Products and Plant Drugs with Pharmacological, Biological or Therapeutical Activity: Proceedings of the First International Congress on Medicinal Plant Research, Section A,.

91. Wagner H, Bladt S (1996) Bitter Drugs. In: Plant Drug Anal. Springer Berlin Heidelberg, Berlin, Heidelberg, pp 73–97

92. Baas WJ (1985) Naturally occurring seco-ring-a-triterpenoids and their possible biological significance. Phytochemistry 24:1875–1889.

93. Morgan ED, Wilson ID (1999) Insect Hormones and Insect Chemical Ecology. In: Otto M-C, Sir Derek B, Koji N (eds) Compr. Nat. Prod. Chem. pp 263–375

94. Dreyer DL (1966) Citrus bitter principles-v. Botanical distribution and chemotaxonomy in the Rutaceae. Phytochemistry 5:367–378. doi: 10.1016/S0040-4020(01)82204-3

95. Sharma K, Kansal A, Chopra S (2013) Premenstrual syndrome, body fat and bitter taste receptor gene TAS2R38 among adult Kullu females of Himachal Pradesh, India. Anthropol Anz 70:203–219.

96. Saroli A (1984) Structure-activity relationship of a bitter compound: Denatonium chloride. Naturwissenschaften 71:428–429. doi: 10.1007/BF00365895

97. Saroli A (1986) Structure-activity relationship of bitter compounds related to denatonium chloride and dipeptide methyl esters. Zeitschrift für Leb und Forsch 182:118–120. doi: 10.1007/BF01454242

98. Mukerji B, Ghosh BK, Siddons LB (1943) The Search for an Anti-Malarial Drug in the Indigenous Materia Medica: Part II—Cæsalpinia Bonducella, Fleming . Ind Med Gaz 78:285–288.

99. Manikandaselvi S, Vadivel V, Brindha P (2015) Review Article Caesalpinia bonducella L .: A nutraceutical plant. J Chem Pharm Res 7:137–142.

100. Brockhoff A, Behrens M, Massarotti A, et al (2007) Broad tuning of the human bitter taste receptor hTAS2R46 to various sesquiterpene lactones, clerodane and labdane diterpenoids, strychnine, and denatonium. J Agric Food Chem 55:6236–6243. doi: 10.1021/jf070503p

101. Bassoli A, Borgonovo G, Busnelli G (2007) Alkaloids and the Bitter Taste. In: Fattorusso E, Taglialatela-Scafati O (eds) Mod. Alkaloids Struct. Isol. Synth. Biol. Wiley-VCH Verlag GmbH & Co. KGaA, Weinheim, Germany, pp 53–72

102. Cocker W (1966) Some aspects of the chemistry of diterpene bitter principles. Planta Med 14:78–85. doi: 10.1055/s-0028-1100084

103. Adam SEI (1978) Toxicity of Indigenous Plants and Agricultural Chemicals in Farm Animals. Clin Toxicol 13:269–280. doi: 10.3109/15563657808988237

104. Barger G, Robinson R, Urushibara Y (1937) 141. Synthetical experiments relating to carpaine. Part I. Synthesis of a basic long-chain lactone. J Chem Soc 0:714. doi: 10.1039/jr9370000714

105. Manske RH., Holmes HL (1950) The Alkaloids- Chemistry and Physiology. Academic Press Inc., New York

106. Rahman A-, Choudhary MI (2015) Applications of NMR spectroscopy. Volume 3. Bentham Science Publishers Ltd

107. Merck E (1902) Merck’s index. Eduard Roether, Darmstadt

108. Wittstein M (1857) On a new Cinchona bark and its alkaloid, cinchonidine. Am J Pharm May:115.

109. Burdock GA (2010) Fenaroli’s Handbook of Flavor Ingredients, Sixth. CRC Press, Taylor and Francis Group, Boca Raton London New York

110. Kempler GM (1983) Production of flavour compounds by microorganisms. In: Laskin AI (ed) Adv. Appl. Microbiol. Vol. 29. Academic Press, pp 29–49

111. Barton DHR, Cheung HT, Cross AD, et al (1961) 1003. Diterpenoid bitter principles. Part III. The constitution of clerodin. J Chem Soc 5061–5073. doi: 10.1039/JR9610005061

112. Van Dyke C, Byck R (1982) Cocaine. Sci Am 246:128–141.

113. Hyyatiä P, Sinclair JD (1993) Oral etonitazene and cocaine consumption by AA, ANA and Wistar rats. Psychopharmacology (Berl) 111:409–414. doi: 10.1007/BF02253529

114. Kefford JF (1960) The Chemical Constituents of Citrus Fruits. In: Chichester CO, Mrak EM, Stewart GFBT-A in FR (eds). Academic Press, pp 285–372

115. Cava MP, Soboczenski EJ (1956) Bitter Principles of Plants. I. Columbin: Preliminary Structural Studies1. J Am Chem Soc 78:5317–5322. doi: 10.1021/ja01601a045

116. Chen JC, Chiu MH, Nie RL, et al (2005) Cucurbitacins and cucurbitane glycosides: structures and biological activities. Nat Prod Rep 22:386–399. doi: 10.1039/b418841c

117. Yanfang Z, Xingping L, Zongde Z, et al (2006) Simultaneous determination of andrographolide and dehydroandrographolide in Andrographis paniculata and Chinese medicinal preparations by microemulsion electrokinetic chromatography. J Pharm Biomed Anal 40:157–161.

118. Fenwick GR, Curl CL, Griffiths NM, et al (1990) Bitter principles in food plants. In: Rouseff RL (ed) Bitternes foods beverages. Dev. Food Sci. vol. 25. Elsevier Science Publishers B.V., Amsterdam, pp 205–250

119. Ivie GW, Witzel DA, Rushing DD (1975) Toxicity and milk bittering properties of tenulin, the major sesquiterpene lactone constituent of Heletiium amarum (bitter sneezeweed). J Agric Food Chem 23:845.

120. Holzer K, Zinke A (1953) Über die Bitterstoffe der Zichorie (Cichorium intybus L). Monatshefte für Chemie und verwandte Teile anderer Wissenschaften 84:901–909. doi: 10.1007/BF00899298

121. Hook IL. (1994) Taraxacum officinale Weber Dandelion In Vitro Culture Micropropagation and the Production of Volatile Metabolites. In: Bajaj YPS (ed) Med. Aromat. Plants- VI. Springer Science & Business Media, pp 356–369

122. Kuusi T, Pyysalo H, Autio K (1985) The bitterness properties of dandelion. II. Chemical investigations. Leb Technol 18:347–349.

123. Webster J, Beck W, Ternai B (1984) Toxicity and bitterness in Australian Dioscorea bulbifera L. and Dioscorea hispida Dennst. from Thailand. J Agric Food Chem 32:1087–1090. doi: 10.1021/jf00125a039

124. Wood HC (1872) A Year-book of Therapeutics, Pharmacy and Allied Sciences - Google Books. William Wood Company, New York

125. Brown GL, Dale H (1935) The pharmacology of ergometrine. Proc R Soc London Ser B, Biol Sci 118:446–477.

126. Moraal LG, Goedhart PW (1999) Differences in palatability of Fraxinus excelsior L., for the vole, Microtus arvalis ,and the scale, Pseudochermes fraxini L. In: Lieutier F, Mattson WJ, Wagner MR (eds) Physiol. Genet. Tree-Phytophage Interact. Int. Symp. lnstitut National de la Recherche Agronornique (INRA) International Union of Forestry Research Organizations (IUFRO), Gujan (France), pp 111–121

127. Kreutzmann S, Christensen LP, Edelenbos M (2008) Investigation of bitterness in carrots (Daucus carota L.) based on quantitative chemical and sensory analyses. LWT - Food Sci Technol 41:193–205. doi: 10.1016/j.lwt.2007.02.024

128. Caballero B, Trugo LC, Finglas PM (2003) Encyclopedia of food sciences and nutrition, 2nd ed. Academic Press

129. Youngken HW (1921) A Text Book of Pharmacognosy. P. Blakiston’s Son & Company

130. Baxter H, Harborne J., Moss GP (1999) Phytochemical dictionary : a handbook of bioactive compounds from plants, 2nd ed. CRC Press. Taylor & Francis

131. Olennikov ND, Kashchenko IN, Chirikova KN, et al (2015) Bitter Gentian Teas: Nutritional and Phytochemical Profiles, Polysaccharide Characterisation and Bioactivity. Mol . doi: 10.3390/molecules201119674

132. Kidwai A, Salooja K, Sharma V, et al (1949) Chemical examination of Tinospora cordifolia. J Sci Ind Res 8:115–118.

133. Thattet UM, Dahanukar SA (1989) Immunotherapeutic modification of experimental infections by Indian medicinal plants. Phyther Res 3:43–49.

134. Sinha RK (1992) Herbal Remedies of Street Vendors for Some Urino-Genital Diseases. Anc Sci Life 11:187–192. doi: ASL-11-187 [pii]

135. Geissman TA (1964) New Substances of Plant Origin. Annu Rev Pharmacol 4:305–316. doi: 10.1146/annurev.pa.04.040164.001513

136. Wieczorek MN, Walczak M, Skrzypczak-Zielinska M, Jelen HH (2017) Bitter taste of Brassica vegetables: The role of genetic factors, receptors, isothiocyanates, glucosinolates, and flavor context. Crit Rev Food Sci Nutr 1–11. doi: 10.1080/10408398.2017.1353478

137. Fenwick GR, Griffiths NM, Heaney RK (1983) Bitterness in brussels sprouts (Brassica oleracea L. var. gemmifera): The role of glucosinolates and their breakdown products. J Sci Food Agric 34:73–80. doi: 10.1002/jsfa.2740340111

138. Fugmann F, Lang-Fugmann S, Steglich W (2000) Encyclopedia of Natural Products, 1st ed. Georg Thieme Verlag

139. Ma W-W, Heinstein PF, McLaughlin JL (1989) Additional Toxic, Bitter Saponins from the Seeds of Chenopodium quinoa. J Nat Prod 52:1132–1135. doi: 10.1021/np50065a035

140. Clark EP (1936) Helenalin. I. Helenalin, the Bitter Sternutative Substance Occurring in Helenium Autumnale. J Am Chem Soc 58:1982–1983. doi: 10.1021/ja01301a046

141. Brieskorn CH (1990) Physiological and therapeutical aspects of bitter compounds. In: Rouseff RL (ed) Bitternes foods beverages. Dev. Food Sci. vol. 25. Elsevier Science Publishers B.V., Amsterdam, pp 15–33

142. Fragner K (1888) Ein neues Alkaloïd «Imperialin». Eur J Inorg Chem 21:3284–3287.

143. Hoogewerff S, Meulen H Ter (1899) Contribution to the knowledge of indican. K Ned Akad van Wet Proc Ser B Phys Sci 2:520–525.

144. Yamane H, Konno K, Sabelis M, et al (2010) Chemical defense and toxins of plants. In: Compr. Nat. Prod. II. pp 1033–1084

145. Sung CK, Kang GH, Yoon SS, et al (1996) Glycosidases that convert natural glycosides to bioactive compounds. In: Waller GR, Yamasaki K (eds) Sapon. Used Tradit. Mod. Med. vol 404. Springer Science & Business Media, pp 23–36

146. Dubey S, Maity S, Singh M, et al (2013) Phytochemistry, pharmacology and toxicology of spilanthes acmella: A review. Adv Pharmacol Sci 2013:9 pages. doi: 10.1155/2013/423750

147. Norris DM (1976) How Certain Insects Take the Bitter with the Sweet. Bull Entomol Soc Am 22:27–30. doi: 10.1093/besa/22.1.27

148. Rodgers S, Busch J, Peters H, Christ-Hazelhof E (2005) Building a Tree of Knowledge: Analysis of Bitter Molecules. Chem Senses 30:547–557. doi: 10.1093/chemse/bji048

149. Wesołowska A, Nikiforuk A, Michalska K, et al (2006) Analgesic and sedative activities of lactucin and some lactucin-like guaianolides in mice. J Ethnopharmacol 107:254–258. doi: https://doi.org/10.1016/j.jep.2006.03.003

150. Kubo I, Matsumoto A, Takase I (1985) A multichemical defense mechanism of bitter oliveOlea europaea (oleaceae). J Chem Ecol 11:251–263.

151. Ayensu ES (1981) Medicinal plants of the West Indies. Reference Publications

152. Rehr SS, Feeny PP, Janzen DH (1973) Chemical Defence in Central American Non-Ant-Acacias. J Anim Ecol 42:405–416. doi: 10.2307/3294

153. Boskou D, Camposeo S, Clodoveo ML (2015) 8 - Table Olives as Sources of Bioactive Compounds BT - Olive and Olive Oil Bioactive Constituents. AOCS Press, pp 217–259

154. Shankar R, Rawat MS (2012) Conservation of traditional medicinal practices and pharmaceutically. J Ethnobiol Tradit Med 117:178–188.

155. El-Hawary ZM, Kholief TS (1990) Biochemical studies on hypoglycemic agents (I) effect ofAzadirachta indica leaf extract. Arch Pharm Res 13:108–112. doi: 10.1007/BF02857845

156. Siddiqui S, Faizi S, Mahmood T, Siddiqui BS (1986) Margosinolide and isomargosinolide, two new tetranortriteprenoids from azadirachta indica a, juss (Meliaceae). Tetrahedron 42:4849–4856. doi: https://doi.org/10.1016/S0040-4020(01)82066-4

157. Palter R, Lundin RE (1970) A bitter principle of safflower; matairesinol monoglucoside. Phytochemistry 9:2407–2409. doi: https://doi.org/10.1016/S0031-9422(00)85750-7

158. Palter R, Lundin RE, Haddon WF (1972) A cathartic lignan glycoside isolated from Carthamus tinctorus. Phytochemistry 11:2871–2874.

159. Moore B, Foster H V, Hanley H, et al (1913) The chemical and pharmacological properties of hederin, a sapo-glucoside contained in the leaves of common ivy (Hedera helix). J Pharmacol Exp Ther 4:263–275.

160. Jacobson M (1977) Isolation and Identification of Toxic Agents from Plants. In: Host Plant Resist. to Pests. AMERICAN CHEMICAL SOCIETY, pp 10–153

161. Aubert S, Daunay M.C., Pochard E (1989) Saponosides stéroïdiques de l’aubergine (Solanum melongena L.) I. Intérêt alimentaire, méthodologie d’analyse, localisation dans le fruit. Agron EDP Sci 9:641–651.

162. Prohens-Tomas J, Nuez F (2008) Vegetables II: Fabaceae, Liliaceae, Solanaceae, and Umbelliferae. Springer Science & Business Media

163. Dutta P, Deb NC, Bose PK (1940) A preliminary note on mesuol, the bitter principle of Mesua ferrea. J Indian Chem Soc 17:277–279.

164. Chakraborty DP, Bose P (1960) On the constitution of mesuol the bitter antibiotic principle of Mesua ferea linn. Part 1. Proc Natl Inst Sci India 26:1–11.

165. Sanyal PK (1968) Chemical investigation of some plants of the family verbenaceae (PhD Thesis). doi: http://hdl.handle.net/10603/156829

166. Thakur AK, Chatterjee SS, Kumar V (2014) Andrographolides and traditionally used Andrographis paniculata as potential adaptogens: Implications for therapeutic innovation. Tang [Humanitas Med 4:15.1-15.14. doi: 10.5667/tang.2014.0002

167. Siddiqui S (1942) A note on isolation of three new bitter principles from the neem oil. Curr Sci 11:278–279.

168. Nakanishi K, Goto T, Ito S, et al (1974) Natural products chemistry. Nat Prod Chem. doi: 10.1016/B978-0-12-513901-4.50006-8

169. Haagen-Smit AJ, Nimmo CC (1963) Chemistry of Isoprenoid Compounds. In: Skulachev VP, Semenza G (eds). Elsevier, pp 115–168

170. Tang W, Eisenbrang G (1992) Chinese Drugs of Plant Origin. Chemistry, Pharmacology, and Use in Traditional and Modem Medicine. Springer. doi: 10.1007/978-3-642-73739-8_8

171. Boutaghane N, Kabouche Z, Voutquenne-Nazabadioko L (2016) Triterpene saponins from Fagonia scabra Forssk and other Fagonia species. Biochem Syst Ecol 67:1–6. doi: https://doi.org/10.1016/j.bse.2016.05.017

172. Mikolajczyk-Bator K, Kikut-Ligaj D (2016) Triterpene saponins as bitter components of beetroot. Zywn Nauk Technol Jakosc/Food Sci Technol Qual 104:128–141. doi: 10.15193/zntj/2016/104/107

173. Palit P, Furman BL, Gray AI (1999) Novel weight-reducing activity of Galega officinalis in mice. J Pharm Pharmacol 51:1313–1319.

174. Evans WC (2009) Trease and Evans Pharmacognosy, 16th ed. Elsevier Ltd

175. Kitagawa I, Hino K, Nishimura T, et al (1971) On the Constituents of Picrorhiza kurrooa. (1). The Structure of Picroside I, a Bitter Principle of the Subterranean Part. Chem Pharm Bull (Tokyo) 19:2534–2544. doi: 10.1248/cpb.19.2534

176. Kitagawa I, Hino K, Nishimura T, et al (1969) Picroside I : A bitter principle of picrorhiza kurrooa. Tetrahedron Lett 10:3837–3840. doi: https://doi.org/10.1016/S0040-4039(01)88526-9

177. Herzog J, Hâncu V (1908) Zur Kenntnis des Pimpinellins. Arch Pharm (Weinheim) 246:402–414. doi: 10.1002/ardp.19082460603

178. Wessely F, Kallab F (1932) Über die Inhaltsstoffe der Wurzel von Pimpinella saxifraga I. Monatshefte für Chemie und verwandte Teile anderer Wissenshaften 59:162–175. doi: 10.1007/BF01638226

179. Dawid C, Hofmann T (2014) Quantitation and bitter taste contribution of saponins in fresh and cooked white asparagus (Asparagus officinalis L.). Food Chem 145:427–436. doi: https://doi.org/10.1016/j.foodchem.2013.08.057

180. Ganzera M, Sturm S (2017) Recent advances on HPLC/MS in medicinal plant analysis—An update covering 2011–2016. J Pharm Biomed Anal. doi: https://doi.org/10.1016/j.jpba.2017.07.038

181. Shimazaki N, Mimaki Y, Sashida Y (1991) Prunasin and acetylated phenylpropanoic acid sucrose esters, bitter principles from the fruits of Prunus jamasakura and P. maximowiczii. Phytochemistry 30:1475–1480. doi: https://doi.org/10.1016/0031-9422(91)84190-4

182. Mancuso G, Borgonovo G, Scaglioni L, Bassoli A (2015) Phytochemicals from Ruta graveolens Activate TAS2R Bitter Taste Receptors and TRP Channels Involved in Gustation and Nociception. Molecules 20:18907–18922. doi: 10.3390/molecules201018907

183. Goyal MR (2017) Samprapti vighatana chikitsa of mutrashmari (urinary calculus). J. Ayurveda Physicians Surg. (JAPS)(EISSN 2394-6350) 1:

184. Price KR, Johnson IT, Fenwick GR, Malinow MR (1987) The chemistry and biological significance of saponins in foods and feedingstuffs. C R C Crit Rev Food Sci Nutr 26:27–135. doi: 10.1080/10408398709527461

185. Santamour FS, Vettel HE (1978) The distribution of rhododendrin in birch (Betula) species. Biochem Syst Ecol 6:107–108.

186. Reichelt K V, Hoffmann-Lücke P, Hartmann B, et al (2012) Phytochemical characterization of South African bush tea (Athrixia phylicoides DC.). South African J Bot 83:1–8. doi: https://doi.org/10.1016/j.sajb.2012.07.006

187. Coelho M, Rocha C, Cunha LM, et al (2016) Influence of harvesting factors on sensory attributes and phenolic and aroma compounds composition of Cymbopogon citratus leaves infusions. Food Res Int 89:1029–1037. doi: 10.1016/j.foodres.2016.07.008

188. Chadwick M, Trewin H, Gawthrop F, Wagstaff C (2013) Sesquiterpenoids lactones: benefits to plants and people. Int J Mol Sci 14:12780–12805. doi: 10.3390/ijms140612780

189. Troszynska A (2004) Non-Nutrient Bioactive Substances in Food of Plant Origin Causing Bitterness and Astringency. Polish J Food Nutr Sci 13/54:65–73.

190. De Waal HL, Neethling LP, Perold GW (1960) Bitter principle of Solanum melongena (egg plant) fruits. J South African Chem Inst XIII:45–47.

191. Zitnak A, Filadelfi MA (1985) Estimation of Taste Thresholds of Three Potato Glycoalkaloids. Can Inst Food Sci Technol J 18:337–339. doi: https://doi.org/10.1016/S0315-5463(85)71970-0

192. Cardenas PD, Sonawane PD, Heinig U, et al (2015) The bitter side of the nightshades: Genomics drives discovery in Solanaceae steroidal alkaloid metabolism. Phytochemistry 113:24–32. doi: 10.1016/J.PHYTOCHEM.2014.12.010

193. Barceloux DG (2009) Potatoes, Tomatoes, and Solanine Toxicity (Solanum tuberosum L., Solanum lycopersicum L.). Disease-a-Month 55:391–402. doi: https://doi.org/10.1016/j.disamonth.2009.03.009

194. Takino Y, Koshioka M, Kawaguchi M, et al (1980) Quantitative determination of bitter components in Swertiae herba. Planta Med 38:351–355.

195. Schaufelberger D, Hostettmann K (1984) Flavonoid glycosides and a bitter principle from lomatogonium carinthiacum. Phytochemistry 23:787–789. doi: https://doi.org/10.1016/S0031-9422(00)85027-X

196. Imbabi E, Ibrahim K, Ahmed B, et al (1992) Chemical characterization of tamarind bitter principle, tamarindineal (tamarindienal). Fitotherapia 63:537–538.

197. Singh A, Malhotra S, Subban R (2008) Dandelion (Taraxacum officinale)-Hepatoprotective herb with therapeutic potential. Pharmacogn Rev 2:163.

198. Koo H-N, Hong S-H, Song B-K, et al (2004) Taraxacum officinale induces cytotoxicity through TNF-α and IL-1α secretion in Hep G2 cells. Life Sci 74:1149–1157. doi: https://doi.org/10.1016/j.lfs.2003.07.030

199. Han X, Jiang H, Han L, et al (2017) A novel quantified bitterness evaluation model for traditional Chinese herbs based on an animal ethology principle. Acta Pharm. Sin. B

200. Szejtli J, Szente L (2005) Elimination of bitter, disgusting tastes of drugs and foods by cyclodextrins. Eur J Pharm Biopharm 61:115–125. doi: 10.1016/j.ejpb.2005.05.006

201. Chatterjee A, GhOsh S (1960) Tinosporine, the furanoid bitter principle of Tinospora cordifolia Miers. Sci Cult 26:140–141.

202. Noble AC (1990) Bitterness and astringency in wine. In: Rouseff RL (ed) Bitternes foods beverages. Dev. Food Sci. vol. 25. Elsevier Science Publishers B.V., pp 146–158

203. Solms J (1969) Taste of amino acids, peptides, and proteins. J Agric Food Chem 17:686–688. doi: 10.1021/jf60164a016

204. Belitz HD, Chen W, Jugel H, et al (1979) Sweet and Bitter Compounds: Structure and Taste Relationship. In: Food Tast. Chem. AMERICAN CHEMICAL SOCIETY, pp 93–131

205. Seigler DS (1998) Plant Secondary Metabolism. Springer US

206. Hedin PA, Miles LR, Thompson AC, Minyard JP (1968) Constituents of cotton bud. Formulation of boll weevil feeding stimulant mixtures. J Agric Food Chem 16:505–513. doi: 10.1021/jf60157a003

207. Tsutsui K, Otoh M, Sakurai K, et al (2016) Variation in ligand responses of the bitter taste receptors TAS2R1 and TAS2R4 among New World monkeys. BMC Evol Biol 16:208. doi: 10.1186/s12862-016-0783-0

208. Xu H, Blair NT, Clapham DE (2005) Camphor activates and strongly desensitizes the transient receptor potential vanilloid subtype 1 channel in a vanilloid-independent mechanism. J Neurosci 25:8924–8937. doi: 10.1523/JNEUROSCI.2574-05.2005

209. Després L, David J-P, Gallet C (2007) The evolutionary ecology of insect resistance to plant chemicals. Trends Ecol Evol 22:298–307.

210. Hirasa K, Takemasa M (1998) Spice science and technology. CRC Press

211. Govindarajan VS (1979) Pungency: The Stimuli and Their Evaluation. In: Boudreau JC (ed) Food Tast. Chem. American Chemical Society, pp 3–53

212. García MJ, Prieto JL, Guevara A, et al (2016) Chemical Studies of Yellow Tamarillo (Solanum betaceum Cav.) Fruit Flavor by Using a Molecular Sensory Approach. Mol . doi: 10.3390/molecules21121729

213. Wagner H, Bladt S (1996) Drugs with Pungent-Tasting Principles BT - Plant Drug Analysis: A Thin Layer Chromatography Atlas. Springer Berlin Heidelberg, Berlin, Heidelberg, pp 291–303

214. Wishart DS, Knox C, Guo AC, et al (2009) HMDB: a knowledgebase for the human metabolome. Nucleic Acids Res 37:D603-10. doi: 10.1093/nar/gkn810

215. Ryan M (1835) Exhibiting a view of improvements and discoveries in the various branches of medical sciences. G. Henderson, 2, Old Bailey, Ludgate Hill, London

216. Bryant B, Mezine I (2002) Pungency and Tingling : Sensations and Mechanisms of Trigeminal Chemical Sensitivity. In: Given P, Paredes D (eds) Chem. Tast. American Chemical Society, pp 202–212

217. Cliff M, Heymann H (1992) Descriptive analyssi of oral pungency. J Sens Stud 7:279–290. doi: 10.1016/0950-3293(93)90367-F

218. Polya G (2003) Biochemical Targets of Plant Bioactive Compounds: A Pharmacological Reference Guide to Sites of Action and Biological Effects. CRC Press

219. Klein AH, Joe CL, Davoodi A, et al (2014) Eugenol and carvacrol excite first- and second-order trigeminal neurons and enhance their heat-evoked responses. Neuroscience 271:45–55. doi: https://doi.org/10.1016/j.neuroscience.2014.04.019

220. Zanotto KL, Merrill AW, Carstens MI, Carstens E (2007) Neurons in superficial trigeminal subnucleus caudalis responsive to oral cooling, menthol, and other irritant stimuli. J Neurophysiol 97:966–978. doi: 10.1152/jn.00996.2006

221. Cheron JB, Casciuc I, Golebiowski J, et al (2017) Sweetness prediction of natural compounds. Food Chem 221:1421–1425. doi: 10.1016/j.foodchem.2016.10.145

222. Steenge GR, Verhoef P, Katan MB (2003) Betaine Supplementation Lowers Plasma Homocysteine in Healthy Men and Women. J Nutr 133:1291–1295.

223. Olthof MR, van Vliet T, Boelsma E, Verhoef P (2003) Low Dose Betaine Supplementation Leads to Immediate and Long Term Lowering of Plasma Homocysteine in Healthy Men and Women. J Nutr 133:4135–4138.

224. Zhou Y, Hu S, Ma X, et al (2008) Synthesis of cyclic carbonates from carbon dioxide and epoxides over betaine-based catalysts. J Mol Catal A Chem 284:52–57. doi: https://doi.org/10.1016/j.molcata.2008.01.010

225. Taylor NW (1928) A physico-chemical theory of sweet and bitter taste excitation based on the properties of the plasma membrane. Protoplasma 4:1–17. doi: 10.1007/BF01607954

226. Ahmed J, Preissner S, Dunkel M, et al (2011) SuperSweet—a resource on natural and artificial sweetening agents. Nucleic Acids Res 39:D377–D382. doi: 10.1093/nar/gkq917

227. Steinhardt RG, Calvin AD, Dodd EA (1962) Taste-Structure Correlation with α-D-Mannose and β-D-Mannose. Science (80- ) 135:367 LP-368.

228. Otero-Losada ME (1999) A kinetic study on benzoic acid pungency and sensory attributes of benzoic acid. Chem Senses 24:245–253.

229. Gautschit M, Yangt X, Eilermant RG, Fratert G (1998) Flavor chemicals with pungent properties. In: Teranishi R, Wick EL, Homstein I (eds) Flavor Chem. Thirty Years Prog. Springer Science & Business Media, pp 199–210

230. Pancharoen O, Prawat U, Tuntiwachwuttikul P (2000) Phytochemistry of the zingiberaceae. Stud Nat Prod Chem 23:797–865. doi: 10.1016/S1572-5995(00)80142-8

231. Yang X, Eilerman RG (1999) Pungent Principal of Alpinia galangal (L.) Swartz and Its Applications. J Agric Food Chem 47:1657–1662. doi: 10.1021/jf9808224

232. Bautista DM, Movahed P, Hinman A, et al (2005) Pungent products from garlic activate the sensory ion channel TRPA1. Proc Natl Acad Sci U S A 102:12248–12252. doi: 10.1073/pnas.0505356102

233. Macpherson LJ, Geierstanger BH, Viswanath V, et al (2005) The pungency of garlic: activation of TRPA1 and TRPV1 in response to allicin. Curr Biol 15:929–934. doi: 10.1016/j.cub.2005.04.018

234. Zhao C, Zeng Y, Wan M, et al (2009) Comparative analysis of essential oils from eight herbal medicines with pungent flavor and cool nature by GC–MS and chemometric resolution methods. J Sep Sci 32:660–670. doi: 10.1002/jssc.200800484

235. Kiple KF, Ornelas KC (2000) The Cambridge world history of food. Vol.1. Cambridge University Press

236. Masada Y, Hashimoto K, Inoue T, Suzuki M (1971) Analysis of the pungent principles of capsicum annuum by combined gas chromatography-mass spectrometry. J Food Sci 36:858–860. doi: 10.1111/j.1365-2621.1971.tb15544.x

237. Kosuge S, Furuta M (1970) Studies on the Pungent Principle of Capsicum. Agric Biol Chem 34:248–256. doi: 10.1080/00021369.1970.10859594

238. Sarpras M, Gaur R, Sharma V, et al (2016) Comparative analysis of fruit metabolites and pungency candidate genes expression between Bhut jolokia and other Capsicum species. PLoS One 11:1–19. doi: 10.1371/journal.pone.0167791

239. Borges RM (2001) Why are chillies pungent? J Biosci 26:289–291.

240. Kasanen J-P, Pasanen A-L, Pasanen P, et al (1999) Evaluation of sensory irritation of 3-carene and turpentine, and acceptable levels of monoterpenes in occupational and indoor environment. J Toxicol Environ Heal Part A 57:89–114. doi: 10.1080/009841099157809

241. Klein AH, Carstens MI, Carstens E (2013) Eugenol and carvacrol induce temporally desensitizing patterns of oral irritation and enhance innocuous warmth and noxious heat sensation on the tongue. PAIN® 154:2078–2087. doi: https://doi.org/10.1016/j.pain.2013.06.025

242. Bandell M, Story GM, Hwang SW, et al (2004) Noxious cold ion channel TRPA1 is activated by pungent compounds and bradykinin. Neuron 41:849–857.

243. Stotz SC, Vriens J, Martyn D, et al (2008) Citral Sensing by TRANSient Receptor Potential Channels in Dorsal Root Ganglion Neurons. PLoS One 3:e2082. doi: 10.1371/journal.pone.0002082

244. Dubey PN, Saxena SN, Mishra BK, et al (2017) Preponderance of cumin (Cuminum cyminum L.) essential oil constituents across cumin growing Agro-Ecological Sub Regions, India. Ind Crops Prod 95:50–59. doi: https://doi.org/10.1016/j.indcrop.2016.10.011

245. Chen CC, Ho CT (1988) Gas chromatographic analysis of volatile components of ginger oil (Zingiber officinale Roscoe) extracted with liquid carbon dioxide. J Agric Food Chem 36:322–328. doi: 10.1021/jf00080a020

246. Machmudah S, Izumi T, Sasaki M, Goto M (2009) Extraction of pungent components from Japanese pepper (Xanthoxylum piperitum DC.) using supercritical CO2. Sep Purif Technol 68:159–164. doi: https://doi.org/10.1016/j.seppur.2009.04.021

247. Holzer P (2011) Transient receptor potential (TRP) channels as drug targets for diseases of the digestive system. Pharmacol Ther 131:142–170. doi: https://doi.org/10.1016/j.pharmthera.2011.03.006

248. Behrendt H-J, Germann T, Gillen C, et al (2004) Characterization of the mouse cold-menthol receptor TRPM8 and vanilloid receptor type-1 VR1 using a fluorometric imaging plate reader (FLIPR) assay. Br J Pharmacol 141:737–745. doi: 10.1038/sj.bjp.0705652

249. Zachariah TJ (2008) Ginger. In: Parthasarathy VA, Chempakam B, Zachariah TJ (eds) Chem. spices. CABI, Cambridge, pp 70–96

250. He X, Bernart MW, Lian L, Lin L (1998) High-performance liquid chromatography–electrospray mass spectrometric analysis of pungent constituents of ginger. J Chromatogr A 796:327–334. doi: https://doi.org/10.1016/S0021-9673(97)01013-3

251. Narasimhan S, Govindarajan VS (1978) Evaluation of spices and oleoresin-VI-pungency of ginger components, gingerols and shogoals and quality. Int J Food Sci Technol 13:31–36. doi: 10.1111/j.1365-2621.1978.tb00773.x

252. Boonen J, Bronselaer A, Nielandt J, et al (2012) Alkamid database: Chemistry, occurrence and functionality of plant N-alkylamides. J Ethnopharmacol 142:563–590. doi: https://doi.org/10.1016/j.jep.2012.05.038

253. Zrybko CL, Fukuda EK, Rosen RT (1997) Determination of glucosinolates in domestic and wild mustard by high-performance liquid chromatography with confirmation by electrospray mass spectrometry and photodiode-array detection. J Chromatogr A 767:43–52. doi: https://doi.org/10.1016/S0021-9673(96)01068-0

254. Oz M, Lozon Y, Sultan A, et al (2015) Effects of monoterpenes on ion channels of excitable cells. Pharmacol Ther 152:83–97. doi: https://doi.org/10.1016/j.pharmthera.2015.05.006

255. Jakribettu RP, Boloor R, Bhat HP, et al (2016) Ginger (Zingiber officinale Rosc.) Oils. In: Preedy VR (ed) Essent. Oils Food Preserv. Flavor Saf. Academic Press, Elsevier, pp 447–454

256. Gulland JM, Hopton GU (1930) II.-Pellitorine, the pungent principle of Anacyclus pyrethrum. J Chem Soc 6–11. doi: 10.1039/JR9300000006

257. Traxler JT (1971) Piperanine, a pungent component of black pepper. J Agric Food Chem 19:1135–1138. doi: 10.1021/jf60178a026

258. Strunz GM (2000) Unsaturated amides from piper species (Piperaceae). Stud Nat Prod Chem 24:683–738. doi: 10.1016/S1572-5995(00)80053-8

259. Beltrán LR, Dawid C, Beltrán M, et al (2013) The pungent substances piperine, capsaicin, 6-gingerol and polygodial inhibit the human two-pore domain potassium channels TASK-1, TASK-3 and TRESK. Front. Pharmacol. 4:

260. Suekawa M, Ishige A, Yuasa K, et al (1984) Pharmacological studies on ginger. I. Pharmacological actions of pungent constitutents, (6)-gingerol and (6)-shogaol. J Pharmacobiodyn 7:836–848.

261. Surburg H, Guentert M, Harder H (1993) Volatile Compounds from Flowers. In: Bioact. Volatile Compd. from Plants. American Chemical Society, pp 13–168

262. Sethi S, Gupta S (2016) Antimicrobial Spices: Use in Antimicrobial Packaging. In: Barros-Velázquez JBT-AFP (ed) Antimicrob. food Packag. Academic Press, San Diego, pp 433–444

263. Ndunda B (2014) Phytochemistry and bioactivity investigations of three Kenyan Croton species.

264. Xu H, Delling M, Jun JC, Clapham DE (2006) Oregano, thyme and clove-derived flavors and skin sensitizers activate specific TRP channels. Nat Neurosci 9:628–635. doi: 10.1038/nn1692

265. Nomura H (1917) Pungent principles of ginger. I. A new ketone, zingiberone, occurring in ginger. Sci Rep Tohoku Imp Univ 6:41–52.

266. Kim Y-S, Sik Hong C, Weon Lee S, et al (2017) Effects of Ginger and its Pungent Constituents on Transient Receptor Potential Channels. Biophys J 112:250a. doi: https://doi.org/10.1016/j.bpj.2016.11.1367

267. Baranowski JD (1985) High-performance liquid chromatographic separation of pungency components of ginger. J Chromatogr A 319:471–474. doi: https://doi.org/10.1016/S0021-9673(01)90593-X

268. Monge P, Scheline R, Solheim E (1976) The metabolism of zingerone, a pungent principle of ginger. Xenobiotica 6:411–423. doi: 10.3109/00498257609151654

269. Karibasappa GS (1987) Post harvest studies in large cardamom (Amomum sublatum Roxb.). Sikk Sci Soc Newsl 6:2–10.

270. Chempakam B, Parthasarathy VA (2008) Turmeric. In: Parthasarathy VA, Chempakam B, Zachariah TJ (eds) Chem. spices. CABI, Cambridge, pp 97–123

271. Miyazawa M, Shindo M (2001) Biotransformation of 1,8-Cineole by Human Liver Microsomes. Nat Prod Lett 15:49–53. doi: 10.1080/10575630108041257

272. Van Der Klaauw NJ, Smith D V. (1995) Taste quality profiles for fifteen organic and inorganic salts. Physiol Behav 58:295–306. doi: 10.1016/0031-9384(95)00056-O

273. DeMan JM (1999) Principles of food chemistry, 3rd ed. Aspen Publishers, New York

274. Neta ERDC, Johanningsmeier SD, Mcfeeters RF (2007) The Chemistry and Physiology of Sour Taste—A Review. J Food Sci 72:R33-8. doi: 10.1111/j.1750-3841.2007.00282.x

275. Wishart D, Arndt D, Pon A, et al (2015) T3DB: the toxic exposome database. Nucleic Acids Res 43:D928-34. doi: 10.1093/nar/gku1004

276. Kinghorn AD, Choi YH (1993) Natural intense sweeteners. https://www.google.com/patents/US5198427

277. Kim N-C, Kinghorn AD (2002) Sweet-tasting and sweetness-modifying constituents of plants. In: Atta-ur-Rahman BT-S in NPC (ed) Bioact. Nat. Prod. (Part H). Elsevier, pp 3–57

278. de Cock P (1999) Erythritol: a novel noncaloric sweetener ingredient. In: Low-Calories Sweeten. Present Futur. Karger Publishers, pp 110–116

279. Lee C-K, Birch GG (1975) Structural functions of taste in the sugar series: Binding characteristics of disaccharides. J Sci Food Agric 26:1513–1521. doi: 10.1002/jsfa.2740261010

280. Baker JL, Pope TH (1900) LVIII.-Mannogalactan and laevulomannan. Two new polysaccharides. J Chem Soc Trans 77:696–705. doi: 10.1039/CT9007700696

281. Schimpf K, Thompson L, Baugh S (2012) Determination of Myo-Inositol (Free and Bound as Phosphatidylinositol) in Infant Formula and Adult Nutritionals by Liquid Chromatography/Pulsed Amperometry with Column Switching: First Action 2011.18. J AOAC Int 95:937–942. doi: 10.5740/jaoacint.CS2011_18

282. Park C, Lee J-S (2012) Mini Review: Natural ingredients for diabetes which are approved by Korean FDA. Biomed. Res. 24:

283. Jakinovich William J, Sugarman D (1988) Sugar taste reception in mammals. Chem Senses 13:13–31.

284. Yu H, Zhao J, Li F, et al (2015) Characterization of Chinese rice wine taste attributes using liquid chromatographic analysis, sensory evaluation, and an electronic tongue. J Chromatogr B 997:129–135. doi: https://doi.org/10.1016/j.jchromb.2015.05.037

285. Morris WL, Ross HA, Ducreux LJM, et al (2007) Umami Compounds Are a Determinant of the Flavor of Potato (Solanum tuberosum L.). J Agric Food Chem 55:9627–9633. doi: 10.1021/jf0717900

286. Yamaguchi S (1979) The Umami Taste. In: Boudreau JC (ed) Food Tast. Chem. AMERICAN CHEMICAL SOCIETY, pp 2–33
