# Supplementary material for: PhytoMolecularTasteDB: An integrative database on the “molecular taste” of Indian medicinal plants
Source: Data Brief. 2018 Apr 21;19:1237–41. doi: 10.1016/j.dib.2018.04.048 (PMC6141601; doi:10.1016/j.dib.2018.04.048)
Supplement: Supplementary material [file mmc3.docx]

# References for chemical composition of plants found in PhytoMolecularTasteDB

1. Abdel-Khalik, S. M., T. Miyase, F. R. Melek, and H. A. el-Ashaal. 2001. “Further Saponins from Fagonia Cretica.” Die Pharmazie 56(3):247–50.
2. Abdel Khalik, S. M., T. Miyase, H. A. El-Ashaal, and F. R. Melek. 2000. “Triterpenoid Saponins from Fagonia Cretica.” Phytochemistry 54(8):853–59.
3. Abdullah, Fauziah et al. 2015. “Chemical Composition, Antifeedant, Repellent, and Toxicity Activities of the Rhizomes of Galangal, Alpinia Galanga against Asian Subterranean Termites, Coptotermes Gestroi and Coptotermes Curvignathus (Isoptera: Rhinotermitidae).” Journal of insect science (Online) 15:175.
4. Aberham, Anita, Serhat Sezai Cicek, Peter Schneider, and Hermann Stuppner. 2010. “Analysis of Sesquiterpene Lactones, Lignans, and Flavonoids in Wormwood (Artemisia Absinthium L.) Using High-Performance Liquid Chromatography (HPLC)-Mass Spectrometry, Reversed Phase HPLC, and HPLC-Solid Phase Extraction-Nuclear Magnetic Resonance.” Journal of agricultural and food chemistry 58(20):10817–23.
5. Abhilash, J. and M. Haridas. 2015. “Metal Ion Coordination Essential for Specific Molecular Interactions of Butea Monosperma Lectin: ITC and MD Simulation Studies.” Applied biochemistry and biotechnology 176(1):277–86.
6. Abrantes, Vanessa Erika Ferreira et al. 2013. “Molecular Modeling of Lectin-like Protein from Acacia Farnesiana Reveals a Possible Anti-Inflammatory Mechanism in Carrageenan-Induced Inflammation.” BioMed research international 2013:253483.
7. Acharya, Niyati, Sanjeev Acharya, Unnati Shah, Ripal Shah, and Lal Hingorani. 2016. “A Comprehensive Analysis on Symplocos Racemosa Roxb.: Traditional Uses, Botany, Phytochemistry and Pharmacological Activities.” Journal of ethnopharmacology 181:236–51.
8. Adewuyi, Adewale and Rotimi Ayodele Oderinde. 2014. “Fatty Acid Composition and Lipid Profile of Diospyros Mespiliformis, Albizia Lebbeck, and Caesalpinia Pulcherrima Seed Oils from Nigeria.” International journal of food science 2014:283614.
9. Affonso, Regina Celis Lopes et al. 2016. “Phytochemical Composition, Antioxidant Activity, and the Effect of the Aqueous Extract of Coffee (Coffea Arabica L.) Bean Residual Press Cake on the Skin Wound Healing.” Oxidative medicine and cellular longevity 2016:1923754.
10. Agarkar, Shruti A. et al. 2011. “Isobutrin from Butea Monosperma (Flame of the Forest): A Promising New Natural Sensitizer Belonging to Chalcone Class.” ACS applied materials & interfaces 3(7):2440–44.
11. Agnihotri, Supriya A., Sharad R. Wakode, and Mohammed Ali. 2012. “Chemical Composition, Antimicrobial and Topical Anti-Inflammatory Activity of Essential Oil of Amomum Subulatum Fruits.” Acta poloniae pharmaceutica 69(6):1177–81.
12. Agrawal, Jyoti and Anirban Pal. 2013. “Nyctanthes Arbor-Tristis Linn--a Critical Ethnopharmacological Review.” Journal of ethnopharmacology 146(3):645–58.
13. Agrawal, Nisha et al. 2013. “Butanolides from Methanolic Extract of Litsea Glutinosa.” Chemistry & biodiversity 10(3):394–400.
14. Aguiar, Sebastian and Thomas Borowski. 2013. “Neuropharmacological Review of the Nootropic Herb Bacopa Monnieri.” Rejuvenation research 16(4):313–26.
15. Ahad, Hindustan Abdul, B.Suma Padmaja, M. Sravanthi, P. Ramyasree, and K. Kavitha. 2012. “Phytochemical Screening and Anti-Inflammatory Actions of Alangium Salviifolium Root Extract.” Natural product research 26(17):1649–53.
16. Ahmad, Bashir, Haroon Khan, Shumaila Bashir, and Murad Ali. 2006. “Antimicrobial Bioassay of Colchicum Luteum Baker.” Journal of enzyme inhibition and medicinal chemistry 21(6):765–69.
17. Ahmad, Hafsa, Sakshi Sehgal, Anurag Mishra, and Rajiv Gupta. 2012. “Mimosa Pudica L. (Laajvanti): An Overview.” Pharmacognosy reviews 6(12):115–24.
18. Ahmad, M. U., S. K. Husain, M. Ahmad, and S. M. Osman. 1976. “Cyclopropenoid Fatty Acids in Seed Oils of Sida Acuta and Sida Rhombifolia (Malvaceae).” Journal of the American Oil Chemists’ Society 53(11):698–99.
19. Ahmad, Manzoor et al. 2008. “Norditerpenoid Alkaloids from the Roots of Aconitum Heterophyllum Wall with Antibacterial Activity.” Journal of enzyme inhibition and medicinal chemistry 23(6):1018–22.
20. Ahmad, Naseem Saud, Muhammad Farman, Muzammil Hasan Najmi, Kouser Bashir Mian, and Aurangzeb Hasan. 2008. “Pharmacological Basis for Use of Pistacia Integerrima Leaves in Hyperuricemia and Gout.” Journal of ethnopharmacology 117(3):478–82.
21. Ahmad, Saeed et al. 2010. “Antioxidant Flavonoids from Alhagi Maurorum.” Journal of Asian natural products research 12(2):138–43.
22. Ahmad, Saeed, Ayesha Hassan, Waheed Mumtaz Abbasi, and Tayyeba Rehman. 2017. “Phytochemistry and Pharmacological Potential of Cassia Absus - a Review.” The Journal of pharmacy and pharmacology.
23. Ahmed, Ajaz, Seema Akbar, and Wajaht Amin Shah. 2017. “Chemical Composition and Pharmacological Potential of Aromatic Water from Salix Caprea Inflorescence.” Chinese journal of integrative medicine.
24. Ahmed, Danish, Manju Sharma, Vikas Kumar, Harish Kumar Bajaj, and Amita Verma. 2015. “2beta-Hydroxybetulinic Acid 3beta-Caprylate: An Active Principle from Euryale Ferox Salisb. Seeds with Antidiabetic, Antioxidant, Pancreas & Hepatoprotective Potential in Streptozotocin Induced Diabetic Rats.” Journal of food science and technology 52(9):5427–41.
25. Ahmed, Dildar, Munim Fatima, and Sana Saeed. 2014. “Phenolic and Flavonoid Contents and Anti-Oxidative Potential of Epicarp and Mesocarp of Lagenaria Siceraria Fruit: A Comparative Study.” Asian Pacific journal of tropical medicine 7S1:S249-55.
26. Ahmed, Dildar, Muhammad Mehboob Khan, and Ramsha Saeed. 2015. “Comparative Analysis of Phenolics, Flavonoids, and Antioxidant and Antibacterial Potential of Methanolic, Hexanic and Aqueous Extracts from Adiantum Caudatum Leaves.” Antioxidants (Basel, Switzerland) 4(2):394–409.
27. Ahmed, Dildar, Saman Zara, and Hira Baig. 2012. “In Vitro Analysis of Antioxidant Activities of Oxalis Corniculata Linn. Fractions in Various Solvents.” African journal of traditional, complementary, and alternative medicines : AJTCAM 10(1):158–65.
28. Ahmed, F., M. Ali, and O. Singh. 2006. “New Compounds from Commiphora Myrrha (Nees) Engl.” Die Pharmazie 61(8):728–31.
29. Ahmed, Hanaa H., Ahmed A. Abd-Rabou, Amal Z. Hassan, and Soheir E. Kotob. 2015. “Phytochemical Analysis and Anti-Cancer Investigation of Boswellia Serrata Bioactive Constituents In Vitro.” Asian Pacific journal of cancer prevention : APJCP 16(16):7179–88.
30. Ahmed, Shabina Ishtiaq et al. 2016. “Pharmacologically Active Flavonoids from the Anticancer, Antioxidant and Antimicrobial Extracts of Cassia Angustifolia Vahl.” BMC complementary and alternative medicine 16(1):460.
31. Ahmed Hamdi, Omer Abdalla et al. 2014. “Cytotoxic Constituents from the Rhizomes of Curcuma Zedoaria.” TheScientificWorldJournal 2014:321943.
32. Akter, Raushanara, Shaikh J. Uddin, Joe Tiralongo, I.Darren Grice, and Evelin Tiralongo. 2015. “A New Cytotoxic Steroidal Glycoalkaloid from the Methanol Extract of Blumea Lacera Leaves.” Journal of pharmacy & pharmaceutical sciences : a publication of the Canadian Society for Pharmaceutical Sciences, Societe canadienne des sciences pharmaceutiques 18(4):616–33.
33. Akter, Raushanara, Shaikh J. Uddin, Joe Tiralongo, I.Darren Grice, and Evelin Tiralongo. 2016. “A New Cytotoxic Diterpenoid Glycoside from the Leaves of Blumea Lacera and Its Effects on Apoptosis and Cell Cycle.” Natural product research 1–6.
34. Al-Alawi, Reem A., Jawhara H. Al-Mashiqri, Jawaher S. M. Al-Nadabi, Badria I. Al-Shihi, and Younis Baqi. 2017. “Date Palm Tree (Phoenix Dactylifera L.): Natural Products and Therapeutic Options.” Frontiers in plant science 8:845.
35. Al-Asmari, Abdulrahman Khazim, Md Tanwir Athar, and Saeed G. Kadasah. 2017. “An Updated Phytopharmacological Review on Medicinal Plant of Arab Region: Apium Graveolens Linn.” Pharmacognosy reviews 11(21):13–18.
36. Al-Massarani, Shaza M., Ali A. El Gamal, Perwez Alam, et al. 2017. “Isolation, Biological Evaluation and Validated HPTLC-Quantification of the Marker Constituent of the Edible Saudi Plant Sisymbrium Irio L.” Saudi pharmaceutical journal : SPJ : the official publication of the Saudi Pharmaceutical Society 25(5):750–59.
37. Al-Massarani, Shaza M., Ali A. El Gamal, Mohamed F. Abd El Halim, et al. 2017. “New Acyclic Secondary Metabolites from the Biologically Active Fraction of Albizia Lebbeck Flowers.” Saudi pharmaceutical journal : SPJ : the official publication of the Saudi Pharmaceutical Society 25(1):110–19.
38. Al-Qudah, Mahmoud A. and Musa H. Abu Zarga. 2010. “Chemical Constituents of Sisymbrium Irio L. from Jordan.” Natural product research 24(5):448–56.
39. Al-Reza, Sharif M., Atiqur Rahman, M. A. Sattar, M.Oliur Rahman, and Hasan M. Fida. 2010. “Essential Oil Composition and Antioxidant Activities of Curcuma Aromatica Salisb.” Food and chemical toxicology : an international journal published for the British Industrial Biological Research Association 48(6):1757–60.
40. Al-Taweel, Areej Mohammad et al. 2017. “Evaluation of Antiulcer and Cytotoxic Potential of the Leaf, Flower, and Fruit Extracts of Calotropis Procera and Isolation of a New Lignan Glycoside.” Evidence-based complementary and alternative medicine : eCAM 2017:8086791.
41. Alam, Mahboob et al. 2016. “Bioassay-Guided Isolation of Sesquiterpene Coumarins from Ferula Narthex Bioss: A New Anticancer Agent.” Frontiers in pharmacology 7:26.
42. Alamelumangai, Muthukumar et al. 2014. “In Vitro Studies on Phytochemical Evaluation and Antimicrobial Activity of Borassus Flabellifer Linn against Some Human Pathogens.” Asian Pacific journal of tropical medicine 7S1:S182-5.
43. Ali, Mohammad and Nisha Chaudhary. 2011. “Ficus Hispida Linn.: A Review of Its Pharmacognostic and Ethnomedicinal Properties.” Pharmacognosy reviews 5(9):96–102.
44. Almagro, Lorena, Francisco Fernandez-Perez, and Maria Angeles Pedreno. 2015. “Indole Alkaloids from Catharanthus Roseus: Bioproduction and Their Effect on Human Health.” Molecules (Basel, Switzerland) 20(2):2973–3000.
45. Althaus, Julia B., Claudine Malyszek, Marcel Kaiser, Reto Brun, and Thomas J. Schmidt. 2017. “Alkamides from Anacyclus Pyrethrum L. and Their in Vitro Antiprotozoal Activity.” Molecules (Basel, Switzerland) 22(5).
46. Alves, Susana, Andreia Duarte, Sonia Sousa, and Fernanda C. Domingues. 2016. “Study of the Major Essential Oil Compounds of Coriandrum Sativum against Acinetobacter Baumannii and the Effect of Linalool on Adhesion, Biofilms and Quorum Sensing.” Biofouling 32(2):155–65.
47. Amalraj, Augustine and Sreeraj Gopi. 2017. “Medicinal Properties of Terminalia Arjuna (Roxb.) Wight & Arn.: A Review.” Journal of traditional and complementary medicine 7(1):65–78.
48. Ben Amar, Mohamed. 2006. “Cannabinoids in Medicine: A Review of Their Therapeutic Potential.” Journal of ethnopharmacology 105(1–2):1–25.
49. Amin, Adnan et al. 2016. “Antiprotozoal and Antiglycation Activities of Sesquiterpene Coumarins from Ferula Narthex Exudate.” Molecules (Basel, Switzerland) 21(10).
50. Angioni, Alberto, Andrea Barra, Valentina Coroneo, Sandro Dessi, and Paolo Cabras. 2006. “Chemical Composition, Seasonal Variability, and Antifungal Activity of Lavandula Stoechas L. Ssp. Stoechas Essential Oils from Stem/leaves and Flowers.” Journal of agricultural and food chemistry 54(12):4364–70.
51. Anilkumar, Kotha et al. 2017. “Evaluation of Anti-Inflammatory Properties of Isoorientin Isolated from Tubers of Pueraria Tuberosa.” Oxidative medicine and cellular longevity 2017:5498054.
52. Anjum, Adeeba, M. Ekramul Haque, M. Mukhlesur Rahman, and Satyajit D. Sarker. 2002. “Antibacterial Compounds from the Flowers of Alangium Salviifolium.” Fitoterapia 73(6):526–28.
53. Ansari, Vaseem Ahmad, Muhammad Arif, Md Sarfaraj Hussain, Hefazat Hussain Siddiqui, and Rakesh Kumar Dixit. 2016. “New 4’-substituted Benzoyl-Beta-D Glycoside from the Fruit Pulp of Terminalia Belerica with Antiplatelet and Antioxidant Potency.” Integrative medicine research 5(4):317–23.
54. Arimboor, Ranjith, Meena Rangan, S. G. Aravind, and C. Arumughan. 2011. “Tetrahydroamentoflavone (THA) from Semecarpus Anacardium as a Potent Inhibitor of Xanthine Oxidase.” Journal of ethnopharmacology 133(3):1117–20.
55. Arulmozhi, D. K., A. Veeranjaneyulu, S. L. Bodhankar, and S. K. Arora. 2005. “Effect of Sapindus Trifoliatus on Hyperalgesic in Vivo Migraine Models.” Brazilian journal of medical and biological research = Revista brasileira de pesquisas medicas e biologicas 38(3):469–75.
56. Arung, Enos Tangke, Kuniyoshi Shimizu, Hiroyuki Tanaka, and Ryuichiro Kondo. 2010. “3-Prenyl Luteolin, a New Prenylated Flavone with Melanin Biosynthesis Inhibitory Activity from Wood of Artocarpus Heterophyllus.” Fitoterapia 81(6):640–43.
57. Arung, Enos Tangke, Keisuke Yoshikawa, Kuniyoshi Shimizu, and Ryuichiro Kondo. 2010. “Isoprenoid-Substituted Flavonoids from Wood of Artocarpus Heterophyllus on B16 Melanoma Cells: Cytotoxicity and Structural Criteria.” Fitoterapia 81(2):120–23.
58. Arya, Aditya et al. 2012. “Chloroform Fraction of Centratherum Anthelminticum (L.) Seed Inhibits Tumor Necrosis Factor Alpha and Exhibits Pleotropic Bioactivities: Inhibitory Role in Human Tumor Cells.” Evidence-based complementary and alternative medicine : eCAM 2012:627256.
59. Asghar, Syeda Farina, Habib-ur-Rehman, Atta-ur-Rahman, and M.Iqbal Choudhary. 2010. “Phytochemical Investigations on Iris Germanica.” Natural product research 24(2):131–39.
60. Ashour, Mohamed Abdel-Ghaffar. 2012. “Isolation, HPLC/UV Characterization and Antioxidant Activity of Phenylethanoids from Blepharis Edulis (Forssk.) Pers. Growing in Egypt.” Bulletin of Faculty of Pharmacy, Cairo University 50(1):67–72. Retrieved (http://www.sciencedirect.com/science/article/pii/S1110093112000129).
61. Ashour, Mohamed Abdel-Ghaffar. 2015. “New Diacyl Flavonoid Derivatives from the Egyptian Plant Blepharis Edulis (Forssk.) Pers.” Bulletin of Faculty of Pharmacy, Cairo University 53(1):11–17. Retrieved (http://www.sciencedirect.com/science/article/pii/S1110093115000046).
62. Asif, Mohammad and Arun Kumar. 2011. “Phytochemical Investigation and Evaluation of Antinociceptive Activity of Ethanolic Extract of Dalbergia Sissoo (Roxb.) Bark.” Journal of natural science, biology, and medicine 2(1):76–79.
63. Astuti, Puji, Sudarsono Sudarsono, Khoirun Nisak, and Giri Wisnu Nugroho. 2014. “Endophytic Fungi Isolated from Coleus Amboinicus Lour Exhibited Antimicrobial Activity.” Advanced pharmaceutical bulletin 4(Suppl 2):599–605.
64. Ata, Athar, Elikana M. Gale, and Radhika Samarasekera. 2009. “Bioactive Chemical Constituents of Caesalpinia Bonduc (Fabaceae).” Phytochemistry Letters 2(3):106–9. Retrieved (http://www.sciencedirect.com/science/article/pii/S1874390009000238).
65. Atarzadeh, Fatemeh et al. 2017. “Cassia Fistula: A Remedy from Traditional Persian Medicine for Treatment of Cutaneous Lesions of Pemphigus Vulgaris.” Avicenna journal of phytomedicine 7(2):107–15.
66. Atawodi, S. E. et al. 2009. “Polyphenol Composition and Antioxidant Potential of Hibiscus Esculentus L. Fruit Cultivated in Nigeria.” Journal of medicinal food 12(6):1316–20.
67. Atiya, Akhtar, Barij Nayan Sinha, and Uma Ranjan Lal. 2017. “New Chemical Constituents from the Piper Betle Linn. (Piperaceae).” Natural product research 1–8.
68. Attarde, D. L., V. M. Aurangabadkar, D. P. Belsare, and S. C. Pal. 2008. “Report: Quantitative Estimation of Beta-Sitosterol, Lupeol, Quercetin and Quercetin Glycosides from Leaflets of Soymida Febrifuga Using HPTLC Technique.” Pakistan journal of pharmaceutical sciences 21(3):316–19.
69. Auwal, Mohammed Shaibu et al. 2014. “Preliminary Phytochemical and Elemental Analysis of Aqueous and Fractionated Pod Extracts of Acacia Nilotica (Thorn Mimosa).” Veterinary research forum : an international quarterly journal 5(2):95–100.
70. Avula, Bharathi et al. 2016. “Simultaneous Determination of Aegeline and Six Coumarins from Different Parts of the Plant Aegle Marmelos Using UHPLC-PDA-MS and Chiral Separation of Aegeline Enantiomers Using HPLC-ToF-MS.” Planta medica 82(6):580–88.
71. Awal, A., Q. N. Haq, M. A. Quader, and M. Ahmed. 1995. “Structural Study of a Polysaccharide from the Seeds of Borassus Flabellifer Linn.” Carbohydrate research 277(1):189–95.
72. Awale, Suresh et al. 2006. “Constituents of Caesalpinia Crista from Indonesia.” Chemical & pharmaceutical bulletin 54(2):213–18.
73. Awale, Suresh et al. 2009. “Cytotoxic Constituents of Soymida Febrifuga from Myanmar.” Journal of natural products 72(9):1631–36.
74. Ayyanar, Muniappan and Pandurangan Subash-Babu. 2012. “Syzygium Cumini (L.) Skeels: A Review of Its Phytochemical Constituents and Traditional Uses.” Asian Pacific journal of tropical biomedicine 2(3):240–46.
75. Azevedo, C. R. et al. 2006. “Isolation and Intracellular Localization of Insulin-like Proteins from Leaves of Bauhinia Variegata.” Brazilian journal of medical and biological research = Revista brasileira de pesquisas medicas e biologicas 39(11):1435–44.
76. Azhar-ul-Haq, Abdul Malik, Anwar-ul-Haq Sher Bahadar Khan, Muhammad Raza Shah, and Pir Muhammad. 2004. “Spinoside, New Coumaroyl Flavone Glycoside from Amaranthus Spinosus.” Archives of pharmacal research 27(12):1216–19.
77. Baananou, Sameh et al. 2013. “Extraction of the Volatile Oil from Carum Carvi of Tunisia and Lithuania by Supercritical Carbon Dioxide: Chemical Composition and Antiulcerogenic Activity.” Natural product research 27(22):2132–36.
78. Baba, Shoib A. and Shahid A. Malik. 2014. “Evaluation of Antioxidant and Antibacterial Activity of Methanolic Extracts of Gentiana Kurroo Royle.” Saudi journal of biological sciences 21(5):493–98.
79. Backes, Michael et al. 2015. “Rubemamine and Rubescenamine, Two Naturally Occurring N-Cinnamoyl Phenethylamines with Umami-Taste-Modulating Properties.” Journal of Agricultural and Food Chemistry 63(39):8694–8704.
80. Badami, Shrishailappa et al. 2004. “Determination of Betulin in Grewia Tiliaefolia by HPTLC.” Journal of separation science 27(1–2):129–31.
81. Badami, Shrishailappa, Sujay R. Rai, Sudheer Moorkoth, S. Rajan, and B. Suresh. 2003. “Pharmacognostical Evaluation of Caesalpinia Sappan Heartwood.” Ancient science of life 23(2):100–107.
82. Badole, Sachin L., Swapnil M. Chaudhari, Ganesh B. Jangam, Amit D. Kandhare, and Subhash L. Bodhankar. 2015. “Cardioprotective Activity of Pongamia Pinnata in Streptozotocin-Nicotinamide Induced Diabetic Rats.” BioMed research international 2015:403291.
83. Badwaik, Hemant, Mukesh Kumar Singh, Deepa Thakur, Tapan Kumar Giri, and D. .. Tripathi. 2011. “The Botany, Chemistry, Pharmacological and Therapeutic Application of Oxalis Corniculata Linn– A Review.” International Journal of Phytomedicine 3:01–08.
84. Bae, Gi-Sang et al. 2012. “Nardostachys Jatamansi Inhibits Severe Acute Pancreatitis via Mitogen-Activated Protein Kinases.” Experimental and therapeutic medicine 4(3):533–37.
85. Baek, N. I. et al. 2000. “Anticonvulsant Compounds from the Wood of Caesalpinia Sappan L.” Archives of pharmacal research 23(4):344–48.
86. Baez, Disnelys, Diego Morales, and Jorge A. Pino. 2012. “Volatiles from Michelia Champaca Flower: Comparative Analysis by Simultaneous Distillation-Extraction and Solid Phase Microextraction.” Natural product communications 7(5):659–60.
87. Bahadur, Shiv et al. 2016. “Metabolism-Mediated Interaction Potential of Standardized Extract of Tinospora Cordifolia through Rat and Human Liver Microsomes.” Indian journal of pharmacology 48(5):576–81.
88. Bai, Jinrong et al. 2015. “Antibacterial Activity of Shikimic Acid from Pine Needles of Cedrus Deodara against Staphylococcus Aureus through Damage to Cell Membrane.” International journal of molecular sciences 16(11):27145–55.
89. Bajpai, A. and J. K. Ojha. 2000. “Comparative Studies of Boerhaavia Diffusa L. And Boerhaavia Verticillata Poir. (Nyctaginaceae).” Ancient science of life 19(3–4):105–9.
90. Bala, Asis, Biswakanth Kar, Pallab K. Haldar, Upal K. Mazumder, and Samit Bera. 2010. “Evaluation of Anticancer Activity of Cleome Gynandra on Ehrlich’s Ascites Carcinoma Treated Mice.” Journal of ethnopharmacology 129(1):131–34.
91. Balakrishnan, Gayathri, Lakshmi Janakarajan, Arun Balakrishnan, and Baddireddi Subhadra Lakshmi. 2010. “Molecular Basis of the Anti-Inflammatory Property Exhibited by Cyclo-Pentano Phenanthrenol Isolated from Lippia Nodiflora.” Immunological investigations 39(7):713–39.
92. Balasubramanian, S., D. Ganesh, Poonam Panchal, Mohammad Teimouri, and V. V. S. Surya Narayana. 2014. “GC-MS Analysis of Phytocomponents in the Methanolic Extract of Emblica Officinalis Gaertn (Indian Gooseberry).” Journal of Chemical and Pharmaceutical Research 6(6):843–45.
93. Baliga, Manjeshwar Shrinath. 2012. “Review of the Phytochemical, Pharmacological and Toxicological Properties of Alstonia Scholaris Linn. R. Br (Saptaparna).” Chinese journal of integrative medicine.
94. Baliga, Manjeshwar Shrinath et al. 2013. “Ocimum Sanctum L (Holy Basil or Tulsi) and Its Phytochemicals in the Prevention and Treatment of Cancer.” Nutrition and cancer 65 Suppl 1:26–35.
95. Bandara, B. M., N. S. Kumar, and K. M. Samaranayake. 1989. “An Antifungal Constituent from the Stem Bark of Butea Monosperma.” Journal of ethnopharmacology 25(1):73–75.
96. BANERJEE, S. K., R. N. CHAKRAVARTI, and H. M. FALES. 1964. “LIRIODENINE FROM MICHELIA CHAMPACA.” Bulletin of the Calcutta School of Tropical Medicine 12:23–24.
97. Banihani, Saleem Ali. 2017. “Radish (Raphanus Sativus) and Diabetes.” Nutrients 9(9).
98. Baranowski, J. D. 1985. “High-Performance Liquid Chromatographic Separation of Pungency Components of Ginger.” Journal of Chromatography A 319(Supplement C):471–74.
99. Barkatullah, - et al. 2017. “Pharmacognostic and Phytochemical Studies of Zanthoxylum Armatum DC.” Pakistan journal of pharmaceutical sciences 30(2):429–38.
100. Barua, A. K. et al. 1976. “The Structure and Stereochemistry of Barrigenic Acid, a New Triterpene Acid Sapogenin from Barringtonia Acutangula.” Phytochemistry 15(11):1780–81. Retrieved (http://www.sciencedirect.com/science/article/pii/S0031942200974843).
101. Barua, A. K. and P. Chakrabarti. 1965. “Triterpenoids—XIX: The Constitution of Barringtogenol C—A New Triterpenoid Sapogenin from Barringtonia Acutangula Gaertn.” Tetrahedron 21(3):381–87. Retrieved (http://www.sciencedirect.com/science/article/pii/S0040402001982766).
102. Barua, A. K., S. P. Dutta, and B. C. Das. 1968. “Triterpenoids—XXIX: The Structure of Barringtogenol B—A New Triterpenoid Sapogenin from Barringtonia Acutangula Gaertn.” Tetrahedron 24(3):1113–17. Retrieved (http://www.sciencedirect.com/science/article/pii/0040402068880603).
103. BARUA, A. K., P. C. MAITI, and S. K. CHAKRABORTI. 1961. “Triterpenoids. XI. New Triterpenoid Sapogenins from the Fruits of Barringtonia Acutangula.” Journal of pharmaceutical sciences 50:937–40.
104. Bautista, Diana M. et al. 2005. “Pungent Products from Garlic Activate the Sensory Ion Channel TRPA1.” Proceedings of the National Academy of Sciences of the United States of America 102(34):12248–52.
105. Beghelli, Daniela et al. 2017. “Antioxidant and Ex Vivo Immune System Regulatory Properties of Boswellia Serrata Extracts.” Oxidative medicine and cellular longevity 2017:7468064.
106. Begum, A.Sajeli, Shweta Verma, Mahendra Sahai, Kathrin Schneider, and Roderich Sussmuth. 2009. “Hyoscyamal, a New Tetrahydrofurano Lignan from Hyoscyamus Niger Linn.” Natural product research 23(7):595–600.
107. Begum, Rayhana, Manjur Ali Sheliya, Showkat R. Mir, Ekta Singh, and Manju Sharma. 2017. “Inhibition of Proinflammatory Mediators by Coumaroyl Lupendioic Acid, a New Lupane-Type Triterpene from Careya Arborea, on Inflammation-Induced Animal Model.” Journal of ethnopharmacology 206:376–92.
108. Begum, Sajeli et al. 2010. “Study of Anti-Inflammatory, Analgesic and Antipyretic Activities of Seeds of Hyoscyamus Niger and Isolation of a New Coumarinolignan.” Fitoterapia 81(3):178–84.
109. Behrens, Maik, Ming Gu, Shengjie Fan, Cheng Huang, and Wolfgang Meyerhof. 2017. “Bitter Substances from Plants Used in Traditional Chinese Medicine Exert Biased Activation of Human Bitter Taste Receptors.” Chemical Biology & Drug Design n/a-n/a.
110. La Bella, Salvatore et al. 2015. “Composition and Variability of the Essential Oil of the Flowers of Lavandula Stoechas from Various Geographical Sources.” Natural product communications 10(11):2001–4.
111. Bendjeddou, D., K. Lalaoui, and D. Satta. 2003. “Immunostimulating Activity of the Hot Water-Soluble Polysaccharide Extracts of Anacyclus Pyrethrum, Alpinia Galanga and Citrullus Colocynthis.” Journal of ethnopharmacology 88(2–3):155–60.
112. Benso, Bruna et al. 2016. “Anti-Inflammatory, Anti-Osteoclastogenic and Antioxidant Effects of Malva Sylvestris Extract and Fractions: In Vitro and In Vivo Studies.” PloS one 11(9):e0162728.
113. Bhadoriya, Santosh Singh, Vijay Mishra, Sushil Raut, Aditya Ganeshpurkar, and Sunil K. Jain. 2012. “Anti-Inflammatory and Antinociceptive Activities of a Hydroethanolic Extract of Tamarindus Indica Leaves.” Scientia pharmaceutica 80(3):685–700.
114. Bhandari, Pamita, Neeraj Kumar, Bikram Singh, and Vijay K. Kaul. 2007. “Cucurbitacins from Bacopa Monnieri.” Phytochemistry 68(9):1248–54.
115. Bhandari, Pamita, Neeraj Kumar, Bikram Singh, and Vijay Kumar Kaul. 2006. “Bacosterol Glycoside, a New 13,14-Seco-Steroid Glycoside from Bacopa Monnieri.” Chemical & pharmaceutical bulletin 54(2):240–41.
116. Bhanwase, Anil Subhash and Kallanagouda Ramappa Alagawadi. 2016. “Antioxidant and Immunomodulatory Activity of Hydroalcoholic Extract and Its Fractions of Leaves of Ficus Benghalensis Linn.” Pharmacognosy research 8(1):50–55.
117. Bharathi, K., B. Pushpalatha, and C. M. Jain. 2014. “Clinical Evaluation of Herbal Compound Drugs in the Management of Leiomyoma Induced Menorrhagia.” Ayushdhara 1(1):38–42.
118. Bhargava, K. P., K. Kishor, M. C. Pant, and P. R. Saxena. 1965. “Identification of Tryptamine Derivatives in Ranunculus Sceleratus L.” British journal of pharmacology and chemotherapy 25(3):743–50.
119. Bharitkar, Yogesh P. et al. 2015. “New Flavonoid Glycosides and Other Chemical Constituents from Clerodendrum Phlomidis Leaves: Isolation and Characterisation.” Natural product research 29(19):1850–56.
120. Bhati, Rajesh, Anupama Singh, Vikas Anand Saharan, Veerma Ram, and Anil Bhandari. 2012. “Strychnos Nux-Vomica Seeds: Pharmacognostical Standardization, Extraction, and Antidiabetic Activity.” Journal of Ayurveda and integrative medicine 3(2):80–84.
121. BHATT, R. H., M. L. KHORANA, J. R. PATEL, B. B. GAITONDE, and M. S. KEKRE. 1958. “Pharmacological Studies of Saponins of the Fruits of Luffa Echinata Roxb. and Seeds of Trigonelia Foenum-Graecum Linn.” Indian journal of psychology 2(1):309–21.
122. Bhatt, Vinod, Sushila Sharma, Neeraj Kumar, Upendra Sharma, and Bikram Singh. 2017. “Simultaneous Quantification and Identification of Flavonoids, Lignans, Coumarin and Amides in Leaves of Zanthoxylum Armatum Using UPLC-DAD-ESI-QTOF-MS/MS.” Journal of pharmaceutical and biomedical analysis 132:46–55.
123. Bhattacharjee, Indranil et al. 2010. “N-Alkane Profile of Argemone Mexicana Leaves.” Zeitschrift fur Naturforschung. C, Journal of biosciences 65(9–10):533–36.
124. Bhattacharjee, Niloy et al. 2017. “Protocatechuic Acid, a Phenolic from Sansevieria Roxburghiana Leaves, Suppresses Diabetic Cardiomyopathy via Stimulating Glucose Metabolism, Ameliorating Oxidative Stress, and Inhibiting Inflammation.” Frontiers in pharmacology 8:251.
125. Bhattacharyya, Dipto, Ragini Sinha, Saptarshi Hazra, Riddhi Datta, and Sharmila Chattopadhyay. 2013. “De Novo Transcriptome Analysis Using 454 Pyrosequencing of the Himalayan Mayapple, Podophyllum Hexandrum.” BMC genomics 14:748.
126. Bheemasankara Rao, C., T. Namosiva Rao, and B. Muralikrishna. 1977. “Flavonoids from Blumea Lacera.” Planta medica 31(3):235–37.
127. Biala et al. 1998. “Strychnochrysine, a New Bisindole Alkaloid from the Roots of Strychnos Nux-vomica1.” Journal of natural products 61(1):139–41.
128. Bodakhe, Surendra H. and Alpana Ram. 2007. “Hepatoprotective Properties of Bauhinia Variegata Bark Extract.” Yakugaku zasshi : Journal of the Pharmaceutical Society of Japan 127(9):1503–7.
129. Bohlmann, Ferdinand and Pradip K. Mahanta. 1979. “Eine Neue Diterpensaure Aus Centipeda Orbicularis.” Phytochemistry 18(6):1067–68. Retrieved (http://www.sciencedirect.com/science/article/pii/S0031942200914857).
130. Boonen, Jente et al. 2012. “Alkamid Database: Chemistry, Occurrence and Functionality of Plant N-Alkylamides.” Journal of Ethnopharmacology 142(3):563–90.
131. Bottini, A. T. et al. 1987. “Sesquiterpene Alcohols from Hedychium Spicatum Var. Acuminatum.” Journal of natural products 50(4):732–34.
132. Bratati, D. and P. C. Datta. 1988. “Alkaloids of Strychnos Nux-Vomica Flower.” Planta medica 54(4):363.
133. Brieskorn, C. H. and W. Riedel. 1977. “[Triterpenic acids from Coleus amboinicus Loureiro (author’s transl)].” Archiv der Pharmazie 310(11):910–16.
134. Brockhoff, Anne, Maik Behrens, Alberto Massarotti, Giovanni Appending, and Wolfgang Meyerhof. 2007. “Broad Tuning of the Human Bitter Taste Receptor hTAS2R46 to Various Sesquiterpene Lactones, Clerodane and Labdane Diterpenoids, Strychnine, and Denatonium.” Journal of Agricultural and Food Chemistry 55(15):6236–43.
135. Bulle, Saradamma, Hymavathi Reddyvari, Varadacharyulu Nallanchakravarthula, and Damodara Reddy Vaddi. 2016. “Therapeutic Potential of Pterocarpus Santalinus L.: An Update.” Pharmacognosy reviews 10(19):43–49.
136. Bulugonda, Ramakrishna K. et al. 2017. “Mangiferin from Pueraria Tuberosa Reduces Inflammation via Inactivation of NLRP3 Inflammasome.” Scientific reports 7:42683.
137. Canini, Antonella, Daniela Alesiani, Giuseppe D’Arcangelo, and Pietro Tagliatesta. 2007. “Gas Chromatography–mass Spectrometry Analysis of Phenolic Compounds from Carica Papaya L. Leaf.” Journal of Food Composition and Analysis 20(7):584–90. Retrieved (http://www.sciencedirect.com/science/article/pii/S0889157507000610).
138. Cao, Kun et al. 2016. “A New Sesquiterpenoid from Saussurea Lappa Roots.” Natural product research 30(19):2160–63.
139. Cao, Shu-Yong, Zhong-Lu Ke, and Li-Min Xi. 2013. “A New Sesquiterpene Lactone from Salvia Plebeia.” Journal of Asian natural products research 15(4):404–7.
140. Caputo, Lucia, Luceia Fatima Souza, Susanna Alloisio, Laura Cornara, and Vincenzo De Feo. 2016. “Coriandrum Sativum and Lavandula Angustifolia Essential Oils: Chemical Composition and Activity on Central Nervous System.” International journal of molecular sciences 17(12).
141. Carvalho, Nathalia S. et al. 2015. “Gastroprotective Properties of Cashew Gum, a Complex Heteropolysaccharide of Anacardium Occidentale, in Naproxen-Induced Gastrointestinal Damage in Rats.” Drug development research 76(3):143–51.
142. Cazarolli, Luisa Helena et al. 2012. “Anti-Hyperglycemic Action of Apigenin-6-C-Beta-Fucopyranoside from Averrhoa Carambola.” Fitoterapia 83(7):1176–83.
143. Chai, Ling, Bu-Ming Liu, Xiao Lin, Qi-Xiu Li, and Mao-Xiang Lai. 2012. “[Analysis of compositions of the essential oil from Curcuma aromatica by gas chromatography-mass spectrometry].” Zhong yao cai = Zhongyaocai = Journal of Chinese medicinal materials 35(7):1102–4.
144. Chai, Tsun-Thai, Chee-Siong Khoo, Chong-Siang Tee, and Fai-Chu Wong. 2016. “Alpha-Glucosidase Inhibitory and Antioxidant Potential of Antidiabetic Herb Alternanthera Sessilis: Comparative Analyses of Leaf and Callus Solvent Fractions.” Pharmacognosy magazine 12(48):253–58.
145. Chakraborti, S. K. and A. K. Barua. 1963. “Triterpenoids—XVI: The Constitution of Barringtogenol D—A New Triterpenoid Sapogenin from Barringtonia Acutangula Gaertn.” Tetrahedron 19(11):1727–32. Retrieved (http://www.sciencedirect.com/science/article/pii/S0040402001992476).
146. Chakraborty, Raja, Biplab De, N. Devanna, and Saikat Sen. 2013. “Antitussive, Expectorant Activity of Marsilea Minuta L., an Indian Vegetable.” Journal of advanced pharmaceutical technology & research 4(1):61–64.
147. Chakravarty, M., C. Chaudhuri, B. Achari, and S. C. Pakrashi. 1988. “N-Acetylnornuciferine and Other Constituents of Aristolochia Bracteata.” Planta medica 54(5):467–68.
148. Chakthong, Suda et al. 2012. “Alkaloid and Coumarins from the Green Fruits of Aegle Marmelos.” Phytochemistry 75:108–13.
149. Chanda, Joydeb et al. 2014. “RP-HPLC Simultaneous Estimation of Betulinic Acid and Ursolic Acid in Carissa Spinarum.” Natural product research 28(21):1926–28.
150. Chandra, Mahesh et al. 2017. “Beta-Selinene-Rich Essential Oils from the Parts of Callicarpa Macrophylla and Their Antioxidant and Pharmacological Activities.” Medicines (Basel, Switzerland) 4(3).
151. Chandrashekhar, V. M., Ashok A. Muchandi, Sarasvathi V Sudi, and Seru Ganapty. 2010. “Hepatoprotective Activity of Stereospermum Suaveolens against CCl4-Induced Liver Damage in Albino Rats.” Pharmaceutical biology 48(5):524–28.
152. Chang, Yuh-Chwen, Pei-Wen Hsieh, et al. 2003. “Two New Protopines Argemexicaines A and B and the Anti-HIV Alkaloid 6-Acetonyldihydrochelerythrine from Formosan Argemone Mexicana.” Planta medica 69(2):148–52.
153. Chang, Yuh-Chwen, Fang-Rong Chang, Ashraf T. Khalil, Pei-Wen Hsieh, and Yang-Chang Wu. 2003. “Cytotoxic Benzophenanthridine and Benzylisoquinoline Alkaloids from Argemone Mexicana.” Zeitschrift fur Naturforschung. C, Journal of biosciences 58(7–8):521–26.
154. Chatterjee, Urmimala, Partha Pratim Bose, Sharmistha Dey, Tej P. Singh, and Bishnu P. Chatterjee. 2008. “Antiproliferative Effect of T/Tn Specific Artocarpus Lakoocha Agglutinin (ALA) on Human Leukemic Cells (Jurkat, U937, K562) and Their Imaging by QD-ALA Nanoconjugate.” Glycoconjugate journal 25(8):741–52.
155. Chaudhary, Anu, Anil Bhandari, and A. Pandurangan. 2012. “Antioxidant Potential and Total Phenolic Content of Methanolic Bark Extract of Madhuca Indica (Koenig) Gmelin.” Ancient science of life 31(3):132–36.
156. Chauhan, S. K., B. Singh, and S. Agrawal. 2000. “Simultaneous Determination of Bergenin and Gallic Acid in Bergenia Ligulata Wall by High-Performance Thin-Layer Chromatography.” Journal of AOAC International 83(6):1480–83.
157. Chaves, Otemberg Souza et al. 2013. “Secondary Metabolites from Sida Rhombifolia L. (Malvaceae) and the Vasorelaxant Activity of Cryptolepinone.” Molecules (Basel, Switzerland) 18(3):2769–77.
158. Chaves, Otemberg Souza et al. 2017. “Alkaloids and Phenolic Compounds from Sida Rhombifolia L. (Malvaceae) and Vasorelaxant Activity of Two Indoquinoline Alkaloids.” Molecules (Basel, Switzerland) 22(1).
159. Chawech, Rachid et al. 2015. “Cucurbitacins from the Leaves of Citrullus Colocynthis (L.) Schrad.” Molecules (Basel, Switzerland) 20(10):18001–15.
160. Che, C. T. et al. 1984. “Studies on Aristolochia III. Isolation and Biological Evaluation of Constituents of Aristolochia Indica Roots for Fertility-Regulating Activity.” Journal of natural products 47(2):331–41.
161. Cheenpracha, Sarot et al. 2005. “New Diterpenoids from Stems and Roots of Caesalpinia Crista.” Tetrahedron 61(36):8656–62. Retrieved (http://www.sciencedirect.com/science/article/pii/S004040200501149X).
162. Chen, Chao Jun et al. 2014. “Oligostilbenoids with Acetylcholinesterase Inhibitory Activity from Dipterocarpus Alatus.” Planta medica 80(17):1641–46.
163. Chen, Chien-chang et al. 2013. “Chemical Constituents and Anticancer Activity of Curcuma Zedoaria Roscoe Essential Oil against Non-Small Cell Lung Carcinoma Cells in Vitro and in Vivo.” Journal of agricultural and food chemistry 61(47):11418–27.
164. Chen, Hua-Dong, Sheng-Ping Yang, Yan Wu, Lei Dong, and Jian-Min Yue. 2009. “Terpenoids from Toona Ciliata.” Journal of natural products 72(4):685–89.
165. Chen, J. L. et al. 1998. “New Iridoids from the Medicinal Plant Barleria Prionitis with Potent Activity against Respiratory Syncytial Virus.” Journal of natural products 61(10):1295–97.
166. Chen, Jingjin, Andrew J. Ferreira, and Christopher M. Beaudry. 2014. “Synthesis of Bis(indole) Alkaloids from Arundo Donax: The Ynindole Diels-Alder Reaction, Conformational Chirality, and Absolute Stereochemistry.” Angewandte Chemie (International ed. in English) 53(44):11931–34.
167. Chen, Jun, Jian-Hua Wei, Shao-Fang Cai, Wei-Sheng Miao, and Li-Wei Pan. 2013. “[Study on chemical constituents of Cardiospermum halicacabum].” Zhong yao cai = Zhongyaocai = Journal of Chinese medicinal materials 36(2):228–30.
168. Chen, Xinglong et al. 2017. “New Phenolic Glycosides from Curculigo Orchioides and Their Xanthine Oxidase Inhibitory Activities.” Fitoterapia 122:144–49.
169. Chen, Yu-Ping et al. 2008. “Chemical Constituents from Sappan Lignum.” Journal of Chinese Pharmaceutical Science 17:82–86.
170. Chen, Yu-Sheng, Chao-Jun Chen, Wei Yan, Hui-Ming Ge, and Ling-Dong Kong. 2017. “Anti-Hyperuricemic and Anti-Inflammatory Actions of Vaticaffinol Isolated from Dipterocarpus Alatus in Hyperuricemic Mice.” Chinese journal of natural medicines 15(5):330–40.
171. Cheriyan, Binoy Varghese Sr et al. 2017. “Anti-Nociceptive Effect of 7-Methoxy Coumarin from Eupatorium Triplinerve Vahl (Asteraceae).” Pharmacognosy magazine 13(49):81–84.
172. Chialva, F. and G. Dada. 1990. “Bitterness in Alcoholic Beverages.” Pp. 103–22 in Bitternes in foods and beverages. Developments in Food Science vol. 25, edited by R. L. Rouseff. Elsevier Science Publishers B.V.
173. Chiruvella, Kishore K., Arifullah Mohammed, Gayathri Dampuri, Rama Gopal Ghanta, and Sathees C. Raghavan. 2007. “Phytochemical and Antimicrobial Studies of Methyl Angolensate and Luteolin-7-O-Glucoside Isolated from Callus Cultures of Soymida Febrifuga.” International journal of biomedical science : IJBS 3(4):269–78.
174. Chitra, M. and J. E. Thoppil. 2002. “Pharmacognostical and Phytochemical Investifations on the Tuberous Roots of Hemidesmus Indicus (Linn.) R.br. (Asclepiadaceae).” Ancient science of life 21(4):248–55.
175. Chiu, Sharon, Thomas Wang, Martin Belski, and Ehab A. Abourashed. 2016. “HPLC-Guided Isolation, Purification and Characterization of Phenylpropanoid and Phenolic Constituents of Nutmeg Kernel (Myristica Fragrans).” Natural product communications 11(4):483–88.
176. Cho, J. Y. et al. 2001. “Savinin, a Lignan from Pterocarpus Santalinus Inhibits Tumor Necrosis Factor-Alpha Production and T Cell Proliferation.” Biological & pharmaceutical bulletin 24(2):167–71.
177. Chokchaisiri, Ratchanaporn et al. 2009. “Bioactive Flavonoids of the Flowers of Butea Monosperma.” Chemical & pharmaceutical bulletin 57(4):428–32.
178. Chothani, Daya L. and H. U. Vaghasiya. 2011. “A Review on Balanites Aegyptiaca Del (Desert Date): Phytochemical Constituents, Traditional Uses, and Pharmacological Activity.” Pharmacognosy reviews 5(9):55–62.
179. Choudhury, Bhaswati et al. 2016. “Anticancer Activity of Garcinia Morella on T-Cell Murine Lymphoma Via Apoptotic Induction.” Frontiers in pharmacology 7:3.
180. Chowdhury, R., R. B. Rashid, M. H. Sohrab, and C. M. Hasan. 2003. “12alpha-Hydroxystigmast-4-En-3-One: A New Bioactive Steroid from Toona Ciliata (Meliaceae).” Die Pharmazie 58(4):272–73.
181. Cirlini, Martina et al. 2016. “Phenolic and Volatile Composition of a Dry Spearmint (Mentha Spicata L.) Extract.” Molecules (Basel, Switzerland) 21(8).
182. Claeson, U. P., T. Malmfors, G. Wikman, and J. G. Bruhn. 2000. “Adhatoda Vasica: A Critical Review of Ethnopharmacological and Toxicological Data.” Journal of ethnopharmacology 72(1–2):1–20.
183. Colegate, Steven M., Dale R. Gardner, Robert J. Joy, Joseph M. Betz, and Kip E. Panter. 2012. “Dehydropyrrolizidine Alkaloids, Including Monoesters with an Unusual Esterifying Acid, from Cultivated Crotalaria Juncea (Sunn Hemp cv.’Tropic Sun’).” Journal of agricultural and food chemistry 60(14):3541–50.
184. Correa-Ferreira, Marilia Locatelli, Guilhermina Rodrigues Noleto, and Carmen Lucia Oliveira Petkowicz. 2014. “Artemisia Absinthium and Artemisia Vulgaris: A Comparative Study of Infusion Polysaccharides.” Carbohydrate polymers 102:738–45.
185. Crestini, Claudia, Heiko Lange, and Giulia Bianchetti. 2016. “Detailed Chemical Composition of Condensed Tannins via Quantitative (31)P NMR and HSQC Analyses: Acacia Catechu, Schinopsis Balansae, and Acacia Mearnsii.” Journal of natural products 79(9):2287–95.
186. Cui, Baolu et al. 2016. “Anthocyanins and Flavonols Are Responsible for Purple Color of Lablab Purpureus (L.) Sweet Pods.” Plant physiology and biochemistry : PPB 103:183–90.
187. Cuong, To Dao et al. 2012. “Phenolic Compounds from Caesalpinia Sappan Heartwood and Their Anti-Inflammatory Activity.” Journal of natural products 75(12):2069–75.
188. Dai, Yiqun et al. 2014. “Four New Eudesmane-Type Sesquiterpenes from the Basal Leaves of Salvia Plebeia R. Br.” Fitoterapia 94:142–47.
189. Dantas, Andrea Dos Santos et al. 2016. “Origanum Majorana Essential Oil Lacks Mutagenic Activity in the Salmonella/Microsome and Micronucleus Assays.” TheScientificWorldJournal 2016:3694901.
190. Das, Debsankar et al. 2009. “Isolation and Characterization of a Heteropolysaccharide from the Corm of Amorphophallus Campanulatus.” Carbohydrate research 344(18):2581–85.
191. Das, P. C. et al. 1999. “Cleogynol, a Novel Dammarane Triterpenoid from Cleome Gynandra.” Journal of natural products 62(4):616–18.
192. Das, Sarita and S.Niranjali Devaraj. 2006. “Glycosides Derived from Hemidesmus Indicus R. Br. Root Inhibit Adherence of Salmonella Typhimurium to Host Cells: Receptor Mimicry.” Phytotherapy research : PTR 20(9):784–93.
193. Dasgupta, B. and K. Basu. 1970. “Chemical Investigation of Abroma Augusta Linn. Identity of Abromine with Betaine.” Experientia 26(5):477–78.
194. Debela, Etana, Adugna Tolera, Lars Olav Eik, and Ragnar Salte. 2012. “Condensed Tannins from Sesbania Sesban and Desmodium Intortum as a Means of Haemonchus Contortus Control in Goats.” Tropical animal health and production 44(8):1939–44.
195. Debnath, Monojit, Moulisha Biswas, Vinay J. Shukla, and K. Nishteswar. 2014. “Phytochemical and Analytical Evaluation of Jyotishmati (Celastrus Paniculatus Willd.) Leaf Extracts.” Ayu 35(1):54–57.
196. Deepak, M., G. K. Sangli, P. C. Arun, and A. Amit. 2005. “Quantitative Determination of the Major Saponin Mixture Bacoside A in Bacopa Monnieri by HPLC.” Phytochemical analysis : PCA 16(1):24–29.
197. Deevanhxay, Phengxay et al. 2009. “Simultaneous Characterization of Quaternary Alkaloids, 8-Oxoprotoberberine Alkaloids, and a Steroid Compound in Coscinium Fenestratum by Liquid Chromatography Hybrid Ion Trap Time-of-Flight Mass Spectrometry.” Journal of pharmaceutical and biomedical analysis 50(3):413–25.
198. Delaviz, Hamdollah, Jamshid Mohammadi, Ghasem Ghalamfarsa, Bahram Mohammadi, and Naser Farhadi. 2017. “A Review Study on Phytochemistry and Pharmacology Applications of Juglans Regia Plant.” Pharmacognosy reviews 11(22):145–52.
199. Deng, Yanshen and Russell A. Nicholson. 2005. “Antifungal Properties of Surangin B, a Coumarin from Mammea Longifolia.” Planta medica 71(4):364–65.
200. Desai, Dattatraya C., Jeenu Jacob, Asha Almeida, Rajendra Kshirsagar, and S. L. Manju. 2014. “Isolation, Structural Elucidation and Anti-Inflammatory Activity of Astragalin, (-)Hinokinin, Aristolactam I and Aristolochic Acids (I & II) from Aristolochia Indica.” Natural product research 28(17):1413–17.
201. Dewanjee, Saikat, Anup Maiti, Ranabir Sahu, Tarun K. Dua, and Vivekananda Mandal. 2011. “Effective Control of Type 2 Diabetes through Antioxidant Defense by Edible Fruits of Diospyros Peregrina.” Evidence-based complementary and alternative medicine : eCAM 2011:675397.
202. Dey, Priyankar and Tapas Kumar Chaudhuri. 2014. “Pharmacological Aspects of Nerium Indicum Mill: A Comprehensive Review.” Pharmacognosy reviews 8(16):156–62.
203. Di, X. et al. 2013. “New Phenolic Compounds from the Twigs of Artocarpus Heterophyllus.” Drug discoveries & therapeutics 7(1):24–28.
204. Dikti Vildina, Jacqueline et al. 2017. “Anti-Onchocerca and Anti-Caenorhabditis Activity of a Hydro-Alcoholic Extract from the Fruits of Acacia Nilotica and Some Proanthocyanidin Derivatives.” Molecules (Basel, Switzerland) 22(5).
205. Ding, Wenbing et al. 2011. “Bioactive Dammarane-Type Saponins from Operculina Turpethum.” Journal of natural products 74(9):1868–74.
206. Ding, Wenbing, Zi-Hua Jiang, Ping Wu, Liangxiong Xu, and Xiaoyi Wei. 2012. “Resin Glycosides from the Aerial Parts of Operculina Turpethum.” Phytochemistry 81:165–74.
207. Dixit, Preety, Mohd Parvez Khan, Gaurav Swarnkar, N. Chattopadhyay, and Rakesh Maurya. 2011. “Osteogenic Constituents from Pterospermum Acerifolium Willd. Flowers.” Bioorganic & medicinal chemistry letters 21(15):4617–21.
208. Dokka, Muni Kumar, Lavanya Seva, and Siva Prasad Davuluri. 2015. “Isolation and Purification of Trypsin Inhibitors from the Seeds of Abelmoschus Moschatus L.” Applied biochemistry and biotechnology 175(8):3750–62.
209. Dong, Cai-Xia, Kyoko Hayashi, Yusuke Mizukoshi, Jung-Bum Lee, and Toshimitsu Hayashi. 2012. “Structures and Anti-HSV-2 Activities of Neutral Polysaccharides from an Edible Plant, Basella Rubra L.” International journal of biological macromolecules 50(1):245–49.
210. Dong, Fa-Wu et al. 2015. “Iridoids and Sesquiterpenoids from the Roots of Valeriana Jatamansi Jones.” Fitoterapia 102:27–34.
211. Dorsaz, A. C., M. Hostettmann, and K. Hostettmann. 1988. “Molluscicidal Saponins from Sesbania Sesban.” Planta medica 54(3):225–27.
212. Doshi, Gaurav M. et al. 2015. “Structural Elucidation of Chemical Constituents from Benincasa Hispida Seeds and Carissa Congesta Roots by Gas Chromatography: Mass Spectroscopy.” Pharmacognosy research 7(3):282–93.
213. Doshi, Gaurav Mahesh and Hemant Devidas Une. 2016. “Quantification of Quercetin and Rutin from Benincasa Hispida Seeds and Carissa Congesta Roots by High-Performance Thin Layer Chromatography and High-Performance Liquid Chromatography.” Pharmacognosy research 8(1):37–42.
214. Doshi, Krunal A., Rabinarayan Acharya, V. J. Shukla, Renuka Kalyani, and Komal Khanpara. 2013. “Phytochemical Evaluation of the Wild and Cultivated Varieties of Eranda Mula (Roots of Ricinus Communis Linn.).” Ayu 34(2):200–203.
215. Drewnowski, Adam and Carmen Gomez-carneros. 2000. “Bitter Taste , Phytonutrients , and the Consumer : A Review 1 – 3.” (22):1424–35.
216. Du, Zhizhi, Robin A. Clery, and Christopher J. Hammond. 2008. “Volatile Organic Nitrogen-Containing Constituents in Ambrette Seed Abelmoschus Moschatus Medik (Malvaceae).” Journal of agricultural and food chemistry 56(16):7388–92.
217. Dubey, Nidhi, Nitin Dubey, Rajendra Mehta, and Ajay Saluja. 2009. “Estimation of Catechin in Ayurvedic Oil Formulations Containing Acacia Catechu.” Journal of AOAC International 92(4):1021–26.
218. Dubois, M. A., S. Benze, and H. Wagner. 1990. “New Biologically Active Triterpene-Saponins from Randia Dumetorum.” Planta medica 56(5):451–55.
219. Duraipandiyan, Veeramuthu, Naif Abdullah Al-Harbi, Savarimuthu Ignacimuthu, and Chinnasamy Muthukumar. 2012. “Antimicrobial Activity of Sesquiterpene Lactones Isolated from Traditional Medicinal Plant, Costus Speciosus (Koen ex.Retz.) Sm.” BMC complementary and alternative medicine 12:13.
220. Duval, Antoine and Luc Averous. 2016. “Characterization and Physicochemical Properties of Condensed Tannins from Acacia Catechu.” Journal of agricultural and food chemistry 64(8):1751–60.
221. El-Aasr, Mona et al. 2009. “A New Spirostanol Glycoside from Fruits of Solanum Indicum L.” Chemical & pharmaceutical bulletin 57(7):747–48.
222. El-Gamal, Ali Ali et al. 2015. “A Novel Beta-Lactam Derivative, Albactam from the Flowers of Albizia Lebbeck with Platelets Anti-Aggregatory Activity in Vitro.” Pakistan journal of pharmaceutical sciences 28(2 Suppl):745–53.
223. El-Hawary, Seham A., Nadia M. Sokkar, Zeinab Y. Ali, and Marwa M. Yehia. 2011. “A Profile of Bioactive Compounds of Rumex Vesicarius L.” Journal of food science 76(8):C1195-202.
224. El-Hawary, Z. M. and T. S. Kholief. 1990. “Biochemical Studies on Hypoglycemic Agents (I) Effect ofAzadirachta Indica Leaf Extract.” Archives of Pharmacal Research 13(1):108–12.
225. el-Mousallamy, A. M. 1998. “Leaf Flavonoids of Albizia Lebbeck.” Phytochemistry 48(4):759–61.
226. el-Sayed, N. H. 1991. “A Rare Kaempferol Trisaccharide Anti-Tumor Promotor from Sesbania Sesban.” Die Pharmazie 46(9):679–80.
227. El-Shazly, Assem, Afaf El-Sayed, and Eman Fikrey. 2012. “Bioactive Secondary Metabolites from Salix Tetrasperma Roxb.” Zeitschrift fur Naturforschung. C, Journal of biosciences 67(7–8):353–59.
228. Eldahshan, Omayma A. and Mohamed M. Abdel-Daim. 2015. “Phytochemical Study, Cytotoxic, Analgesic, Antipyretic and Anti-Inflammatory Activities of Strychnos Nux-Vomica.” Cytotechnology 67(5):831–44.
229. Elfahmi et al. 2007. “Lignan Profile of Piper Cubeba, an Indonesian Medicinal Plant.” Biochemical Systematics and Ecology 35(7):397–402. Retrieved (http://www.sciencedirect.com/science/article/pii/S0305197807000063).
230. Erenler, Ramazan et al. 2016. “Isolation and Identification of Chemical Constituents from Origanum Majorana and Investigation of Antiproliferative and Antioxidant Activities.” Journal of the science of food and agriculture 96(3):822–36.
231. Esimone, Charles O. et al. 2007. “In Vitro Evaluation of the Antiviral Activity of Extracts from the Lichen Parmelia Perlata (L.) Ach. against Three RNA Viruses.” Journal of infection in developing countries 1(3):315–20.
232. Faiz Hossain, Chowdhury et al. 2015. “Analgesic Principle from Curcuma Amada.” Journal of ethnopharmacology 163:273–77.
233. Faizi, S. and M. Ali. 1999. “Shamimin: A New Flavonol C-Glycoside from Leaves of Bombax Ceiba.” Planta medica 65(4):383–85.
234. Faizi, Shaheen, Sadia Zikr-Ur-Rehman, and Muhammad Ali Versiani. 2011. “Shamiminol: A New Aromatic Glycoside from the Stem Bark of Bombax Ceiba.” Natural product communications 6(12):1897–1900.
235. Fakhoury, A. M. and C. P. Woloshuk. 2001. “Inhibition of Growth of Aspergillus Flavus and Fungal Alpha-Amylases by a Lectin-like Protein from Lablab Purpureus.” Molecular plant-microbe interactions : MPMI 14(8):955–61.
236. Fan, Qing-Lu, Yin-Di Zhu, Wen-Hua Huang, Yun Qi, and Bao-Lin Guo. 2014. “Two New Acylated Flavonol Glycosides from the Seeds of Lepidium Sativum.” Molecules (Basel, Switzerland) 19(8):11341–49.
237. Farag, Mohamed A. et al. 2017. “Phytochemical Profiles and Antimicrobial Activities of Allium Cepa Red Cv. and A. Sativum Subjected to Different Drying Methods: A Comparative MS-Based Metabolomics.” Molecules (Basel, Switzerland) 22(5).
238. Farag, S. F., A. S. Ahmed, K. Terashima, Y. Takaya, and M. Niwa. 2001. “Isoflavonoid Glycosides from Dalbergia Sissoo.” Phytochemistry 57(8):1263–68.
239. Feng, Liang et al. 2013. “A Combination of Alkaloids and Triterpenes of Alstonia Scholaris (Linn.) R. Br. Leaves Enhances Immunomodulatory Activity in C57BL/6 Mice and Induces Apoptosis in the A549 Cell Line.” Molecules (Basel, Switzerland) 18(11):13920–39.
240. Fenwick, G. R., C. L. Curl, N. M. Griffiths, R. K. Heaney, and K. R. Price. 1990. “Bitter Principles in Food Plants.” Pp. 205–50 in Bitternes in foods and beverages. Developments in Food Science vol. 25, edited by R. L. Rouseff. Amsterdam: Elsevier Science Publishers B.V.
241. Fenwick, G. R., J. Lutomski, and C. Nieman. 1990. “Liquorice, Glycyrrhiza Glabra L.—Composition, Uses and Analysis.” Food Chemistry 38(2):119–43. Retrieved (http://www.sciencedirect.com/science/article/pii/0308814690901592).
242. Ferreira, Raquel Teixeira et al. 2014. “Mechanisms Underlying the Antinociceptive, Antiedematogenic, and Anti-Inflammatory Activity of the Main Flavonoid from Kalanchoe Pinnata.” Evidence-based complementary and alternative medicine : eCAM 2014:429256.
243. Fiamegos, Yiannis C. et al. 2011. “Antimicrobial and Efflux Pump Inhibitory Activity of Caffeoylquinic Acids from Artemisia Absinthium against Gram-Positive Pathogenic Bacteria.” PloS one 6(4):e18127.
244. Forster, Yvonne, Abdul Ghaffar, and Stefan Bienz. 2016. “A New View on the Codonocarpine Type Alkaloids of Capparis Decidua.” Phytochemistry 128:50–59.
245. Frankish, Neil, Fabio de Sousa Menezes, Clive Mills, and Helen Sheridan. 2010. “Enhancement of Insulin Release from the Beta-Cell Line INS-1 by an Ethanolic Extract of Bauhinia Variegata and Its Major Constituent Roseoside.” Planta medica 76(10):995–97.
246. Franzotti, E. M. et al. 2000. “Anti-Inflammatory, Analgesic Activity and Acute Toxicity of Sida Cordifolia L. (Malva-Branca).” Journal of ethnopharmacology 72(1–2):273–77.
247. Fraternale, Daniele, Guido Flamini, and Donata Ricci. 2014. “Essential Oil Composition and Antimicrobial Activity of Angelica Archangelica L. (Apiaceae) Roots.” Journal of medicinal food 17(9):1043–47.
248. Frerot, Eric, Nathalie Neirynck, Isabelle Cayeux, Yoyo Hui-Juan Yuan, and Yong-Ming Yuan. 2015. “New Umami Amides: Structure–Taste Relationship Studies of Cinnamic Acid Derived Amides and the Natural Occurrence of an Intense Umami Amide in Zanthoxylum Piperitum.” Journal of Agricultural and Food Chemistry 63(32):7161–68.
249. Fu, Lin-chun et al. 2008. “A New 3-Benzylchroman Derivative from Sappan Lignum (Caesalpinia Sappan).” Molecules (Basel, Switzerland) 13(8):1923–30.
250. Fu, Meihong et al. 2010. “[Determination of 5-hydroxy-7-methoxyflavan in Daemonorops draco by HPLC].” Zhongguo Zhong yao za zhi = Zhongguo zhongyao zazhi = China journal of Chinese materia medica 35(23):3192–93.
251. Fugmann, Furkhard, Susanne Lang-Fugmann, and Wolfgang Steglich. 2000. Encyclopedia of Natural Products. 1st ed. Georg Thieme Verlag.
252. Fukushima, S., M. Kuroyanagi, Y. Akahori, Y. Saiki, and A. Ueno. 1968. “[Structure of zedoarone, a new sesquiterpene from Curcuma zedoaria].” Yakugaku zasshi : Journal of the Pharmaceutical Society of Japan 88(6):792–94.
253. Fukushima, S., M. Kuroyanagi, A. Ueno, Y. Akahori, and Y. Saiki. 1970. “[Structure of curzerenone, a new sesquiterpene from Curcuma zedoaria].” Yakugaku zasshi : Journal of the Pharmaceutical Society of Japan 90(7):863–69.
254. Fursule, R. A. and S. D. Patil. 2010. “Hepatoprotective and Antioxidant Activity of Phaseolus Trilobus, Ait on Bile Duct Ligation Induced Liver Fibrosis in Rats.” Journal of ethnopharmacology 129(3):416–19.
255. Gabriele, Bartolo, Alessia Fazio, Paola Dugo, Rosaria Costa, and Luigi Mondello. 2009. “Essential Oil Composition of Citrus Medica L. Cv. Diamante (Diamante Citron) Determined after Using Different Extraction Methods.” Journal of separation science 32(1):99–108.
256. Gabrieli, C. and E. Kokkalou. 2003. “A New Acetylated Glucoside of Luteolin and Two Flavone Glucosides from Lavandula Stoechas Ssp. Stoechas.” Die Pharmazie 58(6):426–27.
257. Gacche, R. N., R. U. Shaikh, S. M. Chapole, A. D. Jadhav, and S. G. Jadhav. 2011. “Kinetics of Inhibition of Monoamine Oxidase Using Cymbopogon Martinii (Roxb.) Wats.: A Potential Antidepressant Herbal Ingredient with Antioxidant Activity.” Indian journal of clinical biochemistry : IJCB 26(3):303–8.
258. Gadgoli, Chhaya and Sandeep Shelke. 2010. “Crocetin from the Tubular Calyx of Nyctanthes Arbor-Tristis.” Natural product research 24(17):1610–15.
259. Gagandeep, Meera, and S. B. Kalidhar. 2009. “Chemical Investigation of Crataeva Nurvala Buch. Ham. Fruits.” Indian journal of pharmaceutical sciences 71(2):129–30.
260. Galani, V. J., B. G. Patel, and N. B. Patel. 2010. “Argyreia Speciosa (Linn. F.) Sweet: A Comprehensive Review.” Pharmacognosy reviews 4(8):172–78.
261. Gandhe, Sreekanth et al. 2013. “Cycloartanes from the Gum Resin of Gardenia Gummifera L.f.” Chemistry & biodiversity 10(9):1613–22.
262. Ganesh, Mani and Murugan Mohankumar. 2017. “Extraction and Identification of Bioactive Components in Sida Cordata (Burm.f.) Using Gas Chromatography-Mass Spectrometry.” Journal of food science and technology 54(10):3082–91.
263. Gangwar, Mayank, R. K. Goel, and Gopal Nath. 2014. “Mallotus Philippinensis Muell. Arg (Euphorbiaceae): Ethnopharmacology and Phytochemistry Review.” BioMed research international 2014:213973.
264. Ganie, Showkat Ahmad and Surender Singh Yadav. 2014. “Holoptelea Integrifolia (Roxb.) Planch: A Review of Its Ethnobotany, Pharmacology, and Phytochemistry.” BioMed research international 2014:401213.
265. Ganjewala, Deepak and Ashish Kumar Gupta. 2013. “Study on Phytochemical Composition, Antibacterial and Antioxidant Properties of Different Parts of Alstonia Scholaris Linn.” Advanced pharmaceutical bulletin 3(2):379–84.
266. Gao, Wenyan et al. 2015. “Cycloartan-24-Ene-1alpha,2alpha,3beta-Triol, a Cycloartane-Type Triterpenoid from the Resinous Exudates of Commiphora Myrrha, Induces Apoptosis in Human Prostatic Cancer PC-3 Cells.” Oncology reports 33(3):1107–14.
267. Gao, Xiao-zhong, Chang-xin Zhou, Shui-li Zhang, Wei Yao, and Yu Zhao. 2005. “[Studies on the chemical constituents in herb of Ranunculus sceleratus].” Zhongguo Zhong yao za zhi = Zhongguo zhongyao zazhi = China journal of Chinese materia medica 30(2):124–26.
268. Garaniya, Narendra and Atul Bapodra. 2014. “Ethno Botanical and Phytophrmacological Potential of Abrus Precatorius L.: A Review.” Asian Pacific journal of tropical biomedicine 4(Suppl 1):S27-34.
269. Garg, S. C. and S. L. Dengre. 1986. “Antibacterial Activity of Essential Oil of Tagetes Erecta Linn.” Hindustan antibiotics bulletin 28(1–4):27–29.
270. Garzoli, Stefania et al. 2017. “Essential Oil Extraction, Chemical Analysis and Anti-Candida Activity of Foeniculum Vulgare Miller - New Approaches.” Natural product research 1–6.
271. Gasparetto, Joao Cleverson, Cleverson Antonio Ferreira Martins, Sirlei Sayomi Hayashi, Michel Fleith Otuky, and Roberto Pontarolo. 2012. “Ethnobotanical and Scientific Aspects of Malva Sylvestris L.: A Millennial Herbal Medicine.” The Journal of pharmacy and pharmacology 64(2):172–89.
272. Gbedema, Stephen Y., Marcel T. Bayor, Kofi Annan, and Colin W. Wright. 2015. “Clerodane Diterpenes from Polyalthia Longifolia (Sonn) Thw. Var. Pendula: Potential Antimalarial Agents for Drug Resistant Plasmodium Falciparum Infection.” Journal of ethnopharmacology 169:176–82.
273. Ge, Jun-Jun et al. 2016. “Two New Tetracyclic Triterpenoids from the Barks of Melia Azedarach.” Journal of Asian natural products research 18(1):20–25.
274. Gegg, C. V and M. E. Etzler. 1994. “Photoaffinity Labeling of the Adenine Binding Sites of Two Dolichos Biflorus Lectins.” The Journal of biological chemistry 269(8):5687–92.
275. Germano, Antonio, Andrea Occhipinti, Francesca Barbero, and Massimo E. Maffei. 2017. “A Pilot Study on Bioactive Constituents and Analgesic Effects of MyrLiq(R), a Commiphora Myrrha Extract with a High Furanodiene Content.” BioMed research international 2017:3804356.
276. Ghannadi, Alireza, Ahmad Movahedian, and Zahra Jannesary. 2015. “Hypocholesterolemic Effects of Balangu (Lallemantia Royleana) Seeds in the Rabbits Fed on a Cholesterol-Containing Diet.” Avicenna journal of phytomedicine 5(3):167–73.
277. Ghildiyal, Shivani, Manish K. Gautam, Vinod K. Joshi, and Raj K. Goel. 2012. “Pharmacological Evaluation of Extracts of Hedychium Spicatum (Ham-Ex-Smith) Rhizome.” Ancient science of life 31(3):117–22.
278. Ghosal, S. and D. K. Jaiswal. 1980. “Chemical Constituents of Gentianaceae XXVIII: Flavonoids of Enicostemma Hyssopifolium (Willd.) Verd.” Journal of pharmaceutical sciences 69(1):53–56.
279. Ghosal, S., A. K. Singh, P. V Sharma, and R. K. Chaudhuri. 1974. “Chemical Constituents of Gentianaceae. IX. Natural Occurrence of Erythrocentaurin in Enicostemma Hyssopifolium and Swertia Lawii.” Journal of pharmaceutical sciences 63(6):944–45.
280. Ghosal, Shibnath, Rama Ballav P. S. Chauhan, and Rakesh Mehta. 1975. “Alkaloids of Sida Cordifolia.” Phytochemistry 14(3):830–32. Retrieved (http://www.sciencedirect.com/science/article/pii/0031942275830573).
281. Ghosh, Ashoke K., Debanjan Sen, and Sanjib Bhattacharya. 2010. “A New Alkaloid Isolated from Abies Webbiana Leaf.” Pharmacognosy research 2(3):186–89.
282. Ghosh, P. et al. 2011. “Triterpenoids from Schleichera Oleosa of Darjeeling Foothills and Their Antimicrobial Activity.” Indian journal of pharmaceutical sciences 73(2):231–33.
283. Ghosh, Santosh K. et al. 2007. “Characterization and Cloning of a Stearoyl/oleoyl Specific Fatty Acyl-Acyl Carrier Protein Thioesterase from the Seeds of Madhuca Longifolia (Latifolia).” Plant physiology and biochemistry : PPB 45(12):887–97.
284. Ghosh, Satyabrata, Runu Chakraborty, and Utpal Raychaudhuri. 2015. “Determination of pH-Dependent Antioxidant Activity of Palm (Borassus Flabellifer) Polyphenol Compounds by Photoluminol and DPPH Methods: A Comparison of Redox Reaction Sensitivity.” 3 Biotech 5(5):633–40.
285. Ghule, B. V and P. G. Yeole. 2012. “In Vitro and in Vivo Immunomodulatory Activities of Iridoids Fraction from Barleria Prionitis Linn.” Journal of ethnopharmacology 141(1):424–31.
286. Gil, R. R. et al. 1995. “Anacardoside from the Seeds of Semecarpus Anacardium.” Phytochemistry 39(2):405–7.
287. Gilani, Anwarul Hassan et al. 2010. “Pharmacological Basis for the Medicinal Use of Holarrhena Antidysenterica in Gut Motility Disorders.” Pharmaceutical biology 48(11):1240–46.
288. Giri, S., C. R. Lokesh, S. Sahu, and N. Gupta. 2014. “Luffa Echinata: Healer Plant or Potential Killer.” Journal of postgraduate medicine 60(1):72–74.
289. Gomez, R., F. M. Goni, and J. M. Macarulla. 1978. “Carotenoids from Marigold (Tagetes Erecta) Petals and Their Esterified Fatty Acids.” Revista espanola de fisiologia 34(3):253–56.
290. Gong, Xiao-Mei et al. 2013. “[Study on chemical constituents of Citrullus vulgaris Schrad vine (II)].” Zhong yao cai = Zhongyaocai = Journal of Chinese medicinal materials 36(10):1614–16.
291. Gopalakrishnan, G., N. D. Pradeep Singh, V. Kasinath, R. Malathi, and S. S. Rajan. 2000. “Photooxidation of Cedrelone, a Tetranortriterpenoid from Toona Ciliata.” Photochemistry and photobiology 72(4):464–66.
292. Goren, Ahmet et al. 2002. “The Chemical Constituents and Biological Activity of Essential Oil of Lavandula Stoechas Ssp. Stoechas.” Zeitschrift fur Naturforschung. C, Journal of biosciences 57(9–10):797–800.
293. Gosetti, Fabio et al. 2016. “Characterization of the Volatile and Nonvolatile Fractions of Heartwood Aqueous Extract from Pterocarpus Marsupium and Evaluation of Its Cytotoxicity against Cancer Cell Lines.” Planta medica 82(14):1295–1301.
294. Gottumukkala, Venkateswara Rao, Tiruganasambandham Annamalai, and Triptikumar Mukhopadhyay. 2011. “Phytochemical Investigation and Hair Growth Studies on the Rhizomes of Nardostachys Jatamansi DC.” Pharmacognosy magazine 7(26):146–50.
295. Gougeon, Rejeanne et al. 2005. “Increase in the Thermic Effect of Food in Women by Adrenergic Amines Extracted from Citrus Aurantium.” Obesity research 13(7):1187–94.
296. Goyal, Manoj, Anil Pareek, B. P. Nagori, and D. Sasmal. 2011. “Aerva Lanata: A Review on Phytochemistry and Pharmacological Aspects.” Pharmacognosy reviews 5(10):195–98.
297. Gross, Jeana, Raphael Ikan, and Gert Eckhardt. 1983. “Carotenoids of the Fruit of Averrhoa Carambola.” Phytochemistry 22(6):1479–81. Retrieved (http://www.sciencedirect.com/science/article/pii/S0031942200840406).
298. Gubbannavar, Jyoti S., H. M. Chandola, C. R. Harisha, Komal Khanpara, and V. J. Shukla. 2013. “A Comparative Pharmacognostical and Preliminary Physico-Chemical Analysis of Stem and Leaf of Bacopa Monnieri (L.) Pennel and Bacopa Floribunda (R.BR.) Wettst.” Ayu 34(1):95–102.
299. Guichard, Elisabeth, Christian Salles, Martine Morzel, and Anne-Marie Le Bon, eds. 2017. Flavour From Food to Perception. Chichester, West SussexHoboken, NJ: John Wiley & Sons, Ltd.
300. Guo, Shanshan et al. 2016. “The Chemical Composition of Essential Oils from Cinnamomum Camphora and Their Insecticidal Activity against the Stored Product Pests.” International journal of molecular sciences 17(11).
301. Guo, Tao, Xiao-Feng Tang, Jun Chang, and Ya Wang. 2017. “A New Lignan Glycoside from the Stems of Zanthoxylum Armatum DC.” Natural product research 31(1):16–21.
302. Gupta, Shweta and Akash Ved. 2017. “Operculina Turpethum (Linn.) Silva Manso as a Medicinal Plant Species: A Review on Bioactive Components and Pharmacological Properties.” Pharmacognosy reviews 11(22):158–66.
303. Haaz, S. et al. 2006. “Citrus Aurantium and Synephrine Alkaloids in the Treatment of Overweight and Obesity: An Update.” Obesity reviews : an official journal of the International Association for the Study of Obesity 7(1):79–88.
304. Hadden, W. L. et al. 1999. “Carotenoid Composition of Marigold (Tagetes Erecta) Flower Extract Used as Nutritional Supplement.” Journal of agricultural and food chemistry 47(10):4189–94.
305. Hamdan, Dalia I., Mona F. Mahmoud, Michael Wink, and Assem M. El-Shazly. 2014. “Effect of Hesperidin and Neohesperidin from Bittersweet Orange (Citrus Aurantium Var. Bigaradia) Peel on Indomethacin-Induced Peptic Ulcers in Rats.” Environmental toxicology and pharmacology 37(3):907–15.
306. Han, Xiao-Na et al. 2013. “New Triterpenoids and Other Constituents from the Fruits of Benincasa Hispida (Thunb.) Cogn.” Journal of agricultural and food chemistry 61(51):12692–99.
307. Han, Zhi, Jun Luo, and Ling-Yi Kong. 2012. “Two New Tocopherol Polymers from the Seeds of Euryale Ferox.” Journal of Asian natural products research 14(8):743–47.
308. Handa, S. S., S. K. Gupta, K. Vasisht, A. T. Keene, and J. D. Phillipson. 1984. “Quinoline Alkaloids from Anthocephalus Chinensis.” Planta medica 50(4):358.
309. Hao, Qian, Yoshinori Saito, Yosuke Matsuo, Hai-Zhou Li, and Tanaka Takashi. 2015. “Three New Flavans in Dragon’s Blood from Daemonorops Draco.” Natural product research 29(15):1419–25.
310. Hapidin, Hermizi, Dalila Rozelan, Hasmah Abdullah, Wan Nurhidayah Wan Hanaffi, and Ima Nirwana Soelaiman. 2015. “Quercus Infectoria Gall Extract Enhanced the Proliferation and Activity of Human Fetal Osteoblast Cell Line (hFOB 1.19).” The Malaysian journal of medical sciences : MJMS 22(1):12–22.
311. Haque, M.Ekramul et al. 2004. “E-Octadec-7-En-5-Ynoic Acid from the Roots of Capparis Zeylanica.” Fitoterapia 75(2):130–33.
312. Hasan, Noviany et al. 2012. “The Chemical Components of Sesbania Grandiflora Root and Their Antituberculosis Activity.” Pharmaceuticals (Basel, Switzerland) 5(8):882–89.
313. Hase, Tapio A., Leena Niskanen, and Elias Suokas. 1981. “Comments on the Structure and Synthesis of Jasminol, A Triterpene Reported from Jasminum Auriculatum.” Phytochemistry 20(11):2594–95. Retrieved (http://www.sciencedirect.com/science/article/pii/0031942281831068).
314. Hassan, Emad M., Azza A. Matloub, Mona E. Aboutabl, Nabaweya A. Ibrahim, and Samy M. Mohamed. 2016. “Assessment of Anti-Inflammatory, Antinociceptive, Immunomodulatory, and Antioxidant Activities of Cajanus Cajan L. Seeds Cultivated in Egypt and Its Phytochemical Composition.” Pharmaceutical biology 54(8):1380–91.
315. He, Wen-fei, Jin-cai Lu, Xiao-min Yu, and You-mei Ding. 2007. “[Studies on chemical constituents of leaves of Cassia angustifolia].” Zhong yao cai = Zhongyaocai = Journal of Chinese medicinal materials 30(9):1082–84.
316. He, Wenjun, Taihui Fang, Ke Zhang, and Pengfei Tu. 2009. “[Vasorelaxation effects of homoisoflavonoids from Caesalpinia sappan in rat thoracic aortic rings].” Zhongguo Zhong yao za zhi = Zhongguo zhongyao zazhi = China journal of Chinese materia medica 34(6):731–34.
317. He, X. et al. 2017. “Chemical Composition and Antifungal Activity of Carica Papaya Linn. Seed Essential Oil against Candida Spp.” Letters in applied microbiology 64(5):350–54.
318. He, Xian-guo, Matthew W. Bernart, Li-zhi Lian, and Long-ze Lin. 1998. “High-Performance Liquid Chromatography–electrospray Mass Spectrometric Analysis of Pungent Constituents of Ginger.” Journal of Chromatography A 796(2):327–34.
319. Hedin, P. A., P. L.3rd Lamar, A. C. Thompson, and J. P. Minyard. 1968. “Isolation and Structural Determination of 13 Flavonoid Glycosides in Hibiscus Esculentus (Okra).” American journal of botany 55(4):431–37.
320. van Der Heijden, Robert, Denise I. Jacobs, Wim Snoeijer, Didier Hallard, and Robert Verpoorte. 2004. “The Catharanthus Alkaloids: Pharmacognosy and Biotechnology.” Current medicinal chemistry 11(5):607–28.
321. Herath, H. M., N. S. Kumar, and K. M. Wimalasiri. 1990. “Structural Studies of an Arabinoxylan Isolated from Litsea Glutinosa (Lauraceae).” Carbohydrate research 198(2):343–51.
322. Hikino, H., T. Taguchi, H. Fujimura, and Y. Hiramatsu. 1977. “Antiinflammatory Principles of Caesalpinia Sappan Wood and of Haematoxylon Campechianum Wood.” Planta medica 31(3):214–20.
323. Hiralal Ghante, Mahavir, Kishore P. Bhusari, Nandkishore J. Duragkar, and Nitin B. Ghiware. 2014. “Pharmacological Evaluation for Anti-Asthmatic and Anti-Inflammatory Potential of Woodfordia Fruticosa Flower Extracts.” Pharmaceutical biology 52(7):804–13.
324. Hlel, Takoua Ben et al. 2017. “Variations in the Bioactive Compounds Composition and Biological Activities of Loofah (Luffa Cylindrica) Fruits in Relation to Maturation Stages.” Chemistry & biodiversity 14(10).
325. Holzer, K. and A. Zinke. 1953. “Über Die Bitterstoffe Der Zichorie (Cichorium Intybus L).” Monatshefte für Chemie und verwandte Teile anderer Wissenschaften 84(5):901–9.
326. Holzer, Peter. 2011. “Transient Receptor Potential (TRP) Channels as Drug Targets for Diseases of the Digestive System.” Pharmacology & Therapeutics 131(1):142–70.
327. Hong, C. H., Y. Kim, and S. K. Lee. 2001. “Sesquiterpenoids from the Rhizome of Curcuma Zedoaria.” Archives of pharmacal research 24(5):424–26.
328. Hook, I. L. .. 1994. “Taraxacum Officinale Weber Dandelion In Vitro Culture Micropropagation and the Production of Volatile Metabolites.” Pp. 356–69 in Medicinal and Aromatic Plants- VI, edited by Y. P. S. Bajaj. Springer Science & Business Media.
329. Hosseinkhani, Faride et al. 2016. “Monoterpene Isolated from the Essential Oil of Trachyspermum Ammi Is Cytotoxic to Multidrug-Resistant Pseudomonas Aeruginosa and Staphylococcus Aureus Strains.” Revista da Sociedade Brasileira de Medicina Tropical 49(2):172–76.
330. Hota, Rai K. and Maringanti Bapuji. 1993. “Triterpenoids from the Resin of Shorea Robusta.” Phytochemistry 32(2):466–68. Retrieved (http://www.sciencedirect.com/science/article/pii/S0031942200950192).
331. Howlader, Md Sariful Islam et al. 2017. “Ficus Hispida Bark Extract Prevents Nociception, Inflammation, and CNS Stimulation in Experimental Animal Model.” Evidence-based complementary and alternative medicine : eCAM 2017:7390359.
332. Hu, Qing-Ping, Xin-Ming Cao, Dong-Lin Hao, and Liang-Liang Zhang. 2017. “Chemical Composition, Antioxidant, DNA Damage Protective, Cytotoxic and Antibacterial Activities of Cyperus Rotundus Rhizomes Essential Oil against Foodborne Pathogens.” Scientific reports 7:45231.
333. Huang, Hui-Chi et al. 2016. “Hypoglycemic Constituents Isolated from Trapa Natans L. Pericarps.” Journal of agricultural and food chemistry 64(19):3794–3803.
334. Hussain, Abdullah I. et al. 2014. “Citrullus Colocynthis (L.) Schrad (Bitter Apple Fruit): A Review of Its Phytochemistry, Pharmacology, Traditional Uses and Nutritional Potential.” Journal of ethnopharmacology 155(1):54–66.
335. Idrees, Saiba et al. 2016. “ETHNOBOTANICAL AND BIOLOGICAL ACTIVITIES OF Leptadenia Pyrotechnica (Forssk.) Decne.: A REVIEW.” African journal of traditional, complementary, and alternative medicines : AJTCAM 13(4):88–96.
336. Inamdar, P. K., R. D. Yeole, A. B. Ghogare, and N. J. de Souza. 1996. “Determination of Biologically Active Constituents in Centella Asiatica.” Journal of Chromatography A 742(1):127–30. Retrieved (http://www.sciencedirect.com/science/article/pii/0021967396002373).
337. Iqbal, Kashif, Javeid Iqbal, Dan Staerk, and Kenneth T. Kongstad. 2017. “Characterization of Antileishmanial Compounds from Lawsonia Inermis L. Leaves Using Semi-High Resolution Antileishmanial Profiling Combined with HPLC-HRMS-SPE-NMR.” Frontiers in pharmacology 8:337.
338. Iqbal, Prince Firdoos, Abdul Roouf Bhat, and Amir Azam. 2009. “Antiamoebic Coumarins from the Root Bark of Adina Cordifolia and Their New Thiosemicarbazone Derivatives.” European journal of medicinal chemistry 44(5):2252–59.
339. Iranshahy, Milad et al. 2014. “Adlumiceine Methyl Ester, a New Alkaloid from Fumaria Vaillantii.” Journal of Asian natural products research 16(12):1148–52.
340. Iranshahy, Milad et al. 2017. “A Review of Traditional Uses, Phytochemistry and Pharmacology of Portulaca Oleracea L.” Journal of ethnopharmacology 205:158–72.
341. Islam, Md Khirul et al. 2013. “Preliminary Pharmacological Evaluation of Alocasia Indica Schott Tuber.” Journal of integrative medicine 11(5):343–51.
342. Ito, Tetsuro et al. 2010. “Chemical Constituents in the Leaves of Vateria Indica.” Chemical & pharmaceutical bulletin 58(10):1369–78.
343. Itoh, Atsuko et al. 2005. “Indole Alkaloids and Other Constituents of Rauwolfia Serpentina.” Journal of natural products 68(6):848–52.
344. Ivie, G. W., D. A. Witzel, and D. D. Rushing. 1975. “Toxicity and Milk Bittering Properties of Tenulin, the Major Sesquiterpene Lactone Constituent of Heletiium Amarum (Bitter Sneezeweed).” J. Agric. Food Chem. 23:845.
345. Iyer, Deepa, Brajesh K. Sharma, and U. K. Patil. 2013. “Isolation of Bioactive Phytoconstituent from Alpinia Galanga L. with Anti-Hyperlipidemic Activity.” Journal of dietary supplements 10(4):309–17.
346. Jacobson, Martin. 1977. “Isolation and Identification of Toxic Agents from Plants.” Pp. 10–153 in Host Plant Resistance to Pests, vol. 62, ACS Symposium Series. AMERICAN CHEMICAL SOCIETY.
347. Jadhav, Atul N., Rahul S. Pawar, Bharathi Avula, and Ikhlas A. Khan. 2007. “Ecdysteroid Glycosides from Sida Rhombifolia L.” Chemistry & biodiversity 4(9):2225–30.
348. Jagan Mohan Rao, Lingamallu, Hiroshi Yada, Hiroshi Ono, and Mitsuru Yoshida. 2002. “Acylated and Non-Acylated Flavonol Monoglycosides from the Indian Minor Spice Nagkesar (Mammea Longifolia).” Journal of agricultural and food chemistry 50(11):3143–46.
349. Jain, Mahendra et al. 2012. “Hepatoprotective Activity of Feronia Limonia Root.” The Journal of pharmacy and pharmacology 64(6):888–96.
350. Jain, Ramya, Anjali Sharma, Sanjay Gupta, Indira P. Sarethy, and Reema Gabrani. 2011. “Solanum Nigrum: Current Perspectives on Therapeutic Properties.” Alternative medicine review : a journal of clinical therapeutic 16(1):78–85.
351. Jain, Ritesh and Sanmati Kumar Jain. 2012. “Effect of Buchanania Lanzan Spreng. Bark Extract on Cyclophosphamide Induced Genotoxicity and Oxidative Stress in Mice.” Asian Pacific journal of tropical medicine 5(3):187–91.
352. Jain, Sourabh, Manish Lavhale, and S. Nayak. 2004. “Preliminary Phytochemical Studies on the Roots of Cocculus Hirsutus, Linn.” Ancient science of life 23(3):42–45.
353. Jaiswal, Sunil K., Mukesh K. Dubey, Sanjib Das, and Ch V Rao. 2014. “Gastroprotective Effect of the Iridoid Fraction from Barleria Prionitis Leaves on Experimentally-Induced Gastric Ulceration.” Chinese journal of natural medicines 12(10):738–44.
354. Jaitak, Vikas, Bikram Singh, and V. K. Kaul. 2008. “Variability of Volatile Constituents in Artemisia Maritima in Western Himalaya.” Natural product research 22(7):565–68.
355. Jakribettu, Ramakrishna Pai et al. 2016. “Ginger (Zingiber Officinale Rosc.) Oils.” Pp. 447–54 in Essential Oils in Food Preservation, Flavor and Safety, edited by V. R. Preedy. Academic Press, Elsevier.
356. Jalalpure, S. S. and N. B. Gadge. 2011. “Diuretic Effects of Young Fruit Extracts of Bombax Ceiba L. In Rats.” Indian journal of pharmaceutical sciences 73(3):306–11.
357. Jamadagni, Pallavi Shrirang et al. 2017. “Review of Holarrhena Antidysenterica (L.) Wall. Ex A. DC.: Pharmacognostic, Pharmacological, and Toxicological Perspective.” Pharmacognosy reviews 11(22):141–44.
358. Jamkhande, Prasad G., Sonal R. Barde, Shailesh L. Patwekar, and Priti S. Tidke. 2013. “Plant Profile, Phytochemistry and Pharmacology of Cordia Dichotoma (Indian Cherry): A Review.” Asian Pacific journal of tropical biomedicine 3(12):1009–16.
359. Jana, Sonali and G. S. Shekhawat. 2011. “Critical Review on Medicinally Potent Plant Species: Gloriosa Superba.” Fitoterapia 82(3):293–301.
360. Janbaz, Khalid Hussain et al. 2012. “Pharmacological Evaluation of Prosopis Cineraria (L.) Druce in Gastrointestinal, Respiratory, and Vascular Disorders.” Evidence-based complementary and alternative medicine : eCAM 2012:735653.
361. Jang, Hyun-Jae et al. 2016. “Eudesmane-Type Sesquiterpenoids from Salvia Plebeia Inhibit IL-6-Induced STAT3 Activation.” Phytochemistry 130:335–42.
362. Jang, M. K., D. H. Sohn, and J. H. Ryu. 2001. “A Curcuminoid and Sesquiterpenes as Inhibitors of Macrophage TNF-Alpha Release from Curcuma Zedoaria.” Planta medica 67(6):550–52.
363. Jang, Mi Kyung, Hwa Jin Lee, Ji Sun Kim, and Jae-Ha Ryu. 2004. “A Curcuminoid and Two Sesquiterpenoids from Curcuma Zedoaria as Inhibitors of Nitric Oxide Synthesis in Activated Macrophages.” Archives of pharmacal research 27(12):1220–25.
364. Jarald, E.Edwin, S. B. Joshi, and D. C. Jain. 2008. “Antidiabetic Activity of Flower Buds of Michelia Champaca Linn.” Indian journal of pharmacology 40(6):256–60.
365. Jayaprakasha, G. K., P. S. Negi, and B. S. Jena. 2006. “Antioxidative and Antimutagenic Activities of the Extracts from the Rinds of Garcinia Pedunculata.” Innovative Food Science & Emerging Technologies 7(3):246–50. Retrieved (http://www.sciencedirect.com/science/article/pii/S1466856406000233).
366. Jeong, Gil-Saeng et al. 2009. “Cytoprotective Constituents of the Heartwood of Caesalpinia Sappan on Glutamate-Induced Oxidative Damage in HT22 Cells.” Biological & pharmaceutical bulletin 32(5):945–49.
367. Jeyadevi, R., T. Sivasudha, A. Ilavarasi, and N. Thajuddin. 2013. “Chemical Constituents and Antimicrobial Activity of Indian Green Leafy Vegetable Cardiospermum Halicacabum.” Indian journal of microbiology 53(2):208–13.
368. Jha, S. and S. Sen. 1983. “Quantitation of Principal Bufadienolides in Different Cytotypes of Urginea Indica.” Planta medica 47(1):43–45.
369. Jhade, D., D. Ahirwar, R. Jain, Nk Sharma, and S. Gupta. 2011. “Pharmacognostic Standardization, Physico- and Phytochemical Evaluation of Amaranthus Spinosus Linn. Root.” Journal of young pharmacists : JYP 3(3):221–25.
370. Jia, A. L. et al. 2008. “A New Indole Alkaloid from Arundo Donax L.” Journal of Asian natural products research 10(1–2):105–9.
371. Jia, Sheng et al. 2015. “Hypoglycemic and Hypolipidemic Effects of Neohesperidin Derived from Citrus Aurantium L. in Diabetic KK-A(y) Mice.” Food & function 6(3):878–86.
372. Jiang, Shuang et al. 2016. “Effects of Marsdenia Tenacissima Polysaccharide on the Immune Regulation and Tumor Growth in H22 Tumor-Bearing Mice.” Carbohydrate polymers 137:52–58.
373. Jiang, Xiang, Fei Kuang, Fansheng Kong, and Chunyan Yan. 2016. “Prediction of the Antiglycation Activity of Polysaccharides from Benincasa Hispida Using a Response Surface Methodology.” Carbohydrate polymers 151:358–63.
374. Jin, Min-Rong, Hong Xu, Chao-Hui Duan, and Gui-Xin Chou. 2015. “Two New Flavones from Salvia Plebeia.” Natural product research 29(14):1315–22.
375. Jin, Xiao-feng, Yan-hua Lu, Dong-zhi Wei, and Zheng-tao Wang. 2008. “Chemical Fingerprint and Quantitative Analysis of Salvia Plebeia R.Br. by High-Performance Liquid Chromatography.” Journal of pharmaceutical and biomedical analysis 48(1):100–104.
376. Jing, Wen-Guang et al. 2014. “[Chemical constituents from seeds of Brassica campestris].” Zhongguo Zhong yao za zhi = Zhongguo zhongyao zazhi = China journal of Chinese materia medica 39(13):2521–25.
377. Johri, R. K. 2011. “Cuminum Cyminum and Carum Carvi: An Update.” Pharmacognosy reviews 5(9):63–72.
378. Jonville, Marie-Caroline et al. 2013. “Dimeric Bisindole Alkaloids from the Stem Bark of Strychnos Nux-Vomica L.” Phytochemistry 87:157–63.
379. Joshi, Bipin Chandra, Anuj Pandey, Ram Prakash Sharma, and Anakshi Khare. 2003. “Quassinoids from Ailanthus Excelsa.” Phytochemistry 62(4):579–84.
380. Joshi, Khem R., Hari P. Devkota, and Shoji Yahara. 2012. “Chemical Analysis of Heartwood of Bijayasal (Pterocarpus Marsupium Roxb.).” Nepal Journal of Science and Technology 13(2):219–24.
381. Joshi, Rajesh Kumar. 2013. “Volatile Composition and Antimicrobial Activity of the Essential Oil of Artemisia Absinthium Growing in Western Ghats Region of North West Karnataka, India.” Pharmaceutical biology 51(7):888–92.
382. Joshi, Robin et al. 2013. “Analysis of the Essential Oil of Large Cardamom (Amomum Subulatum Roxb.) Growing in Different Agro-Climatic Zones of Himachal Pradesh, India.” Journal of the science of food and agriculture 93(6):1303–9.
383. Judzentiene, Asta, Felix Tomi, and Joseph Casanova. 2009. “Analysis of Essential Oils of Artemisia Absinthium L. from Lithuania by CC, GC(RI), GC-MS and 13C NMR.” Natural product communications 4(8):1113–18.
384. Juteau, Fabien et al. 2003. “Composition and Antimicrobial Activity of the Essential Oil of Artemisia Absinthium from Croatia and France.” Planta medica 69(2):158–61.
385. Kadir, Roszaini, Khairul Awang, Zaitihaiza Khamaruddin, and Zaini Soit. 2015. “Chemical Compositions and Termiticidal Activities of the Heartwood from Calophyllum Inophyllum L.” Anais da Academia Brasileira de Ciencias 87(2):743–51.
386. Kalachaveedu, Mangathayaru, Sarah Kuruvilla, and K. Balakrishna. 2011. “Effect of Erythrina Variegata on Experimental Atherosclerosis in Guinea Pigs.” Journal of pharmacology & pharmacotherapeutics 2(4):285–87.
387. Kalaiselvan, A., T. Anand, K. Gokulakrishnan, M. C. Kamaraj, and S. Velavan. 2015. “Modulatory Role of Shorea Robusta Bark on Glucose-Metabolizing Enzymes in Diethylnitrosamine Induced Hepatocellular Carcinoma in Rats.” Pharmacognosy magazine 11(Suppl 3):S496-500.
388. Kalauni, Surya K. et al. 2004. “Cassane- and Norcassane-Type Diterpenes of Caesalpinia Crista from Myanmar.” Journal of natural products 67(11):1859–63.
389. Kalauni, Surya Kant et al. 2005a. “Methyl Migrated Cassane-Type Furanoditerpenes of Caesalpinia Crista from Myanmar.” Chemical & pharmaceutical bulletin 53(10):1300–1304.
390. Kalauni, Surya Kant et al. 2005b. “New Cassane-Type Diterpenes of Caesalpinia Crista from Myanmar.” Chemical & pharmaceutical bulletin 53(2):214–18.
391. Kale, Mrudula, A. V Misar, Vivek Dave, Maruti Joshi, and A. M. Mujumdar. 2007. “Anti-Inflammatory Activity of Dalbergia Lanceolaria Bark Ethanol Extract in Mice and Rats.” Journal of ethnopharmacology 112(2):300–304.
392. Kanaujia, Anil et al. 2010. “Insulinomimetic Activity of Two New Gallotannins from the Fruits of Capparis Moonii.” Bioorganic & medicinal chemistry 18(11):3940–45.
393. Kandimalla, Raghuram et al. 2016. “Antioxidant and Hepatoprotective Potentiality of Randia Dumetorum Lam. Leaf and Bark via Inhibition of Oxidative Stress and Inflammatory Cytokines.” Frontiers in pharmacology 7:205.
394. Kaou, Ali Mohamed et al. 2010. “Antimalarial Compounds from the Aerial Parts of Flacourtia Indica (Flacourtiaceae).” Journal of ethnopharmacology 130(2):272–74.
395. Kar, A., M. K. Menon, and C. S. Chauhan. 1970. “Effect of Essential Oil of Litsea Glutinosa (Lour.) C. B. Robins on Cardiovascular System and Isolated Tissues.” Indian journal of experimental biology 8(1):61–62.
396. Kar, Ashutosh. 2003. Pharmacognosy and Pharmacobiotechnology. New Dehli: New Age International (P) Limited.
397. Karmase, Aniket, K. Prasanna, Sruti Rasabattula, and Kamlesh K. Bhutani. 2014. “Quantification and Comparison of Extraction Methods for Alkaloids in Aegle Marmelos Leaves by HPLC.” Natural product communications 9(7):981–83.
398. Kashima, Yusei, Satoshi Nakaya, and Mitsuo Miyazawa. 2014. “Volatile Composition and Sensory Properties of Indian Herbal Medicine-Pavonia Odorata-Used in Ayurveda.” Journal of oleo science 63(2):149–58.
399. Kashima, Yusei, Hidehiko Yamaki, Takuya Suzuki, and Mitsuo Miyazawa. 2011. “Insecticidal Effect and Chemical Composition of the Volatile Oil from Bergenia Ligulata.” Journal of agricultural and food chemistry 59(13):7114–19.
400. Kaur, Prabhjit et al. 2010. “Modulatory Role of Alizarin from Rubia Cordifolia L. against Genotoxicity of Mutagens.” Food and chemical toxicology : an international journal published for the British Industrial Biological Research Association 48(1):320–25.
401. Kaur, S., R. Dayal, V. K. Varshney, and J. P. Bartley. 2001. “GC-MS Analysis of Essential Oils of Heartwood and Resin of Shorea Robusta.” Planta medica 67(9):883–86.
402. Kaur, Sandeep, V. K. Varshney, and Rameshwar Dayal. 2003. “GC-MS Analysis of Essential Oil of Shorea Robusta Bast.” Journal of Asian natural products research 5(3):231–34.
403. Kaushik, Dhirender, Jyoti Yadav, Pawan Kaushik, Disha Sacher, and Ruby Rani. 2011. “Current Pharmacological and Phytochemical Studies of the Plant Alpinia Galanga.” Zhong xi yi jie he xue bao = Journal of Chinese integrative medicine 9(10):1061–65.
404. Kaushik, Pawan, Dhirender Kaushik, and Sukhbir Lal Khokra. 2013. “Ethnobotany and Phytopharmacology of Pinus Roxburghii Sargent: A Plant Review.” Journal of integrative medicine 11(6):371–76.
405. Kaveh, Mahsa, Akram Eidi, Ali Nemati, and Mohammad Hossein Boskabady. 2017. “The Extract of Portulaca Oleracea and Its Constituent, Alpha Linolenic Acid Affects Serum Oxidant Levels and Inflammatory Cells in Sensitized Rats.” Iranian journal of allergy, asthma, and immunology 16(3):256–70.
406. Kazuma, Kohei, Naonobu Noda, and Masahiko Suzuki. 2003a. “Flavonoid Composition Related to Petal Color in Different Lines of Clitoria Ternatea.” Phytochemistry 64(6):1133–39.
407. Kazuma, Kohei, Naonobu Noda, and Masahiko Suzuki. 2003b. “Malonylated Flavonol Glycosides from the Petals of Clitoria Ternatea.” Phytochemistry 62(2):229–37.
408. Kesari, Achyut Narayan, Rajesh Kumar Gupta, and Geeta Watal. 2004. “Two Aurone Glycosides from Heartwood of Pterocarpus Santalinus.” Phytochemistry 65(23):3125–29.
409. Keshavarz, A., M. Minaiyan, A. Ghannadi, and P. Mahzouni. 2013. “Effects of Carum Carvi L. (Caraway) Extract and Essential Oil on TNBS-Induced Colitis in Rats.” Research in pharmaceutical sciences 8(1):1–8.
410. Khadeer Ahamed, Mohamed B., Venkatarangaiah Krishna, and Chethan J. Dandin. 2010. “In Vitro Antioxidant and in Vivo Prophylactic Effects of Two Gamma-Lactones Isolated from Grewia Tiliaefolia against Hepatotoxicity in Carbon Tetrachloride Intoxicated Rats.” European journal of pharmacology 631(1–3):42–52.
411. Khalid, S. A., S. M. Yagi, P. Khristova, and H. Duddeck. 1989. “(+)-Catechin-5-Galloyl Ester as a Novel Natural Polyphenol from the Bark of Acacia Nilotica of Sudanese Origin1.” Planta medica 55(6):556–58.
412. Khan, Imran, Payare L. Sangwan, Jagdish K. Dhar, and Surrinder Koul. 2012. “Simultaneous Quantification of Five Marker Compounds of Betula Utilis Stem Bark Using a Validated High-Performance Thin-Layer Chromatography Method.” Journal of separation science 35(3):392–99.
413. Khan, M. R., M. Kihara, and A. D. Omoloso. 2002. “Antimicrobial Activity of Michelia Champaca.” Fitoterapia 73(7–8):744–48.
414. Khan, Mohammad Ataullah and Atta-ur-Rahman. 1975. “Karachic Acid: A New Triterpenoid from Betula Utilis.” Phytochemistry 14(3):789–91. Retrieved (http://www.sciencedirect.com/science/article/pii/0031942275830366).
415. Khan, Noor Afshan and Ashutosh Srivastava. 2009. “Antifungal Activity of Bioactive Triterpenoid Saponin from the Seeds of Cassia Angustifolia.” Natural product research 23(12):1128–33.
416. Khan, Zeba, M. Ali, and Priyanka Bagri. 2010. “A New Steroidal Glycoside and Fatty Acid Esters from the Stem Bark of Tectona Grandis Linn.” Natural product research 24(11):1059–68.
417. Khanra, Ritu, Saikat Dewanjee, Tarun K. Dua, and Niloy Bhattacharjee. 2017. “Taraxerol, a Pentacyclic Triterpene from Abroma Augusta Leaf, Attenuates Acute Inflammation via Inhibition of NF-kappaB Signaling.” Biomedicine & pharmacotherapy = Biomedecine & pharmacotherapie 88:918–23.
418. Khare, C. P. 2004. Indian Herbal Remedies: Rational Western Therapy, Ayurvedic and Other Traditional Usage, Botany. edited by C. P. Khare. Berlin, Heidelberg: Springer Berlin Heidelberg.
419. Khazdair, Mohammad Reza, Mohammad Hossein Boskabady, Mahmoud Hosseini, Ramin Rezaee, and Aristidis M Tsatsakis. 2015. “The Effects of Crocus Sativus (Saffron) and Its Constituents on Nervous System: A Review.” Avicenna journal of phytomedicine 5(5):376–91.
420. Khorasany, Alireza Rezaee and Hossein Hosseinzadeh. 2016. “Therapeutic Effects of Saffron (Crocus Sativus L.) in Digestive Disorders: A Review.” Iranian journal of basic medical sciences 19(5):455–69.
421. Kidwai, AR et al. 1949. “Chemical Examination of Tinospora Cordifolia.” J Sci Ind Res 8:115–18.
422. Kikuchi, Takashi et al. 2014. “Three New Triterpene Esters from Pumpkin (Cucurbita Maxima) Seeds.” Molecules (Basel, Switzerland) 19(4):4802–13.
423. Kim, K. I. et al. 2001. “Effects of Polysaccharides from Rhizomes of Curcuma Zedoaria on Macrophage Functions.” Bioscience, biotechnology, and biochemistry 65(11):2369–77.
424. Kim, Tae Hoon et al. 2017. “Antifungal and Ichthyotoxic Sesquiterpenoids from Santalum Album Heartwood.” Molecules (Basel, Switzerland) 22(7).
425. Kim, Young-Soo, Chan Sik Hong, Sang Weon Lee, Joo Hyun Nam, and Byung Joo Kim. 2017. “Effects of Ginger and Its Pungent Constituents on Transient Receptor Potential Channels.” Biophysical Journal 112(3, Supplement 1):250a.
426. Kini, Shruthi G. et al. 2015. “Studies on the Chitin Binding Property of Novel Cysteine-Rich Peptides from Alternanthera Sessilis.” Biochemistry 54(43):6639–49.
427. Kinoshita, Takeshi, Yasuhiro Haga, Shintaro Narimatsu, Motoko Shimada, and Yukihiro Goda. 2005. “The Isolation and Structure Elucidation of New Cassane Diterpene-Acids from Caesalpinia Crista L. (Fabaceae), and Review on the Nomenclature of Some Caesalpinia Species.” Chemical & pharmaceutical bulletin 53(6):717–20.
428. Kiple, Kenneth F. and Kriemhild Coneè Ornelas, eds. 2000. The Cambridge World History of Food. Vol.1. Cambridge University Press.
429. Kishi, Akinobu, Toshio Morikawa, Hisashi Matsuda, and Masayuki Yoshikawa. 2003. “Structures of New Friedelane- and Norfriedelane-Type Triterpenes and Polyacylated Eudesmane-Type Sesquiterpene from Salacia Chinensis LINN. (S. Prinoides DC., Hippocrateaceae) and Radical Scavenging Activities of Principal Constituents.” Chemical & pharmaceutical bulletin 51(9):1051–55.
430. Klasek, A., V. Simanek, and F. Santavy. 1972. “Alkaloids from Aconitum Plants. I. Isolation of Bikhaconitine, Chasmaconitine, Indaconitine and Pseudaconitine from Aconitum Ferox.” Lloydia 35(1):55–60.
431. Kokkalou, E. 1988. “The Constituents of the Essential Oil from Lavandula Stoechas Growing Wild in Greece.” Planta medica 54(1):58–59.
432. Kolniak-Ostek, Joanna. 2016. “Chemical Composition and Antioxidant Capacity of Different Anatomical Parts of Pear (Pyrus Communis L.).” Food chemistry 203:491–97.
433. Komath, S. S., S. K. Nadimpalli, and M. J. Swamy. 1996. “Purification in High Yield and Characterisation of the Galactose-Specific Lectin from the Seeds of Snake Gourd (Trichosanthes Anguina).” Biochemistry and molecular biology international 39(2):243–52.
434. Kosuge, Sadayoshi and Masaji Furuta. 1970. “Studies on the Pungent Principle of Capsicum.” Agricultural and Biological Chemistry 34(2):248–56.
435. Kothavade, Pankaj S. et al. 2015. “Therapeutic Effect of Saponin Rich Fraction of Achyranthes Aspera Linn. on Adjuvant-Induced Arthritis in Sprague-Dawley Rats.” Autoimmune diseases 2015:943645.
436. Kreutzmann, Stine, Lars P. Christensen, and Merete Edelenbos. 2008. “Investigation of Bitterness in Carrots (Daucus Carota L.) Based on Quantitative Chemical and Sensory Analyses.” LWT - Food Science and Technology 41(2):193–205.
437. Krishnan, V. and S. Rangaswami. 1967. “Proanthocyanidins of Salacia Chinensis Linn.” Tetrahedron letters 26:2441–46.
438. Krishnaveni, A. and Santh Rani Thaakur. 2006. “Pharmacognostical and Preliminary Phytochemical Studies of Achyranthes Aspera Linn.” Ancient science of life 26(1–2):1–5.
439. Krishnaveni, K. S. and J. V Rao. 2000. “An Isoflavone from Pterocarpus Santalinus.” Phytochemistry 53(5):605–6.
440. Krishnaveni, K. S. and J. V Srinivasa Rao. 2000a. “A New Acylated Isoflavone Glucoside from Pterocarpus Santalinus.” Chemical & pharmaceutical bulletin 48(9):1373–74.
441. Krishnaveni, K. S. and J. V Srinivasa Rao. 2000b. “A New Triterpene from Callus of Pterocarpus Santalinus.” Fitoterapia 71(1):10–13.
442. Kshirsagar, A. D., K. G. Ingale, N. S. Vyawahare, and V. S. Thorve. 2010. “Hygrophila Spinosa: A Comprehensive Review.” Pharmacognosy reviews 4(8):167–71.
443. Kulkarni, Yogesh A., Ritesh Panjabi, Vishvas Patel, Aditi Tawade, and Alok Gokhale. 2013. “Effect of Gmelina Arborea Roxb in Experimentally Induced Inflammation and Nociception.” Journal of Ayurveda and integrative medicine 4(3):152–57.
444. Kulkarni, Yogesh Anant and Addepalli Veeranjaneyulu. 2013. “Effects of Gmelina Arborea Extract on Experimentally Induced Diabetes.” Asian Pacific journal of tropical medicine 6(8):602–8.
445. Kumar, A., S. Lingadurai, A. Jain, and N. R. Barman. 2010. “Erythrina Variegata Linn: A Review on Morphology, Phytochemistry, and Pharmacological Aspects.” Pharmacognosy reviews 4(8):147–52.
446. Kumar, A.Senthil, V. Venkatesalu, K. Kannathasan, and M. Chandrasekaran. 2010. “Chemical Constituents and Antibacterial Activity of the Leaf Essential Oil of Feronia Limonia.” Indian journal of microbiology 50(Suppl 1):70–73.
447. Kumar, Ashish et al. 2014. “A New Ellagic Acid Glycoside and DNA Topoisomerase IB Inhibitory Activity of Saponins from Putranjiva Roxburghii.” Natural product communications 9(5):675–77.
448. Kumar, Ashish et al. 2016. “A New Bisbenzylisoquinoline Alkaloid Isolated from Thalictrum Foliolosum, as a Potent Inhibitor of DNA Topoisomerase IB of Leishmania Donovani.” Fitoterapia 109:25–30.
449. Kumar, Dinesh, Zulfiqar Ali Bhat, Vijender Kumar, and M. Y. Shah. 2013. “Coumarins from Angelica Archangelica Linn. and Their Effects on Anxiety-like Behavior.” Progress in neuro-psychopharmacology & biological psychiatry 40:180–86.
450. Kumar, Dinesh, Rakesh Kumar, Bikram Singh, and Paramvir Singh Ahuja. 2016. “Comprehensive Chemical Profiling of Picrorhiza Kurroa Royle Ex Benth Using NMR, HPTLC and LC-MS/MS Techniques.” Combinatorial chemistry & high throughput screening 19(3):200–215.
451. Kumar, G. P., S. Sudheesh, and N. R. Vijayalakshmi. 1993. “Hypoglycaemic Effect of Coccinia Indica: Mechanism of Action.” Planta medica 59(4):330–32.
452. Kumar, Gnanesh, Padmanabh Mishra, Vellareddy Anantharam, and Avadhesha Surolia. 2015. “Luffa Acutangula Agglutinin: Primary Structure Determination and Identification of a Tryptophan Residue Involved in Its Carbohydrate-Binding Activity Using Mass Spectrometry.” IUBMB life 67(12):943–53.
453. Kumar, Gopal, Baby Chauhan, and Mohammed Ali. 2014. “Isolation and Identification of New Phytoconstituents from the Fruit Extract of Amomum Subulatum Roxb.” Natural product research 28(2):127–33.
454. Kumar, Manmeet et al. 2010. “Anti-Osteoporotic Constituents from Indian Medicinal Plants.” Phytomedicine 17(13):993–99. Retrieved (http://www.sciencedirect.com/science/article/pii/S0944711310001066).
455. Kumar, Neeraj, Bikram Singh, Pamita Bhandari, Ajai Prakash Gupta, and Vijay Kumar Kaul. 2007. “Steroidal Alkaloids from Holarrhena Antidysenterica (L.) WALL.” Chemical & pharmaceutical bulletin 55(6):912–14.
456. Kumar, Nirmal and Anil Kumar Singh. 2014. “Plant Profile, Phytochemistry and Pharmacology of Avartani (Helicteres Isora Linn.): A Review.” Asian Pacific journal of tropical biomedicine 4(Suppl 1):S22-6.
457. Kumar, Nitesh et al. 2014. “Atypical Antidepressant Activity of 3,4-Bis(3,4-Dimethoxyphenyl) Furan-2,5-Dione Isolated from Heart Wood of Cedrus Deodara, in Rodents.” The Korean journal of physiology & pharmacology : official journal of the Korean Physiological Society and the Korean Society of Pharmacology 18(5):365–69.
458. Kumar, Nitin, Satyendra Singh, Manvi, and Rajiv Gupta. 2012. “Trichosanthes Dioica Roxb.: An Overview.” Pharmacognosy reviews 6(11):61–67.
459. Kumar, Padam et al. 2014. “Neoflavonoids as Potential Osteogenic Agents from Dalbergia Sissoo Heartwood.” Bioorganic & medicinal chemistry letters 24(12):2664–68.
460. Kumar, R.Sunil, Ramesh Balenahalli Narasingappa, Chandrashekhar G. Joshi, Talakatta K. Girish, and Ananda Danagoudar. 2017. “Caesalpinia Crista Linn. Induces Protection against DNA and Membrane Damage.” Pharmacognosy magazine 13(Suppl 2):S250–57.
461. Kumar, Rohit, Yogendra Kumar Gupta, Surender Singh, and S. Arunraja. 2015. “Cissus Quadrangularis Attenuates the Adjuvant Induced Arthritis by down Regulating pro-Inflammatory Cytokine and Inhibiting Angiogenesis.” Journal of ethnopharmacology 175:346–55.
462. Kumar, S., U. K. Sharma, A. K. Sharma, and A. K. Pandey. 2012. “Protective Efficacy of Solanum Xanthocarpum Root Extracts against Free Radical Damage: Phytochemical Analysis and Antioxidant Effect.” Cellular and molecular biology (Noisy-le-Grand, France) 58(1):174–81.
463. Kumar, S. V.Suresh and S. H. Mishra. 2014. “Protective Effect of Extracts of Baliospermum Montanum (Willd.) Muell.-Arg. against Paracetamol-Induced Hepatotoxicity-an in Vivo and in Vitro Study.” Ancient science of life 33(4):216–21.
464. Kumar, S.Sravan, P. Manoj, N. P. Shetty, Maya Prakash, and P. Giridhar. 2015. “Characterization of Major Betalain Pigments -Gomphrenin, Betanin and Isobetanin from Basella Rubra L. Fruit and Evaluation of Efficacy as a Natural Colourant in Product (Ice Cream) Development.” Journal of food science and technology 52(8):4994–5002.
465. Kumar, Sandopu Sravan, Prabhakaran Manoj, Girish Nimisha, and Parvatam Giridhar. 2016. “Phytoconstituents and Stability of Betalains in Fruit Extracts of Malabar Spinach (Basella Rubra L.).” Journal of food science and technology 53(11):4014–22.
466. Kumar, Sanjeev, Satyendra Gautam, and Arun Sharma. 2013. “Identification of Antimutagenic Properties of Anthocyanins and Other Polyphenols from Rose (Rosa Centifolia) Petals and Tea.” Journal of food science 78(6):H948-54.
467. Kumar, V., R. Chand, A. Auzi, Y. Ikeshiro, and S. D. Sarker. 2003. “2’-(2,3-Dihydroxybenzoyloxy)-7-Ketologanin: A Novel Iridoid Glucoside from the Leaves of Gentiana Kurroo.” Die Pharmazie 58(9):668–70.
468. Kumar, Vijay and Johannes Van Staden. 2015. “A Review of Swertia Chirayita (Gentianaceae) as a Traditional Medicinal Plant.” Frontiers in pharmacology 6:308.
469. Kumar Varma, Chekuri Ashok and K. Jayaram Kumar. 2017. “Structural, Functional and pH Sensitive Release Characteristics of Water-Soluble Polysaccharide from the Seeds of Albizia Lebbeck L.” Carbohydrate polymers 175:502–8.
470. Kumari, Sima, Meetali Deori, R. Elancheran, Jibon Kotoky, and Rajlakshmi Devi. 2016. “In Vitro and In Vivo Antioxidant, Anti-Hyperlipidemic Properties and Chemical Characterization of Centella Asiatica (L.) Extract.” Frontiers in pharmacology 7:400.
471. Kumawat, Ramkumar, Sunil Sharma, and Suresh Kumar. 2012. “An Overview for Various Aspects of Multifaceted, Health Care Tecomella Undulata Seem. Plant.” Acta poloniae pharmaceutica 69(5):993–96.
472. Kunert, Olaf et al. 2009. “Cycloartane Triterpenes from Dikamali, the Gum Resin of Gardenia Gummifera and Gardenia Lucida.” Chemistry & biodiversity 6(8):1185–92.
473. Kunhachan, Phanukit, Chuleratana Banchonglikitkul, Tanwarat Kajsongkram, Amonrat Khayungarnnawee, and Wichet Leelamanit. 2012. “Chemical Composition, Toxicity and Vasodilatation Effect of the Flowers Extract of Jasminum Sambac (L.) Ait. ‘G. Duke of Tuscany’.” Evidence-based complementary and alternative medicine : eCAM 2012:471312.
474. Kuo, Ping-Chung et al. 2008. “Chemical Constituents from Abutilon Indicum.” Journal of Asian natural products research 10(7–8):699–703.
475. Kuusi, T., H. Pyysalo, and K. Autio. 1985. “The Bitterness Properties of Dandelion. II. Chemical Investigations.” Lebensmittel-Wiss Technol 18:347–49.
476. Kwak, Jung-Ho et al. 2017. “Variation of Quercetin Glycoside Derivatives in Three Onion (Allium Cepa L.) Varieties.” Saudi journal of biological sciences 24(6):1387–91.
477. Lacret, Rodney, Rosa M. Varela, Jose M. G. Molinillo, Clara Nogueiras, and Francisco A. Macias. 2011. “Anthratectone and Naphthotectone, Two Quinones from Bioactive Extracts of Tectona Grandis.” Journal of chemical ecology 37(12):1341–48.
478. Laghari, Abdul Hafeez et al. 2012. “Determination of Free Phenolic Acids and Antioxidant Capacity of Methanolic Extracts Obtained from Leaves and Flowers of Camel Thorn (Alhagi Maurorum).” Natural product research 26(2):173–76.
479. Lakshmi, C., K.Akshaya Kumar, T. J. Dennis, and T. S. S. P. N. S.Sanath Kumar. 2011. “Antibacterial Activity of Polyphenols of Garcinia Indica.” Indian journal of pharmaceutical sciences 73(4):470–73.
480. Lan, Mei, Ping Wan, Zhi-Ying Wang, and Xiao-Lan Huang. 2012. “[GC-MS analysis of chemical components in seeds oil from Croton tiglium].” Zhong yao cai = Zhongyaocai = Journal of Chinese medicinal materials 35(7):1105–8.
481. Lavhale, Manish S., Santosh Kumar, Shri Hari Mishra, and Sandhya L. Sitasawad. 2009. “A Novel Triterpenoid Isolated from the Root Bark of Ailanthus Excelsa Roxb (Tree of Heaven), AECHL-1 as a Potential Anti-Cancer Agent.” PloS one 4(4):e5365.
482. Lawrence, Lincy, Seema Menon, Sheka Vincent, Vipin P. Sivaram, and Jose Padikkala. 2016. “Radical Scavenging and Gastroprotective Activity of Methanolic Extract of Gmelina Arborea Stem Bark.” Journal of Ayurveda and integrative medicine 7(2):78–82.
483. Lebedeva, A. A. et al. 2017. “Bactericide, Immunomodulating, and Wound Healing Properties of Transgenic Kalanchoe Pinnata Synergize with Antimicrobial Peptide Cecropin P1 In Vivo.” Journal of immunology research 2017:4645701.
484. Lee, Ching-Kuo, Ping-Hung Lee, and Yueh-Hsmng Kuo. 2001. “The Chemical Constituents from the Aril of Cassia Fistula L.” Journal of the Chinese Chemical Society 48:1053–1058.
485. Lefar, M. S., D. Firestone, E. C. Coleman, N. Brown, and D. W. Shaw. 1968. “Lipids from the Seeds of Abrus Precatorius.” Journal of pharmaceutical sciences 57(8):1442–44.
486. Leivas, Carolina Lopes, Marcello Iacomini, and Lucimara M. C. Cordeiro. 2015. “Structural Characterization of a Rhamnogalacturonan I-Arabinan-Type I Arabinogalactan Macromolecule from Starfruit (Averrhoa Carambola L.).” Carbohydrate polymers 121:224–30.
487. Leivas, Carolina Lopes, Marcello Iacomini, and Lucimara M. C. Cordeiro. 2016. “Pectic Type II Arabinogalactans from Starfruit (Averrhoa Carambola L.).” Food chemistry 199:252–57.
488. Li, Bi-Jun et al. 2017. “Two New Stilbene Trimers from Cynodon Dactylon.” Natural product research 31(21):2479–83.
489. Li, Chen et al. 2014. “Tandem Mass Spectrometric Fragmentation Behavior of Lignans, Flavonoids and Triterpenoids in Streblus Asper.” Rapid communications in mass spectrometry : RCM 28(21):2363–70.
490. Li, Da-Hong et al. 2016. “Two New Benzylisoquinoline Alkaloids from Thalictrum Foliolosum and Their Antioxidant and in Vitro Antiproliferative Properties.” Archives of pharmacal research 39(7):871–77.
491. Li, Jia-Zhou, Hua-Nong Mo, and Xian-Mao Ning. 2009. “[Study on chemical constituents of tress of Toona ciliata].” Zhong yao cai = Zhongyaocai = Journal of Chinese medicinal materials 32(10):1539–42.
492. Li, Kuiyong et al. 2013. “Purification of Amide Alkaloids from Piper Longum L. Using Preparative Two-Dimensional Normal-Phase Liquid Chromatography X Reversed-Phase Liquid Chromatography.” The Analyst 138(11):3313–20.
493. Li, Li et al. 2017. “Three New Sesquiterpenes from Pterocarpus Santalinus.” Journal of Asian natural products research 1–7.
494. Li, Mengfei, Lanlan Zhou, Delong Yang, Tiantian Li, and Wei Li. 2012. “Biochemical Composition and Antioxidant Capacity of Extracts from Podophyllum Hexandrum Rhizome.” BMC complementary and alternative medicine 12:263.
495. Li, Xing-Cui, Chao Liu, Li-Xin Yang, and Ruo-Yun Chen. 2011. “Phenolic Compounds from the Aqueous Extract of Acacia Catechu.” Journal of Asian natural products research 13(9):826–30.
496. Li, Xingcui, Hongqing Wang, Chao Liu, and Ruoyun Chen. 2010. “[Chemical constituents of Acacia catechu].” Zhongguo Zhong yao za zhi = Zhongguo zhongyao zazhi = China journal of Chinese materia medica 35(11):1425–27.
497. Li, Yuan-Li et al. 2007. “A Flavonoid Glycoside Isolated from Smilax China L. Rhizome in Vitro Anticancer Effects on Human Cancer Cell Lines.” Journal of ethnopharmacology 113(1):115–24.
498. Li, Yue-Ting et al. 2016. “[Chemical constituents from the fruits of Vitex negundo var. cannabifolia and their biological activities in vitro].” Zhongguo Zhong yao za zhi = Zhongguo zhongyao zazhi = China journal of Chinese materia medica 41(22):4197–4203.
499. Liao, Shang-Gao et al. 2007. “Limonoids from the Leaves and Stems of Toona Ciliata.” Journal of natural products 70(8):1268–73.
500. Lim, Hye-Sun, Sung-Eun Jin, Ohn-Soon Kim, Hyeun-Kyoo Shin, and Soo-Jin Jeong. 2015. “Alantolactone from Saussurea Lappa Exerts Antiinflammatory Effects by Inhibiting Chemokine Production and STAT1 Phosphorylation in TNF-Alpha and IFN-Gamma-Induced in HaCaT Cells.” Phytotherapy research : PTR 29(7):1088–96.
501. Lim, Seol-Wa et al. 2016. “Protective Effects of a Polymethoxy Flavonoids-Rich Citrus Aurantium Peel Extract on Liver Fibrosis Induced by Bile Duct Ligation in Mice.” Asian Pacific journal of tropical medicine 9(12):1158–64.
502. Lima, E. B. C. et al. 2015. “Cocos Nucifera (L.) (Arecaceae): A Phytochemical and Pharmacological Review.” Brazilian journal of medical and biological research = Revista brasileira de pesquisas medicas e biologicas 48(11):953–64.
503. Lin, An-Shen et al. 2009. “Acasiane A and B and Farnesirane A and B, Diterpene Derivatives from the Roots of Acacia Farnesiana.” Planta medica 75(3):256–61.
504. Lin, Peng and Tzi Bun Ng. 2008. “Preparation and Biological Properties of a Melibiose Binding Lectin from Bauhinia Variegata Seeds.” Journal of agricultural and food chemistry 56(22):10481–86.
505. Lin, Xuejing et al. 2016. “Volatile Oil from Saussurea Lappa Exerts Antitumor Efficacy by Inhibiting Epithelial Growth Factor Receptor Tyrosine Kinase-Mediated Signaling Pathway in Hepatocellular Carcinoma.” Oncotarget 7(48):79761–73.
506. Linn, Thein Zaw et al. 2005. “Cassane- and Norcassane-Type Diterpenes from Caesalpinia Crista of Indonesia and Their Antimalarial Activity against the Growth of Plasmodium Falciparum.” Journal of natural products 68(5):706–10.
507. Liu, Hao-Long et al. 2015. “Identification and Simultaneous Quantification of Five Alkaloids in Piper Longum L. by HPLC-ESI-MS(n) and UFLC-ESI-MS/MS and Their Application to Piper Nigrum L.” Food chemistry 177:191–96.
508. Liu, Lu et al. 2015. “Antibacterial Monoterpenoid Indole Alkaloids from Alstonia Scholaris Cultivated in Temperate Zone.” Fitoterapia 105:160–64.
509. Liu, Qi-Bing et al. 2015. “Two New Cassane-Type Diterpenes from the Seeds of Caesalpinia Crista.” Journal of Asian natural products research 17(11):1073–78.
510. Liu, Qi, Jingang Yu, Xiaoyun Liao, Peisen Zhang, and Xiaoqing Chen. 2015. “One-Step Separation of Antioxidant Compounds from Erythrina Variegata by High Speed Counter-Current Chromatography.” Journal of chromatographic science 53(5):730–35.
511. Liu, Wei-Hsien et al. 2015. “Calophyllolide Content in Calophyllum Inophyllum at Different Stages of Maturity and Its Osteogenic Activity.” Molecules (Basel, Switzerland) 20(7):12314–27.
512. Loizzo, Monica Rosa et al. 2007. “Inhibition of Angiotensin Converting Enzyme (ACE) by Flavonoids Isolated from Ailanthus Excelsa (Roxb) (Simaroubaceae).” Phytotherapy research : PTR 21(1):32–36.
513. Lu, Qun, Li Yang, Hai-Yan Zhao, Jian-Guo Jiang, and Xi-Lin Xu. 2013. “Protective Effect of Compounds from the Flowers of Citrus Aurantium L. Var. Amara Engl against Carbon Tetrachloride-Induced Hepatocyte Injury.” Food and chemical toxicology : an international journal published for the British Industrial Biological Research Association 62:432–35.
514. Lutz, Andrea and Peter Winterhalter. 1994. “Dihydroabscisic Alcohol from Averrhoa Carambola Fruit.” Phytochemistry 36(3):811–12. Retrieved (http://www.sciencedirect.com/science/article/pii/S0031942200898246).
515. Ma, Chengyao, Yayun Chen, Jianwei Chen, Xiang Li, and Yong Chen. 2017. “A Review on Annona Squamosa L.: Phytochemicals and Biological Activities.” The American journal of Chinese medicine 45(5):933–64.
516. Ma, Cui-Ying, Wing Keung Liu, and Chun-Tao Che. 2002. “Lignanamides and Nonalkaloidal Components of Hyoscyamus Niger Seeds.” Journal of natural products 65(2):206–9.
517. Ma, Williams, and Che. 1999. “Withanolides from Hyoscyamus Niger Seeds.” Journal of natural products 62(10):1445–47.
518. Madan, Swati, Steve T. Pannakal, Seru Ganapaty, Gyanendra N. Singh, and Yatendra Kumar. 2009. “Phenolic Glucosides from Flacourtia Indica.” Natural product communications 4(3):381–84.
519. Madruga, Marta Suely et al. 2014. “Chemical, Morphological and Functional Properties of Brazilian Jackfruit (Artocarpus Heterophyllus L.) Seeds Starch.” Food chemistry 143:440–45.
520. Mahato, S. B., S. K. Banerjee, and R. N. Chakravarti. 1966. “Triterpenes of Artocarpus Lakoocha Roxb.” Bulletin of the Calcutta School of Tropical Medicine 14(1):16.
521. Mahran, G. H., A. H. Saber, and T. el-Alfy. 1968. “Spectrophotometric Determination of Protoanemonin, Anemonin and Ranunculin in Ranunculus Sceleratus L.” Planta medica 16(3):323–28.
522. Mai, Dinh Tri et al. 2015. “A New Aldehyde Compound from the Fruit of Pandanus Tectorius Parkinson Ex Du Roi.” Natural product research 29(15):1437–41.
523. Maji, Amal K., Subrata Pandit, Pratim Banerji, and Debdulal Banerjee. 2014. “Pueraria Tuberosa: A Review on Its Phytochemical and Therapeutic Potential.” Natural product research 28(23):2111–27.
524. Makabe, H., N. Maru, A. Kuwabara, T. Kamo, and M. Hirota. 2006. “Anti-Inflammatory Sesquiterpenes from Curcuma Zedoaria.” Natural product research 20(7):680–85.
525. Makihara, Hiroko et al. 2016. “Gallic Acid, the Active Ingredient of Terminalia Bellirica, Enhances Adipocyte Differentiation and Adiponectin Secretion.” Biological & pharmaceutical bulletin 39(7):1137–43.
526. Mali, Prashant Y. and Shital S. Panchal. 2013. “A Review on Phyto-Pharmacological Potentials of Euphorbia Thymifolia L.” Ancient science of life 32(3):165–72.
527. Mali, R. G. and R. R. Wadekar. 2008. “In Vitro Anthelmintic Activity of Baliospermum Montanum Muell. Arg Roots.” Indian journal of pharmaceutical sciences 70(1):131–33.
528. Malsawmtluangi, C. et al. 2014. “Physicochemical Characteristics and Antioxidant Activity of Prunus Cerasoides D. Don Gum Exudates.” International journal of biological macromolecules 69:192–99.
529. Mancuso, Giuseppe, Gigliola Borgonovo, Leonardo Scaglioni, and Angela Bassoli. 2015. “Phytochemicals from Ruta Graveolens Activate TAS2R Bitter Taste Receptors and TRP Channels Involved in Gustation and Nociception.” Molecules (Basel, Switzerland) 20(10):18907–22.
530. Maneechai, Suthira et al. 2009. “Quantitative Analysis of Oxyresveratrol Content in Artocarpus Lakoocha and ‘Puag-Haad’.” Medical principles and practice : international journal of the Kuwait University, Health Science Centre 18(3):223–27.
531. Maneechai, Suthira, Wanchai De-Eknamkul, Kaoru Umehara, Hiroshi Noguchi, and Kittisak Likhitwitayawuid. 2012. “Flavonoid and Stilbenoid Production in Callus Cultures of Artocarpus Lakoocha.” Phytochemistry 81:42–49.
532. Manikandaselvi, Sambasivam, Vellingiri Vadivel, and Pemaiah Brindha. 2016. “Studies on Physicochemical and Nutritional Properties of Aerial Parts of Cassia Occidentalis L.” Journal of food and drug analysis 24(3):508–15.
533. Manna, Dipak, Pradeep K. Dutta, Basudeb Achari, and Anuradha Lohia. 2010. “A Novel Galacto-Glycerolipid from Oxalis Corniculata Kills Entamoeba Histolytica and Giardia Lamblia.” Antimicrobial agents and chemotherapy 54(11):4825–32.
534. Manners, Gary D. 2007. “Citrus Limonoids: Analysis, Bioactivity, and Biomedical Prospects.” Journal of agricultural and food chemistry 55(21):8285–94.
535. Manouze, Houria et al. 2017. “Anti-Inflammatory, Antinociceptive, and Antioxidant Activities of Methanol and Aqueous Extracts of Anacyclus Pyrethrum Roots.” Frontiers in pharmacology 8:598.
536. Maqsood, Maria, Dildar Ahmed, Iqra Atique, and Wajeeha Malik. 2017. “Lipase Inhibitory Activity of Lagenaria Siceraria Fruit as a Strategy to Treat Obesity.” Asian Pacific journal of tropical medicine 10(3):305–10.
537. Marikkar, J. M. N., H. M. Ghazali, and K. Long. 2010. “Composition and Thermal Characteristics of Madhuca Longifolia Seed Fat and Its Solid and Liquid Fractions.” Journal of oleo science 59(1):7–14.
538. Marongiu, Bruno, Alessandra Piras, Silvia Porcedda, and Andrea Scorciapino. 2005. “Chemical Composition of the Essential Oil and Supercritical CO2 Extract of Commiphora Myrrha (Nees) Engl. and of Acorus Calamus L.” Journal of agricultural and food chemistry 53(20):7939–43.
539. Masada, Yoshiro, Keiji Hashimoto, Takehisa Inoue, and Mario Suzuki. 1971. “Analysis of the Pungent Principles of Capsicum Annuum by Combined Gas Chromatography-Mass Spectrometry.” Journal of Food Science 36(6):858–60.
540. Mascolo, N., R. Sharma, S. C. Jain, and F. Capasso. 1988. “Ethnopharmacology of Calotropis Procera Flowers.” Journal of ethnopharmacology 22(2):211–21.
541. Massiot, G. et al. 1983. “Occurence of Longicaudatine, a New Type of Bis-Indole Base and Bisnor-Calkaloid H in Strychnos Species.” J. Org. Chem. 48:1869–1872.
542. Matlawska, Irena and Maria Sikorska. 2002. “Flavonoid Compounds in the Flowers of Abutilon Indicum (L.) Sweet (Malvaceae).” Acta poloniae pharmaceutica 59(3):227–29.
543. Matsuda, Hisashi et al. 2009. “Hepatoprotective Amide Constituents from the Fruit of Piper Chaba: Structural Requirements, Mode of Action, and New Amides.” Bioorganic & medicinal chemistry 17(20):7313–23.
544. Matsuo, Yukiko, Hiroshi Sakagami, and Yoshihiro Mimaki. 2014. “A Rare Type of Sesquiterpene and Beta-Santalol Derivatives from Santalum Album and Their Cytotoxic Activities.” Chemical & pharmaceutical bulletin 62(12):1192–99.
545. Maurya, Rakesh et al. 2009. “Osteogenic Activity of Constituents from Butea Monosperma.” Bioorganic & medicinal chemistry letters 19(3):610–13.
546. Maurya, Santosh Kumar, Ashwini Kumar Kushwaha, and Ankit Seth. 2015. “Ethnomedicinal Review of Usnakantaka (Echinops Echinatus Roxb.).” Pharmacognosy reviews 9(18):149–54.
547. Mazumder, Sutapa, Claudine Morvan, Swapnadip Thakur, and Bimalendu Ray. 2004. “Cell Wall Polysaccharides from Chalkumra (Benincasa Hispida) Fruit. Part I. Isolation and Characterization of Pectins.” Journal of agricultural and food chemistry 52(11):3556–62.
548. Meesakul, Pornphimol et al. 2016. “A New Cytotoxic Clerodane Diterpene from Casearia Graveolens Twigs.” Natural product communications 11(1):13–15.
549. Mehenni, Chafiaa et al. 2016. “Hepatoprotective and Antidiabetic Effects of Pistacia Lentiscus Leaf and Fruit Extracts.” Journal of food and drug analysis 24(3):653–69.
550. Mehla, Kamiya et al. 2011. “Ethyl Gallate Isolated from Pistacia Integerrima Linn. Inhibits Cell Adhesion Molecules by Blocking AP-1 Transcription Factor.” Journal of ethnopharmacology 137(3):1345–52.
551. Mejia-Garibay, Beatriz, Enrique Palou, and Aurelio Lopez-Malo. 2015. “Composition, Diffusion, and Antifungal Activity of Black Mustard (Brassica Nigra) Essential Oil When Applied by Direct Addition or Vapor Phase Contact.” Journal of food protection 78(4):843–48.
552. Menichini, Francesco et al. 2014. “Chemical Profiling and in Vitro Biological Effects of Cardiospermum Halicacabum L. (Sapindaceae) Aerial Parts and Seeds for Applications in Neurodegenerative Disorders.” Journal of enzyme inhibition and medicinal chemistry 29(5):677–85.
553. Menon, Nikhil, Jean Sparks, and Felix O. Omoruyi. 2016. “Oxidative Stress Parameters and Erythrocyte Membrane Adenosine Triphosphatase Activities in Streptozotocin-Induced Diabetic Rats Administered Aqueous Preparation of Kalanchoe Pinnata Leaves.” Pharmacognosy research 8(2):85–88.
554. Meriga, Balaji et al. 2017. “Ethanolic Fraction of Terminalia Tomentosa Attenuates Biochemical and Physiological Derangements in Diet Induced Obese Rat Model by Regulating Key Lipid Metabolizing Enzymes and Adipokines.” Pharmacognosy magazine 13(51):385–92.
555. Michl, Johanna et al. 2013. “Is Aristolochic Acid Nephropathy a Widespread Problem in Developing Countries? A Case Study of Aristolochia Indica L. in Bangladesh Using an Ethnobotanical-Phytochemical Approach.” Journal of ethnopharmacology 149(1):235–44.
556. Mihajilov-Krstev, Tatjana et al. 2014. “Antimicrobial, Antioxidative, and Insect Repellent Effects of Artemisia Absinthium Essential Oil.” Planta medica 80(18):1698–1705.
557. Mikail, Hudu Garba, Helen Karvouni, Antonia Kotsiou, Christina Tesseromatis, and Prokopios Magiatis. 2015. “New Alkylresorcinols from a Lipophilic Extract of Urginea Indica L. Bulbs Showing Experimental Trauma Healing Activity.” Fitoterapia 101:41–45.
558. Mills, Clive, Anthony R. Carroll, and Ronald J. Quinn. 2005. “Acutangulosides A-F, Monodesmosidic Saponins from the Bark of Barringtonia Acutangula.” Journal of natural products 68(3):311–18.
559. Min, Byung Sun et al. 2012. “Compounds from the Heartwood of Caesalpinia Sappan and Their Anti-Inflammatory Activity.” Bioorganic & medicinal chemistry letters 22(24):7436–39.
560. Mishra, Amita, Amit Kumar Sharma, Shashank Kumar, Ajit K. Saxena, and Abhay K. Pandey. 2013. “Bauhinia Variegata Leaf Extracts Exhibit Considerable Antibacterial, Antioxidant, and Anticancer Activities.” BioMed research international 2013:915436.
561. Mishra, M., Y. N. Shukla, and S. Kumar. 2000. “Euphane Triterpenoid and Lipid Constituents from Butea Monosperma.” Phytochemistry 54(8):835–38.
562. Mishra, Rohit K. et al. 2016. “Pharmaco-Phylogenetic Investigation of Methyl Gallate Isolated from Acacia Nilotica (L.) Delile and Its Cytotoxic Effect on NIH3T3 Mouse Fibroblast.” Current pharmaceutical biotechnology 17(6):540–48.
563. Mishra, Sunil K., A. Kumar, and A. Talukdar. 2010. “Evaluation of Binding Property of Mucilage from Litsea Glutinosa Wall.” Pharmacognosy research 2(5):289–92.
564. Misra, G., S. C. Bhatnagar, and S. K. Nigam. 1977. “Constituents of Holoptelea Integrifolia Heartwood.” Planta medica 31(3):232–34.
565. Misra, Laxmi N. and Ateeque Ahmad. 1997. “Triterpenoids from Shorea Robusta Resin.” Phytochemistry 45(3):575–78. Retrieved (http://www.sciencedirect.com/science/article/pii/S0031942297000046).
566. Mitani, Kaoru et al. 2013. “Suppression of Melanin Synthesis by the Phenolic Constituents of Sappanwood (Caesalpinia Sappan).” Planta medica 79(1):37–44.
567. Mitra, Mazumder Papiya, Das Saumya, Das Sanjita, and Das Manas Kumar. 2011. “PHYTO-PHARMACOLOGY OF BERBERIS ARISTATA DC: A REVIEW.” Journal of Drug Delivery & Therapeutics 1(2):46–50.
568. Miyaichi, Yukinori, Akiko Segawa, and Tsuyoshi Tomimori. 2006. “Studies on Nepalese Crude Drugs. XXIX. Chemical Constituents of Dronapuspi, the Whole Herb of Leucas Cephalotes SPRENG.” Chemical & pharmaceutical bulletin 54(10):1370–79.
569. Mizokami, Hiroki, Kaori Tomita-Yokotani, and Kunijiro Yoshitama. 2008. “Flavonoids in the Leaves of Oxalis Corniculata and Sequestration of the Flavonoids in the Wing Scales of the Pale Grass Blue Butterfly, Pseudozizeeria Maha.” Journal of plant research 121(1):133–36.
570. Mnif, Sami and Sami Aifa. 2015. “Cumin (Cuminum Cyminum L.) from Traditional Uses to Potential Biomedical Applications.” Chemistry & biodiversity 12(5):733–42.
571. Modi, Manoj et al. 2013. “Anti-HIV-1 Activity, Protease Inhibition and Safety Profile of Extracts Prepared from Rhus Parviflora.” BMC complementary and alternative medicine 13:158.
572. Mohamed, Mona A., Madeha R. Mammoud, and Heiko Hayen. 2009. “Evaluation of Antinociceptive and Anti-Inflammatory Activities of a New Triterpene Saponin from Bauhinia Variegata Leaves.” Zeitschrift fur Naturforschung. C, Journal of biosciences 64(11–12):798–808.
573. Mohammadi, A. et al. 2014. “Seasonal Variation in the Chemical Composition, Antioxidant Activity, and Total Phenolic Content of Artemisia Absinthium Essential Oils.” Pharmacognosy research 7(4):329–34.
574. Mohammed, Magdy M. D., Nabaweya A. Ibrahim, Fatma S. El-Sakhawy, Khaled M. Mohamed, and Doaa A. H. Deabes. 2016. “Two New Cytotoxic Furoquinoline Alkaloids Isolated from Aegle Marmelos (Linn.) Correa.” Natural product research 1–8.
575. Mohan, Shikha and Damodar Gupta. 2017. “Phytochemical Analysis and Differential in Vitro Cytotoxicity Assessment of Root Extracts of Inula Racemosa.” Biomedicine & pharmacotherapy = Biomedecine & pharmacotherapie 89:781–95.
576. Mohan Maruga Raja, Muthu Kumaradoss and Shri Hari Mishra. 2010. “Comprehensive Review of Clerodendrum Phlomidis: A Traditionally Used Bitter.” Zhong xi yi jie he xue bao = Journal of Chinese integrative medicine 8(6):510–24.
577. Mohanty, Sudipta Kumar, Mallappa Kumara Swamy, Uma Rani Sinniah, and Maniyam Anuradha. 2017. “Leptadenia Reticulata (Retz.) Wight & Arn. (Jivanti): Botanical, Agronomical, Phytochemical, Pharmacological, and Biotechnological Aspects.” Molecules (Basel, Switzerland) 22(6).
578. Mohod, Smeeta M., Amit D. Kandhare, and Subhash L. Bodhankar. 2016. “Gastroprotective Potential of Pentahydroxy Flavone Isolated from Madhuca Indica J. F. Gmel. Leaves against Acetic Acid-Induced Ulcer in Rats: The Role of Oxido-Inflammatory and Prostaglandins Markers.” Journal of ethnopharmacology 182:150–59.
579. Molfetta, Ilaria, Lucia Ceccarini, Mario Macchia, Guido Flamini, and Pier Luigi Cioni. 2013. “Abelmoschus Esculentus (L.) Moench. and Abelmoschus Moschatus Medik: Seeds Production and Analysis of the Volatile Compounds.” Food chemistry 141(1):34–40.
580. Momin, Mohammad Abdul Motalib et al. 2014. “Phytopharmacological Evaluation of Ethanol Extract of Sida Cordifolia L. Roots.” Asian Pacific journal of tropical biomedicine 4(1):18–24.
581. Mondal, Arijit, Tanmoy Guria, and Tapan Kumar Maity. 2015. “A New Ester of Fatty Acid from a Methanol Extract of the Whole Plant of Amaranthus Spinosus and Its Alpha-Glucosidase Inhibitory Activity.” Pharmaceutical biology 53(4):600–604.
582. Mondal, Arijit, Tanmoy Guria, Tapan Kumar Maity, and Anupam Bishayee. 2016. “A Novel Tetraenoic Fatty Acid Isolated from Amaranthus Spinosus Inhibits Proliferation and Induces Apoptosis of Human Liver Cancer Cells.” International journal of molecular sciences 17(10).
583. Mondal, Himangsu et al. 2014. “Central-Stimulating and Analgesic Activity of the Ethanolic Extract of Alternanthera Sessilis in Mice.” BMC complementary and alternative medicine 14:398.
584. Monzote, Lianet, Abel Pinon, Ramon Sculli, and William N. Setzer. 2014. “Chemistry and Leishmanicidal Activity of the Essential Oil from Artemisia Absinthium from Cuba.” Natural product communications 9(12):1799–1804.
585. Moon, Hyung-In, Ill-Min Chung, Su-Hyun Seo, and Eun-Young Kang. 2010. “Protective Effects of 3’-deoxy-4-O-Methylepisappanol from Caesalpinia Sappan against Glutamate-Induced Neurotoxicity in Primary Cultured Rat Cortical Cells.” Phytotherapy research : PTR 24(3):463–65.
586. Moorthy, I.Ganesh et al. 2017. “Ultrasound Assisted Extraction of Pectin from Waste Artocarpus Heterophyllus Fruit Peel.” Ultrasonics sonochemistry 34:525–30.
587. Morgan, E.David and Ian D. Wilson. 1999. “Insect Hormones and Insect Chemical Ecology.” Pp. 263–375 in Comprehensive Natural Products Chemistry, edited by M.-C. Otto, B. Sir Derek, and N. Koji.
588. Morikawa, Toshio, Hisashi Matsuda, Itadaki Yamaguchi, Yutana Pongpiriyadacha, and Masayuki Yoshikawa. 2004. “New Amides and Gastroprotective Constituents from the Fruit of Piper Chaba.” Planta medica 70(2):152–59.
589. Morris, John Bradley. 2009. “Morphological and Reproductive Characterization in Hyacinth Bean, Lablab Purpureus (L.) Sweet Germplasm with Clinically Proven Nutraceutical and Pharmaceutical Traits for Use as a Medicinal Food.” Journal of dietary supplements 6(3):263–79.
590. Morris, John Bradley, Brandon Tonnis, and Ming Li Wang. 2014. “Flavonol Content, Oil%, and Fatty Acid Composition Variability in Seeds of Teramnus Labialis and T. Uncinatus Accessions with Nutraceutical Potential.” Journal of dietary supplements 11(3):294–303.
591. Mossa, Abdel-Tawab H., Amel A. Refaie, Amal Ramadan, and Jalloul Bouajila. 2013. “Amelioration of Prallethrin-Induced Oxidative Stress and Hepatotoxicity in Rat by the Administration of Origanum Majorana Essential Oil.” BioMed research international 2013:859085.
592. Motevalian, Manijeh, Saeed Mehrzadi, Samira Ahadi, and Asie Shojaii. 2017. “Anticonvulsant Activity of Dorema Ammoniacum Gum: Evidence for the Involvement of Benzodiazepines and Opioid Receptors.” Research in pharmaceutical sciences 12(1):53–59.
593. Mubashir, Khan, Khalid Ghazanfar, et al. 2014. “Scientific Validation of Gentiana Kurroo Royle for Anti-Inflammatory and Immunomodulatory Potential.” ISRN inflammation 2014:701765.
594. Mubashir, Khan, Bashir A. Ganai, Khalid Ghazanfar, and Seema Akbar. 2014. “Evaluation of Antiarthritic Potential of Methanolic Extract of Gentiana Kurroo Royle.” Arthritis 2014:810615.
595. Muchtaridi, Anas Subarnas, Anton Apriyantono, and Resmi Mustarichie. 2010. “Identification of Compounds in the Essential Oil of Nutmeg Seeds (Myristica Fragrans Houtt.) That Inhibit Locomotor Activity in Mice.” International journal of molecular sciences 11(11):4771–81.
596. Mujeeb, Farina, Preeti Bajpai, and Neelam Pathak. 2014. “Phytochemical Evaluation, Antimicrobial Activity, and Determination of Bioactive Components from Leaves of Aegle Marmelos.” BioMed research international 2014:497606.
597. Mukherjee, Tulika et al. 2012. “Putralone, a Novel 10alpha-Hydroxy-25-nor D:A Friedo-Oleanane Triterpenoid from Putranjiva Roxburghii.” Natural product communications 7(4):511–13.
598. Mulla, Wa, Sb Kuchekar, Vs Thorat, Ar Chopade, and Bs Kuchekar. 2010. “Antioxidant, Antinociceptive and Anti-Inflammatory Activities of Ethanolic Extract of Leaves of Alocasia Indica (Schott.).” Journal of young pharmacists : JYP 2(2):137–43.
599. Muluye, Abrham Belachew, Eshetie Melese, and Getnet Mequanint Adinew. 2015. “Antimalarial Activity of 80 % Methanolic Extract of Brassica Nigra (L.) Koch. (Brassicaceae) Seeds against Plasmodium Berghei Infection in Mice.” BMC complementary and alternative medicine 15:367.
600. Murakami, T., K. Hirano, and M. Yoshikawa. 2001. “Medicinal Foodstuffs. XXIII. Structures of New Oleanane-Type Triterpene Oligoglycosides, Basellasaponins A, B, C, and D, from the Fresh Aerial Parts of Basella Rubra L.” Chemical & pharmaceutical bulletin 49(6):776–79.
601. Muralikrishnan, Kameshwaran, Sharath Asokan, P. R. Geetha Priya, K.Syed Zameer Ahmed, and G. Ayyappadasan. 2017. “Comparative Evaluation of the Local Anesthetic Activity of Root Extract of Anacyclus Pyrethrum and Its Interaction at the Site of Injection in Guinea Pigs.” Anesthesia, essays and researches 11(2):444–48.
602. Murthy, Papolu Bhargava Sriramachandra et al. 2006. “Estimation of Twelve Bacopa Saponins in Bacopa Monnieri Extracts and Formulations by High-Performance Liquid Chromatography.” Chemical & pharmaceutical bulletin 54(6):907–11.
603. Murti, Krishna, Upendra Kumar, and Mayank Panchal. 2011. “Healing Promoting Potentials of Roots of Ficus Benghalensis L. in Albino Rats.” Asian Pacific journal of tropical medicine 4(11):921–24.
604. Muthu, Narmataa, Su Yin Lee, Kia Kien Phua, and Subhash Janardhan Bhore. 2016. “Nutritional, Medicinal and Toxicological Attributes of Star-Fruits (Averrhoa Carambola L.): A Review.” Bioinformation 12(12):420–24.
605. Muthukrishnan, Saradha Devi, Ashokkumar Kaliyaperumal, and Annapoorani Subramaniyan. 2015. “Identification and Determination of Flavonoids, Carotenoids and Chlorophyll Concentration in Cynodon Dactylon (L.) by HPLC Analysis.” Natural product research 29(8):785–90.
606. Muthukrishnan, Suriyavathana et al. 2016. “Phytochemical Profile of Erythrina Variegata by Using High-Performance Liquid Chromatography and Gas Chromatography-Mass Spectroscopy Analyses.” Journal of acupuncture and meridian studies 9(4):207–12.
607. Nagabhushana, Kyatanahalli S. et al. 2002. “Inhibition of Soybean and Potato Lipoxygenases by Bhilawanols from Bhilawan (Semecarpus Anacardium) Nut Shell Liquid and Some Synthetic Salicylic Acid Analogues.” Journal of enzyme inhibition and medicinal chemistry 17(4):255–59.
608. Nagao, T., R. Tanaka, Y. Iwase, H. Hanazono, and H. Okabe. 1991. “Studies on the Constituents of Luffa Acutangula Roxb. I. Structures of Acutosides A--G, Oleanane-Type Triterpene Saponins Isolated from the Herb.” Chemical & pharmaceutical bulletin 39(3):599–606.
609. Nahak, Gayatri and Rajani Kanta Sahu. 2011. “Phytochemical Evaluation and Antioxidant Activity of Piper Cubeba and Piper Nigrum.” Journal of Applied Pharmaceutical Science 1(8):153–57.
610. Nair, Gopakumar Ramachandran et al. 2016. “Clinical Effectiveness of Aloe Vera in the Management of Oral Mucosal Diseases- A Systematic Review.” Journal of clinical and diagnostic research : JCDR 10(8):ZE01-7.
611. Nair, Vimal, Woo Young Bang, Elisa Schreckinger, Nuri Andarwulan, and Luis Cisneros-Zevallos. 2015. “Protective Role of Ternatin Anthocyanins and Quercetin Glycosides from Butterfly Pea (Clitoria Ternatea Leguminosae) Blue Flower Petals against Lipopolysaccharide (LPS)-Induced Inflammation in Macrophage Cells.” Journal of agricultural and food chemistry 63(28):6355–65.
612. Nakamura, Seikou et al. 2011. “Chemical Structures and Hepatoprotective Effects of Constituents from the Leaves of Salacia Chinensis.” Chemical & pharmaceutical bulletin 59(8):1020–28.
613. Nakamura, Seikou et al. 2016. “Structures of Aromatic Glycosides from the Seeds of Cassia Auriculata.” Chemical & pharmaceutical bulletin 64(7):970–74.
614. Nam, Nguyen-Hai et al. 2004. “New Constituents from Crinum Latifolium with Inhibitory Effects against Tube-like Formation of Human Umbilical Venous Endothelial Cells.” Natural product research 18(6):485–91.
615. Namazi, Masoumeh et al. 2014. “Effects of Citrus Aurantium (Bitter Orange) on the Severity of First-Stage Labor Pain.” Iranian journal of pharmaceutical research : IJPR 13(3):1011–18.
616. Narang, Nithithep and Wannee Jiraungkoorskul. 2016. “Anticancer Activity of Key Lime, Citrus Aurantifolia.” Pharmacognosy reviews 10(20):118–22.
617. Narender, Tadigoppula, Tanvir Khaliq, Anju Puri, and Ramesh Chander. 2006. “Antidyslipidemic Activity of Furano-Flavonoids Isolated from Indigofera Tinctoria.” Bioorganic & medicinal chemistry letters 16(13):3411–14.
618. Nasiri, Ebrahim et al. 2015. “Effect of Malva Sylvestris Cream on Burn Injury and Wounds in Rats.” Avicenna journal of phytomedicine 5(4):341–54.
619. Natarajan, Abirami, Shobana Sugumar, Sivakumar Bitragunta, and Natarajan Balasubramanyan. 2015. “Molecular Docking Studies of (4Z, 12Z)-Cyclopentadeca-4, 12-Dienone from Grewia Hirsuta with Some Targets Related to Type 2 Diabetes.” BMC complementary and alternative medicine 15:73.
620. Nawaz, H. R., A. Malik, P. M. Khan, S. Shujaat, and A. Rahman. 2000. “A Novel Beta-Glucuronidase Inhibiting Triterpenoid from Paeonia Emodi.” Chemical & pharmaceutical bulletin 48(11):1771–73.
621. Ndunda, Bethe. 2014. “Phytochemistry and Bioactivity Investigations of Three Kenyan Croton Species.”
622. Negi, Bhawna Sunil and Bharti P. Dave. 2010. “In Vitro Antimicrobial Activity of Acacia Catechu and Its Phytochemical Analysis.” Indian journal of microbiology 50(4):369–74.
623. Nguyen, Hai Xuan et al. 2013. “Cleistanthane Diterpenes from the Seed of Caesalpinia Sappan and Their Antiausterity Activity against PANC-1 Human Pancreatic Cancer Cell Line.” Fitoterapia 91:148–53.
624. Nguyen, Hai Xuan et al. 2016. “A New Cassane-Type Diterpene from the Seed of Caesalpinia Sappan.” Natural product communications 11(6):723–24.
625. Nguyen, Mai Thanh Thi, Suresh Awale, Yasuhiro Tezuka, Quan Le Tran, and Shigetoshi Kadota. 2005. “Xanthine Oxidase Inhibitors from the Heartwood of Vietnamese Caesalpinia Sappan.” Chemical & pharmaceutical bulletin 53(8):984–88.
626. Nguyen, Tan Phat et al. 2016. “A New Dihydrofurocoumarin from the Fruits of Pandanus Tectorius Parkinson Ex Du Roi.” Natural product research 30(21):2389–95.
627. Nguyen, Van Du et al. 2014. “Flavan-3-Ols from the Barks of Barringtonia Acutangula.” Biochemical Systematics and Ecology 55(Supplement C):219–21. Retrieved (http://www.sciencedirect.com/science/article/pii/S0305197814001197).
628. Nhiem, Nguyen Xuan et al. 2015. “1H and 13C NMR Assignments of Tricanguinas A-B, Coumarin Monoterpenes from Trichosanthes Anguina L.” Magnetic resonance in chemistry : MRC 53(2):178–80.
629. Niiho, Yujiro et al. 2006. “Gastroprotective Effects of Bitter Principles Isolated from Gentian Root and Swertia Herb on Experimentally-Induced Gastric Lesions in Rats.” Journal of Natural Medicines 60(1):82–88.
630. Ning, Jing et al. 2010. “Triterpenoids from the Leaves of Toona Ciliata.” Journal of Asian natural products research 12(6):448–52.
631. Niranjan Reddy, V. L., V. Ravikanth, V. V. N. S. Jansi Lakshmi, U. Suryanarayan Murty, and Y. Venkateswarlu. 2003. “Inhibitory Activity of Homoisoflavonoids from Caesalpinia Sappan against Beauveria Bassiana.” Fitoterapia 74(6):600–602.
632. Nisar, Muhammad et al. 2009. “New Diterpenoid Alkaloids from Aconitum Heterophyllum Wall: Selective Butyrylcholinestrase Inhibitors.” Journal of enzyme inhibition and medicinal chemistry 24(1):47–51.
633. Nomura, H. 1917. “Pungent Principles of Ginger. I. A New Ketone, Zingiberone, Occurring in Ginger.” Sci Rep Tohoku Imp Univ 6:41–52.
634. Nomura, Taro, Toshio Fukai, and Toshiyuki Akiyama. 2002. “Chemistry of Phenolic Compounds of Licorice (Glycyrrhiza Species) and Their Estrogenic and Cytotoxic Activities.” Pure Appl. Chem. 74(7):1199–1206.
635. Nooreen, Zulfa et al. 2017. “Characterization and Evaluation of Bioactive Polyphenolic Constituents from Zanthoxylum Armatum DC., a Traditionally Used Plant.” Biomedicine & pharmacotherapy = Biomedecine & pharmacotherapie 89:366–75.
636. Note, Olivier Placide et al. 2015. “Triterpenoid Saponins from Albizia Lebbeck (L.) Benth and Their Inhibitory Effect on the Survival of High Grade Human Brain Tumor Cells.” Carbohydrate research 404:26–33.
637. Nugroho, Agung Endro, Yance Anas, et al. 2011. “Effects of Marmin, a Compound Isolated from Aegle Marmelos Correa, on Contraction of the Guinea Pig-Isolated Trachea.” Pakistan journal of pharmaceutical sciences 24(4):427–33.
638. Nugroho, Agung Endro, Sugeng Riyanto, Mohamad Aspollah Sukari, and Kazutaka Maeyama. 2011. “Effects of Aegeline, a Main Alkaloid of Aegle Marmelos Correa Leaves, on the Histamine Release from Mast Cells.” Pakistan journal of pharmaceutical sciences 24(3):359–67.
639. Ogura, M., GA Cordell, and N. R. Fransworth. 1978. “Alkaloid Constituents of A. Excelsa.” Lloydia 41(2):166–68.
640. Ogura, M., K. Koike, G. A. Cordell, and N. R. Farnsworth. 1978. “Potential Anticancer Agents VIII. Constituents of Baliospermum Montanum (Euphorbiaceae).” Planta medica 33(2):128–43.
641. Ojha, Arnab K. et al. 2008. “Structural Assignment of a Heteropolysaccharide Isolated from the Gum of Cochlospermum Religiosum (Katira Gum).” Carbohydrate research 343(7):1222–31.
642. Okunade, Olukayode Adediran, Sameer Khalil Ghawi, Lisa Methven, and Keshavan Niranjan. 2015. “Thermal and Pressure Stability of Myrosinase Enzymes from Black Mustard (Brassica Nigra L. W.D.J. Koch. Var. Nigra), Brown Mustard (Brassica Juncea L. Czern. Var. Juncea) and Yellow Mustard (Sinapsis Alba L. Subsp. Maire) Seeds.” Food chemistry 187:485–90.
643. Orhan, Ilkay Erdogan. 2012. “Centella Asiatica (L.) Urban: From Traditional Medicine to Modern Medicine with Neuroprotective Potential.” Evidence-based complementary and alternative medicine : eCAM 2012:946259.
644. Padhye, Subhash, Aamir Ahmad, Nikhil Oswal, and Fazlul H. Sarkar. 2009. “Emerging Role of Garcinol, the Antioxidant Chalcone from Garcinia Indica Choisy and Its Synthetic Analogs.” Journal of hematology & oncology 2:38.
645. Padmavathi, Dharamaraj, Lakshmi Susheela, and Rajkishore Vijaya Bharathi. 2011. “Pharmacognostical Evaluation of Barringtonia Acutangula Leaf.” International journal of Ayurveda research 2(1):37–41.
646. Pai, Sandeep R., Vinayak Upadhya, Harsha V Hegde, Rajesh K. Joshi, and Sanjiva D. Kholkute. 2016. “Determination of Betulinic Acid, Oleanolic Acid and Ursolic Acid from Achyranthes Aspera L. Using RP-UFLC-DAD Analysis and Evaluation of Various Parameters for Their Optimum Yield.” Indian journal of experimental biology 54(3):196–202.
647. Pailee, Phanruethai, Vilailak Prachyawarakorn, Somsak Ruchirawat, and Chulabhorn Mahidol. 2015. “Bioactive Cardinane Sesquiterpenes from the Stems of Alangium Salviifolium.” Chemistry, an Asian journal 10(4):910–14.
648. Pakrashi, A. and P. Pakrasi. 1978. “Biological Profile of P-Coumaric Acid Isolated from Aristolochia Indica Linn.” Indian journal of experimental biology 16(12):1285–87.
649. Pakrashi, A. and C. Shaha. 1977. “Effect of a Sesquiterpene from Aristolochia Indica Linn. on Fertility in Female Mice.” Experientia 33(11):1498–99.
650. Pal, Atanu et al. 2012. “Phyto-Chemical Evaluation of Dried Aqueous Extract of Jivanti [Leptadenia Reticulata (Retz.) Wt. et Arn].” Ayu 33(4):557–60.
651. Pal, B. C., B. Achari, K. Yoshikawa, and S. Arihara. 1995. “Saponins from Albizia Lebbeck.” Phytochemistry 38(5):1287–91.
652. Pal, B. C., T. Chaudhuri, K. Yoshikawa, and S. Arihara. 1994. “Saponins from Barringtonia Acutangula.” Phytochemistry 35(5):1315–18.
653. Palbag, Satadru, Bijay Kr Dey, and Narendra Kumar Singh. 2014. “Ethnopharmacology, Phytochemistry and Pharmacology of Tephrosia Purpurea.” Chinese journal of natural medicines 12(1):1–7.
654. Palter, Rhoda and R. E. Lundin. 1970. “A Bitter Principle of Safflower; Matairesinol Monoglucoside.” Phytochemistry 9(11):2407–9.
655. Pan, Ping, Ling-Yun Jia, and Qi-Shi Sun. 2008. “[RP-HPLC determination of betulinic acid in Callicarpa macrophylla].” Zhongguo Zhong yao za zhi = Zhongguo zhongyao zazhi = China journal of Chinese materia medica 33(7):753–55.
656. Pan, Zhiqiang et al. 2016. “Alstoscholarisines H-J, Indole Alkaloids from Alstonia Scholaris: Structural Evaluation and Bioinspired Synthesis of Alstoscholarisine H.” Organic letters 18(4):654–57.
657. Panda, S., M. Jafri, A. Kar, and B. K. Meheta. 2009. “Thyroid Inhibitory, Antiperoxidative and Hypoglycemic Effects of Stigmasterol Isolated from Butea Monosperma.” Fitoterapia 80(2):123–26.
658. Panda, Sunanda and Anand Kar. 2009. “Periplogenin-3-O- -D-Glucopyranosyl -(1-->6)- -D-Glucopyaranosyl- -(1-->4) -D-Cymaropyranoside, Isolated from Aegle Marmelos Protects Doxorubicin Induced Cardiovascular Problems and Hepatotoxicity in Rats.” Cardiovascular therapeutics 27(2):108–16.
659. Pande, Monu, Vikash K. Dubey, Subhash C. Yadav, and Medicherla V Jagannadham. 2006. “A Novel Serine Protease Cryptolepain from Cryptolepis Buchanani: Purification and Biochemical Characterization.” Journal of agricultural and food chemistry 54(26):10141–50.
660. Pandey, Anima and S. P. Bhatnagar. 2009. “Preliminary Phytochemical Screening and Antimicrobial Studies on Artocarpus Lakoocha Roxb.” Ancient science of life 28(4):21–24.
661. Pandey, Arti and Pradeep Singh Negi. 2016. “Traditional Uses, Phytochemistry and Pharmacological Properties of Neolamarckia Cadamba: A Review.” Journal of ethnopharmacology 181:118–35.
662. Pandhare, Ramdas B., B. Sangameswaran, Popat B. Mohite, and Shantaram G. Khanage. 2011. “Antidiabetic Activity of Aqueous Leaves Extract of Sesbania Sesban (L) Merr. in Streptozotocin Induced Diabetic Rats.” Avicenna journal of medical biotechnology 3(1):37–43.
663. Pang, Xu et al. 2015. “New Polyoxypregnane Glycosides from the Roots of Marsdenia Tenacissima.” Steroids 93:68–76.
664. Pani, Saumya R., Satyaranjan Mishra, Sabuj Sahoo, and Prasana K. Panda. 2011. “Nephroprotective Effect of Bauhinia Variegata (Linn.) Whole Stem Extract against Cisplatin-Induced Nephropathy in Rats.” Indian journal of pharmacology 43(2):200–202.
665. Panigrahi, Ghanshyam, Chhayakanta Panda, and Arjun Patra. 2016. “Extract of Sesbania Grandiflora Ameliorates Hyperglycemia in High Fat Diet-Streptozotocin Induced Experimental Diabetes Mellitus.” Scientifica 2016:4083568.
666. Pant, Dipak Raj, Narayan Dutt Pant, Dil Bahadur Saru, Uday Narayan Yadav, and Dharma Prasad Khanal. 2017. “Phytochemical Screening and Study of Antioxidant, Antimicrobial, Antidiabetic, Anti-Inflammatory and Analgesic Activities of Extracts from Stem Wood of Pterocarpus Marsupium Roxburgh.” Journal of intercultural ethnopharmacology 6(2):170–76.
667. Park, Hye-Jin. 2014. “Immune Stimulatory Activity of BRP-4, an Acidic Polysaccharide from an Edible Plant, Basella Rubra L.” Asian Pacific journal of tropical medicine 7(11):849–53.
668. Parkash, O., D. Kumar, and S. Kumar. 2015. “Screening of Methanol Extract and Ethyl Acetate Fraction of Abies Webbiana Lindl. for Neuropharmacological Activities.” Indian journal of pharmaceutical sciences 77(5):536–41.
669. Patel, Jagruti J., Sanjeev R. Acharya, and Niyati S. Acharya. 2014. “Clerodendrum Serratum (L.) Moon. - a Review on Traditional Uses, Phytochemistry and Pharmacological Activities.” Journal of ethnopharmacology 154(2):268–85.
670. Patel, M. B. and S. H. Mishra. 2011. “Hypoglycemic Activity of C-Glycosyl Flavonoid from Enicostemma Hyssopifolium.” Pharmaceutical biology 49(4):383–91.
671. Patel, Maulik R. et al. 2013. “Study on the Mechanism of the Bronchodilatory Effects of Cynodon Dactylon (Linn.) and Identification of the Active Ingredient.” Journal of ethnopharmacology.
672. Patel, Satish, Vikas Sharma, Nagendra S. Chauhan, and Vinod K. Dixit. 2012. “An Updated Review on the Parasitic Herb of Cuscuta Reflexa Roxb.” Zhong xi yi jie he xue bao = Journal of Chinese integrative medicine 10(3):249–55.
673. Pathomwichaiwat, Thanika et al. 2015. “Alkaline Phosphatase Activity-Guided Isolation of Active Compounds and New Dammarane-Type Triterpenes from Cissus Quadrangularis Hexane Extract.” Journal of ethnopharmacology 160:52–60.
674. Patra, Amarendra, Sumana Ghosh, and Biswapati Mukherjee. 2010. “Structure Elucidation of Two New Bisbenzylisoquinoline Alkaloids and NMR Assignments of the Alkaloids from the Fruits of Tiliacora Racemosa.” Magnetic resonance in chemistry : MRC 48(10):823–28.
675. Patro, Ganesh, Subrat Kumar Bhattamisra, Bijay Kumar Mohanty, and Himanshu Bhusan Sahoo. 2016. “In Vitro and In Vivo Antioxidant Evaluation and Estimation of Total Phenolic, Flavonoidal Content of Mimosa Pudica L.” Pharmacognosy research 8(1):22–28.
676. Pattnaik, Ashok et al. 2013. “Pharmacological Studies on Buchanania Lanzan Spreng.- a Focus on Wound Healing with Particular Reference to Anti-Biofilm Properties.” Asian Pacific journal of tropical biomedicine 3(12):967–74.
677. Paul, Atish T., Sanjay Vir, and K. K. Bhutani. 2008. “Liquid Chromatography-Mass Spectrometry-Based Quantification of Steroidal Glycoalkaloids from Solanum Xanthocarpum and Effect of Different Extraction Methods on Their Content.” Journal of chromatography. A 1208(1–2):141–46.
678. Pawar, A. T., R. M. Anap, J. V Ghodasara, and B. S. Kuchekar. 2011. “Protective Effect of Hydroalcoholic Root Extract of Rubia Cordifolia in Indomethacin-Induced Enterocolitis in Rats.” Indian journal of pharmaceutical sciences 73(2):250–53.
679. Pawar, Rahul S. and K. K. Bhutani. 2004. “Madhucosides A and B, Protobassic Acid Glycosides from Madhuca Indica with Inhibitory Activity on Free Radical Release from Phagocytes.” Journal of natural products 67(4):668–71.
680. Pedroso, Marcio P., Ernesto C. Ferreira, Leandro W. Hantao, Stanislau Jr Bogusz, and Fabio Augusto. 2011. “Identification of Volatiles from Pineapple (Ananas Comosus L.) Pulp by Comprehensive Two-Dimensional Gas Chromatography and Gas Chromatography/mass Spectrometry.” Journal of separation science 34(13):1547–54.
681. Peng, Wei et al. 2015. “Areca Catechu L. (Arecaceae): A Review of Its Traditional Uses, Botany, Phytochemistry, Pharmacology and Toxicology.” Journal of ethnopharmacology 164:340–56.
682. Peraza-Sanchez, Sergio R. et al. 2002. “Constituents of the Leaves and Twigs of Ficus Hispida.” Planta medica 68(2):186–88.
683. Pettit, G. R. et al. 2000. “Isolation and Structures of Schleicherastatins 1-7 and Schleicheols 1 and 2 from the Teak Forest Medicinal Tree Schleichera Oleosa.” Journal of natural products 63(1):72–78.
684. Pinto, Luciano S. et al. 2008. “Purification and Molecular Cloning of a New Galactose-Specific Lectin from Bauhinia Variegata Seeds.” Journal of biosciences 33(3):355–63.
685. Pitsikas, Nikolaos and Petros A. Tarantilis. 2017. “Crocins, the Active Constituents of Crocus Sativus L., Counteracted Apomorphine-Induced Performance Deficits in the Novel Object Recognition Task, but Not Novel Object Location Task, in Rats.” Neuroscience letters 644:37–42.
686. Poonia, Amrita and Ashutosh Upadhayay. 2015. “Chenopodium Album Linn: Review of Nutritive Value and Biological Properties.” Journal of food science and technology 52(7):3977–85.
687. Potdar, Dipti, R. R. Hirwani, and Sivakami Dhulap. 2012. “Phyto-Chemical and Pharmacological Applications of Berberis Aristata.” Fitoterapia 83(5):817–30.
688. Pradeep, Hulikere Ananth et al. 2009. “Hepatoprotective Evaluation of Anogeissus Latifolia: In Vitro and in Vivo Studies.” World journal of gastroenterology 15(38):4816–22.
689. Prajapati, ND. 2003. A Handbook of Medicinal Plants. Agrobois P.
690. Prakasa Sastry, C. S. and L. Ramachandra Row. 1967. “New Triterpenes from Barringtonia Acutangula gaertn—III: The Constitution of Tanginol, a New Hexahydroxy Triterpene.” Tetrahedron 23(9):3837–46. Retrieved (http://www.sciencedirect.com/science/article/pii/0040402067800310).
691. Prakash, Tigari, Dupadahalli Kotresha, and Rama Rao Nedendla. 2011. “Neuroprotective Activity of Wedelia Calendulacea on Cerebral Ischemia/reperfusion Induced Oxidative Stress in Rats.” Indian journal of pharmacology 43(6):676–82.
692. Prashar, Yash, N. S. Gill, and Amber Perween. 2014. “AN UPDATED REVIEW ON MEDICINAL PROPERTIES OF Lagenaria Siceraria.” International Journal of Universal Pharmacy and Bio Sciences 3(4):362–76.
693. Premalatha, B. 2000. “Semecarpus Anacardium Linn. Nuts--a Boon in Alternative Medicine.” Indian journal of experimental biology 38(12):1177–82.
694. Priya, K. and S. Krishnakumari. 2007. “Phytochemical Analysis of Achyranthes Aspera and Its Activity on Sesame Oil Induced Lipid Peroxidation.” Ancient science of life 27(1):6–10.
695. Prudente, Arthur S. et al. 2013. “Pre-Clinical Anti-Inflammatory Aspects of a Cuisine and Medicinal Millennial Herb: Malva Sylvestris L.” Food and chemical toxicology : an international journal published for the British Industrial Biological Research Association 58:324–31.
696. Punitha, I. S. R., K. Rajendran, Arun Shirwaikar, and Annie Shirwaikar. 2005. “Alcoholic Stem Extract of Coscinium Fenestratum Regulates Carbohydrate Metabolism and Improves Antioxidant Status in Streptozotocin-Nicotinamide Induced Diabetic Rats.” Evidence-based complementary and alternative medicine : eCAM 2(3):375–81.
697. Puntumchai, Apirak et al. 2004. “Lakoochins A and B, New Antimycobacterial Stilbene Derivatives from Artocarpus Lakoocha.” Journal of natural products 67(3):485–86.
698. Qiao, Di, Li-She Gan, Jian-Xia Mo, and Chang-Xin Zhou. 2012. “[Chemical constituents of Acorus calamus].” Zhongguo Zhong yao za zhi = Zhongguo zhongyao zazhi = China journal of Chinese materia medica 37(22):3430–33.
699. Qureshi, Ahmad Kaleem et al. 2011. “Neolamarckines A and B, New Indole Alkaloids from Neolamarckia Cadamba.” Chemical & pharmaceutical bulletin 59(2):291–93.
700. Qureshi, Huma, Saira Asif, Haroon Ahmed, Hassan A. Al-Kahtani, and Khizar Hayat. 2016. “Chemical Composition and Medicinal Significance of Fagonia Cretica: A Review.” Natural product research 30(6):625–39.
701. Radchatawedchakoon, Widchaya et al. 2015. “A New 3’-Prenyloxypsoralen from the Raw Fruits of Aegle Marmelos and Its Cytotoxic Activity.” Natural product communications 10(11):1973–75.
702. Ragasa, Consolacion Y., Noemi Tremor, and John A. Rideout. 2002. “Ionone Derivatives from Alternanthera Sessilis.” Journal of Asian natural products research 4(2):109–15.
703. Raghavendra, S., V. Kumar, C. K. Ramesh, and M. H.Moinuddin Khan. 2012. “Enhanced Production of L-DOPA in Cell Cultures of Mucuna Pruriens L. and Mucuna Prurita H.” Natural product research 26(9):792–801.
704. Rahman, M.Mukhlesur, Simon Gibbons, and Alexander I. Gray. 2007. “Isoflavanones from Uraria Picta and Their Antimicrobial Activity.” Phytochemistry 68(12):1692–97.
705. Rahman, Md Mukhlesur and Alexander I. Gray. 2002. “Antimicrobial Constituents from the Stem Bark of Feronia Limonia.” Phytochemistry 59(1):73–77.
706. Rai, Prashant Kumar, Sharad Kumar Gupta, Amrita Kumari Srivastava, Rajesh Kumar Gupta, and Geeta Watal. 2013. “A Scientific Validation of Antihyperglycemic and Antihyperlipidemic Attributes of Trichosanthes Dioica.” ISRN pharmacology 2013:473059.
707. Raina, Archana P. and K. S. Negi. 2015. “Essential Oil Composition of Valeriana Jatamansi Jones from Himalayan Regions of India.” Indian journal of pharmaceutical sciences 77(2):218–22.
708. Raja, Subramaniya Bharathi, Malliga Raman Murali, Nirmal Kasinathan Kumar, and Sivasitambaram Niranjali Devaraj. 2011. “Isolation and Partial Characterisation of a Novel Lectin from Aegle Marmelos Fruit and Its Effect on Adherence and Invasion of Shigellae to HT29 Cells.” PloS one 6(1):e16231.
709. Rajagopal Rao, D., K. N. Gurudutt, S. Mamatha, and L. J. Mohan Rao. 2007. “Guttiferic Acid, a Novel Rearrangement Product from Minor Chromenoxanthone Pigments of Garcinia Morella Desr.” Magnetic resonance in chemistry : MRC 45(7):578–82.
710. Rajakrishnan, R. et al. 2017. “Phytochemical Evaluation of Roots of Plumbago Zeylanica L. and Assessment of Its Potential as a Nephroprotective Agent.” Saudi journal of biological sciences 24(4):760–66.
711. Rajashekharan, S. et al. 1989. “ETHNO-MEDICO-BOT ANICAL STUDIES OF CHERIYA ARAYAN-AND VALIYA ARAYAN- (Aristolochia Indica, Linn; Aristolochia Tagala, Cham).” Ancient science of life 9(2):99–106.
712. Rajavel, Tamilselvam, Ramar Mohankumar, Govindaraju Archunan, Kandasamy Ruckmani, and Kasi Pandima Devi. 2017. “Beta Sitosterol and Daucosterol (Phytosterols Identified in Grewia Tiliaefolia) Perturbs Cell Cycle and Induces Apoptotic Cell Death in A549 Cells.” Scientific reports 7(1):3418.
713. Rajesh, P., S. Latha, P. Selvamani, and V.Rajesh Kannan. 2009. “Phytochemical Screening and Toxicity Studies on the Leaves of Capparis Sepiaria Linn. (Capparidaceae).” Journal of basic and clinical pharmacy 1(1):41–46.
714. Rajkapoor, Balasubramanian, Narayanan Murugesh, and Devarakonda Rama Krishna. 2009. “Cytotoxic Activity of a Flavanone from the Stem of Bauhinia Variegata Linn.” Natural product research 23(15):1384–89.
715. Ramachandran, Shakila. 2013. “Review on Sphaeranthus Indicus Linn. (Kottaikkarantai).” Pharmacognosy reviews 7(14):157–69.
716. Ramadan, Mohamed Fawzy, Adel Abdelrazek Abdelazim Mohdaly, Adel M. A. Assiri, Monier Tadros, and Bernd Niemeyer. 2016. “Functional Characteristics, Nutritional Value and Industrial Applications of Madhuca Longifolia Seeds: An Overview.” Journal of food science and technology 53(5):2149–57.
717. Ramesh, B. N. et al. 2014. “Comparative Study on Anti-Oxidant and Anti-Inflammatory Activities of Caesalpinia Crista and Centella Asiatica Leaf Extracts.” Journal of pharmacy & bioallied sciences 6(2):86–91.
718. Ramesh, T., C. Sureka, S. Bhuvana, and V. Hazeena Begum. 2010. “Sesbania Grandiflora Diminishes Oxidative Stress and Ameliorates Antioxidant Capacity in Liver and Kidney of Rats Exposed to Cigarette Smoke.” Journal of physiology and pharmacology : an official journal of the Polish Physiological Society 61(4):467–76.
719. Ramos, Eliezer L. P. et al. 2016. “Lectins from Synadenium Carinatum (ScLL) and Artocarpus Heterophyllus (ArtinM) Are Able to Induce Beneficial Immunomodulatory Effects in a Murine Model for Treatment of Toxoplasma Gondii Infection.” Frontiers in cellular and infection microbiology 6:164.
720. Rana, Vikas, Vineet Kumar, and P. L. Soni. 2012. “Structural Characterization of an Acidic Polysaccharide from Dalbergia Sissoo Roxb. Leaves.” Carbohydrate polymers 90(1):243–50.
721. Ranasinghe, Priyanga et al. 2013. “Medicinal Properties of ‘True’ Cinnamon (Cinnamomum Zeylanicum): A Systematic Review.” BMC complementary and alternative medicine 13:275.
722. Rao, K. V and P. L. Rao. 1967. “Antibiotic Principles of Garcinia Morella: IX. Antimicrobial Activity of Alpha- & Beta-Guttiferins & Their Derivatives.” Indian journal of experimental biology 5(2):101–5.
723. Rao, Lingamallu Jagan Mohan, Hiroshi Yada, Hiroshi Ono, Mayumi Ohnishi-Kameyama, and Mitsuru Yoshida. 2004. “Occurrence of Antioxidant and Radical Scavenging Proanthocyanidins from the Indian Minor Spice Nagkesar (Mammea Longifolia Planch and Triana Syn).” Bioorganic & medicinal chemistry 12(1):31–36.
724. Rao, Meneni S., Helmut Duddeck, and Roman Dembinski. 2002. “Isolation and Structural Elucidation of 3,4’,5,7-Tetraacetyl Quercetin from Adina Cordifolia (Karam Ki Gaach).” Fitoterapia 73(4):353–55.
725. Rao, R.Jagdeeshwar, U.Sampath Kumar, S.Venkat Reddy, Ashok K. Tiwari, and J.Madhusudana Rao. 2005. “Antioxidants and a New Germacrane Sesquiteroene from Carissa Spinarum.” Natural product research 19(8):763–69.
726. Rao, Yerra Koteswara, Shih-Hua Fang, and Yew-Min Tzeng. 2008. “Antiinflammatory Activities of Flavonoids and a Triterpene Caffeate Isolated from Bauhinia Variegata.” Phytotherapy research : PTR 22(7):957–62.
727. Rasheed, Zafar, Nahid Akhtar, Abubakar Khan, Khursheed A. Khan, and Tariq M. Haqqi. 2010. “Butrin, Isobutrin, and Butein from Medicinal Plant Butea Monosperma Selectively Inhibit Nuclear Factor-kappaB in Activated Human Mast Cells: Suppression of Tumor Necrosis Factor-Alpha, Interleukin (IL)-6, and IL-8.” The Journal of pharmacology and experimental therapeutics 333(2):354–63.
728. Rathee, Permender, Dharmender Rathee, Deepti Rathee, and Sushila Rathee. 2012. “In-Vitro Cytotoxic Activity of Beta-Sitosterol Triacontenate Isolated from Capparis Decidua (Forsk.) Edgew.” Asian Pacific journal of tropical medicine 5(3):225–30.
729. Rathi, Bhawana, Juhi Sahu, Sameksha Koul, and R. L. Kosha. 2013. “Detailed Pharmacognostical Studies on Berberis Aristata DC Plant.” Ancient science of life 32(4):234–40.
730. Ravishankara, M. N., N. Shrivastava, H. Padh, and M. Rajani. 2001. “HPTLC Method for the Estimation of Alkaloids of Cinchona Officinalis Stem Bark and Its Marketed Formulations.” Planta medica 67(3):294–96.
731. Razavi, Seyed Mohammad Ali, Steve W. Cui, and Huihuang Ding. 2016. “Structural and Physicochemical Characteristics of a Novel Water-Soluble Gum from Lallemantia Royleana Seed.” International journal of biological macromolecules 83:142–51.
732. Reanmongkol, Wantana, Tassanee Noppapan, and Sanan Subhadhirasakul. 2009. “Antinociceptive, Antipyretic, and Anti-Inflammatory Activities of Putranjiva Roxburghii Wall. Leaf Extract in Experimental Animals.” Journal of natural medicines 63(3):290–96.
733. Reddy, G. C., S. Rangaswami, and R. Sunder. 1977. “Triterpenoids of the Stem Bark of Gardenia Gummifera.” Planta medica 32(3):206–11.
734. Reddy, Mopuru V. B., Muntha K. Reddy, Duvvuru Gunasekar, Cristelle Caux, and Bernard Bodo. 2003. “A Flavanone and a Dihydrodibenzoxepin from Bauhinia Variegata.” Phytochemistry 64(4):879–82.
735. Reddy, P.Prabhakar et al. 2009. “Two New Cytotoxic Diterpenes from the Rhizomes of Hedychium Spicatum.” Bioorganic & medicinal chemistry letters 19(1):192–95.
736. Reddy, V. L., V. Ravikanth, T. P. Rao, P. V Diwan, and Y. Venkateswarlu. 2001. “A New Triterpenoid from the Fern Adiantum Lunulatum and Evaluation of Antibacterial Activity.” Phytochemistry 56(2):173–75.
737. Rekha, K. et al. 2013. “Two New Sesquiterpenoids from the Rhizomes of Nardostachys Jatamansi.” Journal of Asian natural products research 15(2):111–16.
738. Ren, Da-Bing et al. 2014. “Separation of Nine Compounds from Salvia Plebeia R.Br. Using Two-Step High-Speed Counter-Current Chromatography with Different Elution Modes.” Journal of separation science 37(16):2118–25.
739. Ren, Gang et al. 2015. “Structure Elucidation and NMR Assignments of Two New Flavanones from the Roots of Artocarpus Heterophyllus.” Magnetic resonance in chemistry : MRC 53(10):872–74.
740. Reshma, M. V et al. 2017. “First Report on Isolation of 2,3,4-Trihydroxy-5-Methylacetophenone from Palmyra Palm (Borassus Flabellifer Linn.) Syrup, Its Antioxidant and Antimicrobial Properties.” Food chemistry 228:491–96.
741. Revesz, L. et al. 1999. “Isolation and Synthesis of a Novel Immunosuppressive 17alpha-Substituted Dammarane from the Flour of the Palmyrah Palm (Borassus Flabellifer).” Bioorganic & medicinal chemistry letters 9(11):1521–26.
742. Reza, H. M. et al. 2014. “Phytochemical and Pharmacological Investigation of Ethanol Extract of Cissampelos Pareira.” Indian journal of pharmaceutical sciences 76(5):455–58.
743. Rezaeinodehi, A. and S. Khangholi. 2008. “Chemical Composition of the Essential Oil of Artemisia Absinthium Growing Wild in Iran.” Pakistan journal of biological sciences : PJBS 11(6):946–49.
744. Riaz, Naheed, Itrat Anis, Aziz-ur-Rehman, et al. 2003. “Emodinol, Beta-Glucuronidase Inhibiting Triterpene from Paeonia Emodi.” Natural product research 17(4):247–51.
745. Riaz, Naheed, Itrat Anis, Abdul Malik, et al. 2003. “Paeonins A and B, Lipoxygenase Inhibiting Monoterpene Galactosides from Paeonia Emodi.” Chemical & pharmaceutical bulletin 51(3):252–54.
746. Riaz, Naheed et al. 2004. “Lipoxygenase Inhibiting and Antioxidant Oligostilbene and Monoterpene Galactoside from Paeonia Emodi.” Phytochemistry 65(8):1129–35.
747. Rizvi, S. A. and O. C. Saxena. 1974. “New Glycosides, Terpenoids, Colouring Matters, Sugars and Fatty Compounds from the Flowers of Salmalia Malabarica.” Arzneimittel-Forschung 24(3):285–87.
748. Rodriguez Villanueva, Javier and Laura Rodriguez Villanueva. 2017. “Experimental and Clinical Pharmacology of Ziziphus Jujuba Mills.” Phytotherapy research : PTR 31(3):347–65.
749. Roopan, Selvaraj Mohana. 2016. “An Overview of Phytoconstituents, Biotechnological Applications, and Nutritive Aspects of Coconut (Cocos Nucifera).” Applied biochemistry and biotechnology 179(8):1309–24.
750. Row, Lie-Ching, Jiau-Ching Ho, and Chiu-Ming Chen. 2007. “Cerebrosides and Tocopherol Trimers from the Seeds of Euryale Ferox.” Journal of natural products 70(7):1214–17.
751. Roy, S. K., M. Ali, M. P. Sharma, and R. Ramachandram. 2001. “New Pentacyclic Triterpenes from the Roots of Hemidesmus Indicus.” Die Pharmazie 56(3):244–46.
752. Roy, Somendu K. et al. 2013. “NorA Efflux Pump Inhibitory Activity of Coumarins from Mesua Ferrea.” Fitoterapia 90:140–50.
753. Roy, Somendu K., Sonika Pahwa, Hemraj Nandanwar, and Sanjay M. Jachak. 2012. “Phenylpropanoids of Alpinia Galanga as Efflux Pump Inhibitors in Mycobacterium Smegmatis mc(2) 155.” Fitoterapia 83(7):1248–55.
754. Rubio-Pina, Jorge and Felipe Vazquez-Flota. 2013. “Pharmaceutical Applications of the Benzylisoquinoline Alkaloids from Argemone Mexicana L.” Current topics in medicinal chemistry 13(17):2200–2207.
755. Rukachaisirikul, Thitima, Samran Prabpai, Penprapa Champung, and Apichart Suksamrarn. 2002. “Chabamide, a Novel Piperine Dimer from Stems of Piper Chaba.” Planta medica 68(9):853–55.
756. Sabale, Vidya, Harish Kunjwani, and Prafulla Sabale. 2011. “Formulation and in Vitro Evaluation of the Topical Antiageing Preparation of the Fruit of Benincasa Hispida.” Journal of Ayurveda and integrative medicine 2(3):124–28.
757. Saboo, Shweta S., Ganesh G. Tapadiya, Jasvant J. Lamale, and Somshekhar S. Khadabadi. 2014. “Phytochemical Screening and Antioxidant, Antimitotic, and Antiproliferative Activities of Trichodesma Indicum Shoot.” Ancient science of life 34(2):113–18.
758. Sadat, Anwar, Ghias Uddin, M. Alam, Ashfaq Ahmad, and Bina Shaheen Siddiqui. 2015. “Structure Activity Relationship of Bergenin, P-Hydroxybenzoyl Bergenin, 11-O-Galloylbergenin as Potent Antioxidant and Urease Inhibitor Isolated from Bergenia Ligulata.” Natural product research 29(24):2291–94.
759. Safitri, Ratu, Ponis Tarigan, Hans Joachim Freisleben, Rymond J. Rumampuk, and Akira Murakami. 2003. “Antioxidant Activity in Vitro of Two Aromatic Compounds from Caesalpinia Sappan L.” BioFactors (Oxford, England) 19(1–2):71–77.
760. Sage, H. J. and S. L. Connett. 1969. “Studies on a Hemagglutinin from the Meadow Mushroom. II. Purification, Composition, and Structure of Agaricus Campestris Hemagglutinin.” The Journal of biological chemistry 244(17):4713–19.
761. Sahoo, Himanshu Bhusan, Amrita Bhaiji, and Dev Das Santani. 2016. “Lactogenic Activity of Teramnus Labialis (Linn.) Fruit with Special Reference to the Estimation of Serum Prolactin and Cortisol Level in Nursing Rats.” Indian journal of pharmacology 48(6):715–19.
762. Sahu, Niranjan P.; Koike, Kazuo; Jia, Zhonghua; Banerjee, Sukdeb; Mandal, Nirup B.; Nikaido, Tamotsu. 2000. “Triterpene Glycosides from the Bark of Anthocephalus Cadamba.” Journal of Chemical Research 22–23.
763. Sahu, Jyoti, K. M. Koley, and B. D. Sahu. 2017. “Attribution of Antibacterial and Antioxidant Activity of Cassia Tora Extract toward Its Growth Promoting Effect in Broiler Birds.” Veterinary world 10(2):221–26.
764. Sahu, Niranjan P. et al. 1999. “Structures of Two Novel Isomeric Triterpenoid Saponins from Anthocephalus Cadamba.” Magn. Reson Chem 37:837–42.
765. Sahu, Ranabir et al. 2012. “Dereplication Coupled with in Vitro Antioxidant Assay of Two Flavonoid Glycosides from Diospyros Peregrina Fruit.” Natural product research 26(5):454–59.
766. Said, Ataa et al. 2010. “In Vitro Antioxidant and Antiproliferative Activities of Flavonoids from Ailanthus Excelsa (Roxb.) (Simaroubaceae) Leaves.” Zeitschrift fur Naturforschung. C, Journal of biosciences 65(3–4):180–86.
767. Saijyo, Junichi et al. 2008. “Alpha-Glucosidase Inhibitor from Bergenia Ligulata.” Journal of oleo science 57(8):431–35.
768. Saikia, Sangeeta, Nikhil Kumar Mahnot, and Charu Lata Mahanta. 2015. “Optimisation of Phenolic Extraction from Averrhoa Carambola Pomace by Response Surface Methodology and Its Microencapsulation by Spray and Freeze Drying.” Food chemistry 171:144–52.
769. Saima, Y., A. K. Das, K. K. Sarkar, A. K. Sen, and P. Sur. 2000. “An Antitumor Pectic Polysaccharide from Feronia Limonia.” International journal of biological macromolecules 27(5):333–35.
770. Sain, Soumyadeep et al. 2014. “Beta Caryophyllene and Caryophyllene Oxide, Isolated from Aegle Marmelos, as the Potent Anti-Inflammatory Agents against Lymphoma and Neuroblastoma Cells.” Anti-inflammatory & anti-allergy agents in medicinal chemistry 13(1):45–55.
771. Sajeli, Begum et al. 2006. “Hyosgerin, a New Optically Active Coumarinolignan, from the Seeds of Hyoscyamus Niger.” Chemical & pharmaceutical bulletin 54(4):538–41.
772. Sakai, Eiji, Tsuyoshi Katayama, Takeshi Ogasawara, and Mizuo Mizuno. 2013. “Identification of Anogeissus Latifolia Wallich and Analysis of Refined Gum Ghatti.” Journal of natural medicines 67(2):276–80.
773. Sakran, Mohamed, Yasser Selim, and Nahla Zidan. 2014. “A New Isoflavonoid from Seeds of Lepidium Sativum L. and Its Protective Effect on Hepatotoxicity Induced by Paracetamol in Male Rats.” Molecules (Basel, Switzerland) 19(10):15440–51.
774. Salami, Maryam, Mehdi Rahimmalek, and Mohammad Hossein Ehtemam. 2017. “Comprehensive Research on Essential Oil and Phenolic Variation in Different Foeniculum Vulgare Populations During Transition from Vegetative to Reproductive Stage.” Chemistry & biodiversity 14(2).
775. Saleem, Muhammad, Haq Nawaz Bhatti, Muhammad Idrees Jilani, and Muhammad Asif Hanif. 2015. “Bioanalytical Evaluation of Cinnamomum Zeylanicum Essential Oil.” Natural product research 29(19):1857–59.
776. Saleem, R. et al. 1999. “Hypotensive, Hypoglycaemic and Toxicological Studies on the Flavonol C-Glycoside Shamimin from Bombax Ceiba.” Planta medica 65(4):331–34.
777. Saleem, Rubeena et al. 2003. “Hypotensive Activity and Toxicology of Constituents from Bombax Ceiba Stem Bark.” Biological & pharmaceutical bulletin 26(1):41–46.
778. Saleh-E-In, Md Moshfekus et al. 2017. “Chemical Composition and Pharmacological Significance of Anethum Sowa L. Root.” BMC complementary and alternative medicine 17(1):127.
779. Salib, Josline Y. et al. 2014. “New Quinoline Alkaloid from Ruta Graveolens Aerial Parts and Evaluation of the Antifertility Activity.” Natural product research 28(17):1335–42.
780. Sampaio, Karina L., Deborah S. Garruti, Maria Regina B. Franco, Natalia S. Janzantti, and Maria Aparecida Ap Da Silva. 2011. “Aroma Volatiles Recovered in the Water Phase of Cashew Apple (Anacardium Occidentale L.) Juice during Concentration.” Journal of the science of food and agriculture 91(10):1801–9.
781. Sanadi, A. R. and A. Surolia. 1994. “Studies on a Chitooligosaccharide-Specific Lectin from Coccinia Indica. Thermodynamics and Kinetics of Umbelliferyl Glycoside Binding.” The Journal of biological chemistry 269(7):5072–77.
782. Sanchez, E., N. Heredia, M.Del R. Camacho-Corona, and S. Garcia. 2013. “Isolation, Characterization and Mode of Antimicrobial Action against Vibrio Cholerae of Methyl Gallate Isolated from Acacia Farnesiana.” Journal of applied microbiology 115(6):1307–16.
783. Sanei-Dehkordi, Alireza, Mohammad Mehdi Sedaghat, Hassan Vatandoost, and Mohammad Reza Abai. 2016. “Chemical Compositions of the Peel Essential Oil of Citrus Aurantium and Its Natural Larvicidal Activity against the Malaria Vector Anopheles Stephensi (Diptera: Culicidae) in Comparison with Citrus Paradisi.” Journal of arthropod-borne diseases 10(4):577–85.
784. Sani, B. P. and P. L. Rao. 1966. “Antibiotic Principles of Garcinia Morella. VII. Antiprotozoal Activity of Morellin, Neomorellin & Other Insoluble Neutral Phenols of the Seed Coat of Garcinia Morella.” Indian journal of experimental biology 4(1):27–28.
785. Sanna, Giuseppina et al. 2015. “Limonoids from Melia Azedarach Fruits as Inhibitors of Flaviviruses and Mycobacterium Tubercolosis.” PloS one 10(10):e0141272.
786. Dos Santos, Delair Silveira et al. 2014. “Seasonal Phytochemical Study and Antimicrobial Potential of Vetiveria Zizanioides Roots.” Acta pharmaceutica (Zagreb, Croatia) 64(4):495–501.
787. Saraswathy, A., S.Nandini Devi, and D. Ramasamy. 2008. “Antioxidant, Heavy Metals and Elemental Analysis of Holoptelea Integrifolia Planch.” Indian journal of pharmaceutical sciences 70(5):576–683.
788. Saravanan, Nadana and Namasivayam Nalini. 2007. “Inhibitory Effect of Hemidesmus Indicus and Its Active Principle 2-Hydroxy 4-Methoxy Benzoic Acid on Ethanol-Induced Liver Injury.” Fundamental & clinical pharmacology 21(5):507–14.
789. Sarkar, S. K., G. Poddar, and S. B. Mahato. 1987. “Glucosides from Abies Webbiana.” Planta medica 53(2):219–20.
790. Sarma, Rahul, Sima Kumari, Ramakrishnan Elancheran, Meetali Deori, and Rajlakshmi Devi. 2016. “Polyphenol Rich Extract of Garcinia Pedunculata Fruit Attenuates the Hyperlipidemia Induced by High Fat Diet.” Frontiers in pharmacology 7:294.
791. Saroya, Amritpal Singh. 2011. Herbalism , Phytochemistry and Ethnopharmacology. Enfield USA: Science Publishers, CRC Press Taylor & Francis Group.
792. Sasidharan, Suja Rani et al. 2014. “Ameliorative Potential of Tamarindus Indica on High Fat Diet Induced Nonalcoholic Fatty Liver Disease in Rats.” TheScientificWorldJournal 2014:507197.
793. Satyal, Prabodh et al. 2013. “Chemical Compositions, Phytotoxicity, and Biological Activities of Acorus Calamus Essential Oils from Nepal.” Natural product communications 8(8):1179–81.
794. Satyal, Prabodh et al. 2015. “Chemical Composition of Blumea Lacera Essential Oil from Nepal. Biological Activities of the Essential Oil and (Z)-Lachnophyllum Ester.” Natural product communications 10(10):1749–50.
795. Satyal, Prabodh, Noura S. Dosoky, Brittany L. Kincer, and William N. Setzer. 2012. “Chemical Compositions and Biological Activities of Amomum Subulatum Essential Oils from Nepal.” Natural product communications 7(9):1233–36.
796. Schimming, T., K. Jenett-Siems, K. Siems, L. Witte, and E. Eich. 2005. “N1,N10-Ditigloylspermidine, a Novel Alkaloid from the Seeds of Ipomoea Nil.” Die Pharmazie 60(12):958–59.
797. Schweiggert, Ralf M. et al. 2016. “Carotenoids, Carotenoid Esters, and Anthocyanins of Yellow-, Orange-, and Red-Peeled Cashew Apples (Anacardium Occidentale L.).” Food chemistry 200:274–82.
798. Sebastiani, Bartolomeo, Martino Giorgini, and Stefano Falcinelli. 2017. “Chemical Characterization of Lodoicea Maldivica Fruit.” Chemistry & biodiversity 14(8).
799. Sehrawat, Anuradha and Vijay Kumar. 2012. “Butein Imparts Free Radical Scavenging, Anti-Oxidative and Proapoptotic Properties in the Flower Extracts of Butea Monosperma.” Biocell : official journal of the Sociedades Latinoamericanas de Microscopia Electronica ... et. al 36(2):63–71.
800. Sekhar, Shailasree, K. K. Sampath-Kumara, S. R. Niranjana, and H. S. Prakash. 2015. “Attenuation of Reactive Oxygen/nitrogen Species with Suppression of Inducible Nitric Oxide Synthase Expression in RAW 264.7 Macrophages by Bark Extract of Buchanania Lanzan.” Pharmacognosy magazine 11(42):283–91.
801. Selles, Chaouki et al. 2013. “Antimicrobial Activity and Evolution of the Composition of Essential Oil from Algerian Anacyclus Pyrethrum L. through the Vegetative Cycle.” Natural product research 27(23):2231–34.
802. Selvam, C. and Sanjay M. Jachak. 2004. “A Cyclooxygenase (COX) Inhibitory Biflavonoid from the Seeds of Semecarpus Anacardium.” Journal of ethnopharmacology 95(2–3):209–12.
803. Selvam, C., Sanjay M. Jachak, and K. K. Bhutani. 2004. “Cyclooxygenase Inhibitory Flavonoids from the Stem Bark of Semecarpus Anacardium Linn.” Phytotherapy research : PTR 18(7):582–84.
804. Semalty, Mona, Ajay Semalty, Ashutosh Badola, Geeta Pant Joshi, and M. S. M. Rawat. 2010. “Semecarpus Anacardium Linn.: A Review.” Pharmacognosy reviews 4(7):88–94.
805. Sendker, Jandirk et al. 2017. “Phytochemical Characterization of Low Molecular Weight Constituents from Marshmallow Roots (Althaea Officinalis) and Inhibiting Effects of the Aqueous Extract on Human Hyaluronidase-1.” Journal of natural products 80(2):290–97.
806. Sengupta, A. and S. K. Roychoudhury. 1977. “Triglyceride Composition of Buchanania Lanzan Seed Oil.” Journal of the science of food and agriculture 28(5):463–68.
807. Senthilkumar, A., M. Jayaraman, and V. Venkatesalu. 2013. “Chemical Constituents and Larvicidal Potential of Feronia Limonia Leaf Essential Oil against Anopheles Stephensi, Aedes Aegypti and Culex Quinquefasciatus.” Parasitology research 112(3):1337–42.
808. Septama, Abdi Wira and Pharkphoom Panichayupakaranant. 2015. “Antibacterial Assay-Guided Isolation of Active Compounds from Artocarpus Heterophyllus Heartwoods.” Pharmaceutical biology 53(11):1608–13.
809. Shafi, P. M., M. K. Rosamma, Kaiser Jamil, and P. S. Reddy. 2002. “Antibacterial Activity of the Essential Oil from Aristolochia Indica.” Fitoterapia 73(5):439–41.
810. Shah, Abdul Jabbar et al. 2011. “Studies on the Chemical Composition and Possible Mechanisms Underlying the Antispasmodic and Bronchodilatory Activities of the Essential Oil of Artemisia Maritima L.” Archives of pharmacal research 34(8):1227–38.
811. Shah, Naseer Ali et al. 2013. “Investigation on Flavonoid Composition and Anti Free Radical Potential of Sida Cordata.” BMC complementary and alternative medicine 13:276.
812. Shah, Naseer Ali, Muhammad Rashid Khan, and Dereje Nigussie. 2017. “Phytochemical Investigation and Nephroprotective Potential of Sida Cordata in Rat.” BMC complementary and alternative medicine 17(1):388.
813. Shaikh, Rahimullah and Imran Zainuddin Syed. 2015. “Proximate and Phytochemical Analysis of Cassia Tora Leaves.” Journal of Research in Pharmaceutical Science 2(8):2347–2995.
814. Shailendra Kumar, M. B. et al. 2010. “Screening of Selected Biological Activities of Artocarpus Lakoocha Roxb (Moraceae) Fruit Pericarp.” Journal of basic and clinical pharmacy 1(4):239–45.
815. Shalavadi, M. H., V. M. Chandrashekhar, S. P. Avinash, C. Sowmya, and A. Ramkishan. 2012. “Neuroprotective Activity of Stereospermum Suaveolens DC against 6-OHDA Induced Parkinson’s Disease Model.” Indian journal of pharmacology 44(6):737–43.
816. Shankar, Rama and M. S. Rawat. 2012. “Conservation of Traditional Medicinal Practices and Pharmaceutically.” The Journal of Ethnobiology and Traditional Medicine 117:178–88.
817. Shao, Bo, Hong-zhu Guo, et al. 2007. “Simultaneous Determination of Six Major Stilbenes and Flavonoids in Smilax China by High Performance Liquid Chromatography.” Journal of pharmaceutical and biomedical analysis 44(3):737–42.
818. Shao, Bo, Hongzhu Guo, et al. 2007. “Steroidal Saponins from Smilax China and Their Anti-Inflammatory Activities.” Phytochemistry 68(5):623–30.
819. Sharma, Bhesh Raj, Lekh Nath S. Gautam, Deepak Adhikari, and Rajendra Karki. 2017. “A Comprehensive Review on Chemical Profiling of Nelumbo Nucifera: Potential for Drug Development.” Phytotherapy research : PTR 31(1):3–26.
820. Sharma, S. C., R. Chand, B. S. Bhatti, and O. P. Sati. 1982. “New Oligospirostanosides and Oligofurostanosides from Asparagus Adscendens Roots.” Planta medica 46(1):48–51.
821. Sharma, Sunayana, Hirday Narain Verma, and Nilesh Kumar Sharma. 2014. “Cationic Bioactive Peptide from the Seeds of Benincasa Hispida.” International journal of peptides 2014:156060.
822. Sharma, Veena and Aastha Agarwal. 2015. “Physicochemical and Antioxidant Assays of Methanol and Hydromethanol Extract of Ariel Parts of Indigofera Tinctoria Linn.” Indian journal of pharmaceutical sciences 77(6):729–34.
823. Sharma, Vikas, Isha Singh, and Priyanka Chaudhary. 2014. “Acorus Calamus (The Healing Plant): A Review on Its Medicinal Potential, Micropropagation and Conservation.” Natural product research 28(18):1454–66.
824. Shen, Yuh-Chiang, Chi-Wen Juan, Che-San Lin, Chien-Chih Chen, and Chia-Lin Chang. 2017. “NEUROPROTECTIVE EFFECT OF TERMINALIA CHEBULA EXTRACTS AND ELLAGIC ACID IN PC12 CELLS.” African journal of traditional, complementary, and alternative medicines : AJTCAM 14(4):22–30.
825. Shewale, Pallavi B., Rupali A. Patil, and Yogesh A. Hiray. 2012. “Antidepressant-like Activity of Anthocyanidins from Hibiscus Rosa-Sinensis Flowers in Tail Suspension Test and Forced Swim Test.” Indian journal of pharmacology 44(4):454–57.
826. Shi, Xiaofeng et al. 2016. “Extraction and Purification of Total Flavonoids from Pine Needles of Cedrus Deodara Contribute to Anti-Tumor in Vitro.” BMC complementary and alternative medicine 16:245.
827. Shi, Zheng-Feng, Chun Lei, Bang-Wei Yu, He-Yao Wang, and Ai-Jun Hou. 2016. “New Alkaloids and Alpha-Glucosidase Inhibitory Flavonoids from Ficus Hispida.” Chemistry & biodiversity 13(4):445–50.
828. Shih, C. Y., A. A. Khan, S. Jia, J. Wu, and D. S. Shih. 2001. “Purification, Characterization, and Molecular Cloning of a Chitinase from the Seeds of Benincasa Hispida.” Bioscience, biotechnology, and biochemistry 65(3):501–9.
829. Shih, C. Y. T. et al. 2001. “Purification of an Osmotin-like Protein from the Seeds of Benincasa Hispida and Cloning of the Gene Encoding This Protein.” Plant science : an international journal of experimental plant biology 160(5):817–26.
830. Shimizu, N., M. Tomoda, I. Suzuki, and K. Takada. 1993. “Plant Mucilages. XLIII. A Representative Mucilage with Biological Activity from the Leaves of Hibiscus Rosa-Sinensis.” Biological & pharmaceutical bulletin 16(8):735–39.
831. Shinde, P. B., S. D. Katekhaye, M. B. Mulik, and K. S. Laddha. 2014. “Rapid Simultaneous Determination of Marmelosin, Umbelliferone and Scopoletin from Aegle Marmelos Fruit by RP-HPLC.” Journal of food science and technology 51(9):2251–55.
832. Shinde, Prashant B. and Kirti S. Laddha. 2015. “Simultaneous Quantification of Furanocoumarins from Aegle Marmelos Fruit Pulp Extract.” Journal of chromatographic science 53(4):576–79.
833. Shirole, R. L., N. L. Shirole, A. A. Kshatriya, R. Kulkarni, and M. N. Saraf. 2014. “Investigation into the Mechanism of Action of Essential Oil of Pistacia Integerrima for Its Antiasthmatic Activity.” Journal of ethnopharmacology 153(3):541–51.
834. Shrestha, Sabina et al. 2012. “Rhus Parviflora and Its Biflavonoid Constituent, Rhusflavone, Induce Sleep through the Positive Allosteric Modulation of GABA(A)-Benzodiazepine Receptors.” Journal of ethnopharmacology 142(1):213–20.
835. Shrestha, Sabina, Dae-Young Lee, et al. 2013. “Phenolic Components from Rhus Parviflora Fruits and Their Inhibitory Effects on Lipopolysaccharide-Induced Nitric Oxide Production in RAW 264.7 Macrophages.” Natural product research 27(23):2244–47.
836. Shrestha, Sabina, Sathishkumar Natarajan, et al. 2013. “Potential Neuroprotective Flavonoid-Based Inhibitors of CDK5/p25 from Rhus Parviflora.” Bioorganic & medicinal chemistry letters 23(18):5150–54.
837. Shrestha, Suraj Prakash, Yuri Amano, Yuji Narukawa, and Tadahiro Takeda. 2008. “Nitric Oxide Production Inhibitory Activity of Flavonoids Contained in Trunk Exudates of Dalbergia Sissoo.” Journal of natural products 71(1):98–101.
838. Shu, Shi-Hui, An-Jun Deng, Zhi-Hong Li, and Hai-Lin Qin. 2011. “Two Novel Biphenyl Dimers from the Heartwood of Caesalpinia Sappan.” Fitoterapia 82(5):762–66.
839. Shu, Shi-Hui, Jing-Lan Han, Guan-Hua Du, and Hai-Lin Qin. 2008. “[A new flavonoid from heartwood of Caesalpinia sappan].” Zhongguo Zhong yao za zhi = Zhongguo zhongyao zazhi = China journal of Chinese materia medica 33(8):903–5.
840. Shukla, Nivedita et al. 2010. “Tectone, a New Antihyperglycemic Anthraquinone from Tectona Grandis Leaves.” Natural product communications 5(3):427–30.
841. Siddiqi, S. A. and L. Misra. 2000. “Fatty Acids and Sterols from Cymbopogon Martinii Var. Motia Roots.” Zeitschrift fur Naturforschung. C, Journal of biosciences 55(9–10):843–45.
842. Siddiqui, Bina S., Fayaz Ahmad, Fouzia A. Sattar, and Sabira Begum. 2007. “Chemical Constituents from the Aerial Parts of Lippia Nodiflora Linn.” Archives of pharmacal research 30(12):1507–10.
843. Siddiqui, Bina S., Fayaz Ahmed, S.Kashif Ali, Sobiya Perwaiz, and Sabira Begum. 2009. “Steroidal Constituents from the Aerial Parts of Lippia Nodiflora Linn.” Natural product research 23(5):436–41.
844. Siddiqui, S. 1942. “A Note on Isolation of Three New Bitter Principles from the Neem Oil.” Curr Sci 11:278–279.
845. Siddiqui, Salimuzzaman, Shaheen Faizi, Taziq Mahmood, and Bina S. Siddiqui. 1986. “Margosinolide and Isomargosinolide, Two New Tetranortriteprenoids from Azadirachta Indica A, Juss (Meliaceae).” Tetrahedron 42(17):4849–56.
846. Sigler, P., R. Saksena, D. Deepak, and A. Khare. 2000. “C21 Steroidal Glycosides from Hemidesmus Indicus.” Phytochemistry 54(8):983–87.
847. Silva, A. A. S. et al. 2014. “Activity of Cycloartane-Type Triterpenes and Sterols Isolated from Musa Paradisiaca Fruit Peel against Leishmania Infantum Chagasi.” Phytomedicine : international journal of phytotherapy and phytopharmacology 21(11):1419–23.
848. Silva, Jose A. et al. 2007. “Isolation and Biochemical Characterization of a Galactoside Binding Lectin from Bauhinia Variegata Candida (BvcL) Seeds.” The protein journal 26(3):193–201.
849. Sindhia, V. R. and R. Bairwa. 2010. “PLANT REVIEW: Butea Monosperma.” International Journal of Pharmaceutical and Clinical Research 2(2):90–94.
850. Sindhu, Rakesh K. and Sandeep Arora. 2013. “Therapeutic Effect of Ficus Lacor Aerial Roots of Various Fractions on Adjuvant-Induced Arthritic Rats.” ISRN pharmacology 2013:634106.
851. Singh, Amritpal, Samir Malhotra, and Ravi Subban. 2008. “Dandelion (Taraxacum Officinale)-Hepatoprotective Herb with Therapeutic Potential.” Pharmacognosy Reviews 2(3):163.
852. Singh, Anil K., Chandan S. Chanotiya, Anju Yadav, and Alok Kalra. 2010. “Volatiles of Callicarpa Macrophylla: A Rich Source of Selinene Isomers.” Natural product communications 5(2):269–72.
853. Singh, B. et al. 2006. “Isolation, Structure Elucidation and in Vivo Hepatoprotective Potential of Trans-Tetracos-15-Enoic Acid from Indigofera Tinctoria Linn.” Phytotherapy research : PTR 20(10):831–39.
854. Singh, Damanpreet, Bikram Singh, and Rajesh Kumar Goel. 2011. “Traditional Uses, Phytochemistry and Pharmacology of Ficus Religiosa: A Review.” Journal of ethnopharmacology 134(3):565–83.
855. Singh, Ekta et al. 2011. “Phytochemistry, Traditional Uses and Cancer Chemopreventive Activity of Amla (Phyllanthus Emblica): The Sustainer.” Journal of Applied Pharmaceutical Science 2(1):176–83.
856. Singh, N. and S. S. Jaswal. 1968. “Alkaloids of Aconitum Palmatum: Carbon Skeleton of Vakognavine.” Tetrahedron letters 18:2219–22.
857. Singh, Neetu et al. 2010. “Verbascoside Isolated from Tectona Grandis Mediates Gastric Protection in Rats via Inhibiting Proton Pump Activity.” Fitoterapia 81(7):755–61.
858. Singh, Onkar, Mohammed Ali, and Shahnaz S. Husain. 2012. “Phytochemical Investigation and Antifungal Activity of the Seeds of Centratherum Anthelminticum Kuntze.” Acta poloniae pharmaceutica 69(6):1183–87.
859. Singh, Rajbir et al. 2008. “Anti-Free Radical Activities of Kaempferol Isolated from Acacia Nilotica (L.) Willd. Ex. Del.” Toxicology in vitro : an international journal published in association with BIBRA 22(8):1965–70.
860. Singh, Ram. 2016. “Asparagus Racemosus: A Review on Its Phytochemical and Therapeutic Potential.” Natural product research 30(17):1896–1908.
861. Singh, Sarita, Vidya Bhushan Pandey, and Tryambak Deo Singh. 2012. “Alkaloids and Flavonoids of Argemone Mexicana.” Natural product research 26(1):16–21.
862. Singh, Sarita, Tryambak Deo Singh, Virendra Pratap Singh, and Vidya Bhushan Pandey. 2010. “Quaternary Alkaloids of Argemone Mexicana.” Pharmaceutical biology 48(2):158–60.
863. Singh, Shiv Vardan et al. 2016. “A Mechanism-Based Pharmacological Evaluation of Efficacy of Flacourtia Indica in Management of Dyslipidemia and Oxidative Stress in Hyperlipidemic Rats.” Journal of basic and clinical physiology and pharmacology 27(2):121–29.
864. Singh, Shiv Vardan et al. 2017. “A Phenolic Glycoside from Flacourtia Indica Induces Heme Mediated Oxidative Stress in Plasmodium Falciparum and Attenuates Malaria Pathogenesis in Mice.” Phytomedicine : international journal of phytotherapy and phytopharmacology 30:1–9.
865. Sinha, R. K. 1992. “Herbal Remedies of Street Vendors for Some Urino-Genital Diseases.” Anc Sci Life 11(3–4):187–92.
866. Sinha, Sadhna et al. 2013. “A New Cytotoxic Quinolone Alkaloid and a Pentacyclic Steroidal Glycoside from the Stem Bark of Crataeva Nurvala: Study of Anti-Proliferative and Apoptosis Inducing Property.” European journal of medicinal chemistry 60:490–96.
867. Sini, Karanayil R., Barij N. Sinha, and Aiyolu Rajasekaran. 2011. “Antidiarrheal Activity of Capparis Zeylanica Leaf Extracts.” Journal of advanced pharmaceutical technology & research 2(1):39–42.
868. Sivaramakrishna, Chillara, Chirravuri V Rao, Golakoti Trimurtulu, Mulabagal Vanisree, and Gottumukkala V Subbaraju. 2005. “Triterpenoid Glycosides from Bacopa Monnieri.” Phytochemistry 66(23):2719–28.
869. Sohn, Charles E. 1894. Dictionary of the Active Principles of Plants : Alkaloids, Bitter Principles, Glucosides, Their Sources, Nature, and Chemical Characteristics, with Tabular Summary, Classification of Reactions, and Full Botanical and General Indexes. London: Baillière, Tindall and Cox.
870. Song, Q., D. Yang, G. Zhang, and C. Yang. 2001. “Volatiles from Ficus Hispida and Their Attractiveness to Fig Wasps.” Journal of chemical ecology 27(10):1929–42.
871. Sonkar, Nisha et al. 2014. “An Experimetal Evaluation of Nephroprotective Potential of Butea Monosperma Extract in Albino Rats.” Indian journal of pharmacology 46(1):109–12.
872. Sreelakshmi, V. and Annie Abraham. 2016a. “Anthraquinones and Flavonoids of Cassia Tora Leaves Ameliorate Sodium Selenite Induced Cataractogenesis in Neonatal Rats.” Food & function 7(2):1087–95.
873. Sreelakshmi, V. and Annie Abraham. 2016b. “Polyphenols of Cassia Tora Leaves Prevents Lenticular Apoptosis and Modulates Cataract Pathology in Sprague-Dawley Rat Pups.” Biomedicine & pharmacotherapy = Biomedecine & pharmacotherapie 81:371–78.
874. Srinivas, Pullela V, R.Ranga Rao, and J.Madhusudana Rao. 2006. “Two New Tetracyclic Triterpenes from the Heartwood of Ailanthus Excelsa Roxb.” Chemistry & biodiversity 3(8):930–34.
875. Sritularak, Boonchoo, Kullasap Tantrakarnsakul, Kittisak Likhitwitayawuid, and Vimolmas Lipipun. 2010. “New 2-Arylbenzofurans from the Root Bark of Artocarpus Lakoocha.” Molecules (Basel, Switzerland) 15(9):6548–58.
876. Sritularak, Boonchoo, Kullasap Tantrakarnsakul, Vimolmas Lipipun, and Kittisak Likhitwitayawuid. 2013. “Flavonoids with Anti-HSV Activity from the Root Bark of Artocarpus Lakoocha.” Natural product communications 8(8):1079–80.
877. Srivastava, Bhavana, Vikas C. Sharma, Pramila Pant, N. K. Pandey, and A. D. Jadhav. 2016. “Evaluation for Substitution of Stem Bark with Small Branches of Myrica Esculenta for Medicinal Use - A Comparative Phytochemical Study.” Journal of Ayurveda and integrative medicine 7(4):218–23.
878. SRIVASTAVA, K. C. and J. P. TEWARI. 1965. “[RESEARCH ON FATTY ACIDS IN THE OIL FROM THE SEED OF LUFFA ECHINATA ROXB].” Archiv der Pharmazie und Berichte der Deutschen Pharmazeutischen Gesellschaft 298:145–48.
879. Srivastava, Neena, Sayadda Khatoon, A. K. S. Rawat, Vartika Rai, and Shanta Mehrotra. 2009. “Chromatographic Estimation of P-Coumaric Acid and Triacontanol in an Ayurvedic Root Drug Patala (Stereospermum Suaveolens Roxb.).” Journal of chromatographic science 47(10):936–39.
880. Srivastava, Pooja, Jyotshna, Namita Gupta, Anil Kumar Maurya, and Karuna Shanker. 2014. “New Anti-Inflammatory Triterpene from the Root of Ricinus Communis.” Natural product research 28(5):306–11.
881. Srivastava, Pooja and Karuna Shanker. 2012. “Pluchea Lanceolata (Rasana): Chemical and Biological Potential of Rasayana Herb Used in Traditional System of Medicine.” Fitoterapia 83(8):1371–85.
882. Srivastava, S. K. and H. O. Gupta. 1983. “A New Flavanone from Adina Cordifolia.” Planta medica 48(1):58–59.
883. Srividhya, M., H. Hridya, V. Shanthi, and K. Ramanathan. 2017. “Bioactive Amento Flavone Isolated from Cassia Fistula L. Leaves Exhibits Therapeutic Efficacy.” 3 Biotech 7(1):33.
884. Srividya, A. R. et al. 2009. “Pharmacognostic, Phytochemical and Anti-Microbial Studies of Solanum Indicum Leaves.” Ancient science of life 29(1):3–5.
885. Srividya, A. R., S. P. Dhanabal, S. Jeevitha, V. J.Vishnu Varthan, and R.Rajesh Kumar. 2012. “Relationship between Antioxidant Properties and Chemical Composition of Abutilon Indicum Linn.” Indian journal of pharmaceutical sciences 74(2):163–67.
886. Stappen, Iris et al. 2014. “Chemical Composition and Biological Effects of Artemisia Maritima and Artemisia Nilagirica Essential Oils from Wild Plants of Western Himalaya.” Planta medica 80(13):1079–87.
887. Stephen, J. and P. L. Vijayammal. 2000. “Anti-Tumor Activity of Tylophora Asthmatica.” Ancient science of life 20(1–2):88–91.
888. Stintzing, Florian C. et al. 2004. “Betacyanins and Phenolic Compounds from Amaranthus Spinosus L. and Boerhavia Erecta L.” Zeitschrift fur Naturforschung. C, Journal of biosciences 59(1–2):1–8.
889. Stohs, Sidney J. and Sidhartha D. Ray. 2013. “A Review and Evaluation of the Efficacy and Safety of Cissus Quadrangularis Extracts.” Phytotherapy research : PTR 27(8):1107–14.
890. Strunz, George M. 2000. “Unsaturated Amides from Piper Species (Piperaceae).” Studies in Natural Products Chemistry 24(PART E):683–738.
891. Su, Hai-Guo et al. 2016. “[Study on chemical constituents of seeds of Croton tiglium and their cytotoxicities].” Zhongguo Zhong yao za zhi = Zhongguo zhongyao zazhi = China journal of Chinese materia medica 41(19):3620–23.
892. Su, Shu-Lan et al. 2009. “Isolation and Biological Activities of Neomyrrhaol and Other Terpenes from the Resin of Commiphora Myrrha.” Planta medica 75(4):351–55.
893. Subramaniam, Dharmalingam et al. 2008. “Activation of Apoptosis by 1-Hydroxy-5,7-Dimethoxy-2-Naphthalene-Carboxaldehyde, a Novel Compound from Aegle Marmelos.” Cancer research 68(20):8573–81.
894. Sultan, Phalisteen, Arif Jan, and Qazi Pervaiz. 2016. “Phytochemical Studies for Quantitative Estimation of Iridoid Glycosides in Picrorhiza Kurroa Royle.” Botanical studies 57(1):7.
895. Sultana, Nasim et al. 2010. “Novel Flavonoid Glycosides from the Bulbs of Urginea Indica Kunth.” Natural product research 24(11):1018–26.
896. Sun, Hai-lin, Ya-qiong Zhang, Xiao-yan Xie, and Yan-yun Che. 2014. “[Studies on chemical constituents from seeds of Euryale ferox].” Zhong yao cai = Zhongyaocai = Journal of Chinese medicinal materials 37(11):2019–21.
897. Sun, Jing et al. 2015. “A New Gamma-Alkylated-Gamma-Butyrolactone from the Roots of Solanum Melongena.” Chinese journal of natural medicines 13(9):699–703.
898. Sun, Ya Nan et al. 2016. “A New Phenolic Derivative with Soluble Epoxide Hydrolase and Nuclear Factor-kappaB Inhibitory Activity from the Aqueous Extract of Acacia Catechu.” Natural product research 30(18):2085–92.
899. Suri, Om P., Rajinder S. Sawhney, Manohar S. Bhatia, and Chand K. Atal. 1976. “Novel Secopyrrolizidine Alkaloids from Crotalaria Verrucosa.” Phytochemistry 15(6):1061–63. Retrieved (http://www.sciencedirect.com/science/article/pii/S0031942200844052).
900. Sutradhar, R. K. et al. 2006. “Bioactive Alkaloid from Sida Cordifolia Linn. with Analgesic and Anti-Inflammatory Activities.” Iranian Journal of Pharmacology and Therapeutics 5(2):175–78.
901. Sutradhar, Ranajit K., A. K. M.Matior Rahman, Mesbah U. Ahmad, and Sitesh C. Bachar. 2008. “Bioactive Flavones of Sida Cordifolia.” Phytochemistry Letters 1(4):179–82. Retrieved (http://www.sciencedirect.com/science/article/pii/S1874390008000621).
902. Sutradhar, Ranajit Kumar et al. 2007. “Anti-Inflammatory and Analgesic Alkaloid from Sida Cordifolia Linn.” Pakistan journal of pharmaceutical sciences 20(3):185–88.
903. Swamy, A. H. M.Viswanatha et al. 2013. “Neuroprotective Activity of Pongamia Pinnata in Monosodium Glutamate-Induced Neurotoxicity in Rats.” Indian journal of pharmaceutical sciences 75(6):657–63.
904. Swetha, M. P. and S. P. Muthukumar. 2016. “Characterization of Nutrients, Amino Acids, Polyphenols and Antioxidant Activity of Ridge Gourd (Luffa Acutangula) Peel.” Journal of food science and technology 53(7):3122–28.
905. Syed Abdul Rahman, Syarifah Nur, Norhanom Abdul Wahab, and Sri Nurestri Abd Malek. 2013. “In Vitro Morphological Assessment of Apoptosis Induced by Antiproliferative Constituents from the Rhizomes of Curcuma Zedoaria.” Evidence-based complementary and alternative medicine : eCAM 2013:257108.
906. Syu, W. J., M. J. Don, G. H. Lee, and C. M. Sun. 2001. “Cytotoxic and Novel Compounds from Solanum Indicum.” Journal of natural products 64(9):1232–33.
907. Takahashi, Toru, Aya Okiura, and Masahiro Kohno. 2017. “Phenylpropanoid Composition in Fig (Ficus Carica L.) Leaves.” Journal of natural medicines.
908. Talukdar, Sattya Narayan and Mohammad Nazir Hossain. 2014. “Phytochemical, Phytotherapeutical and Pharmacological Study of Momordica Dioica.” Evidence-based complementary and alternative medicine : eCAM 2014:806082.
909. Tamburini, Elena, Stefania Costa, Irene Rugiero, Paola Pedrini, and Maria Gabriella Marchetti. 2017. “Quantification of Lycopene, Beta-Carotene, and Total Soluble Solids in Intact Red-Flesh Watermelon (Citrullus Lanatus) Using On-Line Near-Infrared Spectroscopy.” Sensors (Basel, Switzerland) 17(4).
910. Tan, Mario A. et al. 2008. “Antitubercular Triterpenes and Phytosterols from Pandanus Tectorius Soland. Var. Laevis.” Journal of natural medicines 62(2):232–35.
911. Tanaka, Hitoshi et al. 2010. “A New Biisoflavonoid from the Roots of Erythrina Variegata.” Natural product communications 5(11):1781–84.
912. Tanaka, Hitoshi et al. 2011. “Three New Constituents from the Roots of Erythrina Variegata and Their Antibacterial Activity against Methicillin-Resistant Staphylococcus Aureus.” Chemistry & biodiversity 8(3):476–82.
913. Tanaka, Hitoshi et al. 2015. “Two New Isoflavanones from the Roots of Erythrina Variegata.” Natural product communications 10(3):499–501.
914. Tang, Shao-Nan et al. 2017. “Three New Areca Alkaloids from the Nuts of Areca Catechu.” Journal of Asian natural products research 1–5.
915. Teh, Soek Sin, Gwendoline Cheng Lian Ee, Siau Hui Mah, Yang Mooi Lim, and Zuraini Ahmad. 2013. “Cytotoxicity and Structure-Activity Relationships of Xanthone Derivatives from Mesua Beccariana, Mesua Ferrea and Mesua Congestiflora towards Nine Human Cancer Cell Lines.” Molecules (Basel, Switzerland) 18(2):1985–94.
916. Telagari, Madhusudhan and Kirankumar Hullatti. 2015. “In-Vitro Alpha-Amylase and Alpha-Glucosidase Inhibitory Activity of Adiantum Caudatum Linn. and Celosia Argentea Linn. Extracts and Fractions.” Indian journal of pharmacology 47(4):425–29.
917. Telek, Lehel., Franklin W. Martin, and Ruth M. Ruberte. 1974. “Bitter Compounds in Tubers of Dioscorea Bulbifera L.” Journal of Agricultural and Food Chemistry 22(2):332–34.
918. Terahara, N. et al. 1996. “Five New Anthocyanins, Ternatins A3, B4, B3, B2, and D2, from Clitoria Ternatea Flowers.” Journal of natural products 59(2):139–44.
919. Thattet, U. M. and S. A. Dahanukar. 1989. “Immunotherapeutic Modification of Experimental Infections by Indian Medicinal Plants.” Phytotherapy Research 3(2):43–49.
920. Thind, Tarunpreet S., Geetanjali Rampal, Satyam K. Agrawal, Ajit K. Saxena, and Saroj Arora. 2010. “Diminution of Free Radical Induced DNA Damage by Extracts/fractions from Bark of Schleichera Oleosa (Lour.) Oken.” Drug and chemical toxicology 33(4):329–36.
921. Thirupathy, Kumaresan P., Asish Tulshkar, and C. Vijaya. 2011. “Neuropharmacological Activity of Lippia Nodiflora Linn.” Pharmacognosy research 3(3):194–200.
922. Tian, Qi-Jian, Yang-Hui Ou, Xing-Bing He, and Yong-Dong Jiang. 2013. “One New Antitumour Cassane-Type Diterpene from Caesalpinia Crista.” Natural product research 27(6):537–40.
923. Tiwari, Neerja et al. 2008. “Iridoid Glycosides from Gmelina Arborea.” Phytochemistry 69(12):2387–90.
924. To, Kenneth K. W. et al. 2017. “Reversal of Multidrug Resistance by Marsdenia Tenacissima and Its Main Active Ingredients Polyoxypregnanes.” Journal of ethnopharmacology 203:110–19.
925. Toegel, Stefan et al. 2012. “Caesalpinia Sappan Extract Inhibits IL1beta-Mediated Overexpression of Matrix Metalloproteinases in Human Chondrocytes.” Genes & nutrition 7(2):307–18.
926. Tomar, S. S. and P. Dureja. 2001. “New Minor Constituents from Anethum Sowa.” Fitoterapia 72(1):76–77.
927. Toomer, Ondulla T. 2017. “Nutritional Chemistry of the Peanut (Arachis Hypogaea).” Critical reviews in food science and nutrition 1–12.
928. Tram, Nguyen Thi Ngoc, Maya Mitova, Vassya Bankova, Nedyalka Handjieva, and Simeon S. Popov. 2002. “GC-MS of Crinum Latifolium L. Alkaloids.” Zeitschrift fur Naturforschung. C, Journal of biosciences 57(3–4):239–42.
929. Tran, Manh Hung et al. 2009. “Phenolic Glycosides from Alangium Salviifolium Leaves with Inhibitory Activity on LPS-Induced NO, PGE(2), and TNF-Alpha Production.” Bioorganic & medicinal chemistry letters 19(15):4389–93.
930. Traxler, James T. 1971. “Piperanine, a Pungent Component of Black Pepper.” Journal of Agricultural and Food Chemistry 19(6):1135–38.
931. Tripathi, P., R. Kumar, A. K. Sharma, A. Mishra, and R. Gupta. 2010. “Pistia Stratiotes (Jalkumbhi).” Pharmacognosy reviews 4(8):153–60.
932. Tsuzuki, K. et al. 2001. “Triterpenoids from Adiantum Caudatum.” Phytochemistry 58(2):363–67.
933. Turak, Ablajan, She-Po Shi, Yong Jiang, and Peng-Fei Tu. 2014. “Dimeric Guaianolides from Artemisia Absinthium.” Phytochemistry 105:109–14.
934. Ubillas, R. P. et al. 1999. “Antihyperglycemic Furanosesquiterpenes from Commiphora Myrrha.” Planta medica 65(8):778–79.
935. Uchikoba, T., H. Yonezawa, and M. Kaneda. 1998. “Cucumisin like Protease from the Sarcocarp of Benincasa Hispida Var. Ryukyu.” Phytochemistry 49(8):2215–19.
936. Uddin, Ghias et al. 2012. “Pistagremic Acid a New Leishmanicidal Triterpene Isolated from Pistacia Integerrima Stewart.” Journal of enzyme inhibition and medicinal chemistry 27(5):646–48.
937. Ueng, Yune-Fang et al. 2015. “Effects of Aqueous Extract of Ruta Graveolens and Its Ingredients on Cytochrome P450, Uridine Diphosphate (UDP)-Glucuronosyltransferase, and Reduced Nicotinamide Adenine Dinucleotide (Phosphate) (NAD(P)H)-Quinone Oxidoreductase in Mice.” Journal of food and drug analysis 23(3):516–28.
938. Ukani, M. D., N. K. Mehta, and D. D. Nanavati. 1996. “Aconitum Heterophyllum (Ativisha) in Ayurveda.” Ancient science of life 16(2):166–71.
939. Ulbricht, Catherine et al. 2011. “An Evidence-Based Systematic Review of Senna (Cassia Senna) by the Natural Standard Research Collaboration.” Journal of dietary supplements 8(2):189–238.
940. Ullah, Zia, Rashad Mehmood, Muhammad Imran, Abdul Malikb, and Rehana A. Afzal. 2012. “Flavonoid Constituents of Pistacia Integerrima.” Natural product communications 7(8):1011–14.
941. Valecha, Rekha and Dinesh Dhingra. 2016. “Behavioral and Biochemical Evidences for Antidepressant-Like Activity of Celastrus Paniculatus Seed Oil in Mice.” Basic and clinical neuroscience 7(1):49–56.
942. Vanajothi, Ramar and Pappu Srinivasan. 2015. “Bioassay-Guided Isolation and Identification of Bioactive Compound from Aerial Parts of Luffa Acutangula against Lung Cancer Cell Line NCI-H460.” Journal of receptor and signal transduction research 35(4):295–302.
943. Varshney, S. C., S. A. Rizvi, and P. C. Gupta. 1972. “Chemical Examination of the Flowers of Pterospermum Acerifolium.” Planta medica 21(4):358–63.
944. Venkatesh, Pichairajan, Pulok K. Mukherjee, and Bikas C. Pal. 2011. “Acridanone Alkaloid in Baliospermum Montanum--Evaluation of Its Effect against Anaphylaxis.” Planta medica 77(17):1947–49.
945. Venkateswara, R., K. Sankara Rao, and C. S. Vaidyanathan. 1987. “Cryptosin - a New Cardenolide in Tissue Culture and Intact Plants of Cryptolepis Buchanani Roem. & Schult.” Plant cell reports 6(4):291–93.
946. Verma, Amita, Bahar Ahmed, et al. 2017. “Novel Glycoside from Wedelia Calendulacea Inhibits Diethyl Nitrosamine-Induced Renal Cancer via Downregulating the COX-2 and PEG2 through Nuclear Factor-kappaB Pathway.” Inflammopharmacology 25(1):159–75.
947. Verma, Amita, Deepika Singh, et al. 2017. “Triterpenoids Principle of Wedelia Calendulacea Attenuated Diethynitrosamine-Induced Hepatocellular Carcinoma via down-Regulating Oxidative Stress, Inflammation and Pathology via NF-kB Pathway.” Inflammopharmacology.
948. Verma, Ram S., Rajendra C. Padalia, and Amit Chauhan. 2014. “Essential Oil Composition of Aegle Marmelos (L.) Correa: Chemotypic and Seasonal Variations.” Journal of the science of food and agriculture 94(9):1904–13.
949. Verma, S. M. and K. B. Suresh. 2002. “Phytochemical Investivations of Indigofera Tinctoria Linn Leaves.” Ancient science of life 21(4):235–39.
950. Verotta, Luisella et al. 2004. “4-Alkyl- and 4-Phenylcoumarins from Mesua Ferrea as Promising Multidrug Resistant Antibacterials.” Phytochemistry 65(21):2867–79.
951. Vien, Le Thi et al. 2017. “Flavonoid Glycosides from Barringtonia Acutangula.” Bioorganic & medicinal chemistry letters 27(16):3776–81.
952. Vijayakumar, S., G. Presannakumar, and N. R. Vijayalakshmi. 2009. “Investigations on the Effect of Flavonoids from Banana, Musa Paradisiaca L. on Lipid Metabolism in Rats.” Journal of dietary supplements 6(2):111–23.
953. Vinayagam, A. and P. N. Sudha. 2015. “Separation and Identification of Phenolic Acid and Flavonoids from Nerium Indicum Flowers.” Indian journal of pharmaceutical sciences 77(1):91–95.
954. Vinod, N. V, R. Shijina, K. V Dileep, and C. Sadasivan. 2010. “Inhibition of Beta-Lactamase by 1,4-Naphthalenedione from the Plant Holoptelea Integrifolia.” Applied biochemistry and biotechnology 160(6):1752–59.
955. Viswanathan, M. B., D. Thangadurai, K. T. Vendan, and N. Ramesh. 1999. “Chemical Analysis and Nutritional Assessment of Teramnus Labialis (L.) Spreng. (Fabaceae).” Plant foods for human nutrition (Dordrecht, Netherlands) 54(4):345–52.
956. Wagner, H., B. Geyer, M. Fiebig, Y. Kiso, and H. Hikino. 1986. “Isobutrin and Butrin, the Antihepatotoxic Principles of Butea Monosperma Flowers.” Planta medica (2):77–79.
957. Wagner, H., B. Geyer, Y. Kiso, H. Hikino, and G. S. Rao. 1986. “Coumestans as the Main Active Principles of the Liver Drugs Eclipta Alba and Wedelia Calendulacea.” Planta medica (5):370–74.
958. Wagner, Hildebert and Sabine Bladt, eds. 1996. “Drugs with Pungent-Tasting Principles BT - Plant Drug Analysis: A Thin Layer Chromatography Atlas.” Pp. 291–303 in. Berlin, Heidelberg: Springer Berlin Heidelberg.
959. Wang, Chao-Min et al. 2016. “Antibacterial and Synergistic Activity of Pentacyclic Triterpenoids Isolated from Alstonia Scholaris.” Molecules (Basel, Switzerland) 21(2):139.
960. Wang, Guo-Kai, Bin-Bin Lin, and Min-Jian Qin. 2014. “[Study on chemical constituents from leaf of Bombax ceiba (II)].” Zhong yao cai = Zhongyaocai = Journal of Chinese medicinal materials 37(2):240–42.
961. Wang, Guo Kai et al. 2013. “A New Lignan with Anti-HBV Activity from the Roots of Bombax Ceiba.” Natural product research 27(15):1348–52.
962. Wang, Hexiang and Tzi Bun Ng. 2002. “Luffangulin, a Novel Ribosome Inactivating Peptide from Ridge Gourd (Luffa Acutangula) Seeds.” Life sciences 70(8):899–906.
963. Wang, Jian-Rong et al. 2011. “Structural and Stereochemical Studies of Five New Pregnane Steroids from the Stem Bark of Toona Ciliata Var. Pubescens.” Steroids 76(6):571–76.
964. Wang, Qiu Hong et al. 2014. “Structural Characterization and Antioxidant Activities of Polysaccharides from Citrus Aurantium L.” International journal of biological macromolecules 67:112–23.
965. Wang, Xing-Biao et al. 2009. “Nematicidal Cardenolides from Nerium Indicum Mill.” Chemistry & biodiversity 6(3):431–36.
966. Wang, Yan-Hong, Bharathi Avula, Atul N. Jadhav, Troy J. Smillie, and Ikhlas A. Khan. 2008. “Structural Characterization and Identification of Ecdysteroids from Sida Rhombifolia L. in Positive Electrospray Ionization by Tandem Mass Spectrometry.” Rapid communications in mass spectrometry : RCM 22(16):2413–22.
967. Wang, Ying et al. 2012. “Curculigoside Isolated from Curculigo Orchioides Prevents Hydrogen Peroxide-Induced Dysfunction and Oxidative Damage in Calvarial Osteoblasts.” Acta biochimica et biophysica Sinica 44(5):431–41.
968. Wang, Yun-Song, Rong Huang, Hao Lu, Feng-Ya Li, and Jing-Hua Yang. 2010. “A New 2’-oxygenated Flavone Glycoside from Litsea Glutinosa (Lour.) C. B. Rob.” Bioscience, biotechnology, and biochemistry 74(3):652–54.
969. Wang, Zhen-Hui et al. 2013. “Phenolic Glycosides from Curculigo Orchioides Gaertn.” Fitoterapia 86:64–69.
970. Wang, Zhen-Hui, Chao Niu, De-Jun Zhou, Ji-Chuan Kong, and Wen-Kui Zhang. 2017. “Three New Abietane-Type Diterpenoids from Callicarpa Macrophylla Vahl.” Molecules (Basel, Switzerland) 22(5).
971. Wangteeraprasert, Ruchira et al. 2012. “Bioactive Compounds from Carissa Spinarum.” Phytotherapy research : PTR 26(10):1496–99.
972. Washiyama, Makiko, Yohei Sasaki, Tomokazu Hosokawa, and Seiji Nagumo. 2009. “Anti-Inflammatory Constituents of Sappan Lignum.” Biological & pharmaceutical bulletin 32(5):941–44.
973. Wen, Jing, Tian-Yi Qiu, Xin-Jia Yan, and Feng Qiu. 2017. “Four Novel Bisabolane-Type Sesquiterpenes from Curcuma Longa.” Journal of Asian natural products research 1–6.
974. Wen, Qingwei et al. 2012. “Phenolic and Lignan Glycosides from the Butanol Extract of Averrhoa Carambola L. Root.” Molecules (Basel, Switzerland) 17(10):12330–40.
975. Wieczorek, Martyna N., Michal Walczak, Marzena Skrzypczak-Zielinska, and Henryk H. Jelen. 2017. “Bitter Taste of Brassica Vegetables: The Role of Genetic Factors, Receptors, Isothiocyanates, Glucosinolates, and Flavor Context.” Critical reviews in food science and nutrition 1–11.
976. Win, Nwet Nwet et al. 2017. “Labdane Diterpenoids from Curcuma Amada Rhizomes Collected in Myanmar and Their Antiproliferative Activities.” Fitoterapia 122:34–39.
977. Wirngo, Fonyuy E., Max N. Lambert, and Per B. Jeppesen. 2016. “The Physiological Effects of Dandelion (Taraxacum Officinale) in Type 2 Diabetes.” The review of diabetic studies : RDS 13(2–3):113–31.
978. Wongkham, S. et al. 1995. “Isolectins from Seeds of Artocarpus Lakoocha.” Phytochemistry 40(5):1331–34.
979. Wongkham, S., K. Taketa, M. Liu, and H. Taga. 1996. “Affinity Electrophoretic Determination of Oligosaccharide Specificity of Butea Monosperma Agglutinin.” Electrophoresis 17(1):98–103.
980. Wood, Horatio C. 1872. A Year-Book of Therapeutics, Pharmacy and Allied Sciences - Google Books. New York: William Wood Company.
981. Woolfe, J. A. 1977. “The Effect of Okra Mucilage (Hibiscus Esculentus L.) on the Plasma Cholesterol Level in Rats.” The Proceedings of the Nutrition Society 36(2):59A.
982. Wu, Hai-Feng et al. 2014. “Norcassane- and Cassane-Type Furanoditerpenoids from the Seeds of Caesalpinia Sappan.” Fitoterapia 98:22–26.
983. Wu, Li-Sheng et al. 2010. “Cytotoxic Polyphenols against Breast Tumor Cell in Smilax China L.” Journal of ethnopharmacology 130(3):460–64.
984. Wu, Qiu-ping et al. 2007. “[Chemical constituents from the leaves of Cassia angustifolia].” Zhong yao cai = Zhongyaocai = Journal of Chinese medicinal materials 30(10):1250–52.
985. Wu, Sheng and Li Tian. 2017. “Diverse Phytochemicals and Bioactivities in the Ancient Fruit and Modern Functional Food Pomegranate (Punica Granatum).” Molecules (Basel, Switzerland) 22(10).
986. Wu, Shou-Fang, Fang-Rong Chang, et al. 2011. “Anti-Inflammatory and Cytotoxic Neoflavonoids and Benzofurans from Pterocarpus Santalinus.” Journal of natural products 74(5):989–96.
987. Wu, Shou-Fang, Tsong-Long Hwang, et al. 2011. “Bioactive Components from the Heartwood of Pterocarpus Santalinus.” Bioorganic & medicinal chemistry letters 21(18):5630–32.
988. Wu, Yanping et al. 2016. “Antibacterial Activity and Membrane-Disruptive Mechanism of 3-P-Trans-Coumaroyl-2-Hydroxyquinic Acid, a Novel Phenolic Compound from Pine Needles of Cedrus Deodara, against Staphylococcus Aureus.” Molecules (Basel, Switzerland) 21(8).
989. Xie, Guo-Yong et al. 2013. “New Isoflavones with Cytotoxic Activity from the Rhizomes of Iris Germanica L.” Natural product research 27(23):2173–77.
990. Xie, Guo-Yong et al. 2014. “[Chemical constituents from rhizomes of Iris germanica].” Zhongguo Zhong yao za zhi = Zhongguo zhongyao zazhi = China journal of Chinese materia medica 39(5):846–50.
991. Xie, Qiuqiao et al. 2016. “Protective Effect of 2-Dodecyl-6-Methoxycyclohexa-2, 5-Diene-1, 4-Dione, Isolated from Averrhoa Carambola L., Against Palmitic Acid-Induced Inflammation and Apoptosis in Min6 Cells by Inhibiting the TLR4-MyD88-NF-kappaB Signaling Pathway.” Cellular physiology and biochemistry : international journal of experimental cellular physiology, biochemistry, and pharmacology 39(5):1705–15.
992. Xu, Guang-Kai et al. 2017. “Effects of Ethanol Extract of Bombax Ceiba Leaves and Its Main Constituent Mangiferin on Diabetic Nephropathy in Mice.” Chinese journal of natural medicines 15(8):597–605.
993. Xu, H., Z. H. Zhou, and J. S. Yang. 1994. “[Compounds from Caesalpinia sappan L].” Zhongguo Zhong yao za zhi = Zhongguo zhongyao zazhi = China journal of Chinese materia medica 19(8):485–486,511.
994. Xu, Hui-Wang, Fan Cheng, Jun Wu, and Yuan Zhou. 2012. “[Chemical constituents from wood of the Chinese Hainan mangrove-associated plant, Thespesia populnea].” Zhong yao cai = Zhongyaocai = Journal of Chinese medicinal materials 35(12):1953–56.
995. Xu, Jing, Yuanqiang Guo, Peng Zhao, et al. 2011. “Neuroprotective Cadinane Sesquiterpenes from the Resinous Exudates of Commiphora Myrrha.” Fitoterapia 82(8):1198–1201.
996. Xu, Jing, Yuanqiang Guo, Yushan Li, et al. 2011. “Sesquiterpenoids from the Resinous Exudates of Commiphora Myrrha and Their Neuroprotective Effects.” Planta medica 77(18):2023–28.
997. Xu, Jing et al. 2012. “Four New Sesquiterpenes from Commiphora Myrrha and Their Neuroprotective Effects.” Fitoterapia 83(4):801–5.
998. Xu, Jing, Yihang Sun, et al. 2015. “Bioactive Diterpenoids from the Leaves of Callicarpa Macrophylla.” Journal of natural products 78(7):1563–69.
999. Xu, Jing, Feifei Ji, et al. 2015. “Characterization and Biological Evaluation of Diterpenoids from Casearia Graveolens.” Journal of natural products 78(11):2648–56.
1000. Xu, Jing et al. 2016. “Bioactive Terpenoids from Salvia Plebeia: Structures, NO Inhibitory Activities, and Interactions with iNOS.” Journal of natural products 79(11):2924–32.
1001. Xu, Pingping, Shuhong Guan, Ruihong Feng, Renneng Tang, and Dean Guo. 2012. “Separation of Four Homoisoflavonoids from Caesalpinia Sappan by High-Speed Counter-Current Chromatography.” Phytochemical analysis : PCA 23(3):228–31.
1002. Xu, Rui et al. 2015. “New Pregnane Glycosides from Gymnema Sylvestre.” Molecules (Basel, Switzerland) 20(2):3050–66.
1003. Xu, Xiaohui et al. 2014. “Protective Effects of Total Extracts of Averrhoa Carambola L. (Oxalidaceae) Roots on Streptozotocin-Induced Diabetic Mice.” Cellular physiology and biochemistry : international journal of experimental cellular physiology, biochemistry, and pharmacology 33(5):1272–82.
1004. Xu, Xudong et al. 2016. “Cassane Diterpenes with Oxygen Bridge from the Seeds of Caesalpinia Sappan.” Fitoterapia 112:205–10.
1005. Yadav, Kavita N., Prasad V Kadam, Jigna A. Patel, and Manohar J. Patil. 2014. “Strychnos Potatorum: Phytochemical and Pharmacological Review.” Pharmacognosy reviews 8(15):61–66.
1006. Yadav, Navneet Kumar et al. 2015. “Saraca Indica Bark Extract Shows in Vitro Antioxidant, Antibreast Cancer Activity and Does Not Exhibit Toxicological Effects.” Oxidative medicine and cellular longevity 2015:205360.
1007. Yadav, Prem P. et al. 2009. “Cassane Diterpenes from Caesalpinia Bonduc.” Phytochemistry 70(2):256–61. Retrieved (http://www.sciencedirect.com/science/article/pii/S0031942208005980).
1008. Yadav, Rajnish Kumar, Bankim Chandra Nandy, Siddhartha Maity, Srimanta Sarkar, and Sudipta Saha. 2015. “Phytochemistry, Pharmacology, Toxicology, and Clinical Trial of Ficus Racemosa.” Pharmacognosy reviews 9(17):73–80.
1009. Yadav, Suraj Singh, Manish Kumar Singh, Pawan Kumar Singh, and Vipin Kumar. 2017. “Traditional Knowledge to Clinical Trials: A Review on Therapeutic Actions of Emblica Officinalis.” Biomedicine & pharmacotherapy = Biomedecine & pharmacotherapie 93:1292–1302.
1010. Yadava, R. N. and Shivani Jain. 2004. “A Novel Bioactive Flavonol Glycoside from Teramnus Labialis Spreng.” Natural product research 18(6):537–42.
1011. Yadava, R. N. and V. M. Reddy. 2001. “A New Flavone Glycoside, 5-Hydroxy 7,3’,4’,5’-tetra-Methoxyflavone 5-O-Beta-D-Xylopyranosyl-(1-->2)-Alpha-L-Rhamnopyranoside from Bauhinia Variegata Linn.” Journal of Asian natural products research 3(4):341–46.
1012. Yadava, R. N. and V.Madhu Sudhan Reddy. 2003. “Anti-Inflammatory Activity of a Novel Flavonol Glycoside from the Bauhinia Variegata Linn.” Natural product research 17(3):165–69.
1013. Yadava, R. N. and Sheetal Sodhi. 2002. “A New Flavone Glycoside: 5,7,3’,4’-tetrahydroxy-3-Methoxy Flavone-7-O-Beta-D-Galactopyranosyl-(1-->4)-O-Beta-D-Glucopyranoside from the Stem of Acacia Catechu Willd.” Journal of Asian natural products research 4(1):11–15.
1014. Yadava, R. N. and Y. Syeda. 1994. “An Isoflavone Glycoside from the Seeds of Trichosanthes Anguina.” Phytochemistry 36(6):1519–21.
1015. Yadava, R. N. and Lata Tiwari. 2005. “A Potential Antiviral Flavone Glycoside from the Seeds of Butea Monosperma O. Kuntze.” Journal of Asian natural products research 7(2):185–88.
1016. Yadava, R. N. and Lata Tiwari. 2007. “New Antifungal Flavone Glycoside from Butea Monosperma O. Kuntze.” Journal of enzyme inhibition and medicinal chemistry 22(4):497–500.
1017. Yagi, Nobuo, Hiroshi Nakahashi, Yusei Kashima, and Mitsuo Miyazawa. 2014. “Isolation and Biological Activity of a Novel Cadinane-Type Sesquiterpenoid from the Essential Oil of Alangium Salviifolium.” Journal of oleo science 63(12):1223–29.
1018. Yakubu, Musa T. et al. 2015. “Antidiarrhoeal Activity of Musa Paradisiaca Sap in Wistar Rats.” Evidence-based complementary and alternative medicine : eCAM 2015:683726.
1019. Yamada, Y. and T. Hashimoto. 1982. “Production of Tropane Alkaloids in Cultured Cells of Hyoscyamus Niger.” Plant cell reports 1(3):101–3.
1020. Yanfang, Zhao, Luo Xingping, Zhai Zongde, Chen Liren, and Li Yongmin. 2006. “Simultaneous Determination of Andrographolide and Dehydroandrographolide in Andrographis Paniculata and Chinese Medicinal Preparations by Microemulsion Electrokinetic Chromatography.” Journal of pharmaceutical and biomedical analysis 40(1):157–61.
1021. Yang, Chang-Syun et al. 2017. “New Benzenoid Derivatives and Other Constituents from Lawsonia Inermis with Inhibitory Activity against NO Production.” Molecules (Basel, Switzerland) 22(6).
1022. Yang, Dan, Haihui Xie, Bao Yang, and Xiaoyi Wei. 2014. “Two Tetrahydroisoquinoline Alkaloids from the Fruit of Averrhoa Carambola.” Phytochemistry Letters 7:217–20.
1023. Yang, Guang-Ming, Xia Tu, Liang-Jing Liu, and Yang Pan. 2010. “Two New Bisindole Alkaloids from the Seeds of Strychnos Nux-Vomica.” Fitoterapia 81(7):932–36.
1024. Yang, Zhong-Duo, Dong-Zhu Duan, Wei-Wei Xue, Xiao-Jun Yao, and Shuo Li. 2012. “Steroidal Alkaloids from Holarrhena Antidysenterica as Acetylcholinesterase Inhibitors and the Investigation for Structure-Activity Relationships.” Life sciences 90(23–24):929–33.
1025. Yao, Sheng et al. 2014. “Polyoxypregnane Steroids from the Stems of Marsdenia Tenacissima.” Journal of natural products 77(9):2044–53.
1026. Yao, Xue et al. 2016. “Moracin C, A Phenolic Compound Isolated from Artocarpus Heterophyllus, Suppresses Lipopolysaccharide-Activated Inflammatory Responses in Murine Raw264.7 Macrophages.” International journal of molecular sciences 17(8).
1027. Yap, Veronica Alicia et al. 2015. “Hispidacine, an Unusual 8,4’-oxyneolignan-Alkaloid with Vasorelaxant Activity, and Hispiloscine, an Antiproliferative Phenanthroindolizidine Alkaloid, from Ficus Hispida Linn.” Phytochemistry 109:96–102.
1028. Yarla, Nagendra Sastry et al. 2015. “5-Lipoxygenase and Cyclooxygenase Inhibitory Dammarane Triterpenoid 1 from Borassus Flabellifer Seed Coat Inhibits Tumor Necrosis Factor-Alpha Secretion in LPSInduced THP-1 Human Monocytes and Induces Apoptosis in MIA PaCa-2 Pancreatic Cancer Cells.” Anti-cancer agents in medicinal chemistry 15(8):1066–77.
1029. Ye, Bengui, Jinrong Yang, Jun Li, Ting Niu, and Shu Wang. 2014. “In Vitro and in Vivo Antitumor Activities of Tenacissoside C from Marsdenia Tenacissima.” Planta medica 80(1):29–38.
1030. Yin, Hai-Long et al. 2013. “Four New Coumarinolignoids from Seeds of Solanum Indicum.” Fitoterapia 84:360–65.
1031. Yin, Hai-Long et al. 2014. “Two New Coumarins from the Seeds of Solanum Indicum.” Journal of Asian natural products research 16(2):153–57.
1032. Yin, Hao et al. 2013. “The Separation of Flavonoids from Pongamia Pinnata Using Combination Columns in High-Speed Counter-Current Chromatography with a Three-Phase Solvent System.” Journal of chromatography. A 1315:80–85.
1033. Yin, Wei, Zu-rong Song, Jin-qi Liu, and Guo-sheng Zhang. 2015. “[Chemical Constituents of Citrus medica Fruit].” Zhong yao cai = Zhongyaocai = Journal of Chinese medicinal materials 38(10):2091–94.
1034. Ying, Ju and Xiao Bing. 2016. “Chemical Constituents of Cyperus Rotundus L. and Their Inhibitory Effects on Uterine Fibroids.” African health sciences 16(4):1000–1006.
1035. Yodsaoue, Orapun et al. 2008. “Phanginin A-K, Diterpenoids from the Seeds of Caesalpinia Sappan Linn.” Phytochemistry 69(5):1242–49.
1036. Yokosuka, Akihito, Koji Sato, and Yoshihiro Mimaki. 2010. “Cycloartane Glycosides from the Rhizomes of Curculigo Orchioides.” Phytochemistry 71(17–18):2174–81.
1037. Yoshikawa, K. et al. 2000. “New Oleanene Triterpenoid Saponins from Madhuca Longifolia.” Journal of natural products 63(12):1679–81.
1038. Yoshikawa, Masayuki et al. 2007. “Medicinal Flowers. XII.(1)) New Spirostane-Type Steroid Saponins with Antidiabetogenic Activity from Borassus Flabellifer.” Chemical & pharmaceutical bulletin 55(2):308–16.
1039. Yoshikawa, Masayuki, Yi Zhang, Tao Wang, Seikou Nakamura, and Hisashi Matsuda. 2008. “New Triterpene Constituents, Foliasalacins A(1)-A(4), B(1)-B(3), and C, from the Leaves of Salacia Chinensis.” Chemical & pharmaceutical bulletin 56(7):915–20.
1040. You, Ting-Ting, Jian-Zhen Mao, Tong-Qi Yuan, Jia-Long Wen, and Feng Xu. 2013. “Structural Elucidation of the Lignins from Stems and Foliage of Arundo Donax Linn.” Journal of agricultural and food chemistry 61(22):5361–70.
1041. Youn, Ui Joung et al. 2014. “Bioactive Sesquiterpene Lactones and Other Compounds Isolated from Vernonia Cinerea.” Fitoterapia 93:194–200.
1042. Yu, Guo-Hua et al. 2016. “[Analysis of chemical constituents from Citrus aurantium by UHPLC-LTQ-Orbitrap-MS/MS].” Zhongguo Zhong yao za zhi = Zhongguo zhongyao zazhi = China journal of Chinese materia medica 41(18):3371–78.
1043. Yu, Man-Shan et al. 2007. “New Polysaccharide from Nerium Indicum Protects Neurons via Stress Kinase Signaling Pathway.” Brain research 1153:221–30.
1044. Yuan, Huaibo et al. 2014. “Hypoglycemic Effect of Triterpenoid-Rich Extracts from Euryale Ferox Shell on Normal and Streptozotocin-Diabetic Mice.” Pakistan journal of pharmaceutical sciences 27(4):859–64.
1045. Yuan, Jiu-zhi et al. 2004. “[Studies on dihydroflavonol glycosides from rhizome of Smilax glabra].” Zhongguo Zhong yao za zhi = Zhongguo zhongyao zazhi = China journal of Chinese materia medica 29(9):867–70.
1046. Yuan, Ting et al. 2017. “Chemical Constituents from Curcuma Longa L. and Their Inhibitory Effects of Nitric Oxide Production.” Natural product research 1–6.
1047. Yuan, Wen-Jun et al. 2017. “Flavonoids from the Roots of Artocarpus Heterophyllus.” Fitoterapia 117:133–37.
1048. Zachariah, T.John. 2008. “Ginger.” Pp. 70–96 in Chemistry of spices, edited by V. A. Parthasarathy, B. Chempakam, and T. J. Zachariah. Cambridge: CABI.
1049. Zahara, Kulsoom et al. 2014. “A Review of Therapeutic Potential of Saussurea Lappa-An Endangered Plant from Himalaya.” Asian Pacific journal of tropical medicine 7S1:S60-9.
1050. Zalke, Ashish S., B. Duraiswamy, Upendra B. Gandagule, and Nidhi Singh. 2013. “Pharmacognostical Evaluation of Cardiospermum Halicacabum Linn. Leaf and Stem.” Ancient science of life 33(1):15–21.
1051. Zhang, Bing-Jie et al. 2014. “Dimeric Erythrina Alkaloids from the Flower of Erythrina Variegata.” Organic letters 16(24):6400–6403.
1052. Zhang, Wei-Ming et al. 2014. “Triterpenoids and Sterols from the Leaves and Twigs of Melia Azedarach.” Natural products and bioprospecting 4(3):157–62.
1053. Zhang, Wenna, Wei Zhang, Jianguang Luo, and Lingyi Kong. 2013. “A New Steroidal Glycoside from the Seeds of Hyoscyamus Niger.” Natural product research 27(21):1971–74.
1054. Zhang, Xiaopo et al. 2013. “Anti-Hyperlipidemic Effects and Potential Mechanisms of Action of the Caffeoylquinic Acid-Rich Pandanus Tectorius Fruit Extract in Hamsters Fed a High Fat-Diet.” PloS one 8(4):e61922.
1055. Zhang, Xuenong, Wenwen Zhao, Ying Wang, Jinjian Lu, and Xiuping Chen. 2016. “The Chemical Constituents and Bioactivities of Psoralea Corylifolia Linn.: A Review.” The American journal of Chinese medicine 44(1):35–60.
1056. Zhang, Yi, Seikou Nakamura, Yutana Pongpiriyadacha, Hisashi Matsuda, and Masayuki Yoshikawa. 2008. “Absolute Structures of New Megastigmane Glycosides, Foliasalaciosides E(1), E(2), E(3), F, G, H, and I from the Leaves of Salacia Chinensis.” Chemical & pharmaceutical bulletin 56(4):547–53.
1057. Zhang, Yu and Ting-Ting Zhang. 2010. “Studies on the Chemical Constituents from the Stem and Leaves of Tagetes Erecta.” Zhong yao cai = Zhongyaocai = Journal of Chinese medicinal materials 33(9):1412–14.
1058. Zhao, Gui-Qin and Jun-Xing Dong. 2008. “[Triterpenoid saponins from flower bud of Jasminum officinale var. grandiflorum].” Zhongguo Zhong yao za zhi = Zhongguo zhongyao zazhi = China journal of Chinese materia medica 33(1):38–42.
1059. Zhao, Gui-qin, Jing-jing Xia, and Jun-xing Dong. 2007. “[Glycosides from flowers of Jasminum officinale L. var. grandiflorum].” Yao xue xue bao = Acta pharmaceutica Sinica 42(10):1066–69.
1060. Zhao, Gui-Qin, Zhi-Feng Yin, and Jun-Xing Dong. 2008. “[A new secoiridoid from the flowers of Jasminum officinale L. var. grandiflorum].” Yao xue xue bao = Acta pharmaceutica Sinica 43(5):513–17.
1061. Zhao, Gui-qin, Zhi-feng Yin, Yu-cui Liu, and Hong-bo Li. 2011. “[Iridoid glycosides from buds of Jasminum officinale L. var. grandiflorum].” Yao xue xue bao = Acta pharmaceutica Sinica 46(10):1221–24.
1062. Zhao, H. R., S. X. Zhao, C. Q. Sun, and D. Guillaume. 1989. “Glucosylsterols in Extracts of Euryale Ferox Identified by High Resolution NMR and Mass Spectrometry.” Journal of lipid research 30(10):1633–37.
1063. Zhao, Huanxin, Hong Bai, Yuanshu Wang, Wei Li, and Kazuo Koike. 2008. “A New Homoisoflavan from Caesalpinia Sappan.” Journal of natural medicines 62(3):325–27.
1064. Zhao, R., C. Chen, and Y. Wu. 1991. “[Isolation and structure determination of furan sesquiterpene from Chinese traditional herb ezhu (rhizome of Curcuma zedoaria Rosc.)].” Zhongguo Zhong yao za zhi = Zhongguo zhongyao zazhi = China journal of Chinese materia medica 16(5):291–292,319.
1065. Zhao, Y. Y., C. B. Cui, B. Cai, B. Han, and Q. S. Sun. 2005. “A New Phenanthraquinone from the Stems of Bauhinia Variegata L.” Journal of Asian natural products research 7(6):835–38.
1066. Zheng, Zong-Ping et al. 2009. “Chemical Components and Tyrosinase Inhibitors from the Twigs of Artocarpus Heterophyllus.” Journal of agricultural and food chemistry 57(15):6649–55.
1067. Zheng, Zong-Ping et al. 2014. “Characterization of Antiproliferative Activity Constituents from Artocarpus Heterophyllus.” Journal of agricultural and food chemistry 62(24):5519–27.
1068. Zhong, Cheng et al. 2017. “Phenolic Compounds from the Rhizomes of Smilax China L. and Their Anti-Inflammatory Activity.” Molecules (Basel, Switzerland) 22(4).
1069. Zhou, Liang et al. 2013. “Inhibition of Vascular Endothelial Growth Factor-Mediated Angiogenesis Involved in Reproductive Toxicity Induced by Sesquiterpenoids of Curcuma Zedoaria in Rats.” Reproductive toxicology (Elmsford, N.Y.) 37:62–69.
1070. Zhou, Yun et al. 2007. “Triterpene Saponins from Bacopa Monnieri and Their Antidepressant Effects in Two Mice Models.” Journal of natural products 70(4):652–55.
1071. Zhu, Jianhua, Mingxuan Wang, Wei Wen, and Rongmin Yu. 2015. “Biosynthesis and Regulation of Terpenoid Indole Alkaloids in Catharanthus Roseus.” Pharmacognosy reviews 9(17):24–28.
1072. Zhu, Kexue et al. 2017. “Physicochemical Properties and in Vitro Antioxidant Activities of Polysaccharide from Artocarpus Heterophyllus Lam. Pulp.” Carbohydrate polymers 155:354–61.
1073. Zhu, N. et al. 2001. “Furanosesquiterpenoids of Commiphora Myrrha.” Journal of natural products 64(11):1460–62.
1074. Zhu, Wenyi, Yijie Du, Hong Meng, Yinmao Dong, and Li Li. 2017. “A Review of Traditional Pharmacological Uses, Phytochemistry, and Pharmacological Activities of Tribulus Terrestris.” Chemistry Central journal 11(1):60.
1075. Zia-Ul-Haq, Muhammad, Sanja Cavar, Mughal Qayum, Imran Imran, and Vincenzo de Feo. 2011. “Compositional Studies: Antioxidant and Antidiabetic Activities of Capparis Decidua (Forsk.) Edgew.” International journal of molecular sciences 12(12):8846–61.
1076. Zia-Ul-Haq, Muhammad, Milan S. Stankovic, Komal Rizwan, and Vincenzo De Feo. 2013. “Grewia Asiatica L., a Food Plant with Multiple Uses.” Molecules (Basel, Switzerland) 18(3):2663–82.
1077. Zuo, Ai-Xue et al. 2012. “Two New Triterpenoid Glycosides from Curculigo Orchioides.” Journal of Asian natural products research 14(5):407–12.
